# Charge transport through linear carbon atomic chains

**DOI:** 10.1038/s41557-026-02175-w

**Published:** 2026-06-12

**Authors:** James M. F. Morris, Jarred Potter, Elena Gorenskaia, R. Tom Abram, Masnun Naher, Chiara E. Spano, Roberto Listo, Eloise L. Dixon, Amit Sil, Élodie Rousset, Simon J. Higgins, Richard J. Nichols, Paul J. Low, Andrea Vezzoli

**Affiliations:** 1 Department of Chemistry, University of Liverpool, Liverpool, UK; 2 School of Molecular Sciences, University of Western Australia, Crawley, Western Australia Australia; 3 Department of Electronics and Telecommunications, Politecnico di Torino, Corso Duca degli Abruzzi, Torino, Italy

**Keywords:** Electron transfer, Electronic materials, Molecular electronics, Electronic properties and materials

## Abstract

The linear carbon allotrope carbyne has been predicted to display outstanding electrical and mechanical properties, but its preparation and characterization are hindered by synthetic challenges. Although oligoyne and [*n*]cumulene models of carbyne have been explored, the end-groups used to avoid decomposition have a profound effect on their electronic configuration. Here we show that transmetallation of linear carbon fragments from Au(I) species to Au(0) electrodes delivers stable Au|CC…CC|Au devices. Scanning tunnelling microscope break junction techniques were used to characterize charge-transport behaviour in these one-dimensional carbon chains (up to 16 atoms) free of end-capping groups. Shorter chains exhibited oligoyne-like behaviour, with conductance attenuation as a function of length, whereas longer chains show evidence of bond-length equalization towards a cumulenic structure, with remarkably enhanced charge transport. The direct contact between the electrode and the carbon fragment at the Au|C interfaces grant high conductance and quasi-ballistic transport to one-dimensional carbon chains, providing a pathway to advanced carbon-based nanoelectronics based on the stabilization of carbyne within the junction environment.

**Figure:**
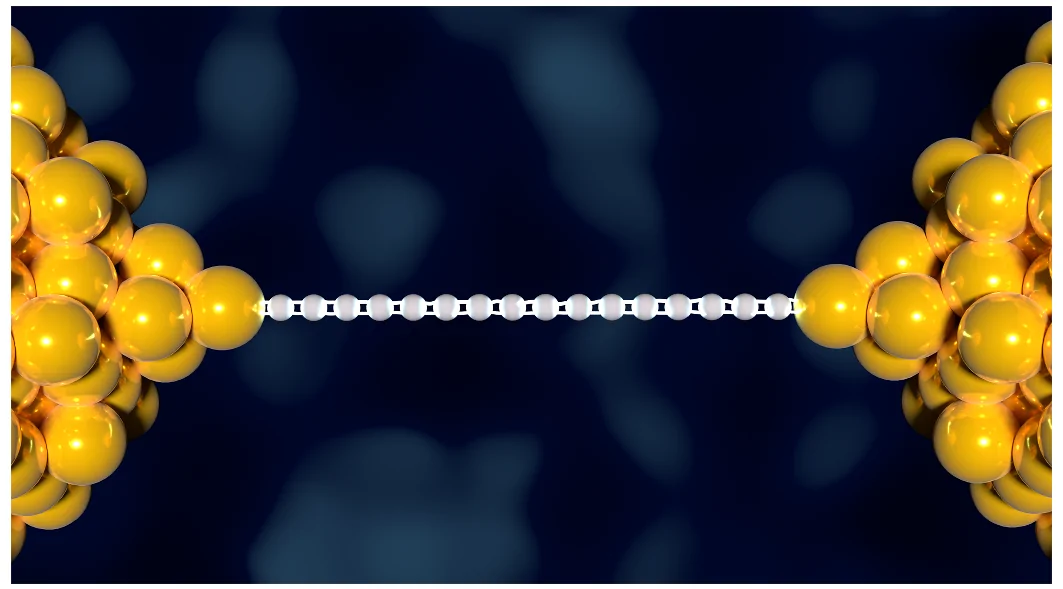

## Abstract (web summary)

Extreme reactivity has long hindered the study of linear atomic carbon chains (carbyne). Now it has been shown that transmetallation of suitable precursors overcomes stability issues, delivering 1D Au|**C*n***|Au junctions that sustain high currents through a cumulene ⋯C=C=C=C⋯ valence structure with 12 or more carbon atoms and enabling their potential for 1D electronics.

## Main

**Fig. 1:**
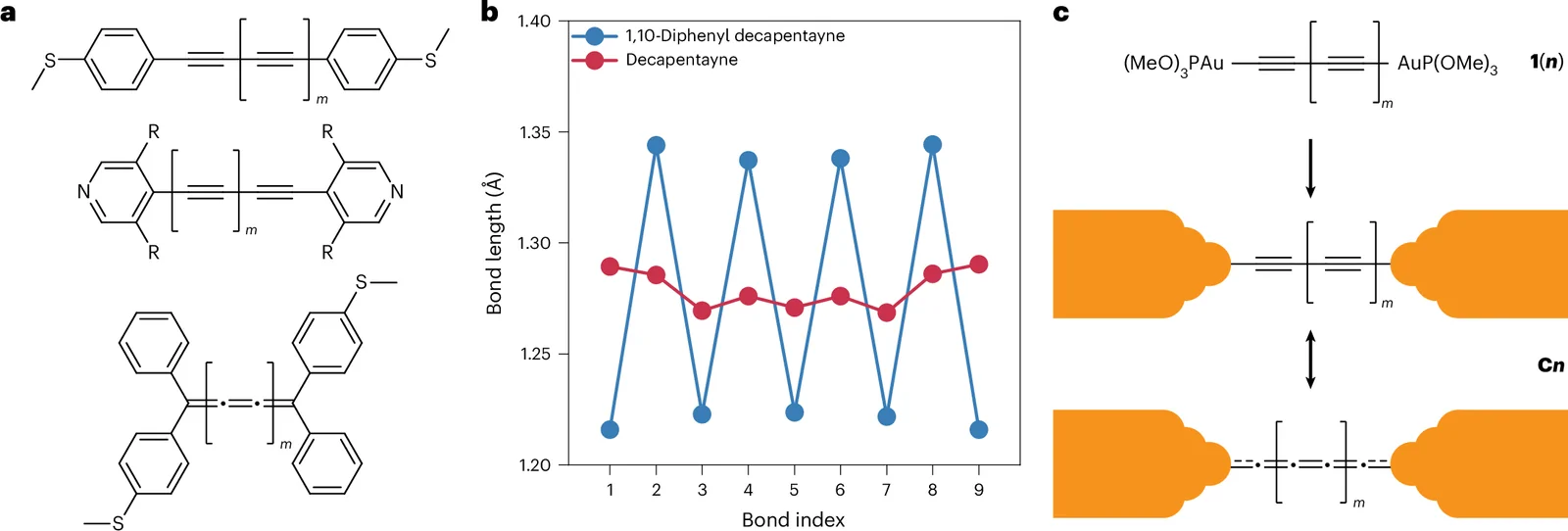
Scope and rationale for this study. **a**, Examples of molecular wires incorporating linear carbon atomic chains in their structure, featuring aryl capping units to grant greater bench stability than the corresponding H-terminated oligoyne. With R = 3,5-di-*tert*-butylphenyl, bench-stable pyridyl-capped oligoynes of up to 48 C atoms have been synthesized^16^. **b**, DFT calculations on bond length between neighbouring carbons in decapentayne derivatives Ph(C≡C)_5_Ph and H(C≡C)_5_H. Capping the pentayne chain with phenyl moieties exacerbates BLA^32^. **c**, Our approach to device fabrication, based on transmetallation from the Au(I) precursors **1**(***n***) (where *n* is the number of carbon atoms in the chain, that is, *n* = 2*m* + 2) to the electrodes Au(0) to deliver **C*n*** devices, with charge transfer (for example, via *d*–π* backdonation, which becomes more important for the longer carbon chains) promoting an equalized, cumulenic structure.

The linear one-dimensional (1D) carbon allotrope carbyne, which might be best described as a chain of *sp*-hybridized carbon atoms, has attracted interest from both theoretical and experimental points of view. Structurally and electronically distinct from the two-dimensional (2D) array of *sp*^2^-hybridized carbon in graphene and the more three-dimensional arrangements of fullerenes and diamond^1^, carbyne has been predicted to have outstanding mechanical, thermal, spectroscopic and electrical properties^2^, and its putative presence in the interstellar medium^3–5^ has contributed to interest in this material over a vast range of scientific disciplines. Such linear chains of *sp*-hybridized carbon atoms, which offer either cumulene (=C=C=C=) or oligoyne (–C≡C–C≡C–) valence structure, represent the most archetypal molecular wire^6^, having a continuous distribution of electron density over a truly 1D backbone^7^. The cumulene form is predicted to have a metallic nature and transport properties independent of the overall length, whereas the alternating single and triple bonds of the oligoyne form would give semiconducting behaviour with transport efficiency decaying exponentially with length^8^. The structure of linear atomic carbon chains is therefore best described by the bond-length alternation (BLA) parameter, with $\mathrm{BLA}=0$ representing the pure cumulene (that is, a chain of equally spaced carbon atoms, the separation of which is consistent with a C=C valence description) and non-zero values describing some degree of alternating short/long bonds along the chain (approaching oligoyne character). Different strategies have been developed and applied over the last few decades to the preparation of linear carbon atom chains, but all these methods failed to deliver a truly archetypal system suitable for transport characterization. Physical top-down methods result in no control on the length of the carbon chain^9^, materials contaminated with *sp*^2^ and *sp*^3^ carbon and with poorly defined overall chemical structure^10^. Chemical bottom-up synthesis, although ensuring atomistic control over chain length and wire structure, requires the capping of the carbon chain by end-groups to tame the extreme reactivity of these materials^11–15^ (Fig. 1a), and the terminal substituents have profound effects on the electronic structure and transport characteristics of these carbyne models^16–18^. Although the introduction of a large group at each terminus can be used to stabilize long carbon chains, these end-caps also promote large BLA and oligoyne-like structures. When α,ω-capped oligoyne species have been used to fabricate electronic devices, the bulky end-groups further reduce charge transport efficiency either by extending the overall length or by reducing the coupling of the carbon atomic chain to the electrodes. It is therefore not completely surprising that measurements of the charge-transport properties of aryl-capped models of a 1D carbon chain showed relatively poor conductance ($G < {10}^{-3}{G}_{0}$, where ${G}_{0}$ is the quantum of conductance $\frac{2{{\rm{e}}}^{2}}{h}\cong 77.48\,{\upmu{\mathrm{S}}}$), rapidly decaying with length when synthesized with primarily an oligoyne structure^19,20^.

Long uncapped chains (that is, carbyne), on the other hand, have been predicted to have BLA as low as $0.04\,\mathring{\rm A}$ (Fig. 1b) and, therefore, quasi-cumulenic character^7,21^. Models of the cumulene form of carbyne require two end-caps at each terminus. These [*n*]cumulenes display pronounced odd-even effects with the number of double bonds in the cumulene chain, *n*, and a range of more unusual conductance behaviours^22,23^. For models with an even number of double bonds (that is, an odd number of carbons, such as the [2]cumulene allene), the orthogonal end-groups interrupt end-to-end conjugation and lead to lower through-molecule conductance^22^, albeit with potential to be used to introduce circular currents by careful control of the chirality of the helical frontier orbitals^24^. The molecular conductance of cumulenes with an odd number of double bonds in the backbone (that is, an even number of chain atoms) is remarkably distance independent, which in the [3]- and [5]cumulenes studied to date is attributed to the decrease in highest occupied molecular orbital (HOMO)–lowest unoccupied molecular orbital (LUMO) gap with increasing conjugation that accompanies increasing chain length^22,25^.

Directly interfacing the carbon-atom chain with two metallic electrodes should, in principle, remove these issues. A direct Au|C interface has been shown to be a very efficient anchor for charge transport in a variety of contexts^26–31^, whereas contact of the carbon chain with large electronic reservoirs (such as the metallic electrodes) should promote charge-transfer phenomena that change the electronic configuration to a more equalized (cumulenic) structure^32^. Among the various techniques developed over the years, here, the decision was made to focus on a transmetallation approach (Fig. 1c), where the carbon-metal bond of an organometallic precursor spontaneously cleaves in the presence of the Au electrodes to deliver Au–C covalent bonds. For the carbon chains we investigated, we used Au(I)trimethylphosphite termini, which offer sufficient steric bulk to stabilize oligomers containing up to 16 carbon atoms. The efficient, spontaneous transmetallation of acetylide fragments from Au(C≡CR){P(OMe)_3_} complexes to the electrodes (…C≡C–Au(I) → …C≡C–Au(0)) has been demonstrated in several studies, giving rise to junctions with a variety of structures^33–35^, while the thermal stability of Au(0)-acetylide bonds has been confirmed by a number of studies performed on either Au surfaces^36–38^ or Au nanoparticles/nanoclusters^39,40^. In what follows, the success of this strategy in delivering electronic devices comprised of only the elements of gold and carbon, together with evidence for the formation of additional junctions featuring coordination polymers with Au atoms linking carbon chains into a conductive backbone is demonstrated. Conductance measurements carried out on single-molecule junctions comprised of a single, pure carbon chain, supported by spectroscopic methods (surface-enhanced Raman spectroscopy, SERS), indicate a transition from semiconducting (oligoyne-like) character in the shorter chains to more electronically transparent transport in the longer carbon atomic chains with greater cumulenic character, approaching the metallic behaviour predicted for carbyne. Additional transport measurements and tight-binding calculations further support these results, suggesting that these simple Au|**C*n***|Au devices provide a route to trap and study fragments of the elusive linear carbon allotrope free of end-capping moieties, at room temperature and in the open laboratory environment.

## Results and discussion

### Synthesis

The compounds {(MeO)_3_P}Au{μ-(C≡C)_*x*_}Au{P(OMe)_3_} (**1**(***n***) with *n* = 2*x* = 4, 6, 8, 10, 12 and 16) used in this study were synthesized from reaction of the appropriate trimethylsilyl- or triisopropylsilyl-protected oligoyne with AuCl{P(OMe)_3_} in the presence of potassium fluoride and potassium *tert*-butoxide in a methanol/tetrahydrofuran solvent mixture. Synthetic details, procedures, spectroscopic data and single-crystal X-ray diffraction studies can be found in Supplementary Information, along with a description of the attempts at synthesizing and isolating **1**(**18**) and **1**(**20**).

### Conductance measurements

The charge-transport properties of the Au|**C*n***|Au junctions, obtained by in situ transmetallation of complexes **1**(**4**)–**1**(**16**) to the Au(0) electrodes, was determined using scanning tunnelling microscope break junction (STMBJ) methods^41^ at a bias of 200 mV (scanning tunnelling microscope tip held at ground). In this technique, the scanning tunnelling microscope Au tip is driven into the Au substrate to form a microcontact having conductance ≫*G*_0_ in the presence of the molecular wire of choice as a dilute solution. The tip is then withdrawn at constant rate to thin down the microcontact to a single atom (having $G={G}_{0}$). Further withdrawal ruptures the atomic point contact, delivering two atomically sharp electrodes separated by a nanogap. Molecular wires present in solution or pre-adsorbed on the electrodes^42^ can self-assemble in the freshly formed nanogap (in this case by Au(I) → Au(0) transmetallation), thus generating the molecular junction. The tip is further withdrawn at constant speed to stretch the junction until its rupture. Tip-substrate bias, tip position and current through the device are simultaneously recorded throughout the entire process. For each compound, data were collected from more than 5,000 individual junctions and the results compiled into 1D histograms, showing the distribution of conductance values as a function of ${G}_{0}$ and 2D conductance-displacement density maps to analyse junction evolution and rupture. A detailed protocol and a description of the equipment and materials used in this study are available in Supplementary Section 2.2. Further data analysis and control experiments to investigate Au|C covalent interfaces are available in Supplementary Sections 2.3–2.5.

**Fig. 2:**
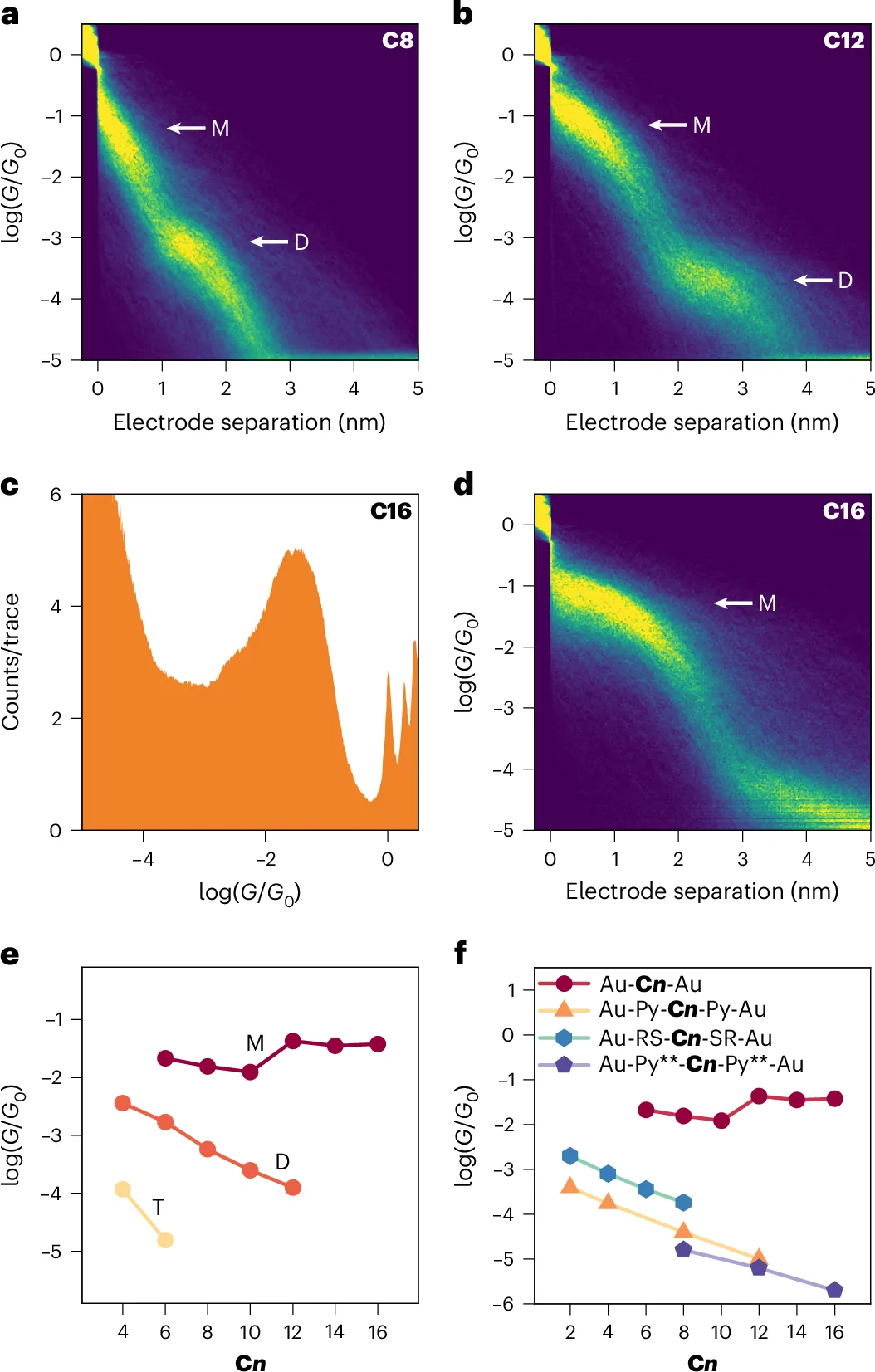
STMBJ charge transport measurements. All measurements illustrated have been performed at 200 mV bias, with the target **C*n*** at 250 μM concentration in anisole. See supporting information for further details, experimental parameters and data analysis protocol. **a**,**b**, Example 2D density maps for junctions formed from **C8** (**a**) and **C12** (**b**) carbon chains. Multiple conductance features are present, attributed to transport through a monomer (M) and a dimer (D) of the target molecular wire. Trimeric (T) junctions are also visible in the shorter **C4** and **C6** chains (see Supplementary Information for details). **c**,**d**, Conductance histogram (**c**) and 2D density map (**d**) for **C16**, noting that in this case, only conductance features of the monomer carbon chain are visible, with the conductance features of the dimer falling below the noise level of the instrumentation. **e**, Conductance versus number of *sp* carbons in the **C*n*** chain measured in this work. **f**, A comparison of the conductance through bare carbon atom chains (this work, red) and values reported in studies on oligoynes capped with aryl units (dimethyldihydrobenzothiophene Au–S(R)^81^, pyridyl Au–Py^19^ and 3,5-bis(3,5-di-*tert*-butylphenyl)pyridyl, Au–Py**)^52^ coordinatively interfacing to the electrodes. In **e** and **f**, *n* = 2*x*. A reliable conductance value for the **C4** monomer could not be determined.

With the exception of **C14** and **C16** (Fig. 2c,d), each of the complexes explored here gave rise to junctions exhibiting multiple conductance features (Fig. 2a,b; see Supplementary Section 2.3 for further data on **C4**, **C6**, **C10** and **C14**). Analysis of the 2D density maps and correlation with the molecular lengths of **C*n*** (estimated either from the C(α)–C(ω) distance in the single-crystal X-ray structures of the corresponding **1**(***n***) precursors or from density functional theory calculations of the postulated species) enabled the attribution of these features to either a monomeric junction or to their oligomers (dimer/trimer) fabricated during the tip withdrawal process by extrusion of small clusters of Au atoms from the electrodes that link the carbon fragments in the growing chain. The formation of oligomeric electrode|molecule–metal–molecule…|electrode species has been described on a number of occasions in the literature^43,44^; the metal (or metal cluster) bridged oligomers formed during tip-retraction in the STMBJ measurement are especially prominent when strong, covalent metal-molecule bonds are used for junction assembly^45–47^. A sequential monomer → dimer → trimer junction evolution process is confirmed by autocorrelation analysis^48–50^, details of which can be found in Supplementary Information. The hypothesis of metal (cluster) bridged oligomer formation is also supported by analysis of the conductance decay with length (Fig. 2e), that within the Landauer formalism should follow the relationship $G\propto {{\rm{e}}}^{-\beta L}$, where L is some measure of the molecular length or number of repeat units in a homologous series, and β is the system-specific attenuation factor versus *L*. The features assigned to the dimers decay at a faster rate (${\beta }_{\mathrm{DIMER}}\cong 0.20\,{\mathring{\rm A} }^{-1}$), than the shorter **C*n*** monomers (vide infra); ${\beta }_{\mathrm{TRIMER}}$ could not be realistically determined as only two datapoints are available. Such behaviour is as expected since the extruded metal cluster(s) that link the carbon chains disrupt *π*-conjugation along the molecular junction^44^, and it rules out alternative mechanisms of dimerization (for example, Au-catalysed oxidative homocoupling of **C*n*** to give **C*n***–**C*n***) that would not deliver such behaviour. Further details and control experiments on the transmetallation mechanism leading to Au|**C*n***|Au (monomer), Au|**C*n***(Au)_*y*_**C*n***|Au (dimer) and Au|**C*n***(Au)_*y*_**C*n***(Au)_*y*_**C*n***|Au (trimer) junctions can be found in Sections 2.5 and 2.6 of Supplementary Section 2.5.

The monomeric junctions Au|**C*n***|Au, on the other hand, do not follow the expected conductance versus distance trendline over all **C*n*** chain lengths. After an initial very shallow decay (${\beta }_{{\mathrm{C}}4-{\mathrm{C}}10}\cong 0.09\,{\mathring{\rm A} }^{-1}$, albeit only three datapoints could be fitted), conductance increases from **C10** to **C12** and remains essentially constant up to **C16** (${\beta }_{{\mathrm{C}}12-{\mathrm{C}}16}\cong 0$) indicative of $\mathrm{BLA}\cong 0$ for *sp* carbon chains^51^. The nonlinearity of conductance versus number of (C≡C) moieties notwithstanding, when the conductance properties of these all-carbon Au|**C*n***|Au junctions are compared with more conventional junctions formed from α,ω-capped oligoynes, Au|R–(C≡C)_*n*_–R|Au (Fig. 1a), the removal of aryl capping units R and associated Au|R interfaces between the linear carbon fragment and the electrodes increases conductance by several orders of magnitude (Fig. 2f). For example, the longest carbon atomic wire we synthesized (**C16**) has charge transport efficiency ($G={10}^{-1.41}\,{G}_{0}$) more than four orders of magnitude greater than its recently reported pyridyl-capped analogue ($G={10}^{-5.70}\,{G}_{0}$)^52^. Even accounting for the overall reduction in molecular wire length, removal of the capping aryl moieties delivers considerable improvements to transport (see Supplementary Fig. 88 for conductance versus molecular wire length comparison). Although the switch to a covalent Au|C interface could in principle explain the high values of conductance observed for the **C*n*** monomer series, which in the case of each chain length examined remains above ${10}^{-2}\,{G}_{0}$, the sudden change in transport efficiency between **C10** and **C12** and the undetectable conductance decay between **C12**, **C14** and **C16** require further interpretation.

As discussed in the introduction, directly interfacing an oligoyndiyl core with the large electronic reservoirs of metallic electrodes can promote a structural transition from the polyyne configuration (…C≡C–C≡C–…) found in the organometallic precursor towards a cumulenic (…C=C=C=C=…) valence structure. Charge transfer from the gold electrodes into the oligoyndiyl backbone is the driving force behind electronic equalization: by populating the ${\pi }^{* }$ antibonding orbital, the triple bonds weaken towards double-bond character, causing an increase in the *π*-character of the neighbouring single bonds. The overall effect is the reduction of BLA, resulting in the near-zero β values observed in the longer **C12**–**C16** series. The propensity for this charge transfer is length-dependent: as the wire grows, the terminal carbon atoms become increasingly electron-deficient, as evidenced by the downfield shift in the ^13^C{^1^H^31^,P} resonance for the terminal C atom (Supplementary Information). Therefore, longer wires experience a more pronounced injection of charge and more extensive electronic equalization. Such *d*–π* backdonation has already been observed in gold cumulenes^53^ and gold allenylidenes^54^, and the latter have been demonstrated to have their allenylidenyl character further enhanced upon photoexcitation (for example, optically populated ${\pi }^{* }$). This behaviour is chemically analogous to the reduction of oligoyndiyl derivatives to the corresponding cumulenes^55,56^ and the inverse electrochemical oxidation of cumulenes, which increases BLA towards an oligoyndiyl structure^57^.

### Theoretical modelling

**Fig. 3:**
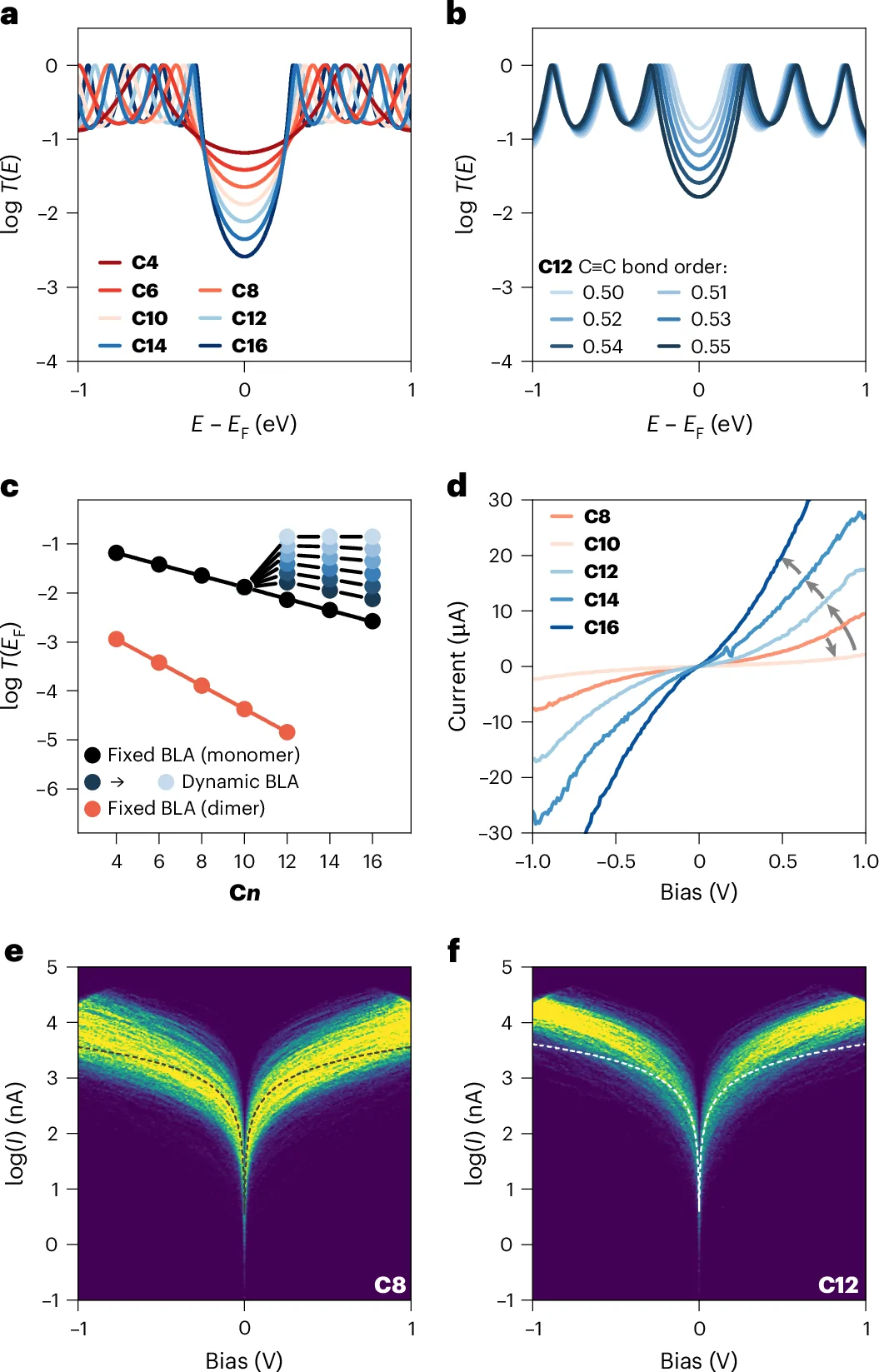
Theoretical calculations and further electrical characterization. **a**, Tight-binding transmission curves for **C4**–**C16** assuming a local bond order (*β*/(*α* + *β*)) of 0.5676 through the entire series as described in the literature^58^. **b**, Tight-binding transmission curves for **C12** over a range of bond orders, from that shown in **a** to a perfect cumulene (that is, bond order of 0.5). **c**, Transmission coefficient at the Fermi level for all compounds measured in this study as both their monomer and the dimeric form. For **C12**–**C16**, the results obtained by changing the bond order to obtain a dynamic BLA are also shown. The latter follows the same colour scheme shown in **b**. **d**, A comparison of the *I*–*V* characteristics for **C8**–**C16** in linear scale, obtained by Gaussian fitting of the corresponding *I*–*V* maps. Arrows included as guide to the eye to follow the evolution of *I*–*V* behaviour across the **C*n*** series. **e**,**f**, Example semilogarithmic *I*–*V* characteristic density map are shown for **C8** (**e**) and **C12** (**f**). The dashed lines in **e** and **f** are the low bias (±50 mV) fittings to the *I*–*V* characteristics (linear regression of linear scale data, intercept set at zero. Slope for **C8** = 1,605 ± 47 nS; slope for **C12** = 4,003 ± 95 nS. Both fittings gave Pearson’s *r* > 0.999 and *R*^2^ > 0.99). *E* in **a** and **b** is the energy of the tunnelling electrons, and *E*_F_ in **a**–**c** is the Fermi level of the electrodes.

Given the known inadequacies of density functional theory (DFT) ab initio methods in modelling linear carbon chains in the presence of metallic surfaces by poorly evaluating $\mathrm{BLA}$^58,59^, we used a simple Hückel model (tight binding with only nearest neighbour interactions) to explore charge transport through the **C*n*** series. We wrote Hamiltonians with one site per atom, with all Coulomb integrals (on-site energy) set to zero. Alternating bonds were then modelled with resonance (hopping) integrals for the single (*α*) and triple (*β*) bonds defined by a maximum value $t=-1.5\,\mathrm{eV}$ multiplied by the infinite polyyne band structure derived bond order for α and β, respectively^58^ (Fig. 3).

When the C≡C (and C–C) bond order(s) and, hence, the BLA are kept invariant throughout the series (see Supplementary Section 4 for details about bond order and BLA relationship), the transmission coefficient at the Fermi level of the electrodes $T({E}_{F})$ decays exponentially with increasing length of the 1D carbon atomic chain (Fig. 3a). Within the Landauer formalism, this means that conductance also should decay exponentially with length, as $G={G}_{0}T\left({E}_{\mathrm{F}}\right)$, in line with the expectation but not the experimental observation. To account for the increased conductance of **C12**–**C16**, the C≡C and C–C bond orders were modified through a dynamic BLA regime towards perfect cumulenic behaviour (where a bond order of 0.5 delivers BLA = 0). As the bond orders along the chain are allowed to equalize and BLA decreases, $T({E}_{\mathrm{F}})$ increases (Fig. 3b). This modification of the molecular and electronic structures leads to good agreement with the experimental conductance results in cumulenic (or quasi-cumulenic) structures of **C12**–**C16**. This agreement includes the conductance plateauing at an upper ceiling after **C12** (Fig. 3c), as a result of the rapid decrease in the HOMO–LUMO gap which offsets electronic decoupling due to the increasing molecular length^23,60,61^. We want to stress here that modifying the bond strength at the molecule–electrode interface is not enough to reproduce the observed behaviour (see Supplementary Section 4.1 for details on the effect of interfacial coupling on the trend). The same tight-binding model calculations for the dimeric junction, including a Au–Au unit between two **C*n*** wires to deliver the ‘dimeric’ junction, correctly followed the experimental data returning a more rapid conductance decay with length than for the pure carbon junctions. The agreement between experiments and the Hückel model confirms a static oligoyndiyl *BLA* regime in the dimers, as the small Au cluster extruded is not capable of sufficient charge transfer to induce electronic equalization to a cumulenic structure.

### *I*–*V* characteristics

The Hückel model therefore predicts that as the **C*n*** fragment within the junction assumes a more cumulenic character, its zero-bias transmission coefficient increases as the HOMO–LUMO gap shrinks rapidly, leading to an increased $T({E}_{\mathrm{F}})$ across the entire transmission spectrum (Fig. 3b). To further explore this phenomenon, the *I*–*V* characteristics of the compounds in the **C8**–**C16** range (**C4** and **C6** are too short for reliable data collection) were measured. Further details of the *I*–*V* measurements and data analysis procedures are given in Supplementary Section 2.7 and elsewhere^60,62,63^. The **C8** and **C10** carbon chains exhibit wide windows of linear *I*–*V* response, with the current supported by the longer **C10** chain lower than the **C8** analogue across the entire bias window ($\pm 1\,{\rm{V}}$), as expected for the tunnelling regime through an oligoyne-like fragment. The differences in these *I*–*V* characteristics are clearly displayed when data are plotted on a linear scale (Fig. 3d, extracted from maps such as those shown in Fig. 3e,f). By contrast, **C12** supports a consistently higher current than both shorter **C8** and **C10** carbon chains across the bias window, supporting the evidence of a higher zero-bias transmission coefficient. The *I*–*V* response of **C12**–**C16** deviates from the linear Ohmic behaviour at increasingly low bias and more rapidly than **C8** and **C10**, providing further evidence of a change in the overall electronic structure of the longer 1D carbon chain, approaching metallic behaviour (that is, quasi-linear *I*–*V* characteristics) in **C16**. This rapid deviation from semiconducting *I*–*V* behaviour starting in **C12** is probably a consequence of the narrowing HOMO–LUMO gap and better alignment of the transport resonances with the Fermi level of the electrodes. These properties combined render the longer **C*n*** wires capable of sustaining very high currents (>40 µA at 1 V source-drain bias, corresponding to ${\sim}{}0.6\,{G}_{0}$ measured in **C16**) over distances exceeding 2 nm. To provide further evidence for the proposed electronic equalization towards a cumulenic structure, as opposed to a simple resonant transport phenomena, such as those observed in diketopyrrolopyrrole molecular wires^64^, that would deliver similar results at sufficiently high bias, we extracted the conductance values over a range of biases from the *I*–*V* curves (G=I/V). The increase in transport efficiency observed in **C12**–**C16** is retained even at biases as low as $5\,\mathrm{mV}$ (see Supplementary Fig. 101 for conductance versus **C*n*** plot), where resonant transport phenomena should be negligible.

### Raman spectroscopy

**Fig. 4:**
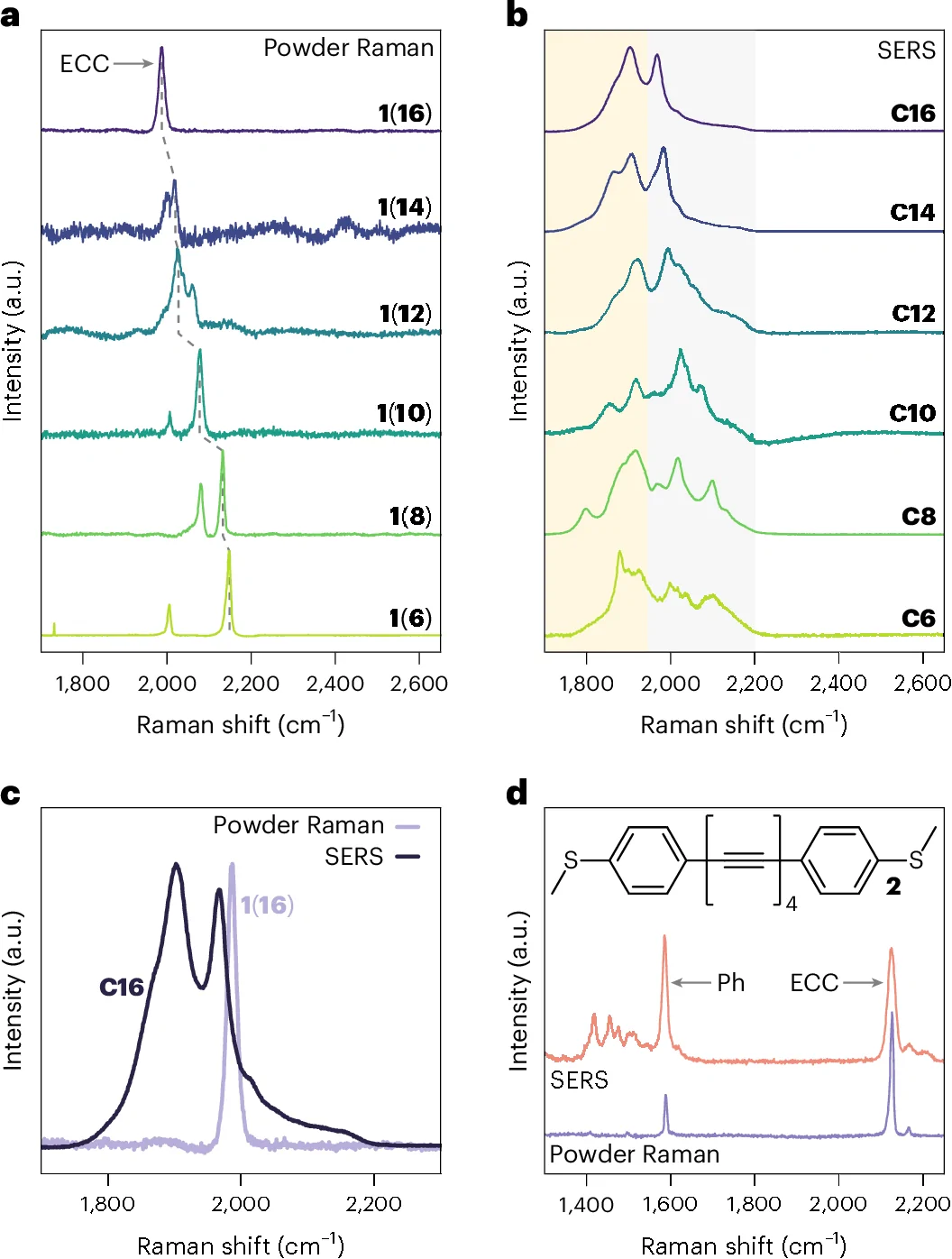
Raman (785 nm) characterization. **a**,**b**, Powder Raman spectra of the precursors **1**(***n***) (**a**) and SERS spectra of the **C*n*** series after transmetallation to Au nanoparticles in the region 1,700–2,650 cm^−1^ (**b**). The dashed line in **a** follows the ECC mode evolution across the **1**(***n***) series, whereas the oligoynyl and cumulene regions of the ECC band in **c** are shaded, respectively, in grey and yellow. **c**, Direct comparison of powder Raman and SERS spectra within the 1,700–2,300 cm^−1^ window. **d**, A comparison of the SERS and powder Raman spectra for the thioanisolyl-capped analogue **2** (structure shown). The signal relative to the ECC oligoynyl mode and the phenyl (Ph) breathing mode are marked with arrows. Spectra in **a**–**c** are normalized to laser intensity. See Supplementary Information for full spectra and additional details.

As further support for the shift from an oligoyne-like backbone towards a more cumulenic structure in these 1D carbon chains contacted between the Au electrodes, attention was turned towards Raman spectroscopy of various model systems. The Raman spectra of solid powder samples of the Au phosphite complexes **1**(**4**)–**1**(**10**) exhibit a strong α line at 2,100–2,200 cm^−1^ (ref. ^65^), corresponding to the effective conjugation coordinate (ECC) or ${\mathscr{R}}$ mode^66^, along with the less intense β line at lower Raman shift (Fig. 4a). These bands are related to different, collective stretching vibrations of *sp*-hybridized carbon–carbon bonds (BLA oscillation modes), and they red shift with increasing **C*n*** due to electron-phonon coupling^67^. In **1**(**12**), **1**(**14**) and **1**(**16**), however, these α- and β-bands start to broaden and weaken, as predicted for more equilibrated 1D carbon chains that have a reduced BLA^21^, suggesting that the longer carbon chains in these molecular models may begin to approach the carbyne limit^16^.

Intrigued by these initial results on the solid-state materials **1**(***n***), attention was turned to model constructions using Au nanoparticles as both ‘surrogates’ for the metallic electrodes and plasmonic enhancers to permit interrogation of the end-cap free carbon chains by SERS, taking advantage of the facile transmetallation of the **C*n*** fragment from the Au(I) phosphite complexes **1**(***n***) to Au(0) nanoparticle(s). Samples of ~30–60-nm diameter citrate-protected nanoparticles^68^ were drop-cast onto a Si wafer and then treated with a dilute (0.25 mM in acetone) solution of the complex **1**(***n***) containing the carbon chain of interest, followed by gentle rinsing with fresh solvent and drying to remove all solvent traces. This pseudo solid-state procedure^65^ for the preparation of the SERS samples using immobilized Au(0) nanoparticles was preferred to solution-based methods to avoid aggregation-induced decomposition of the longer complexes (**1**(**12**) and **1**(**16**)) in colloidal nanoparticle suspensions^69^. Evidence of transmetallation can be found in the SERS spectra as a broad C–Au(0) stretching signal at 478 cm^−1^ (absent in the powder Raman of the **1**(***n***) precursors; Supplementary Fig. 104), observed in all samples and consistent with the literature^70^. This signal is remarkably blue shifted from that observed in alkyl-Au (ref. ^71^) or phenyl-Au (ref. ^72^) interfaces obtained by electrografting of diazonium precursors or in directly adsorbed phenylacetylene^73^ (alkyl: 387 cm^−1^; phenyl: 413 cm^−1^; phenylacetylene: 405 cm^−1^), reflecting the stronger nature of the Au–oligoynyl interface. In each networked assembly, the α and β ECC bands are severely broadened in the SERS, with the oligoyne ${\mathscr{R}}$ mode resonances observed at 2,000–2,200 cm^−1^ in **C6**–**C16** accompanied by additional bands at 1,800–1,900 cm^−1^ attributable to a quasi-cumulenic structure with a small HOMO–LUMO gap^74^ (Fig. 4b). These latter features, which are observed for all chain lengths examined, may be due to portions of the network with charge transfer induced by charged nanoparticles being much stronger than that happening with the ‘neutral’ electrodes used in STMBJ measurements, mirroring similar results obtained using Ag nanoparticles as SERS substrates^75,76^. The broadness and poor definition of these features arise from the relative disorder of the networked structures and the lack of control over the orientation of the **C*n*** fragments relative to the Au nanoparticle surface normal and, therefore, on the greater chain-to-chain variation in the degree of charge transfer and electronic equilibration of the 1D carbon chain and its alignment with the oscillating electric field of the plasmonic resonance with the network. Most importantly, in the longest carbon chain **C16** the band at $1,880\,{\mathrm{cm}}^{-1}$ is clearly defined and stronger in intensity than the ${\mathscr{R}}$ mode, in excellent agreement with previous results on encapsulated carbyne^77^. It should be noted that broad features also appear at $1,600\,{\mathrm{cm}}^{-1}$, in the *ν*(C=C) stretching region (see Supplementary Fig. 103 for full spectra), reminiscent of the multiple signals exhibited by cumulenes synthesized *ex situ*^78^ and already observed in encapsulated linear carbon chains as a broad shoulder on the nanotube G-band^77^. The broad SERS shape is consistent with the presence of electronic defects and mechanical kinks in the 1D carbon chain^79^, again arising from the intrinsic disorder of the nanoparticle–**C*n*** network structures, although laser-induced cross-linking cannot be completely discounted in spite of the low-energy photons ($785\,\mathrm{nm},\, {\sim}{1.58}\,\mathrm{eV}$) used in this study^80^. As a control experiment, the same experiment was performed using the α,ω-thioanisolyl-capped octatetrayne **2** (ref. ^81^). In this compound, the aromatic capping group should preserve an oligoyne structure by reducing charge transfer from/to the nanoparticle and by constraining BLA oscillations. Our results (Fig. 4d) indeed show very little broadening and red-shifting of the SERS signals, confirming the unique electronic structure of the **C*n*** 1D carbon atomic chains when directly coupled to metallic electron reservoirs by transmetallation.

## Conclusion

Through transmetallation chemistry, 1D carbon chains can be directly interfaced to metallic electrodes to yield Au|**C*n***|Au electrical junctions, delivering a truly archetypal electronic device made of only two elements: gold and carbon. When the number of carbon atoms is low (<10) a very weak attenuation of charge transport efficiency with length is observed, consistent with an oligoynyl electronic structure and alternation between short (C≡C) and long (C–C) bonds. Above a critical value of 10 C atoms, however, conductance suddenly increases and maintains high low-bias values (~3 μS), even as the 1D carbon chain is made longer. Theoretical modelling and a series of charge transport and spectroscopic experiments support the attribution of these phenomena to electronic equalization towards a cumulenic (…C=C=C=C…) structure, facilitated by the presence of the electrodes as large electronic reservoirs. The longer 1D carbon chains present some unique properties such as non-uniform conductance decay with increasing chain length and very efficient charge transport, capable of sustaining currents as high as 40 μA over distances >2 nm (among the highest ever reported at such length scales, see Supplementary Section 6 for comparison), approaching the quantum of conductance and thereby providing a pathway to length-independent, quasi-ballistic transport in non-metallic nanostructures. The electrical properties of these longer chains are consistent with many predictions of the 1D carbon allotrope carbyne, and the prospects for use of this chemistry to prepare and further study samples within device-like structures, free of end-caps that characterize molecular oligoyne and cumulene models of this elusive species, are tantalising.

## Methods

Caution: all polyyne compounds should be treated as potentially explosive. All reactions were carried out under inert atmosphere, using Schlenk techniques. All compounds were characterized by multinuclear nuclear magnetic resonance, Fourier-transform infrared spectroscopy, HRMS, Raman and UV–Vis spectroscopy. Single-crystal X-ray diffraction was used to structurally characterize **1**(**4**) and **1**(**6**). Single-molecule charge transport properties have been measured with a bespoke scanning tunnelling microscope. Detailed methods, instrumentation, experimental procedures, data analysis protocols and the theoretical framework used for the interpretation of the data are reported in Supplementary Information. See Supplementary Section 1 for synthesis and characterization of the **1**(***n***) species, Supplementary Section 2 for the scanning tunnelling microscopy measurements, Supplementary Section 3 for Raman spectroscopy and Supplementary Section 4 for theoretical modelling.

## Online content

Any methods, additional references, Nature Portfolio reporting summaries, source data, extended data, supplementary information, acknowledgements, peer review information; details of author contributions and competing interests; and statements of data and code availability are available at https://doi.org/10.1038/s41557-026-02175-w.

## Supplementary information


Supplementary InformationSupplementary Figs. 1–107, Tables 1–8 and Discussion.


## Data Availability

All data acquired in Liverpool is available via the University of Liverpool Data Catalogue as entry #3014 at https://doi.org/10.17638/datacat.liverpool.ac.uk/3014 (ref. ^82^). Crystallographic data for the structures reported in this Article have been deposited at the Cambridge Crystallographic Data Centre, under deposition numbers CCDC 2440861 (1(**4**)) and 2440860 (1(**6**)). Copies of the data can be obtained free of charge at https://www.ccdc.cam.ac.uk/structures.
