# Supplementary material for: Charge transport through linear carbon atomic chains
Source: Nat Chem. 2026 Jun 12;18(9):1574–81. doi: 10.1038/s41557-026-02175-w (PMC13524795; doi:10.1038/s41557-026-02175-w)
Supplement: Supplementary file 1 [file 41557_2026_2175_MOESM1_ESM.pdf]

nature chemistry

Supplementary information

https://doi.org/10.1038/s41557-026-02175-w

Charge transport through linear carbon atomic chains

In the format provided by the authors and unedited

Contents

1. Synthesis 2

1.1 General Procedures 2

1.2 Reagents 2

1.3 Instruments 2

1.4 X-ray Data Collection and Structure Solution 2

1.5 Synthetic Procedures 3

1.6 Plots of NMR spectra and summaries of data. 17

1.7 UV-Vis Absorption Spectra 52

1.8 Single Crystal X-ray Diffraction Data 54

2. STM Measurements 58

2.1 Materials 58

2.2 Instrumentation 58

2.3 Additional STMBJ Data 59

2.4 Calculation of most probable conductance value 63

2.5 Additional Data Analysis 64

2.6 Additional Control Experiments 67

2.7 $I-V$ Measurements 69

2.8 STMBJ and $I-V$ Statistics 71

3. Raman & SERS Spectroscopy 72

3.1 Additional Raman Spectra 72

4. Details on Tight-Binding (Hückel) Calculations 74

4.1 Supporting Tight-Binding (Hückel) calculations 76

5. Density Functional Theory Calculations 77

6. Literature Survey 79

7. Supporting Information References 80

1. Synthesis

CAUTION: All polyyne compounds should be treated as potentially explosive. Care should be taken when handling, transferring, and heating solid samples. Attempts to extend the methodologies reported below to the putative compound 1(20) gave a product that exploded within a sintered funnel when scratched with a metal spatula.

1.1 General Procedures

All reactions were undertaken under nitrogen atmosphere using Schlenk techniques, unless otherwise stated. No particular care was taken to exclude air upon work-up of reaction products. Reaction flasks were heated under vacuum and cooled under nitrogen atmosphere three times prior to use. Solvents were deoxygenated by standard procedures. For column chromatography, silica with a particle size of 60 μm was used.

1.2 Reagents

Tetrahydrofuran, dichloromethane and hexanes were purified and dried on an Inert PureSolv Micro Solvent Purification System and stored over 3 Å molecular sieves. Methanol was dried by distillation over magnesium and iodine. All other solvents were obtained from commercial suppliers and used as received. The compounds 1,4-bis(trimethylsilyl)-1,3-butadiyne,[1] 1,6-bis(trimethylsilyl)-1,3,5-hexatriyne,[2] 1-(trimethylsilyl)-buta-1,3-diyne,[3] 1,10-bis(triisopropylsilyl)-1,3,5,7,9-decapentayne,[4] 3-(dibromomethylidene)-1,5-bis(trimethylsilyl)penta-1,4-diyne,[5] 3-(triisopropylsilyl)propiolaldehyde,[6] 3-triisopropylsilylpropynoic acid,[7] 1,12-bis(triisopropylsilyl)-1,3,5,7,9,11-dodecahexayne,[4] 1,16-bis(triisopropylsilyl)-1,3,5,7,9,11,13,15-hexadecaoctayne,[4] 5-triisopropylsilylbutadiynoic acid,[8] 1,20-bis(triisopropylsilyl)-1,3,5,7,9,11,13,15,17,19-eicosadecayne,[4] 1,4-bis[(trimethylsilyl)ethynyl]-phenylene,[9] 4-ethynylthioanisole,[10] and 4-[(trimethylsilyl)ethynyl]toluene[11] were synthesised according to literature methods. All other materials were obtained from commercial suppliers and used as received.

1.3 Instruments

NMR spectra were recorded in deuterated chloroform solution on a Bruker Avance 400 MHz (^{1}H: 400.15 MHz, ^{13}C{^{1}H}: 100.62 MHz, ^{31}P{^{1}H}: 161.98 MHz), 500 MHz (^{1}H: 500.10 MHz, ^{13}C{^{1}H}: 125.75 MHz, ^{31}P{^{1}H}: 202.44 MHz), or 600 MHz (^{13}C{^{1}H,^{31}P}: 150.90 MHz) spectrometer and referenced against residual proteo-solvent resonances ($CHCl_3$: ^{1}H 7.26 ppm, ^{13}C{^{1}H}/^{13}C{^{1}H,^{31}P} 77.16 ppm) or by the ^{2}H solvent lock signal. For all NMR spectra, second order multiplets are reported according to their closest first order approximation. FT-IR spectra were recorded on an Agilent Technologies Cary 630 FTIR spectrometer using ATR sampling methods. High-resolution mass spectra (HR-MS) were recorded using a Waters LCT Premier XE or Thermo Scientific Orbitrap Exploris 120 mass spectrometer using positive mode electrospray ionization (ESI+). Samples were prepared in MeCN and inserted by direct injection via the on-board injector. UV-Vis absorption spectra were collected in CH_2Cl_2 using an Agilent Technologies Cary 5000 UV-Vis-NIR spectrometer and were normalised by scaling the spectra such that the relative absorbance of λ_{max} = 1.0.

1.4 X-ray Data Collection and Structure Solution

Data were collected with a Rigaku-Oxford Diffraction XtaLAB Synergy-S diffractometer using a micro-focus sealed Cu Kα radiation (λ = 1.54184 Å) X-ray source for all compounds. The compounds were slightly light-sensitive and therefore were mounted relatively quickly with precaution, to minimise the magnitude of light exposure. All data were reduced using the CrysAlis^{PRO} software. Using Olex2,[12] the molecular structures were

solved by intrinsic phasing using SHELXT[13] and refined by full-matrix least-squares procedures on F^2 using SHELXL.[14] All non-hydrogen atoms were refined anisotropically and a riding model was employed in the treatment of the hydrogen atom positions. Graphics of the molecular structures have been created by using Diamond.† Further crystallographic data have been deposited at the Cambridge Crystallographic Data Centre. Copies of the data with CCDC numbers 2440860 and 2440861 can be obtained free of charge via https://www.ccdc.cam.ac.uk/structures/, or from the Cambridge Crystallographic Data Centre, 12 Union Road, Cambridge CB2 1EZ, U.KCB21EZ, UK (fax +441223336033; email deposit@ccdc.cam.ac.uk).

1.5 Synthetic Procedures

Chloro(tetrahydrothiophene)gold(I) AuCl(SC_4H_8)

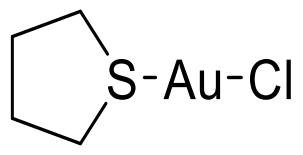

Synthesised according to modified literature procedures.[15,16] A solution of aqua regia (20 mL, 3:1 32% HCl solution:70% HNO_3 solution) was poured onto 99.99 % purity gold pieces (4.5 g, 22.8 mmol) and the mixture was allowed to react overnight. The resulting bright orange solution was heated to 80 °C and a gentle stream of nitrogen blown through the flask to evaporate the solution. Upon complete evaporation, an additional charge of 32 % HCl solution (6 mL) was added and the solution evaporated again. This process was repeated a total of three times to ensure complete consumption of HNO_3. After the final evaporation, the resulting bright orange $HAuCl_3$•XH_2O crystals were dissolved in a 5:1 EtOH:H_2O solution (48 mL). Tetrahydrothiophene (4.20 mL, 4.22 g, 47.9 mmol) was slowly added while stirring at room temperature. The reaction initially formed a yellow precipitate which transitioned to white. After complete addition the mixture was stirred for 30 min and then filtered, washing with EtOH (3 × 10 mL). The resulting solid was dried under vacuum to obtain the product as a white powder (7.12 g, 97 %).

^{1}H NMR ($CDCl_3$, 400 MHz): δ 2.20 (br d, J = 70 Hz, 4H, SC_4H_8); 3.41 (br d, J = 70 Hz, 4H, SC_4H_8) ppm. ^{13}C{^{1}H} NMR ($CDCl_3$, 101 MHz): δ 30.7 (SC_4H_8), 39.8 (br, SC_4H_8) ppm. The spectroscopic data is in accordance with literature values.[15]

Chloro(trimethylphosphite)gold(I) AuCl{$P(OMe)_3$}

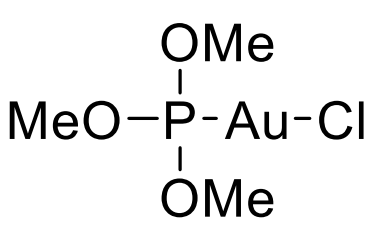

Synthesised according to modified literature procedure.[17] To a dry, degassed solution of chloro(tetrahydrothiophene)gold(I) (0.50 g, 1.6 mmol) in tetrahydrofuran (25 mL) was added trimethylphosphite (0.19 mL, 0.20 g, 1.64 mmol) and the mixture stirred for 2 hr at room temperature. The

† Crystal Impact Diamond https://www.crystalimpact.com/diamond/ [retrieved 15/11/2024]

volatiles were removed and the sample dried under vacuum (1.5 mbar) for 2 hr to afford the product as a white solid (0.54 g, 98 %).

^{1}H NMR ($CDCl_3$, 400 MHz): δ 3.78 (d, J = 14.0 Hz, 9H, OMe) ppm. ^{13}C{^{1}H} NMR ($CDCl_3$, 101 MHz): δ 53.4 (OMe) ppm. ^{31}P{^{1}H} NMR ($CDCl_3$, 162 MHz): δ 120.6 ppm (unreferenced). The spectroscopic data is in accordance with literature values.[17]

1,4-Bis[(trimethylphosphite)gold(I)]-1,3-butadiyne 1(4)

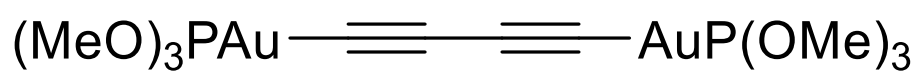

To a dry, degassed solution of 1,4-bis(trimethylsilyl)-1,3-butadiyne (75 mg, 0.39 mmol) and AuCl{P(OMe)$_3$} (275 mg, 0.77 mmol) in a mixture of MeOH (10 mL) and tetrahydrofuran (10 mL) was added KF (44 mg, 0.77 mmol) and KO^{t}Bu (86 mg, 0.77 mmol) and the resulting mixture stirred for 2 hr at room temperature. The solvent was removed under vacuum and the residue sonicated in cold MeOH (~10 mL). The resulting mixture was filtered, and the collected material washed with cold MeOH (10 mL) and hexanes (10 mL), to afford the product as a white solid (152 mg, 57 %).

IR (ATR, $\tilde{\nu}$): ν(C≡C) 2011 cm^{-1}. ^{1}H NMR ($CDCl_3$, 400 MHz): δ 3.73 (d, J =13.4 Hz, 18H, OMe) ppm. ^{13}C{^{1}H} NMR ($CDCl_3$, 101 MHz): δ 52.8 (OMe) ppm. The acetylenic carbons are not observed. ^{13}C{^{1}H,^{31}P} ($CDCl_3$, 151 MHz): δ 52.8 (OMe), 88.6 (Au–C≡*C*), 121.2 (Au–*C*≡C) ppm. ^{31}P{^{1}H} NMR ($CDCl_3$, 162 MHz): δ 149.3 ppm (unreferenced). HR-MS (ESI(+)) *m*/*z*: calcd for $C_{10}H_{18}Au_2O_6P_2$+H 690.9982; found 690.9989 [M+H]$^+$.

1,6-Bis[(trimethylphosphite)gold(I)]-1,3,5-hexatriyne 1(6)

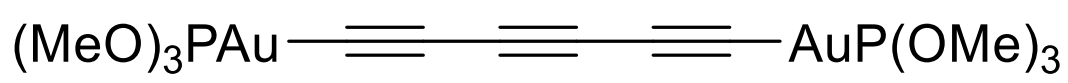

To a dry, degassed solution of 1,6-bis(trimethylsilyl)-1,3,5-hexatriyne (157 mg, 0.72 mmol) and AuCl{P(OMe)$_3$} (512 mg, 1.44 mmol) in a mixture of MeOH (15 mL) and tetrahydrofuran (15 mL) was added KF (53 mg, 0.92 mmol) and KO^{t}Bu (103 mg, 0.92 mmol) and the resulting mixture stirred for 2 hr at room temperature. The solvent was removed under vacuum and the residue sonicated in MeOH (~10 mL). The resulting mixture was filtered, and the collected material washed with MeOH (10 mL) and hexanes (10 mL), to afford the product as a pale-yellow solid (132 mg, 26 %).

IR (ATR, $\tilde{\nu}$): ν(C≡C) 2107 cm^{-1}. ^{1}H NMR ($CDCl_3$, 400 MHz): δ 3.74 (d, J =13.4 Hz, 18H, OMe) ppm. ^{13}C{^{1}H} NMR ($CDCl_3$, 101 MHz): δ 52.9 (OMe), 57.8 (C_{sp}) ppm. All other acetylenic carbons are not observed. ^{13}C{^{1}H,^{31}P} ($CDCl_3$, 151 MHz): δ 52.9 (OMe), 57.8 (C_{sp}), 88.8 (Au–C≡*C*), 126.6 (Au–*C*≡C) ppm. ^{31}P{^{1}H} NMR ($CDCl_3$, 162 MHz): δ 147.6 ppm (unreferenced). HR-MS (ESI(+)) *m*/*z*: calcd for $C_{12}H_{18}Au_2O_6P_2$+H 714.9982; found 714.9988 [M+H]$^+$.

1,8-Bis(trimethylsilyl)-1,3,5,7-octatetrayne

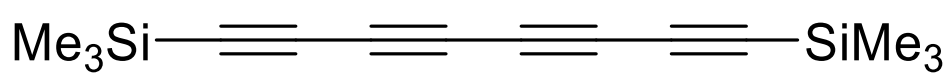

Synthesised according to modified literature procedures.[1,18] A three-necked flask fitted with a −78 °C condenser was charged with a mixture of CuI (24 mg, 0.12 mmol) and 4-dimethylaminopyridine (30 mg, 0.25 mmol) in acetonitrile (10 mL), and purged with O_2 for 10 min. Then, 1-(trimethylsilyl)-buta-1,3-diyne (300 mg, 2.45 mmol) was added and the mixture was stirred under an O_2 atmosphere for 1 hr. The solvent was evaporated in vacuo and the crude residue was dissolved in hexanes (~50 mL) and filtered through a silica plug, eluting with additional volumes of hexanes. The eluent was evaporated to obtain an orange solid (150 mg, 49 %) which was used without further purification. ^{1}H NMR ($CDCl_3$, 400 MHz): δ 0.21 (s, 18H, $SiMe_3$) ppm. The spectroscopic data is in accordance with literature values.[19]

1,8-Bis[(trimethylphosphite)gold(I)]-1,3,5,7-octatetrayne 1(8)

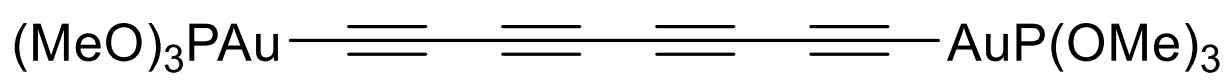

To a dry, degassed solution of 1,8-bis(trimethylsilyl)-1,3,5,7-octatetrayne (41 mg, 0.17 mmol) and AuCl{P(OMe)$_3$} (120 mg, 0.34 mmol) in a mixture of MeOH (5 mL) and tetrahydrofuran (5 mL) was added KF (20 mg, 0.34 mmol) and KO^{t}Bu (38 mg, 0.34 mmol) and the resulting mixture stirred for 2 hr at room temperature. The solvent was removed under vacuum and the residue sonicated in MeOH (~10 mL). The resulting mixture was filtered, and the collected material washed with MeOH (10 mL) and hexanes (10 mL), to afford the product as a yellow solid (90 mg, 64 %).

IR (ATR, $\tilde{\nu}$): ν(C≡C) 2002, 2146 cm^{-1}. ^{1}H NMR ($CDCl_3$, 400 MHz): δ 3.75 (d, J = 13.4 Hz, 18H, OMe) ppm. $^{13}C\{^{1}H\}$ NMR ($CDCl_3$, 101 MHz): δ 52.9 (OMe), 58.0 (C_{sp}), 61.1 (C_{sp}) ppm. All other acetylenic carbons are not observed. $^{13}C\{^{1}H,^{31}P\}$ ($CDCl_3$, 151 MHz): δ 52.9 (OMe), 58.0 (C_{sp}), 61.1 (C_{sp}), 88.0 (Au–C≡*C*), 129.2 (Au–*C*≡C) ppm. $^{31}P\{^{1}H\}$ NMR ($CDCl_3$, 162 MHz): δ 146.8 ppm (unreferenced). HR-MS (ESI(+)) *m*/*z*: calcd for $C_{14}H_{18}Au_2O_6P_2$+H 738.9982; found 738.9987 [M+H]$^+$.

1,10-Bis[(trimethylphosphite)gold(I)]-1,3,5,7,9-decapentayne 1(10)

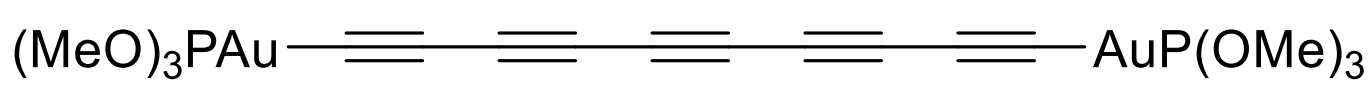

To a dry, degassed solution of 1,10-bis(triisopropylsilyl)-1,3,5,7,9-decapentayne (30 mg, 0.07 mmol) and AuCl{P(OMe)$_3$} (53 mg, 0.15 mmol) in a mixture of MeOH (15 mL) and tetrahydrofuran (5 mL) was added KF (10 mg, 0.18 mmol) and KO^{t}Bu (20 mg, 0.18 mmol) and the resulting mixture stirred for 2 hr at room temperature. The solvent was removed under vacuum and the residue sonicated in MeOH (~10 mL). The resulting mixture was filtered, and the collected material washed with MeOH (10 mL) and hexanes (10 mL), to afford the product as a yellow-brown solid (19 mg, 36 %).

IR (ATR, $\tilde{\nu}$): ν(C≡C) 1996, 2061, 2151 cm^{-1}. ^{1}H NMR ($CDCl_3$, 400 MHz): δ 3.75 (d, J = 13.4 Hz, 18H, OMe) ppm. $^{13}C\{^{1}H\}$ NMR ($CDCl_3$, 101 MHz): δ 52.9 (OMe), 57.4 (C_{sp}), 61.3 (C_{sp}), 62.3 (C_{sp}) ppm. All other acetylenic carbons are not observed. $^{13}C\{^{1}H,^{31}P\}$ ($CDCl_3$, 151 MHz): δ 52.9 (OMe), 57.4 (C_{sp}), 61.3 (C_{sp}), 62.3 (C_{sp}), 87.7

(Au–C≡*C*), 130.9 (Au–*C*≡C) ppm. $^{31}P\{^1H\}$ NMR ($CDCl_3$, 162 MHz): *δ* 146.3 ppm (unreferenced). HR-MS (ESI(+)) *m*/*z*: calcd for $C_{16}H_{18}Au_2O_6P_2$+H 762.9982; found 762.9974 [M+H]$^+$.

1,12-Bis[(trimethylphosphite)gold(I)]-1,3,5,7,9,11-dodecahexayne 1(12)

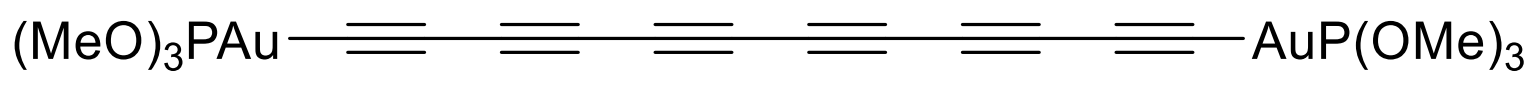

To a dry, degassed solution of 1,12-bis(triisopropylsilyl)-1,3,5,7,9,11-dodecahexayne (32 mg, 0.07 mmol) and AuCl{P(OMe)$_3$} (55 mg, 0.15 mmol) in a mixture of MeOH (15 mL) and tetrahydrofuran (5 mL) was added KF (10 mg, 0.18 mmol) and KO^{t}Bu (20 mg, 0.18 mmol) and the resulting mixture stirred for 2 hr at room temperature. The solvent was removed under vacuum and the residue sonicated in MeOH (~10 mL). The resulting mixture was filtered, and the collected material washed with MeOH (10 mL) and hexanes (10 mL), to afford the product as a yellow solid (30 mg, 54 %).

IR (ATR, $\tilde{\nu}$): *ν*(C≡C) 1997, 2093 cm^{-1}. ^1H NMR ($CDCl_3$, 400 MHz): *δ* 3.75 (d, *J* = 13.4 Hz, 18H, OMe) ppm. $^{13}C\{^1H\}$ NMR ($CDCl_3$, 101 MHz): *δ* 53.0 (OMe), 57.1 (C_{sp}), 60.7 (C_{sp}), 62.6 (C_{sp}), 63.3 (C_{sp}) ppm. All other acetylenic carbons are not observed. $^{13}C\{^1H,^{31}P\}$ ($CDCl_3$, 151 MHz): *δ* 53.0 (OMe), 57.1 (C_{sp}), 60.7 (C_{sp}), 62.6 (C_{sp}), 63.3 (C_{sp}), 87.5 (Au–C≡*C*), 132.0 (Au–*C*≡C) ppm. $^{31}P\{^1H\}$ NMR ($CDCl_3$, 162 MHz): *δ* 145.9 ppm (unreferenced). HR-MS (ESI(+)) *m*/*z*: calcd for $C_{18}H_{18}Au_2O_6P_2$+H 786.9982; found 786.9974 [M+H]$^+$.

Scheme for the synthesis of **S1**

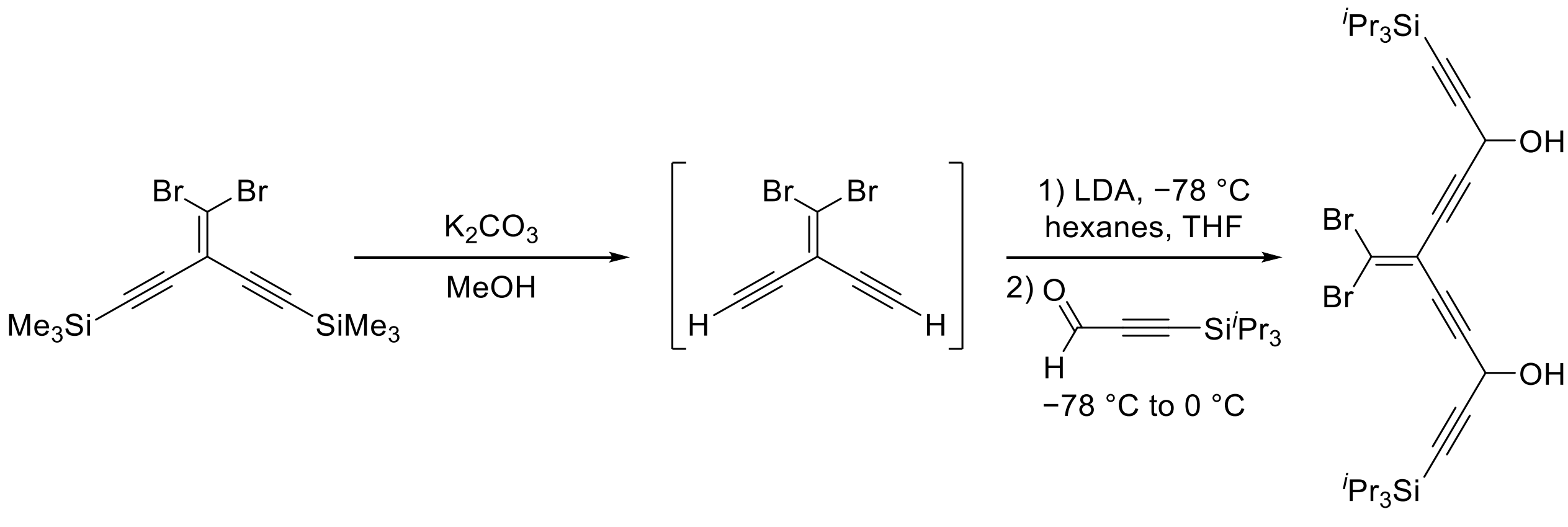

S1

6-(Dibromomethylidene)-1,11-bis(triisopropylsilyl)-3,9-dihydroxyundeca-1,4,7,10-tetrayne S1

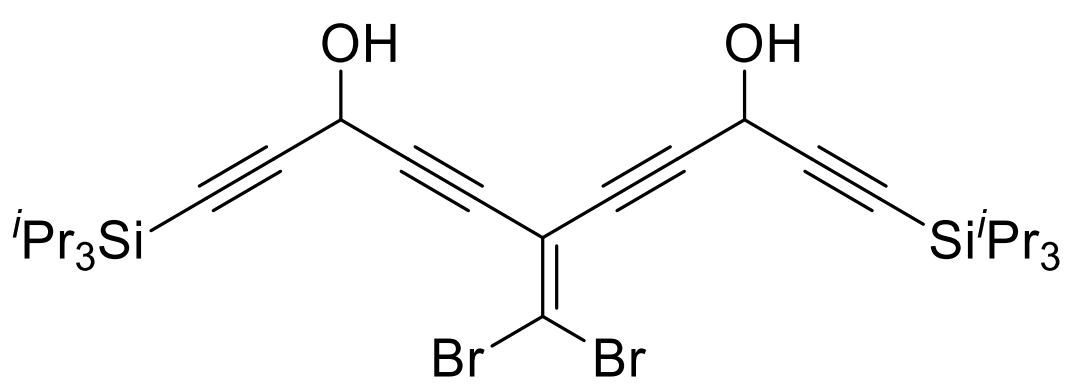

To a solution of 3-(dibromomethylidene)-1,5-bis(trimethylsilyl)penta-1,4-diyne (1.0 g, 2.6 mmol) in MeOH (30 mL) was added K_2CO_3 (0.91 g, 6.6 mmol) and the solution stirred at room temperature for 1 hr. The white

mixture was poured onto a saturated NH_4Cl solution (100 mL) and extracted with hexanes (3 × 10 mL). The combined organic layers were flushed through a small silica column (~5 cm long × 3 cm wide) eluting with additional volumes of hexanes. The 3-(dibromomethylidene)penta-1,4-diyne containing fractions (~70 mL hexanes) were transferred directly to a flask containing dry tetrahydrofuran (20 mL). In a separate flask, *n*-BuLi solution (3.3 mL, 2.0 M in cyclohexane, 6.6 mmol) was added to a dry solution of HN^iPr_2 (0.96 mL, 0.69 g, 6.9 mmol) in tetrahydrofuran (10 mL) at 0 °C and stirred for 30 min. The lithium diisopropylamide solution was then added dropwise, over 5 min, to the vigorously stirring solution of 3-(dibromomethylidene)penta-1,4-diyne at −78 °C. The resulting cloudy mixture was stirred for 1 hr, then 3-(triisopropylsilyl)propiolaldehyde (1.9 g, 9.0 mmol) was added. The mixture was stirred at −78 °C for 30 min, then warmed to 0 °C and stirred for 1 hr. Without warming, the solution was poured onto a saturated NH_4Cl solution (100 mL), extracted with diethyl ether (30 mL), dried over $MgSO_4$ and filtered. The solvent was evaporated and the residue purified by column chromatography (silica, 3:2 CH_2Cl_2:hexanes). Evaporation of the eluent afforded the product as a yellow oil (0.64 g, 37 %).

IR (ATR, $\tilde{v}$): *v*(C=C) 1624; *v*(C≡C) 2149, 2177; *v*(O–H) 3362 cm^{-1}. ^1H NMR ($CDCl_3$, 400 MHz): δ 1.08 (s, 42H, Si^iPr_3), 2.36 (d, J = 8.0 Hz, 2H, OH), 5.24 (d, J = 8.0 Hz, 2H, CH) ppm. $^{13}C\{^1H\}$ NMR ($CDCl_3$, 101 MHz): δ 11.2, 18.7, 53.2, 80.2, 87.4, 92.9, 102.7, 111.3, 112.6 ppm. HR-MS (APCI(+)) m/z: calcd for $C_{30}H_{46}{}^{79}Br^{81}BrO_2Si_2$+H 655.1455; found 655.1455 $[M+H]^+$.

Scheme for the syntheses of **S2**

Route 1

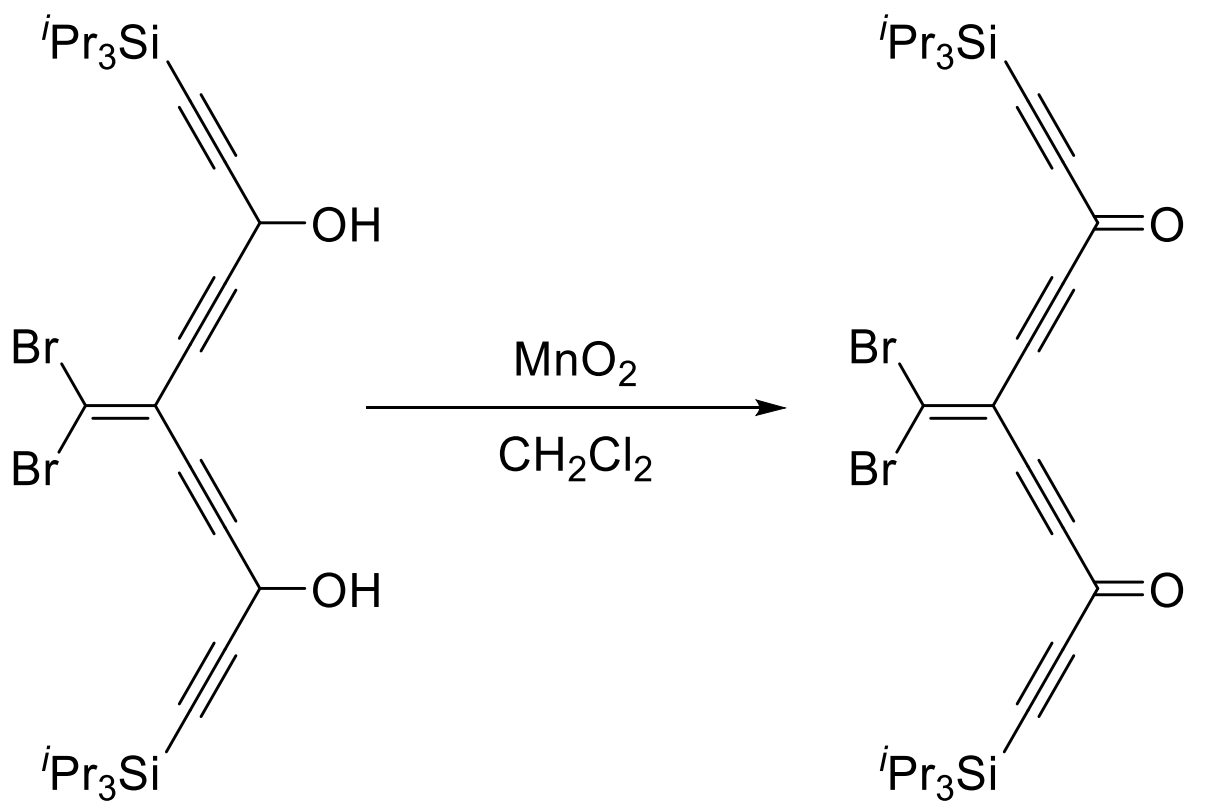

S1 **S2**

Route 2

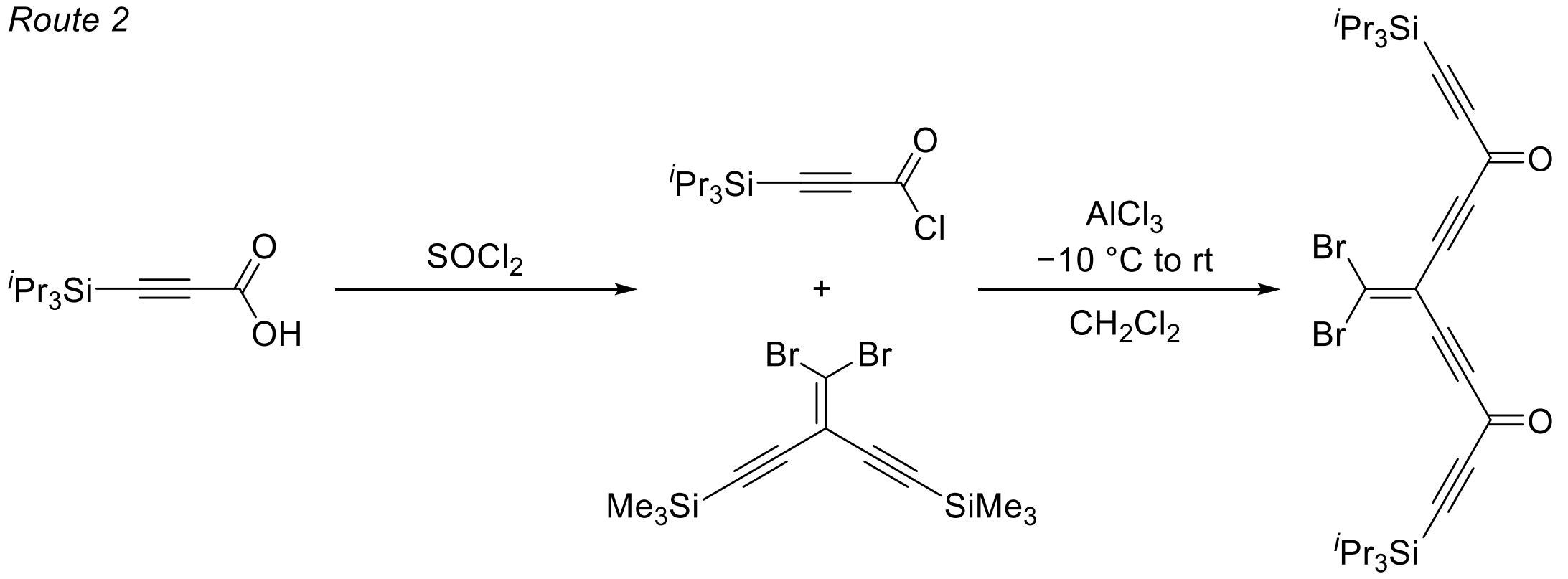

S2

6-(Dibromomethylidene)-1,11-bis(triisopropylsilyl)undeca-1,4,7,10-tetrayne-3,9-dione S2

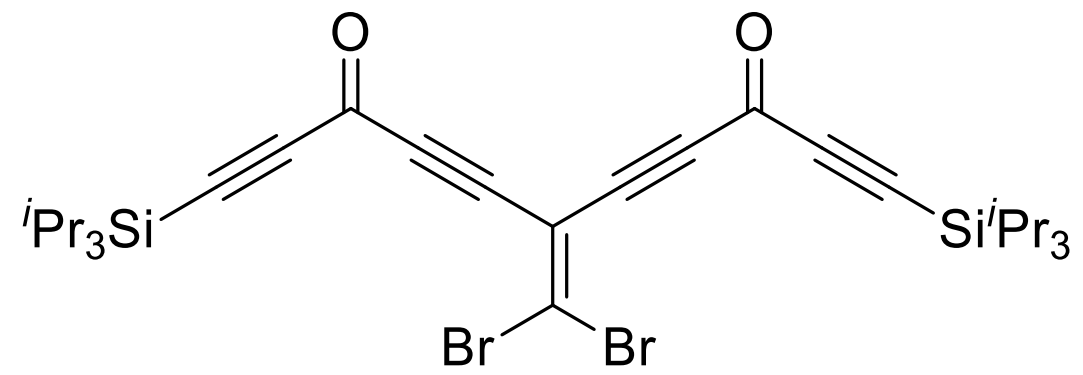

Synthetic Route 1: A mixture of **S1** (0.54 g, 0.82 mmol) and MnO_2‡ (0.54 g, 6.2 mmol) in dry CH_2Cl_2 (20 mL) was stirred at room temperature for 16 hr. The mixture was concentrated to ~5 mL, hexanes (5 mL) was added and the mixture filtered through silica eluting with 1:1 CH_2Cl_2:hexanes. The eluent was evaporated to afford the product as a pale-yellow solid (0.42 g, 79 %).

Synthetic Route 2: A solution of 3-triisopropylsilylpropynoic acid (1.0 g, 4.4 mmol) in thionyl chloride (4.0 mL, 6.5 g, 55 mmol) was stirred at room temperature overnight. The volatiles were evaporated under vacuum and the residue dissolved in dry CH_2Cl_2 (15 mL). To this solution was added 3-(dibromomethylidene)-1,5-bis(trimethylsilyl)penta-1,4-diyne (0.67 g, 1.8 mmol) and the mixture cooled to −10 °C. Then, $AlCl_3$ (0.59 g, 4.4 mmol) was added portionwise over 5 min. After complete addition the mixture was stirred for a further 1 hr at −10 °C then warmed to room temperature over 1 hr. The mixture was then quenched with 10 % HCl solution (50 mL). The organic layer was washed with saturated $NaHCO_3$ solution (50 mL) and brine (50 mL), then dried over $MgSO_4$ and filtered. The solvent was evaporated and the residue purified by column chromatography (silica, 2:3 CH_2Cl_2:hexanes). Evaporation of the eluent afforded the product as a pale-yellow solid (0.88 mg, 77 %).

IR (ATR, $\tilde{\nu}$): ν(C=O) 1635; ν(C≡C) 2150, 2188 cm^{-1}. ^1H NMR ($CDCl_3$, 400 MHz): δ 1.11 (m, 42H, Si^iPr_3) ppm. $^{13}C\{^1H\}$ NMR ($CDCl_3$, 101 MHz): δ 11.2, 18.6, 83.4, 94.0, 100.6, 104.5, 110.9, 122.8, 159.1 ppm. HR-MS (APCI(+)) *m/z*: calcd for $C_{30}H_{42}{}^{79}Br^{81}BrO_2Si_2$+H 651.1142; found 651.1139 $[M+H]^+$.

Scheme for the synthesis of **1,14-bis(triisopropylsilyl)-1,3,5,7,9,11,13-tetradecaheptayne**

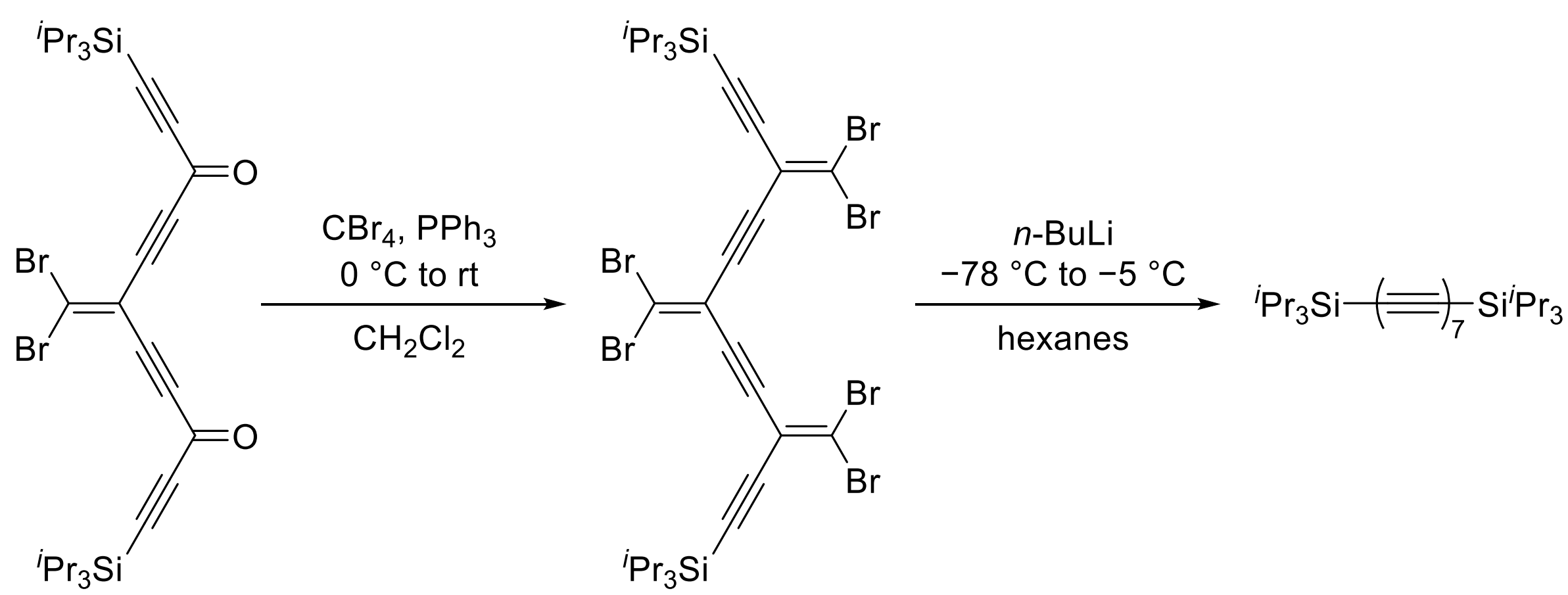

S2 **S3**

‡ 'Highly active' MnO_2 prepared from $MnCl_2$•$4H_2O$ and $KMnO_4$ by Dr Louisa Tham.[20,21]

3,6,9-Tris(dibromomethylidene)-1,11-bis(triisopropylsilyl)undeca-1,4,7,10-tetrayne S3

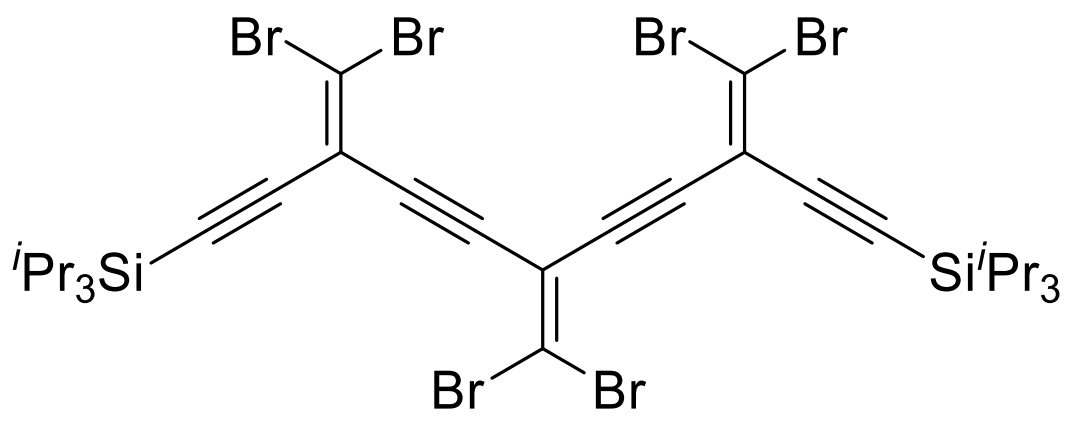

To a solution of PPh_3 (0.91 g, 3.5 mmol) in dry CH_2Cl_2 (25 mL) was added CBr_4 (0.57 g, 1.7 mmol) and the resulting solution stirred at room temperature for 15 min. The ylid solution was then added dropwise over 5 min to a solution of **S2** (0.42 g, 0.65 mmol) in dry CH_2Cl_2 (100 ml) at 0 °C. The dark-red solution was stirred for 30 min then warmed to room temperature over 2 hr. The solution was then poured onto hexanes (100 mL) and filtered through silica, eluting with further volumes of hexanes. The solvent was evaporated, and the residue purified by column chromatography (silica, hexanes). Evaporation of the eluent afforded the product as a pale-yellow oil (0.48 g. 77 %).

IR (ATR, $\tilde{\nu}$): No distinct functional group bands observed. ^{1}H NMR ($CDCl_3$, 400 MHz): δ 1.10 (m, 42H, Si^{i}Pr$_3$) ppm. ^{13}C{^{1}H} NMR ($CDCl_3$, 101 MHz): δ 11.3, 18.7, 90.6, 92.3, 100.8, 101.2, 110.9, 111.4, 113.5, 114.1 ppm. HR-MS (APCI(+)) *m*/*z*: calcd for $C_{32}H_{42}{}^{79}Br_3{}^{81}Br_3Si_2$ 961.7858; found 961.7838 [M]$^+$.

1,14-Bis(triisopropylsilyl)-1,3,5,7,9,11,13-tetradecaheptayne

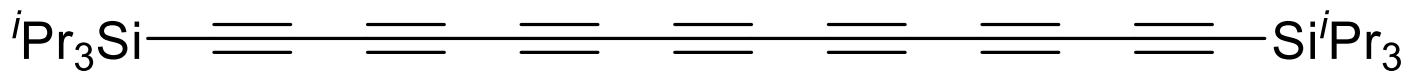

A solution of *n*-BuLi (0.34 mL, 2.0 M in cyclohexane, 0.69 mmol) was added dropwise over 5 min to a solution of **S3** (200 mg, 0.21 mmol) in hexanes (15 mL) at −78 °C. The resulting mixture was allowed to warm to −5 °C over 1.5 hr, then poured without warming onto saturated NH_4Cl solution (50 mL). The organic layer was separated, dried over $MgSO_4$ and filtered. The residue was purified by column chromatography (silica, hexanes). Evaporation of the eluent afforded the product as a pale yellow solid (31 mg, 31 %).

IR (ATR, $\tilde{\nu}$): ν(C≡C) 2037, 2069, 2137, 2164 cm^{-1}. ^{1}H NMR ($CDCl_3$, 400 MHz): δ 1.08 (m, 42H, Si^{i}Pr$_3$) ppm. ^{13}C{^{1}H} NMR ($CDCl_3$, 101 MHz): δ 11.4, 18.6, 61.3, 62.4, 62.8, 63.1, 63.1, 87.5, 89.5 ppm. HR-MS (APCI(+)) *m*/*z*: calcd for $C_{32}H_{42}Si_2$ 482.2820; found 482.2817 [M]$^+$. The spectroscopic data is in accordance with literature values.[22]

1,14-Bis[(trimethylphosphite)gold(I)]-1,3,5,7,9,11,13-tetradecaheptayne 1(14)

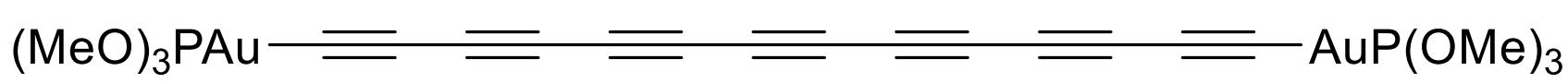

To a dry, degassed solution of 1,14-bis(triisopropylsilyl)-1,3,5,7,9,11,13-tetradecaheptayne (100 mg, 0.21 mmol) and AuCl{P(OMe)$_3$} (150 mg, 0.42 mmol) in a mixture of MeOH (10 mL) and tetrahydrofuran (10 mL) was added KF (26 mg, 0.46 mmol) and KO^{t}Bu (51 mg, 0.46 mmol) and the resulting mixture stirred for 3 hr at room temperature. The solvent was removed under vacuum and the residue sonicated in MeOH

(~10 mL). The resulting mixture was filtered, and the collected material washed with MeOH (10 mL) and hexanes (10 mL), to afford the product as a brown solid (82 mg, 49 %).

IR (ATR, $\tilde{\nu}$): ν(C≡C) 1988, 2102, 2150 cm^{-1}. ^{1}H NMR ($CDCl_3$, 500 MHz): δ 3.75 (d, J = 13.4 Hz, 18H, OMe) ppm. ^{13}C{^{1}H} NMR ($CDCl_3$, 126 MHz): δ 53.0 (OMe), 56.9 (C_{sp}), 60.3 (C_{sp}), 61.9 (C_{sp}), 63.6 (C_{sp}), 63.9 (d, J = 3.0 Hz, C_{sp}), 87.4 (d, J = 37 Hz, Au–C≡*C*), 132.7 (d, J = 209 Hz, Au–*C*≡C) ppm. ^{13}C{^{1}H,^{31}P} ($CDCl_3$, 151 MHz): δ 53.0 (OMe), 56.9 (C_{sp}), 60.3 (C_{sp}), 61.9 (C_{sp}), 63.6 (C_{sp}), 63.9 (C_{sp}), 87.4 (Au–C≡*C*), 132.7 (Au–*C*≡C) ppm. ^{31}P{^{1}H} NMR ($CDCl_3$, 202 MHz): δ 145.7 ppm (unreferenced). HR-MS (ESI(+)) *m*/*z*: calcd for $C_{20}H_{18}Au_2O_6P_2$+H 810.9982; found 810.9984 [M+H]$^{+}$.

1,16-Bis[(trimethylphosphite)gold(I)]-1,3,5,7,9,11,13,15-hexadecaoctayne 1(16)

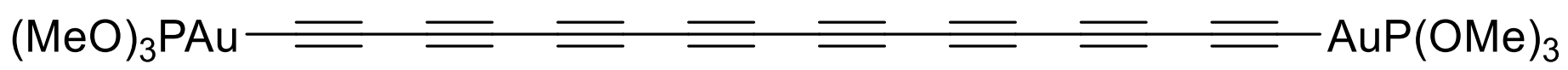

To a dry, degassed solution of 1,16-bis(triisopropylsilyl)-1,3,5,7,9,11,13,15-hexadecaoctayne (79 mg, 0.16 mmol) and AuCl{P(OMe)$_3$} (110 mg, 0.31 mmol) in a mixture of MeOH (10 mL) and tetrahydrofuran (10 mL) was added KF (22 mg, 0.38 mmol) and KO^{t}Bu (41 mg, 0.38 mmol) and the resulting mixture stirred for 3 hr at room temperature. The solvent was removed under vacuum and the residue sonicated in MeOH (~10 mL). The resulting mixture was filtered, and the collected material washed with MeOH (10 mL) and hexanes (10 mL), to afford the product as a brown solid (72 mg, 55 %).

IR (ATR, $\tilde{\nu}$): ν(C≡C) 1986, 2063, 2091 cm^{-1}. ^{1}H NMR ($CDCl_3$, 500 MHz): δ 3.75 (d, J = 13.4 Hz, 18H, OMe) ppm. ^{13}C{^{1}H} NMR ($CDCl_3$, 101 MHz): δ 53.0 (OMe), 56.8 (C_{sp}), 60.1 (C_{sp}), 61.6 (C_{sp}), 62.8 (C_{sp}), 64.2 (C_{sp}), 64.3 (C_{sp}) ppm. All other acetylenic carbons are not observed. ^{13}C{^{1}H,^{31}P} ($CDCl_3$, 151 MHz): δ 53.0 (OMe), 56.8 (C_{sp}), 60.1 (C_{sp}), 61.6 (C_{sp}), 62.8 (C_{sp}), 64.2 (C_{sp}), 64.3 (C_{sp}), 87.3 (Au–C≡*C*), 133.2 (Au–*C*≡C) ppm. ^{31}P{^{1}H} NMR ($CDCl_3$, 162 MHz): δ 145.6 ppm (unreferenced). HR-MS (ESI(+)) *m*/*z*: calcd for $C_{22}H_{18}Au_2O_6P_2$+H 834.9982; found 834.9988 [M+H]$^{+}$.

Scheme for the synthesis of **1,18-bis(triisopropylsilyl)-1,3,5,7,9,11,13,15,17-octadecanonayne**

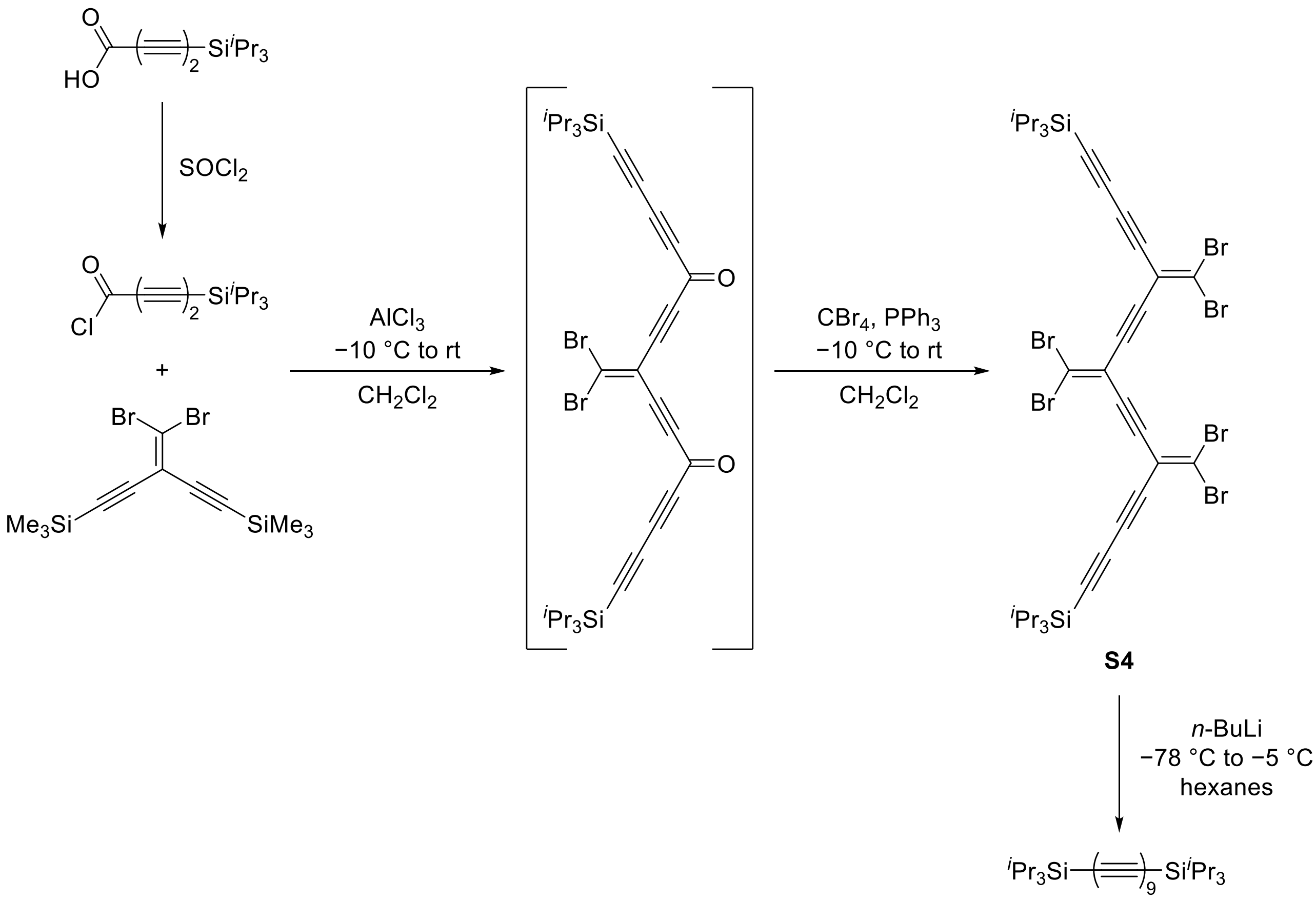

5,8,11-Tris(dibromomethylidene)-1,15-bis(triisopropylsilyl)pentadeca-1,3,6,9,12,14-hexayne S4

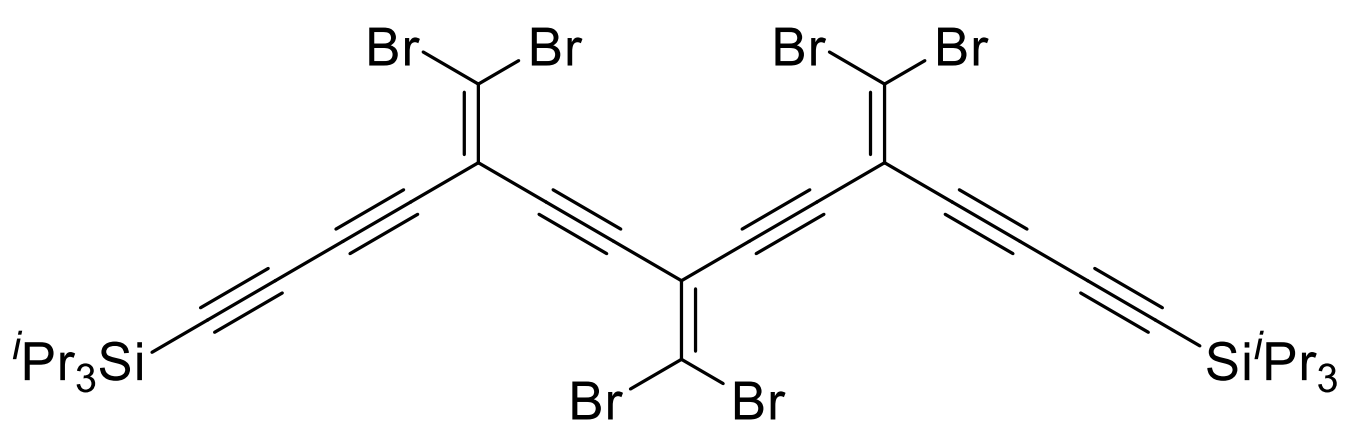

A solution of 5-triisopropylsilylbutadiynoic acid (820 mg, 3.3 mmol) in thionyl chloride (2.9 mL, 4.8 g, 40 mmol) was stirred at room temperature overnight. The volatiles were removed under vacuum and the residue dissolved in dry CH_2Cl_2 (30 mL). To this solution was added 3-(dibromomethylidene)-1,5-bis(trimethylsilyl)penta-1,4-diyne (500 mg, 1.3 mmol) and the mixture cooled to −10 °C. Then, $AlCl_3$ (440 mg, 3.3 mmol) was added portion-wise over 5 min. After complete addition the mixture was stirred for a further 1 hr at −10 °C then allowed to warm to room temperature over 1 hr. The mixture was then quenched with a 10 % HCl solution (50 mL). The organic layer was separated and directly flushed through a small silica plug eluting with additional volumes of CH_2Cl_2. The diketone solution (~60 mL) was transferred directly to a reaction flask. *NOTE: Attempts at purification and isolation of the diketone intermediate resulted in marked decomposition leading to decreased yields.* In a separate flask, CBr_4 (1.2 g, 3.5 mmol) was added to a solution of PPh_3 (1.8 g, 7.0 mmol) in CH_2Cl_2 (10 mL) at 0 °C and the resulting solution stirred for 15 min. The ylid solution was added dropwise over 5 min to the diketone solution at −10 °C. The dark-red solution was stirred for 1 hr at −10 °C

then warmed to room temperature over 1 hr. The solution was then poured onto hexanes (100 mL) and filtered through silica, eluting with further volumes of hexanes. The solvent was removed under reduced pressure, and the residue purified by column chromatography (silica, hexanes). Evaporation of the eluent afforded the product as a yellow oil (0.70 g, 53 %).

IR (ATR, $\tilde{\nu}$): *ν*(C≡C) 2095, 2197 cm^{-1}. ^{1}H NMR ($CDCl_3$, 400 MHz): *δ* 1.10 (m, 42H, Si^{i}Pr$_3$) ppm. ^{13}C{^{1}H} NMR ($CDCl_3$, 101 MHz): *δ* 11.4, 18.7, 70.4, 81.6, 88.8, 91.3, 91.6, 93.6, 112.0, 112.9, 113.2, 113.6 ppm. HR-MS (APCI(+)) *m*/*z*: calcd for $C_{36}H_{42}{}^{79}Br_3{}^{81}Br_3Si_2$ 1009.7858; found 1009.7850 [M]$^+$.

1,18-Bis(triisopropylsilyl)-1,3,5,7,9,11,13,15,17-octadecanonayne

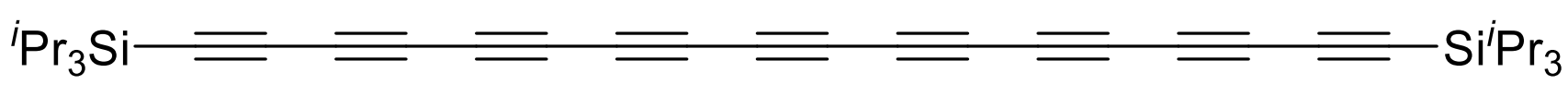

A solution of *n*-BuLi (0.33 mL, 2.0 M in cyclohexane, 0.67 mmol) was added dropwise over 5 min to a solution of **S4** (380 mg, 0.38 mmol) in hexanes (30 mL) at −78 °C. The resulting mixture was allowed to warm to −5 °C over 1.5 hr, then poured without warming onto saturated NH_4Cl solution (50 mL). The organic layer was separated, dried over $MgSO_4$ and filtered. The residue was purified by column chromatography (silica, hexanes). Evaporation of the eluent afforded the product as a pale yellow solid (31 mg, 15 %).

IR (ATR, $\tilde{\nu}$): *ν*(C≡C) 1992, 2055, 2094, 2131, 2178 cm^{-1}. ^{1}H NMR ($CDCl_3$, 400 MHz): *δ* 1.08 (m, 42H, Si^{i}Pr$_3$) ppm. ^{13}C{^{1}H} NMR ($CDCl_3$, 101 MHz): *δ* 11.4, 18.6, 61.3, 62.3, 62.6, 62.9, 63.3, 63.4, 63.6, 88.0, 89.4 ppm. HR-MS (APCI(+)) *m*/*z*: calcd for $C_{36}H_{42}Si_2$ 530.2820; found 530.2816 [M]$^+$. This compound has been previously reported without characterisation.[4]

1,18-Bis[(trimethylphosphite)gold(I)]-1,3,5,7,9,11,13,15,17-octadecanonayne 1(18)

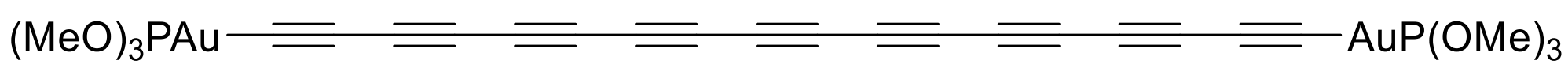

To a dry, degassed solution of 1,18-bis(triisopropylsilyl)-1,3,5,7,9,11,13,15,17-octadecanonayne (33 mg, 0.06 mmol) and AuCl{P(OMe)$_3$} (44 mg, 0.12 mmol) in a mixture of MeOH (10 mL) and tetrahydrofuran (10 mL) was added KF (9 mg, 0.16 mmol) and KO^{t}Bu (17 mg, 0.16 mmol) and the resulting mixture stirred for 3 hr at room temperature. The solvent was removed under vacuum and the residue sonicated in MeOH (~10 mL). The resulting mixture was filtered, and the collected material washed with MeOH (10 mL) and hexanes (10 mL), to afford the product as a brown solid (28 mg, 53 %). *NOTE: The product could not be obtained in sufficient purity for further analysis. Spectroscopic data from a crude sample is provided for comparison only.*

IR (ATR, $\tilde{\nu}$): *ν*(C≡C) 1934, 2000, 2059, 2119 cm^{-1}. ^{1}H NMR ($CDCl_3$, 400 MHz): *δ* 3.75 (d, *J* = 13.4 Hz, 18H, OMe) ppm. ^{31}P{^{1}H} NMR ($CDCl_3$, 162 MHz): *δ* 145.5 ppm (unreferenced). The solubility of the product was too low to obtain ^{13}C{^{1}H} NMR data. HR-MS (ESI(+)) *m*/*z*: calcd for $C_{24}H_{18}Au_2O_6P_2$+H 858.9982; found 858.9975 [M+H]$^+$.

1,20-Bis[(trimethylphosphite)gold(I)]-1,3,5,7,9,11,13,15,17,19-eicosadecayne 1(20)

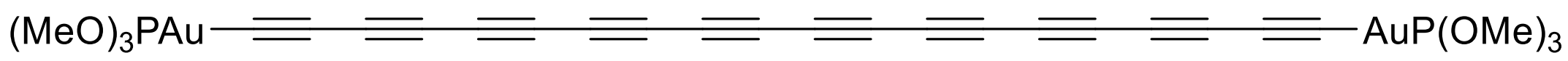

To a dry, degassed solution of 1,20-bis(triisopropylsilyl)-1,3,5,7,9,11,13,15,17,19-eicosadecayne (10 mg, 0.02 mmol) and AuCl{P(OMe)$_3$} (15 mg, 0.04 mmol) in a mixture of MeOH (10 mL) and tetrahydrofuran (10 mL) was added KF (3 mg, 0.04 mmol) and KO^{t}Bu (5 mg, 0.04 mmol) and the resulting mixture stirred for 2 hr at room temperature. The solvent was removed under vacuum and residue sonicated in MeOH (~10 mL). The resulting mixture was filtered, and the collected material washed with MeOH (10 mL) and hexanes (10 mL), to afford the product as a red solid. **When attempting to transfer the product from a sintered funnel using a spatula the solid detonated.** *NOTE: A small sample of crude material was recovered from the reaction flask and characterised in dilute solution to confirm formation of the product.*

^{1}H NMR (CDCl$_3$, 400 MHz): δ 3.76 (d, J = 13.4 Hz, 18H, OMe) ppm. ^{31}P{^{1}H} NMR (CDCl$_3$, 162 MHz): δ 145.4 ppm (unreferenced). The solubility of the product was too low to obtain ^{13}C{^{1}H} NMR data. HR-MS (ESI(+)) *m*/*z*: calcd for $C_{26}H_{18}Au_2O_6P_2$+H 882.9982; found 882.9980 [M+H]$^+$.

[1,4-(Diethynyl)phenyl]-bis[(trimethylphosphite)gold(I)] A1

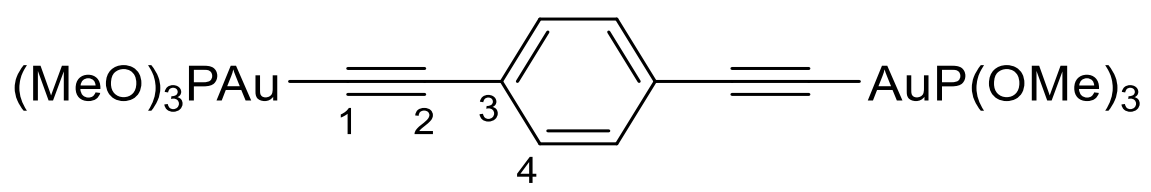

To a dry, degassed solution of 1,4-bis((trimethylsilyl)ethynyl)-phenylene (38 mg, 0.14 mmol) and AuCl{P(OMe)$_3$} (100 mg, 0.28 mmol) in MeOH (10 mL) was added KF (16 mg, 0.28 mmol) and KO^{t}Bu (30 mg, 0.28 mmol) and the resulting mixture stirred for 3 hr at room temperature. The solvent was removed under vacuum and the residue was sonicated in MeOH (~10 mL). The resulting mixture was filtered, and the collected material washed with MeOH (10 mL) and hexanes (10 mL), to afford the product as a white solid (61 mg, 29 %).

IR (ATR, $\tilde{\nu}$): ν(C≡C) 2113 cm^{-1}. ^{1}H NMR (CDCl$_3$, 400 MHz): δ 3.77 (d, J = 13.4 Hz, 18H, OMe), 7.35 (s, 4H, H^4) ppm. ^{13}C{^{1}H} NMR (CDCl$_3$, 101 MHz): δ 52.8 (OMe), 123.1 (C^3), 132.2 (C^4) ppm. All acetylenic carbons are not observed. ^{31}P{^{1}H} NMR (CDCl$_3$, 162 MHz): δ 150.3 ppm (unreferenced). HR-MS (ESI(+)) *m*/*z*: calcd for $C_{16}H_{22}Au_2O_6P_2$+H 767.0295; found 767.0304 [M+H]$^+$. The spectroscopic data is in accordance with literature values.[23]

[4-(Methylthio)phenyl]ethynyl-(trimethylphosphite)gold(I) A2

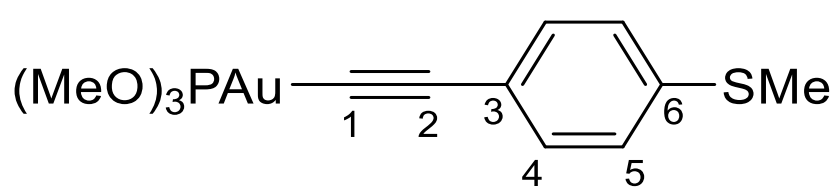

To a dry, degassed solution of 4-ethynylthioanisole (41 mg, 0.28 mmol) and AuCl{P(OMe)$_3$} (100 mg, 0.28 mmol) in MeOH (10 mL) was added KO^{t}Bu (31 mg, 0.28 mmol) and the resulting mixture stirred for 3 hr at room temperature. The solvent was evaporated under vacuum and the residue washed with pentane (2 × 5 mL) and ethanol (2 mL) to afford the product as a yellow solid on standing (33 mg, 25 %).

IR (ATR, $\tilde{\nu}$): ν(C≡C) 2114 cm^{-1}. ^{1}H NMR ($CDCl_3$, 400 MHz): δ 2.46 (s, 3H, SMe), 3.78 (d, J = 13.4 Hz, 9H, OMe), 7.13 (d, J = 8.51 Hz, 2H, H), 7.39 (d, J = 8.47 Hz, 2H, H) ppm. ^{13}C{^{1}H} NMR ($CDCl_3$, 101 MHz): δ 15.7 (SMe), 52.8 (OMe), 121.0 (C3), 126.0 (C5), 132.9 (C4), 137.7 (C6) ppm. All acetylenic carbons are not observed. ^{31}P{^{1}H} NMR ($CDCl_3$, 162 MHz): δ 150.1 ppm (unreferenced). HR-MS (ESI(+)) m/z: calcd for $C_{12}H_{16}AuO_3PS$ 468.0218; found 468.0217 [M]$^+$.

(4-Tolyl)ethynyl-(trimethylphosphite)gold(I) A3

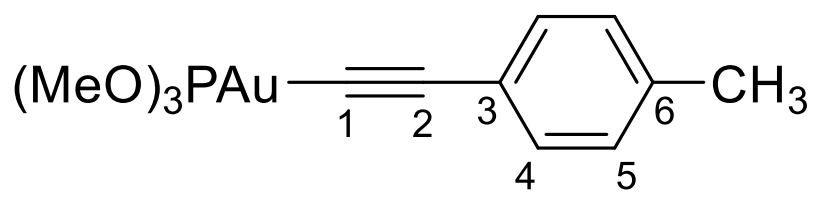

To a dry, degassed solution of 4-[(trimethylsilyl)ethynyl]toluene (57 mg, 0.30 mmol) and AuCl{P(OMe)$_3$} (95 mg, 0.27 mmol) in MeOH (10 mL) was added KF (16 mg, 0.27 mmol) and the resulting solution stirred for 3 hr at room temperature. The solution was concentrated to ~2 mL under vacuum and the resulting cold mixture was filtered, and the collected material washed with cold MeOH (1 mL) and hexanes (5 mL), to afford the product as a colourless, crystalline solid (19 mg, 16 %).

IR (ATR, $\tilde{\nu}$): ν(C≡C) 2119 cm^{-1}. ^{1}H NMR ($CDCl_3$, 400 MHz): δ 2.32 (s, 3H, CH_3), 3.77 (d, J = 13.4 Hz, 9H, OMe), 7.06 (d, J = 8.0 Hz, 2H, H5), 7.38 (d, J = 8.0 Hz, 2H, H4) ppm. ^{13}C{^{1}H} NMR ($CDCl_3$, 101 MHz): δ 21.6 (CH_3), 52.8 (OMe), 121.4 (C3), 128.9 (C5), 132.4 (C4), 137.2 (C6) ppm. All acetylenic carbons are not observed. ^{31}P{^{1}H} NMR ($CDCl_3$, 162 MHz): δ 150.5 ppm (unreferenced). HR-MS (ESI(+)) m/z: calcd for $C_{12}H_{16}AuO_3P$+Na 459.0395; found 459.0392 [M+Na]$^+$.

1-(Triisopropylsilyl)hexa-1,4-diyn-3-one S5

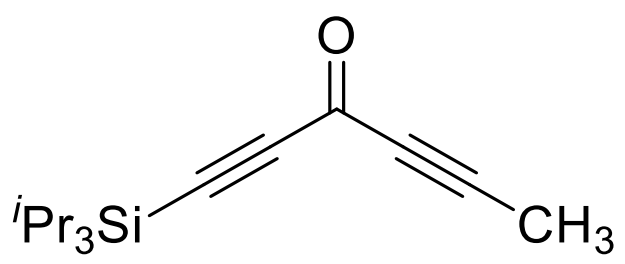

A solution of 3-triisopropylsilylpropynoic acid (0.50 g, 2.2 mmol) in thionyl chloride (2.0 mL, 3.3 g, 28 mmol) was stirred at room temperature overnight. The volatiles were evaporated under vacuum and the residue dissolved in dry CH_2Cl_2 (10 mL). To this solution was added 1-(trimethylsilyl)propyne (0.30 mL, 0.23 g, 2.0 mmol) and the mixture cooled to 0 °C. Then, $AlCl_3$ (0.29 g, 2.2 mmol) was added portionwise over 5 min. After complete addition the mixture was stirred for a further 30 min at 0 °C then warmed to room temperature over 1 hr. The mixture was then quenched with 10 % HCl solution (50 mL). The organic layer was separated, combined with hexanes (10 mL) and filtered through silica eluting with 1:1 CH_2Cl_2/hexanes. Evaporation of the eluent afforded the product as a yellow oil (0.50 mg, 90 %).

IR (ATR, $\tilde{\nu}$): ν(C=O) 1628; ν(C≡C) 2146, 2196, 2242 cm^{-1}. ^{1}H NMR ($CDCl_3$, 400 MHz): δ 1.11 (m, 21H, Si^{i}Pr$_3$), 2.07 (s, 3H, CH_3) ppm. ^{13}C{^{1}H} NMR ($CDCl_3$, 101 MHz): δ 4.5 (CH_3), 11.2 (Si^{i}Pr$_3$), 18.6 (Si^{i}Pr$_3$), 81.7 (C_{sp}), 91.4 (C_{sp}), 96.3 (C_{sp}), 105.2 (C_{sp}), 160.8 (C=O) ppm. HR-MS (APCI(+)) m/z: calcd for $C_{15}H_{24}OSi$+H 249.1669; found 249.1669 [M+H]$^+$.

3-(Dibromomethylidene)-1-(triisopropylsilyl)hexa-1,4-diyne S6

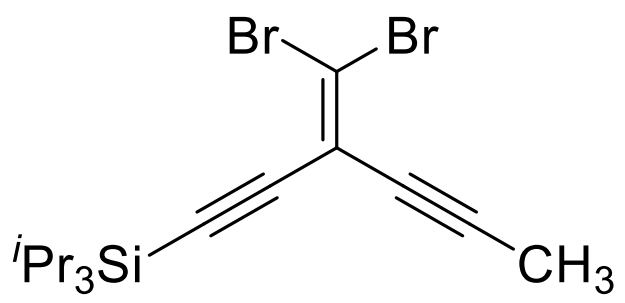

To a solution of PPh_3 (1.28 g, 4.9 mmol) in dry CH_2Cl_2 (10 mL) was added CBr_4 (0.78 g, 2.4 mmol) and the resulting solution stirred at room temperature for 15 min. A solution of **S5** (0.45 g, 1.8 mmol) in dry CH_2Cl_2 (10 mL) was added dropwise at 0 °C. The dark solution was stirred for 30 min then warmed to room temperature over 2 hr. The solution was then poured onto hexanes (100 mL) and filtered through silica, eluting with further volumes of hexanes. Evaporation of the eluent afforded the product as a yellow oil (0.34 g. 46 %).

IR (ATR, $\tilde{\nu}$): ν(C≡C) 2141, 2229. ^{1}H NMR ($CDCl_3$, 400 MHz): δ 1.10 (m, 21H, Si^{i}Pr$_3$), 1.99 (s, 3H, CH_3) ppm. ^{13}C{^{1}H} NMR ($CDCl_3$, 101 MHz): δ 5.0 (CH_3), 11.3 (Si^{i}Pr$_3$), 18.7 (Si^{i}Pr$_3$), 77.0, 93.7, 98.9, 102.7, 107.5, 114.9 ppm. HR-MS (APCI(+)) *m*/*z*: calcd for $C_{16}H_{24}{}^{79}Br^{81}BrSi$ 403.9888; found 403.9861 [M]$^+$.

1-(Triisopropylsilyl)hepta-1,3,5-triyne S7

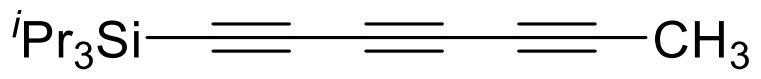

A solution of *n*-BuLi (0.50 mL, 2.0 M in cyclohexane, 0.98 mmol) was added dropwise over 5 min to a solution of **S6** (330 mg, 0.82 mmol) in hexanes (10 mL) at −78 °C. The resulting mixture was allowed to warm to 0 °C over 2 hr, then poured without warming onto saturated NH_4Cl solution (50 mL). The organic layer was separated, dried over $MgSO_4$ and filtered. The residue was purified by column chromatography (silica, hexanes). Evaporation of the eluent afforded the product as a yellow oil (172 mg, 85 %).

IR (ATR, $\tilde{\nu}$): ν(C≡C) 2079, 2166, 2216 cm^{-1}. ^{1}H NMR ($CDCl_3$, 400 MHz): δ 1.08 (s, 21H, Si^{i}Pr$_3$), 1.97 (s, 3H, CH_3) ppm. ^{13}C{^{1}H} NMR ($CDCl_3$, 101 MHz): δ 4.7 (CH_3), 11.4 (Si^{i}Pr$_3$), 18.7 (Si^{i}Pr$_3$), 60.0, 61.7, 64.9, 76.3, 83.0, 90.2 ppm. HR-MS (APCI(+)) *m*/*z*: calcd for $C_{16}H_{24}$Si+MeCN+H 286.1986; found 286.1985 [M+MeCN+H]$^+$.

Hepta-1,3,5-triynyl-(trimethylphosphite)gold(I) A4

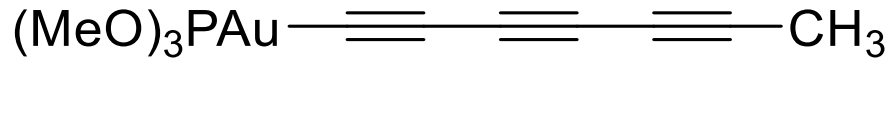

To a dry, degassed solution of 1-(triisopropylsilyl)hepta-1,3,5-triyne (80 mg, 0.33 mmol) and AuCl{P(OMe)$_3$} (117 mg, 0.33 mmol) in a mixture of MeOH (10 mL) and tetrahydrofuran (10 mL) was added KF (22 mg, 0.36 mmol) and KO^{t}Bu (40 mg, 0.36 mmol) and the resulting mixture stirred for 3 hr at room temperature. The solvent was removed under vacuum and the residue sonicated in MeOH (~10 mL). The resulting mixture was filtered, and the collected material washed with MeOH (10 mL) and hexanes (10 mL), to afford the product as a brown solid (17 mg, 13 %).

IR (ATR, $\tilde{\nu}$): ν(C≡C) 1991, 2049, 2147 cm^{-1}. ^{1}H NMR ($CDCl_3$, 400 MHz): δ 1.94 (s, 3H, CH_3), 3.75 (d, *J* = 13.4 Hz, 9H, OMe) ppm. ^{13}C{^{1}H} NMR ($CDCl_3$, 101 MHz): δ 4.7 (CH_3), 52.9 (OMe), 56.9 (C_{sp}), 60.8 (C_{sp}), 65.7

(C_{sp}), 73.5 (C_{sp}) ppm. All other acetylenic carbons are not observed. $^{31}P\{^1H\}$ NMR ($CDCl_3$, 162 MHz): δ 147.1 ppm (unreferenced). HR-MS (ESI(+)) *m*/*z*: calcd for $C_{10}H_{12}AuO_3P$+Na 431.0082; found 431.0081 $[M+Na]^+$.

1.6 Plots of NMR spectra and summaries of data.

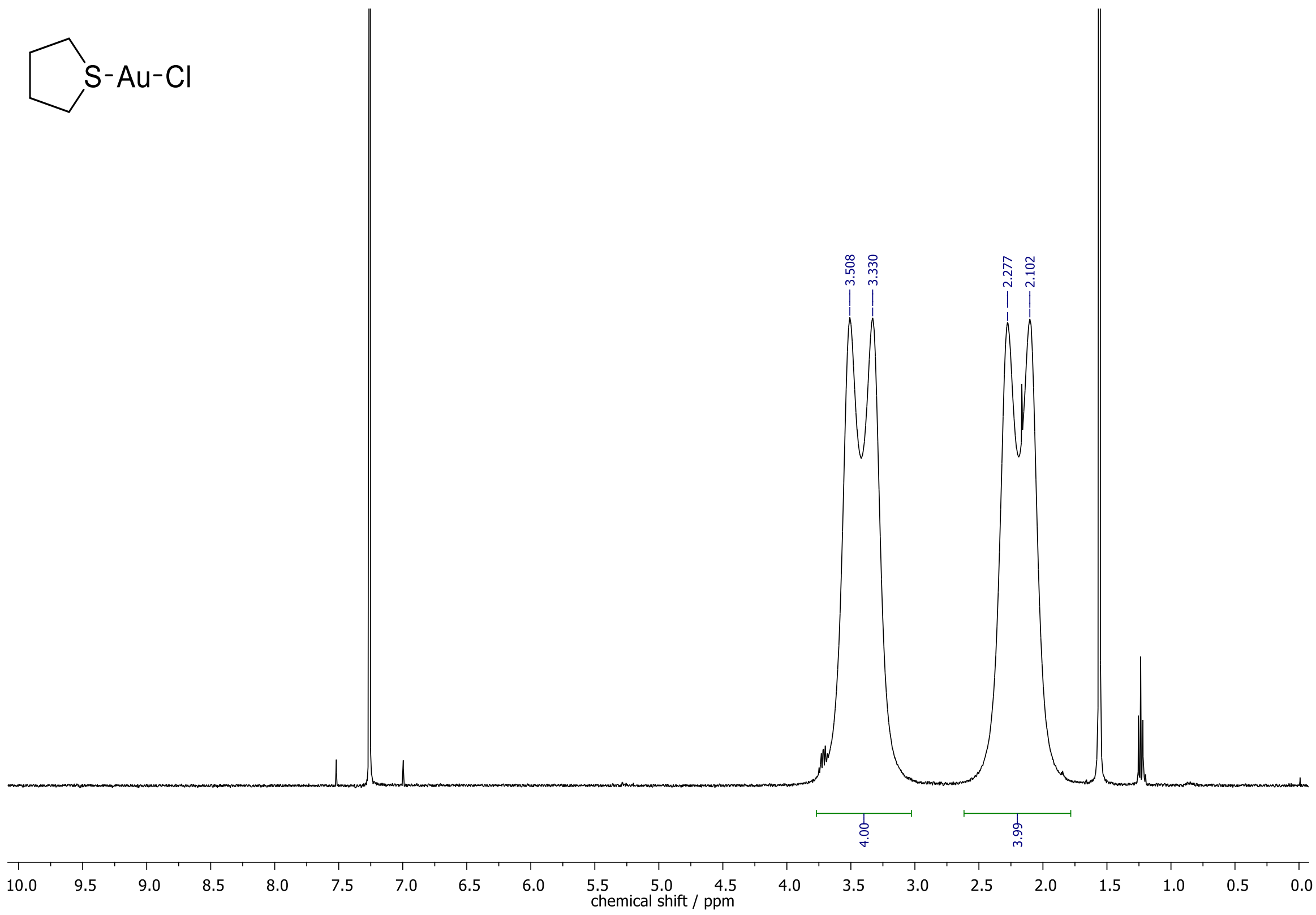

Supplementary Figure 1: ^{1}H NMR spectrum of **AuCl(S_4H_8)**.

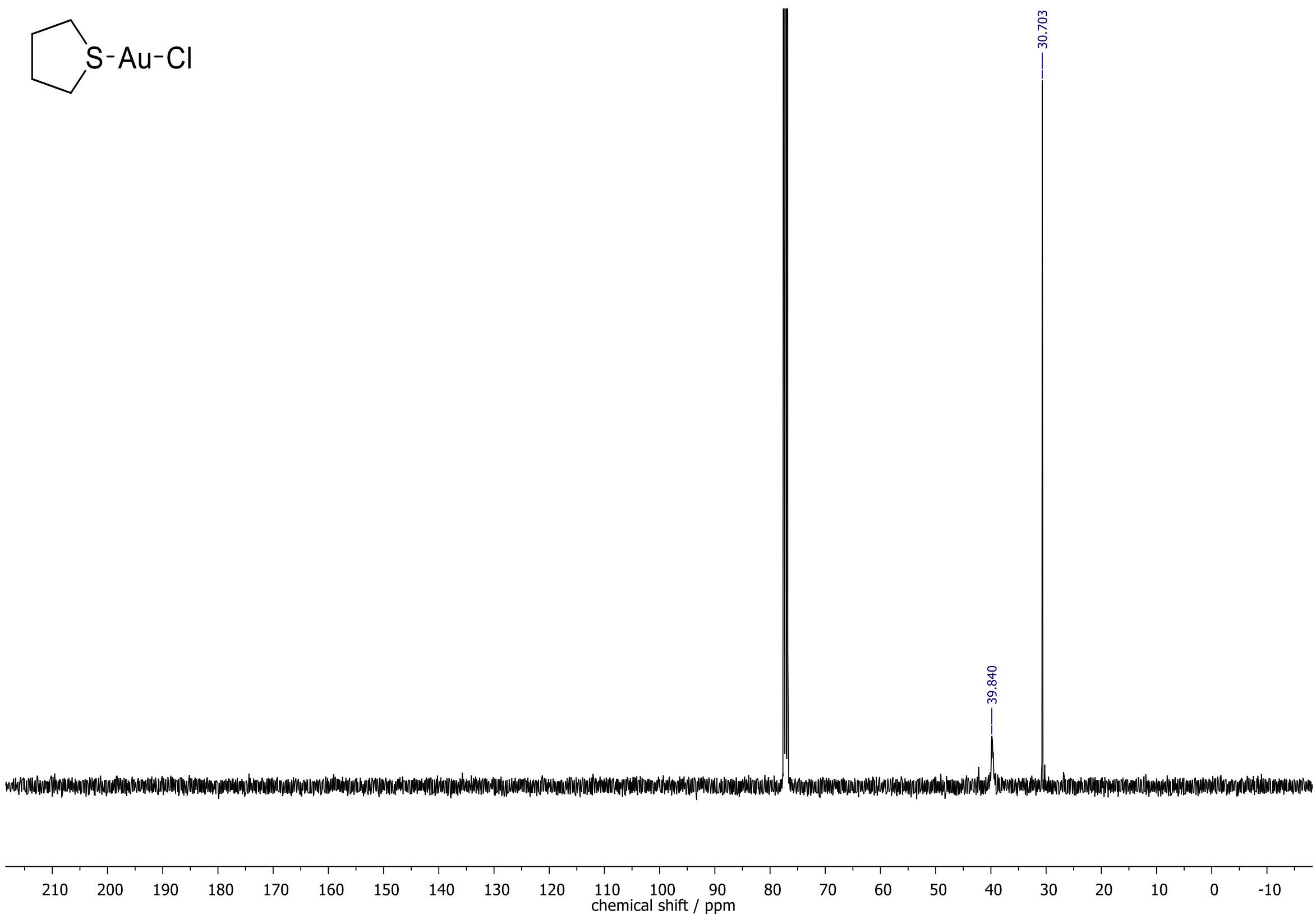

Supplementary Figure 2: $^{13}C\{^{1}H\}$ NMR spectrum of **AuCl(S_4H_8)**.

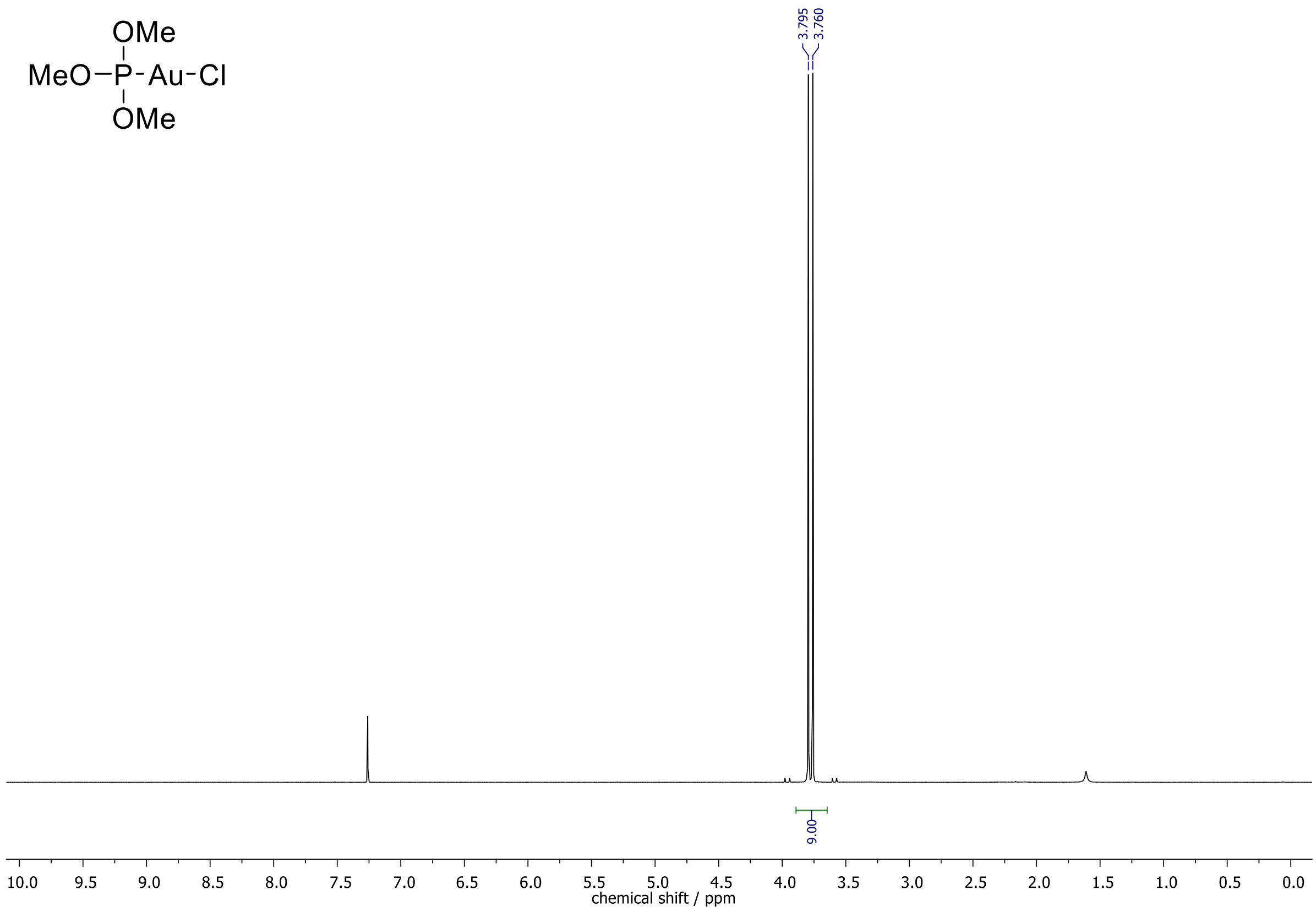

Supplementary Figure 3: ^{1}H NMR spectrum of **AuCl{P(OMe)$_3$}**.

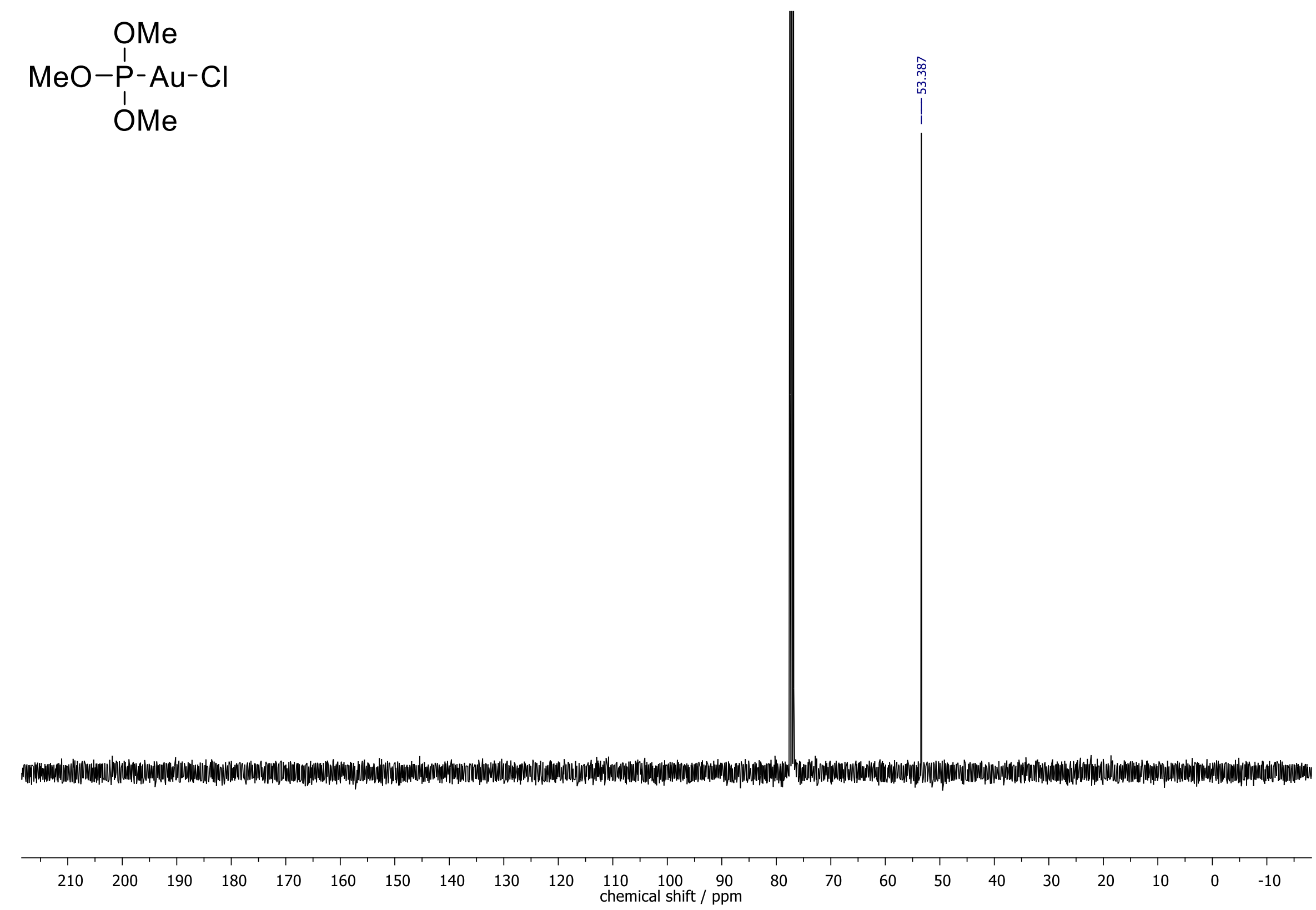

Supplementary Figure 4: The $^{13}C\{^{1}H\}$ NMR spectrum of **AuCl{P(OMe)$_3$}**.

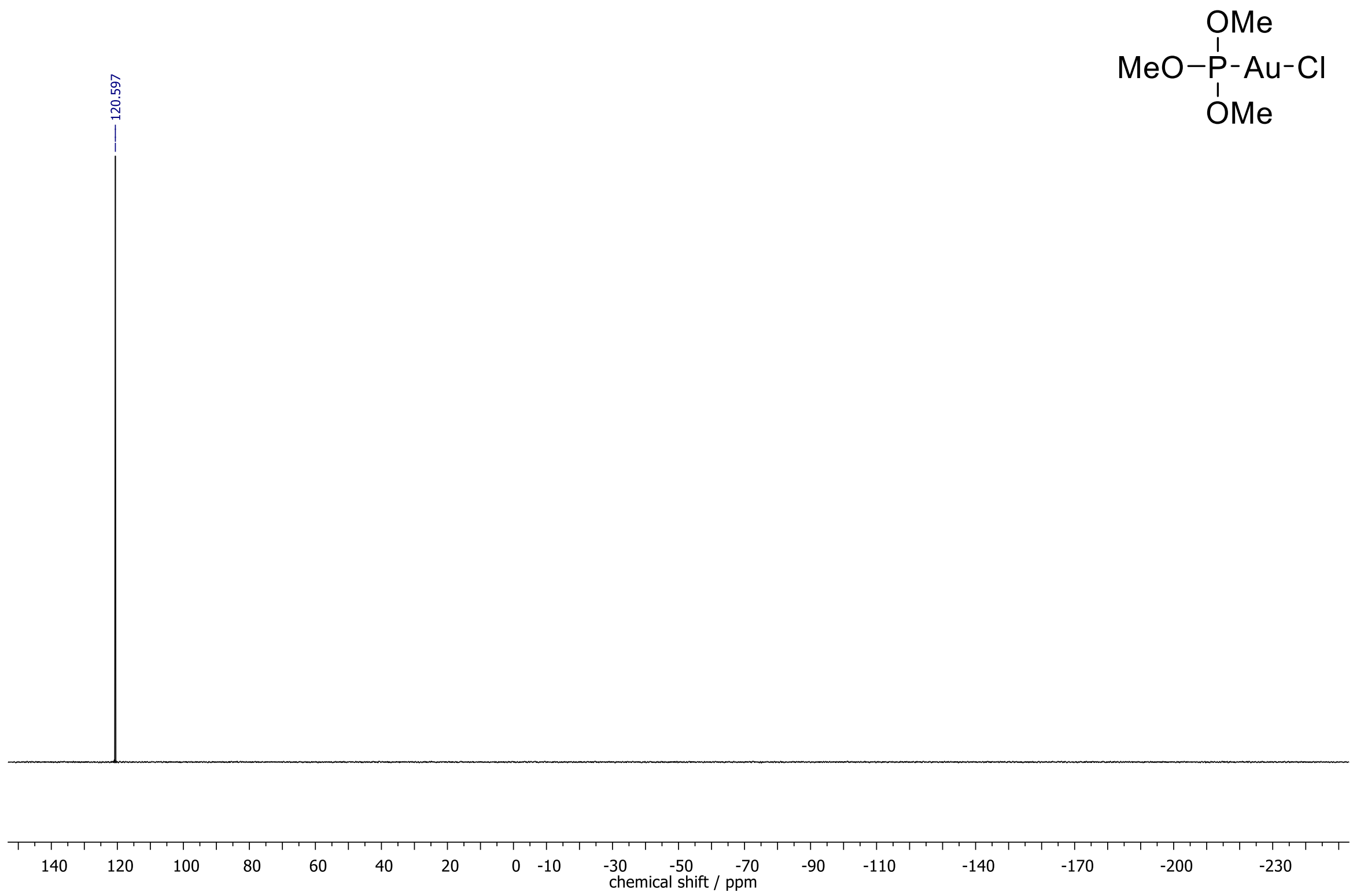

Supplementary Figure 5: $^{31}P\{^{1}H\}$ NMR spectrum of **AuCl{P(OMe)₃}**.

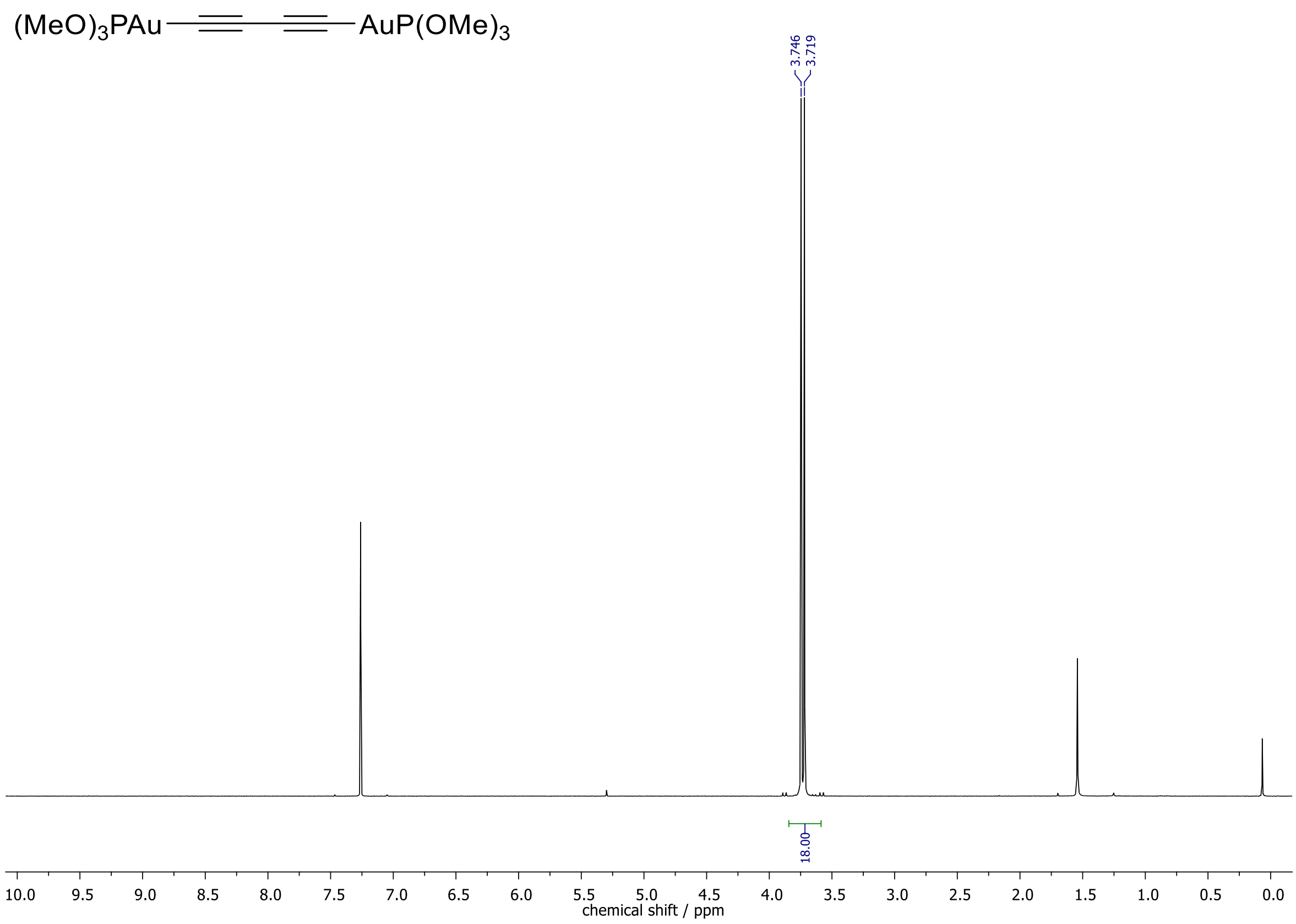

Supplementary Figure 6: ^{1}H NMR spectrum of **1(4)**.

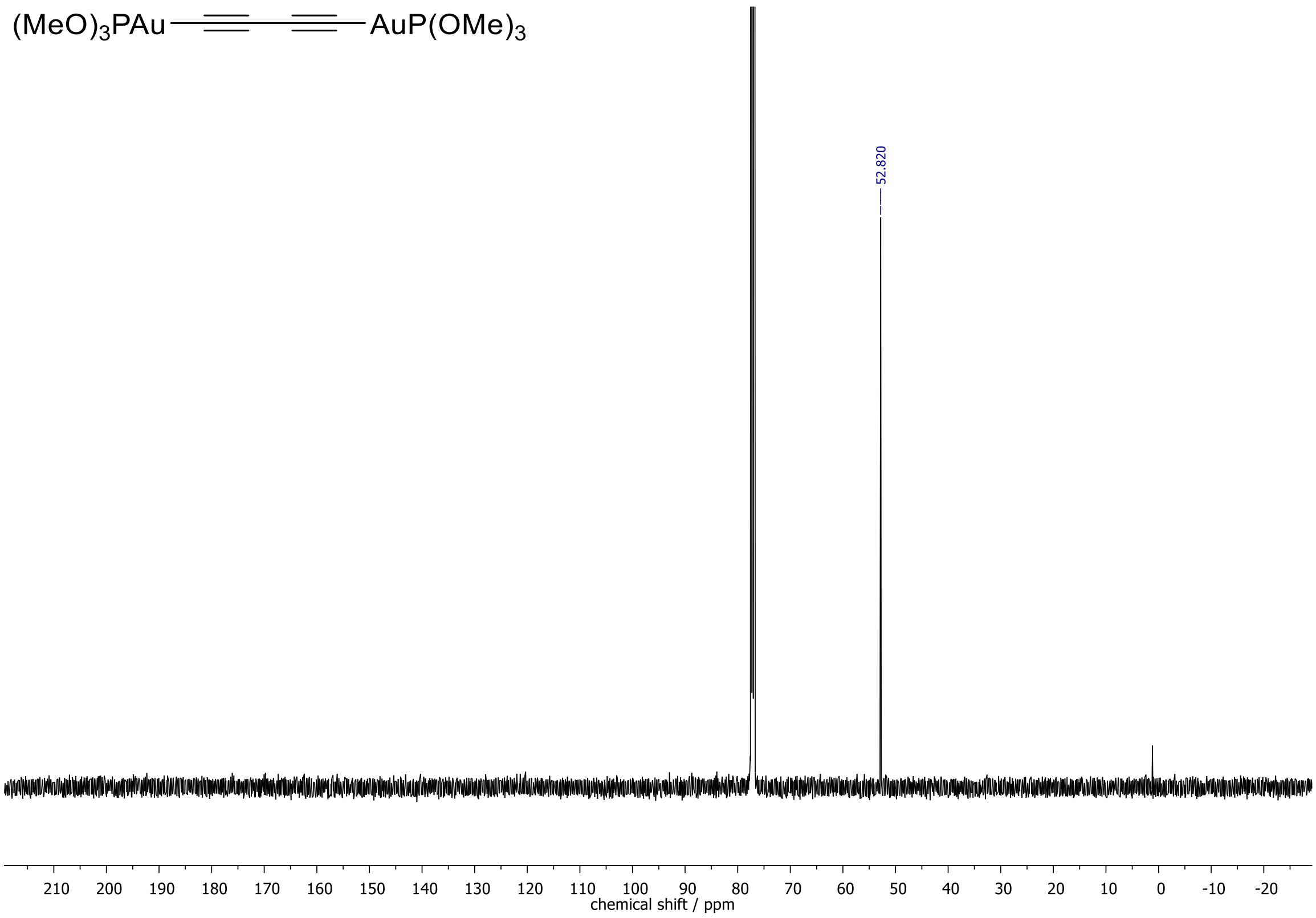

Supplementary Figure 7: $^{13}C\{^{1}H\}$ NMR spectrum of **1(4).**

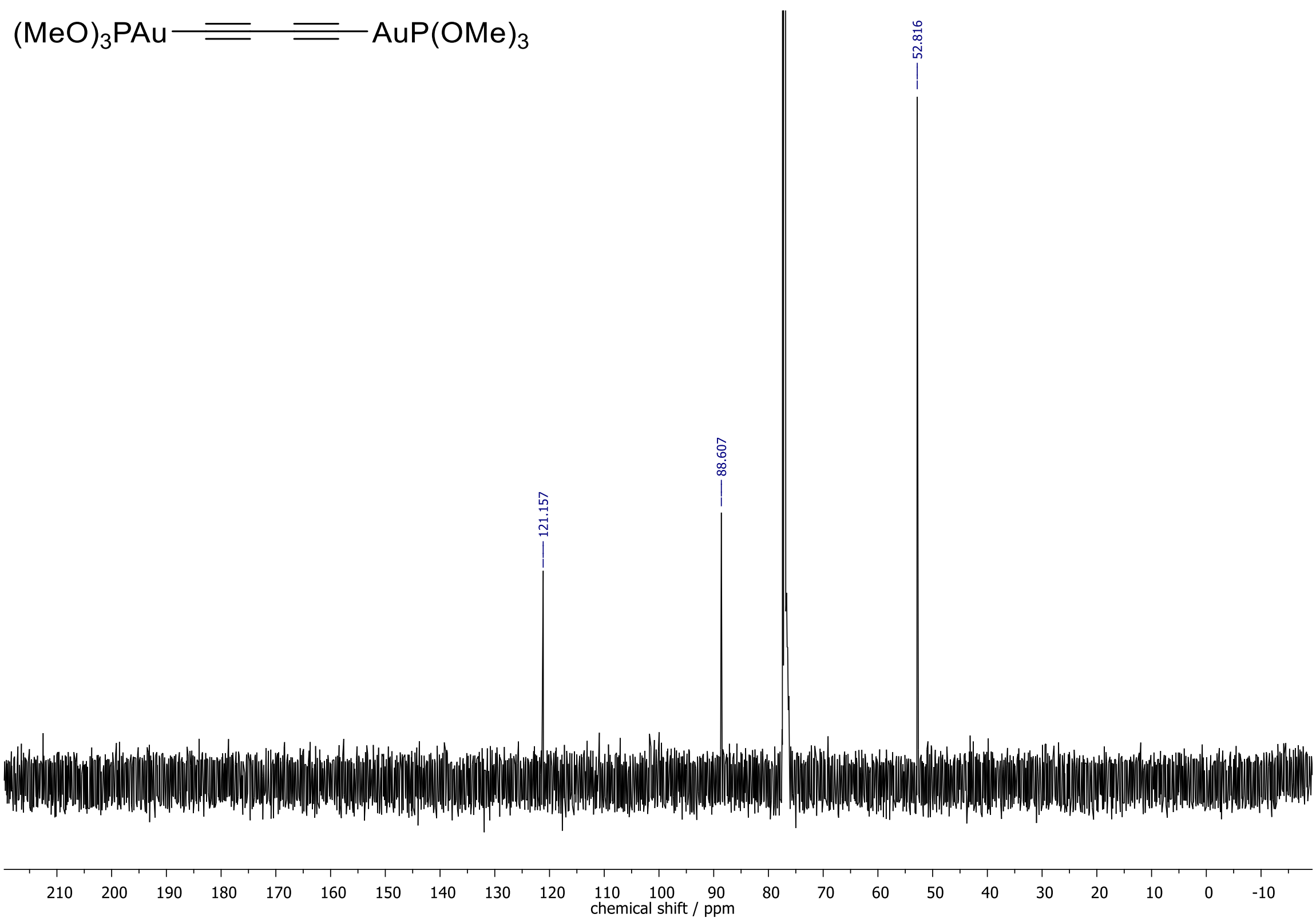

Supplementary Figure 8: $^{13}C\{^{1}H,^{31}P\}$ NMR spectrum of **1(4).**

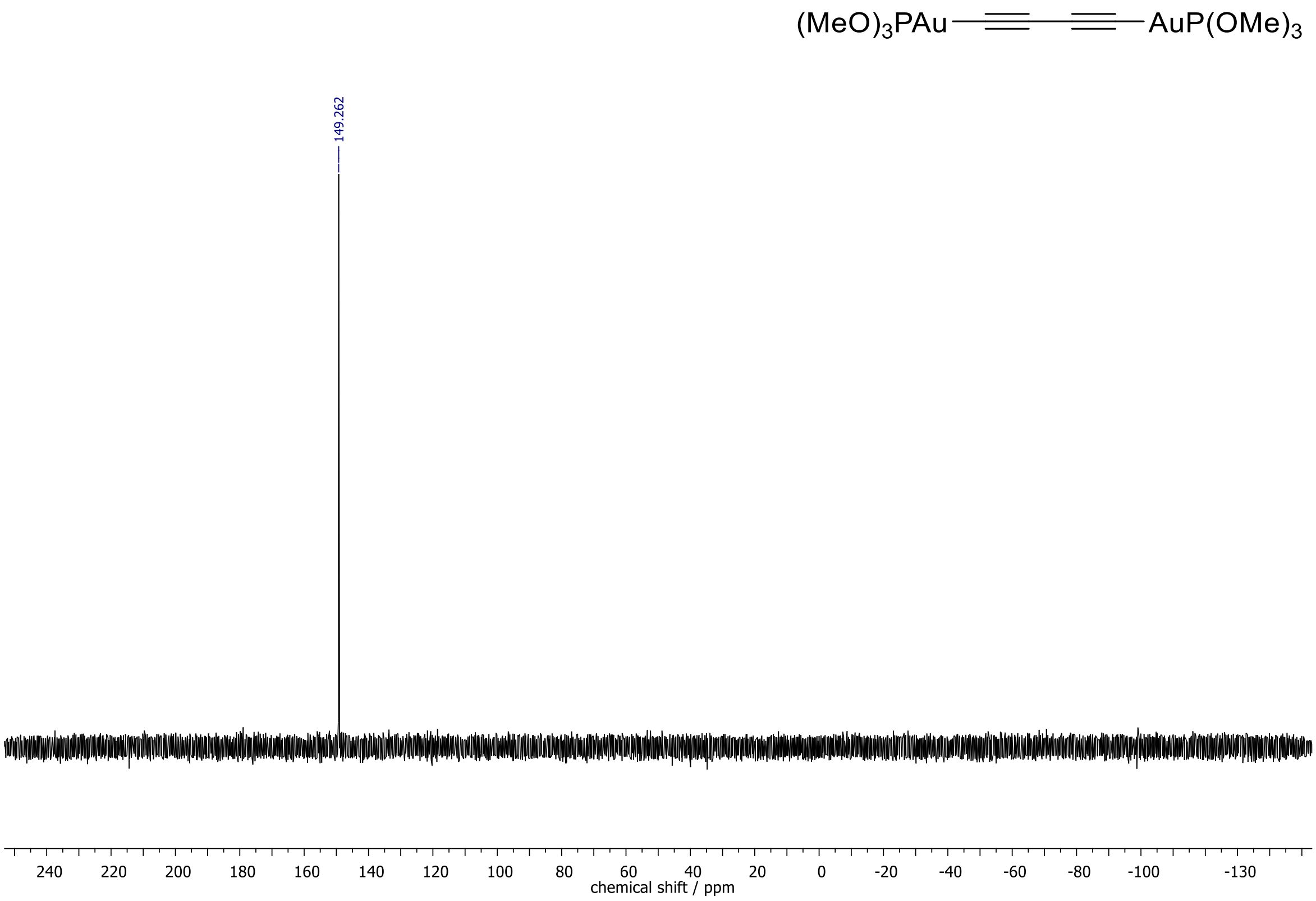

Supplementary Figure 9: $^{31}P\{^{1}H\}$ NMR spectrum of **1(4)**.

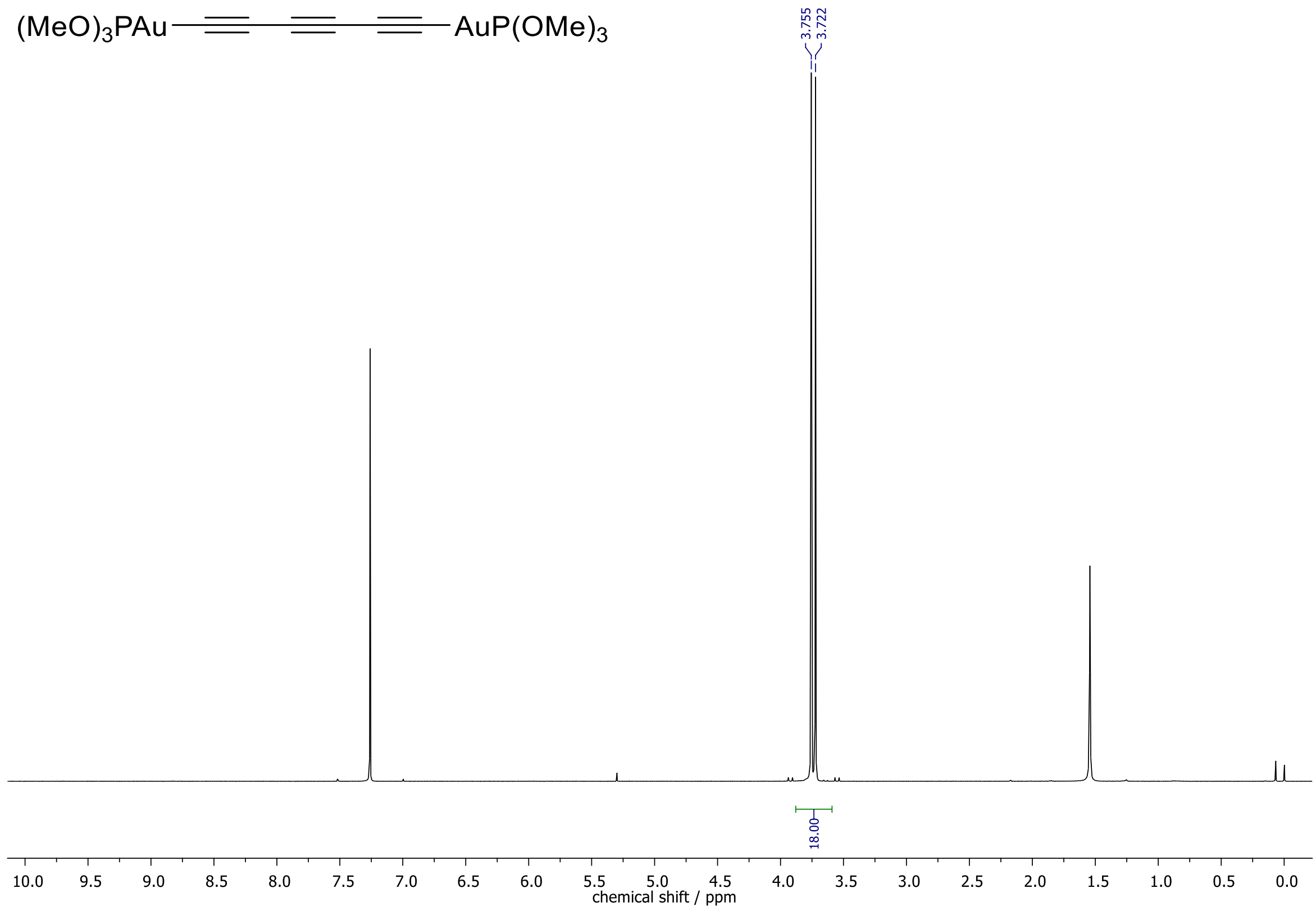

Supplementary Figure 10: ^{1}H NMR spectrum of **1(6)**.

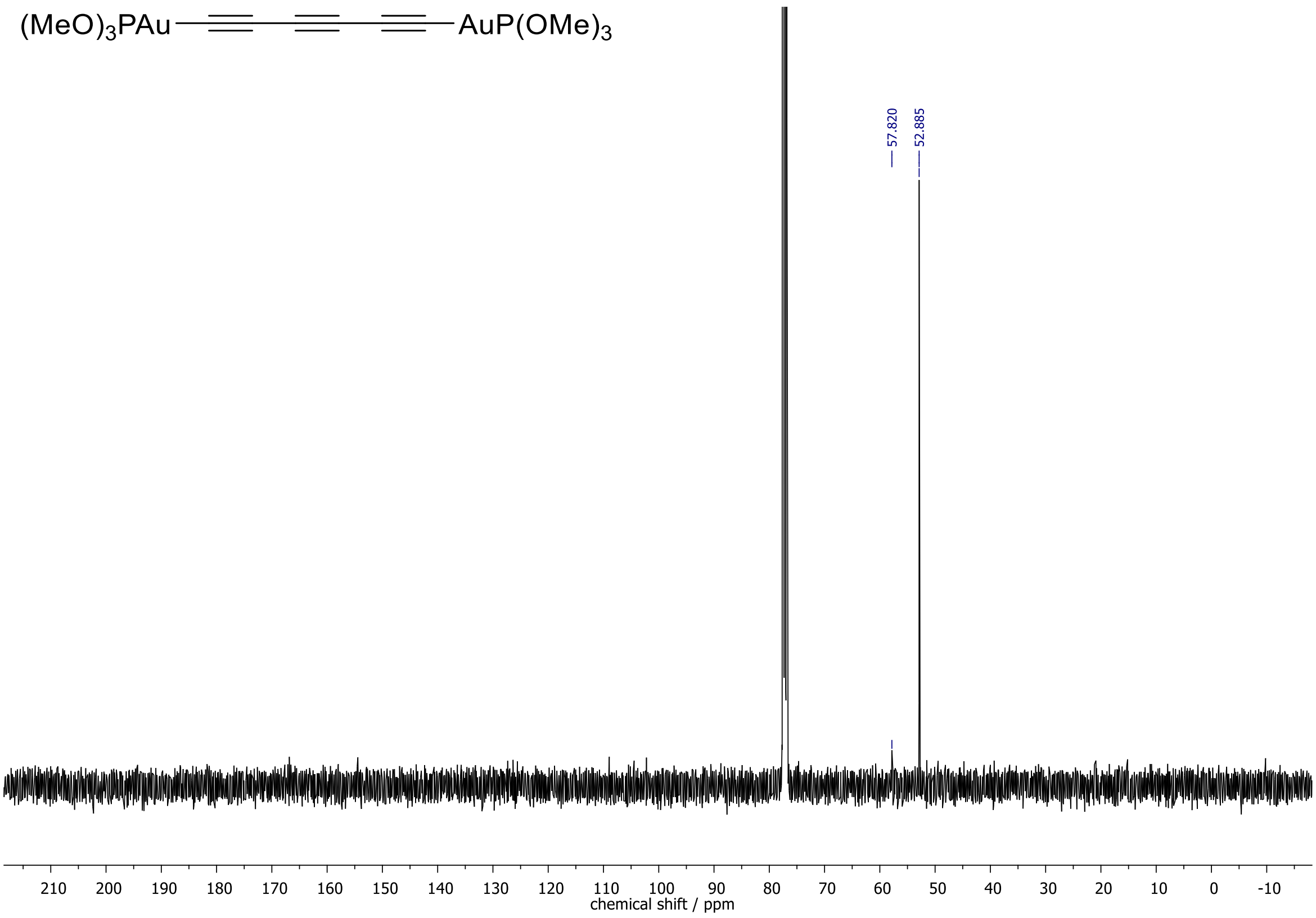

Supplementary Figure 11: $^{13}C\{^{1}H\}$ NMR spectrum of **1(6)**.

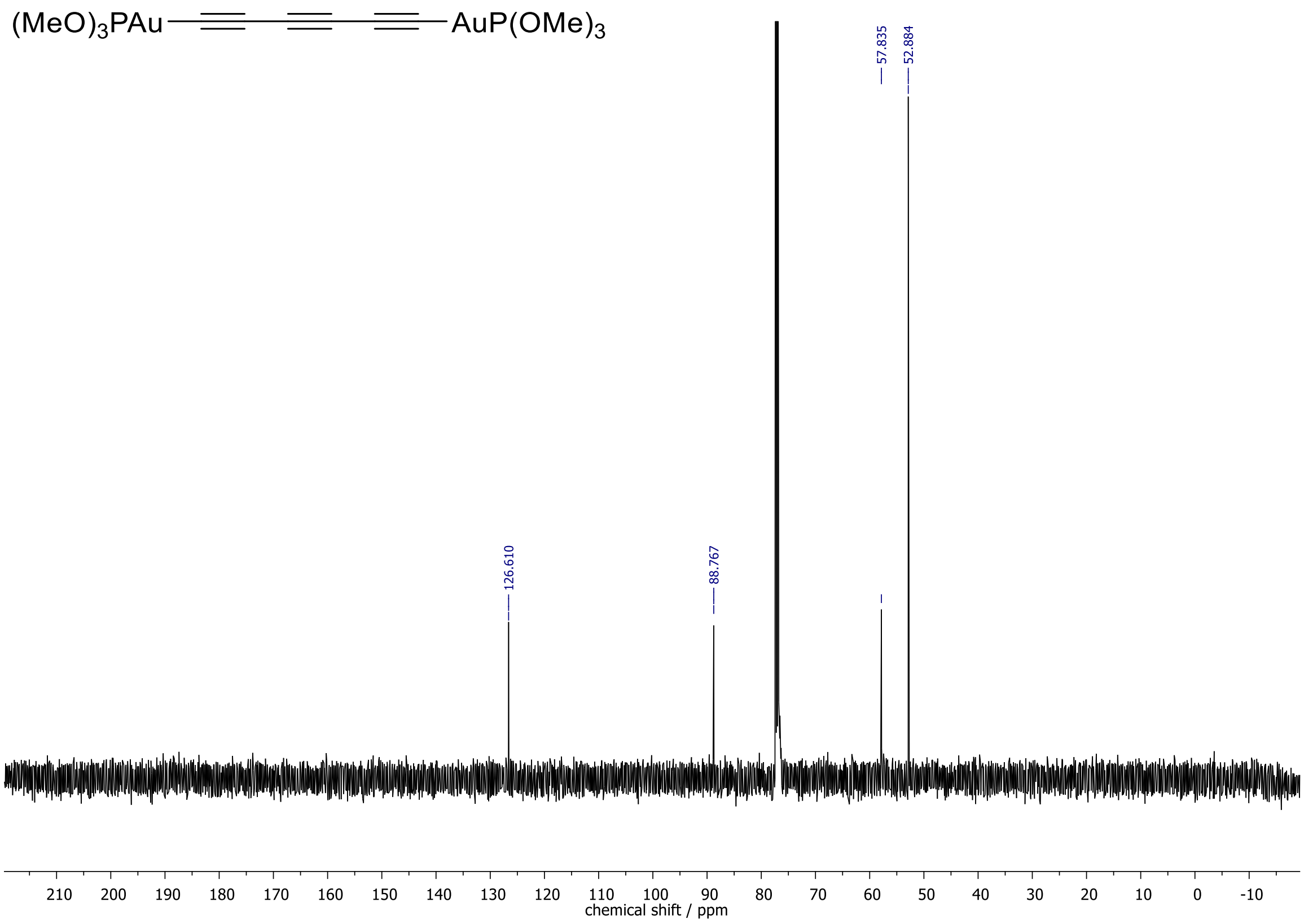

Supplementary Figure 12: $^{13}C\{^{1}H,^{31}P\}$ NMR spectrum of **1(6)**.

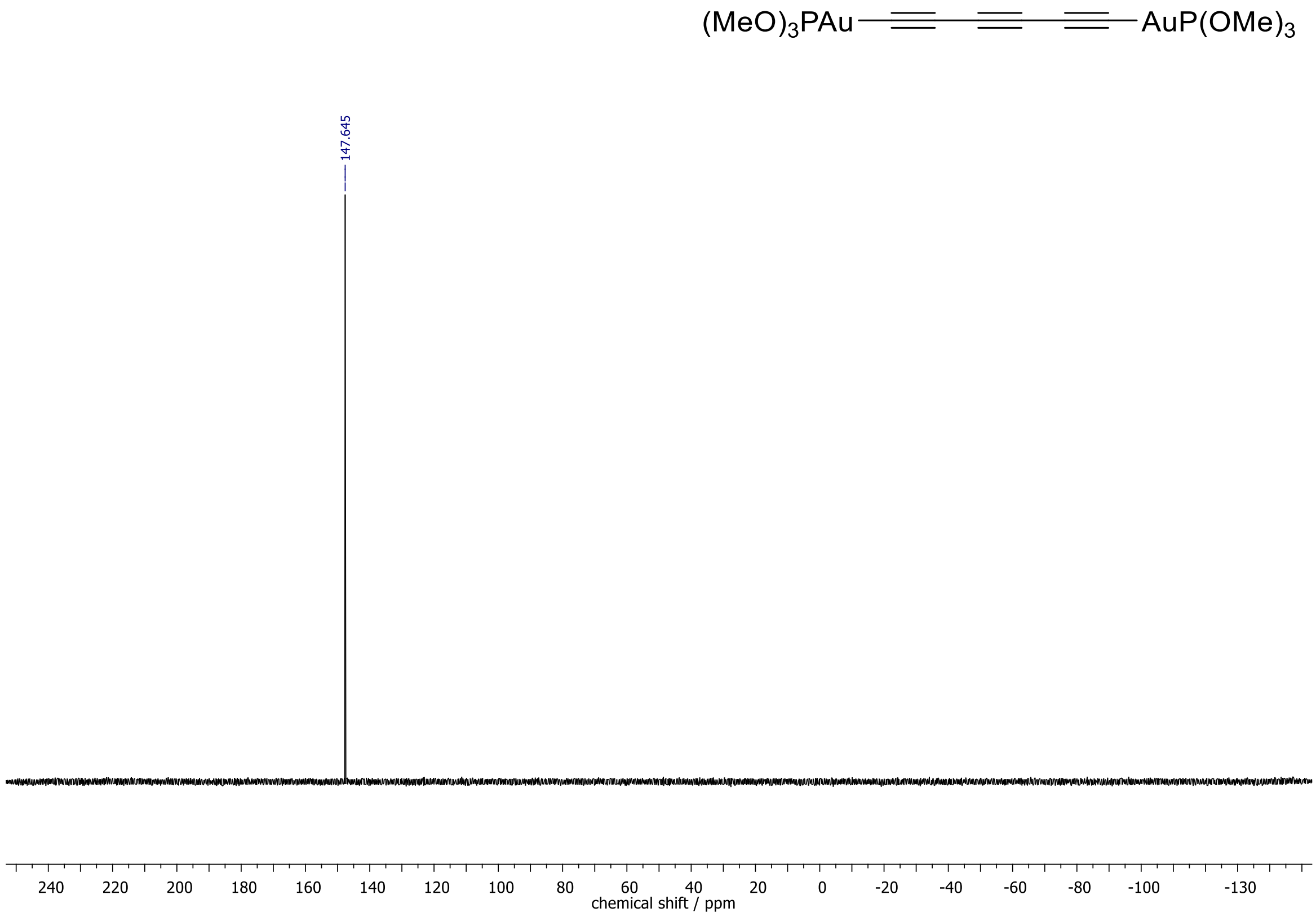

Supplementary Figure 13: $^{31}P\{^{1}H\}$ NMR spectrum of **1(6)**.

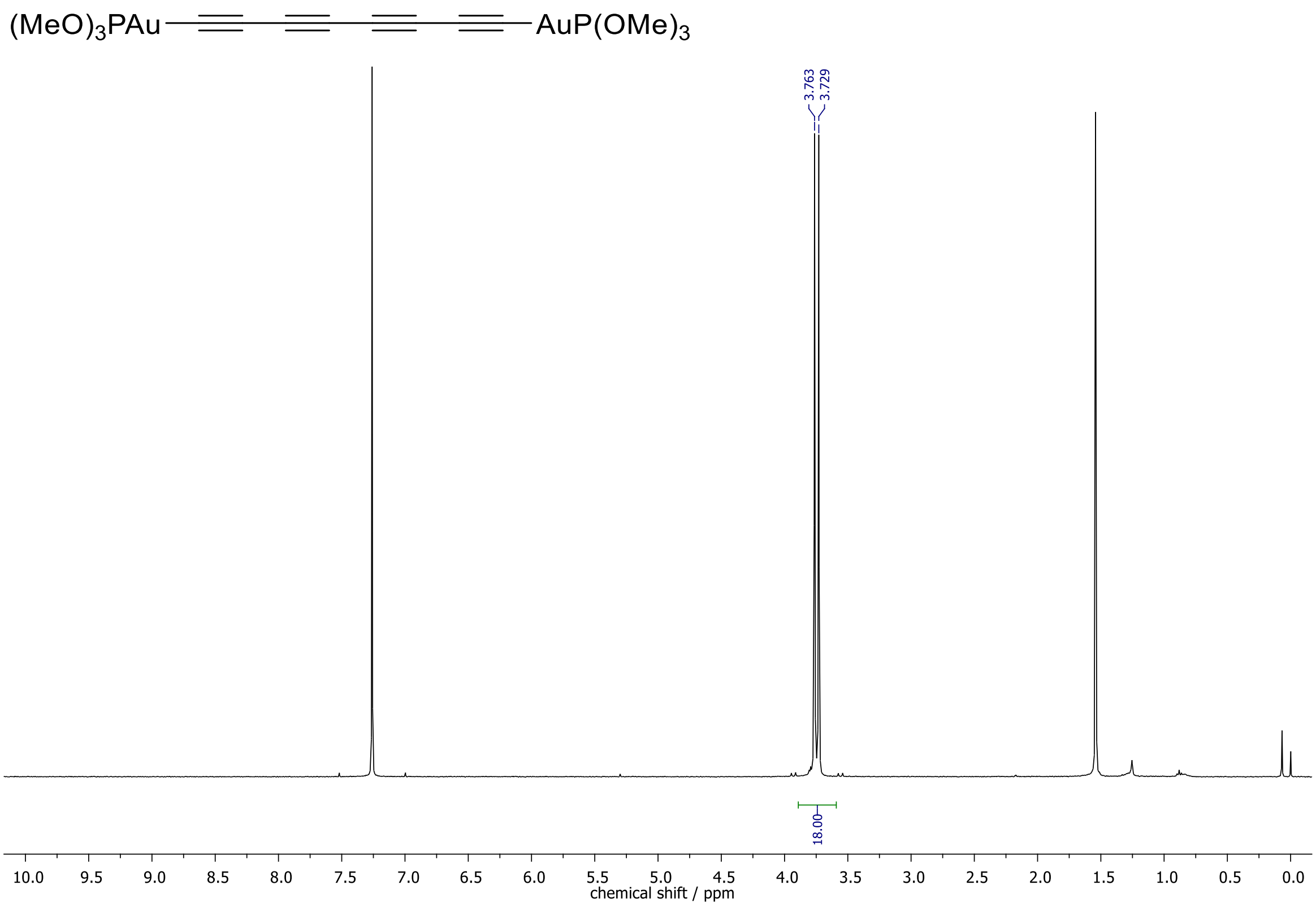

Supplementary Figure 14: ^{1}H NMR spectrum of **1(8)**.

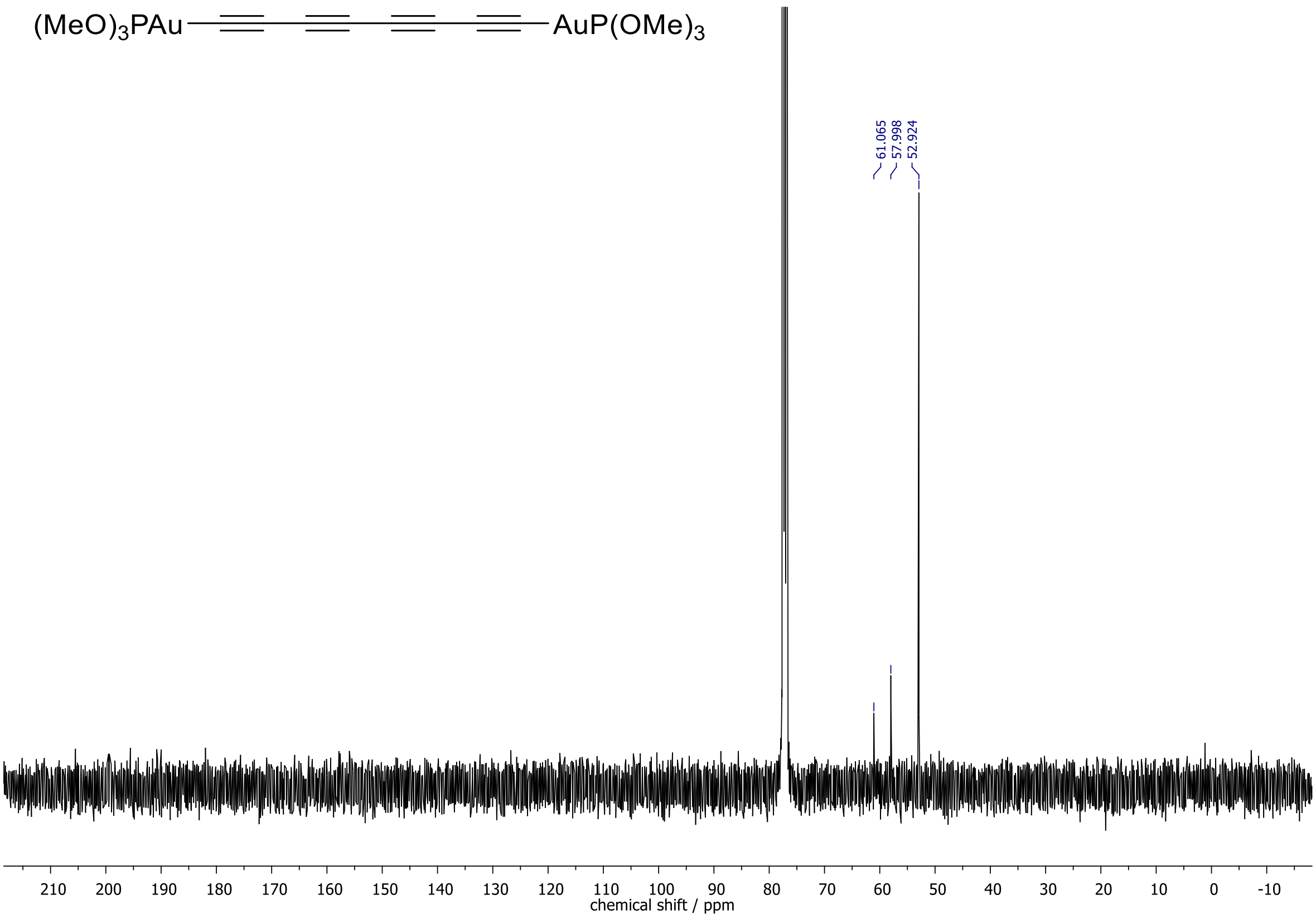

Supplementary Figure 15: $^{13}C\{^{1}H\}$ NMR spectrum of **1(8)**.

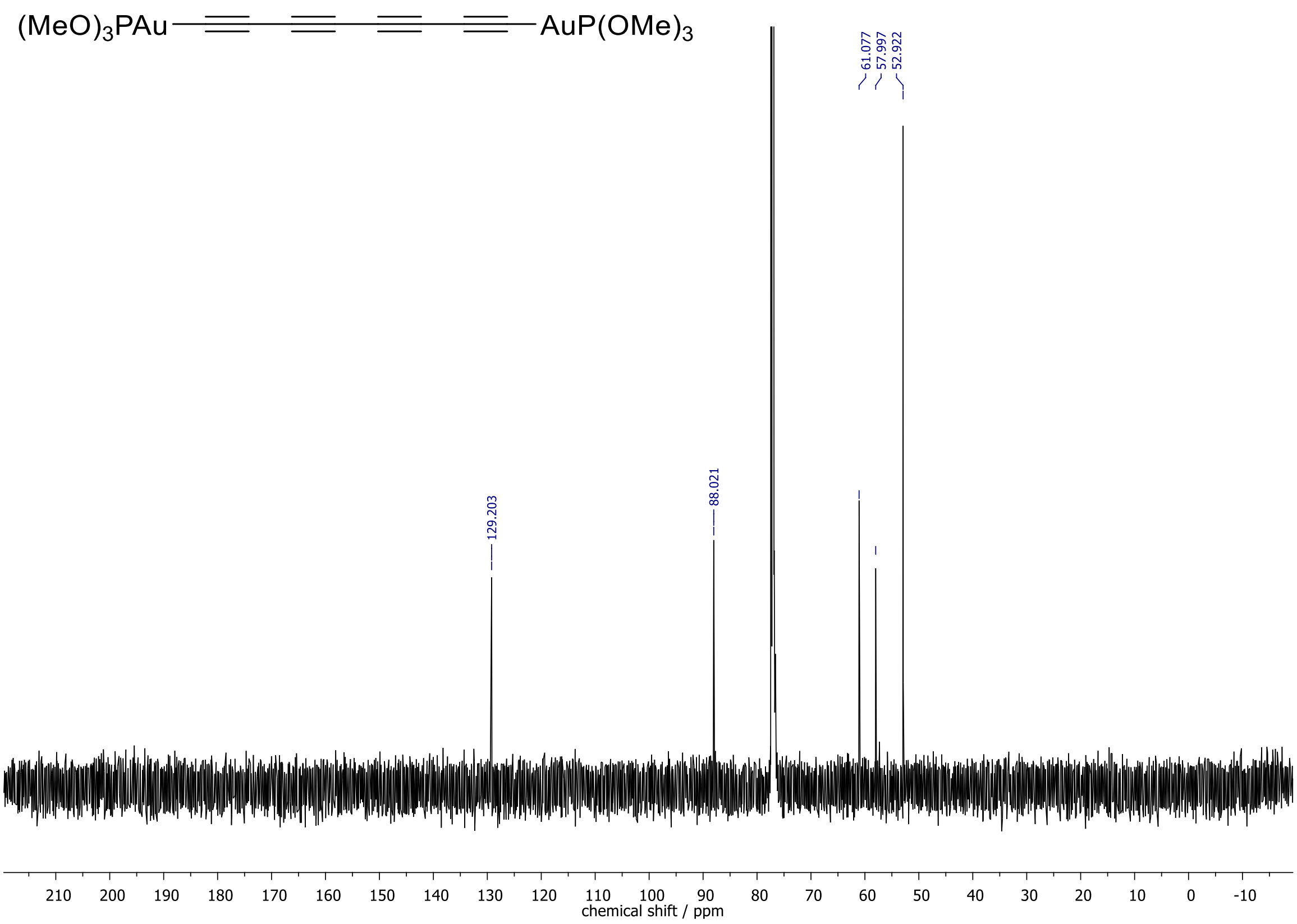

Supplementary Figure 16: $^{13}C\{^{1}H,^{31}P\}$ NMR spectrum of **1(8).**

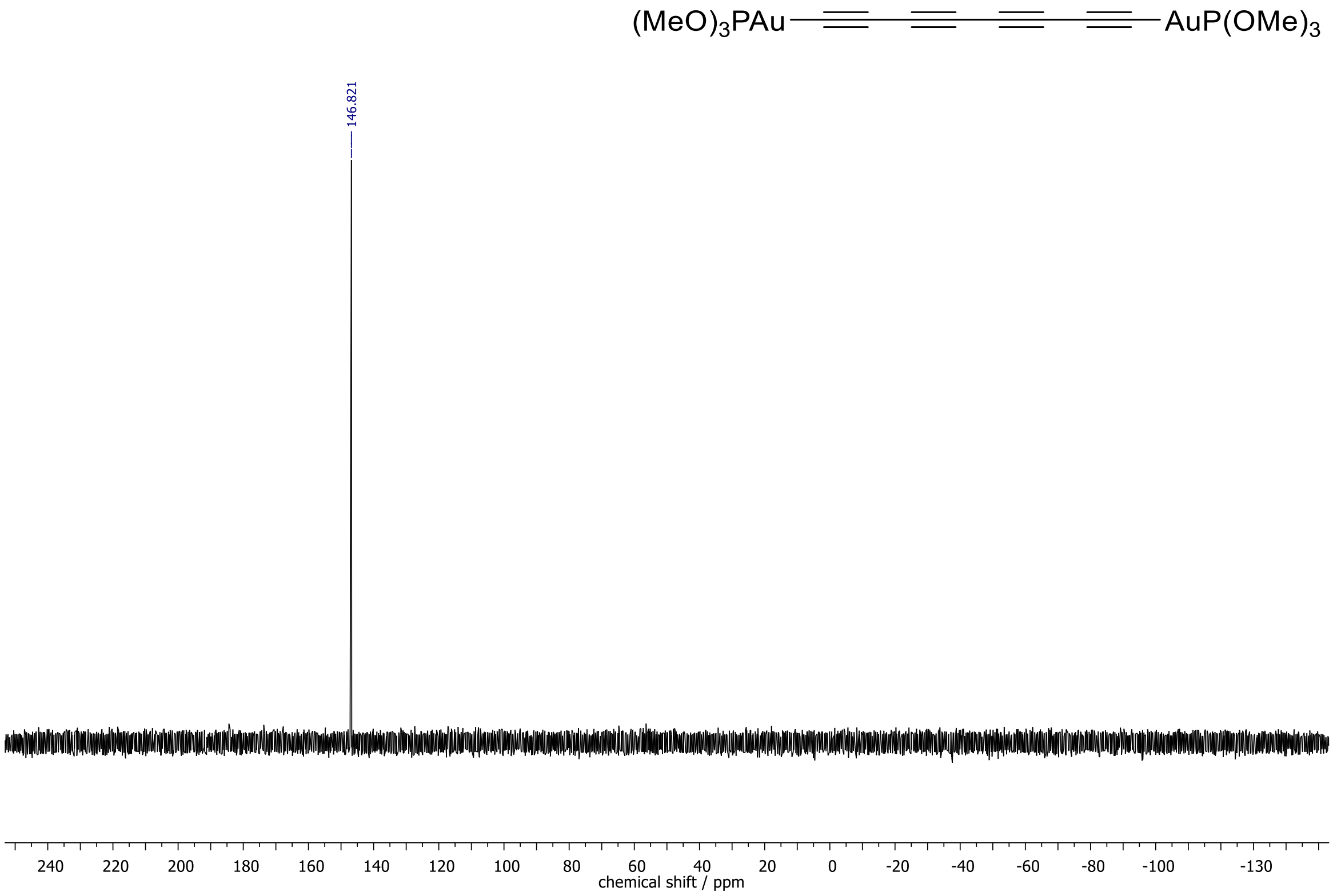

Supplementary Figure 17**:** $^{31}P\{^{1}H\}$ NMR spectrum of **1(8)**.

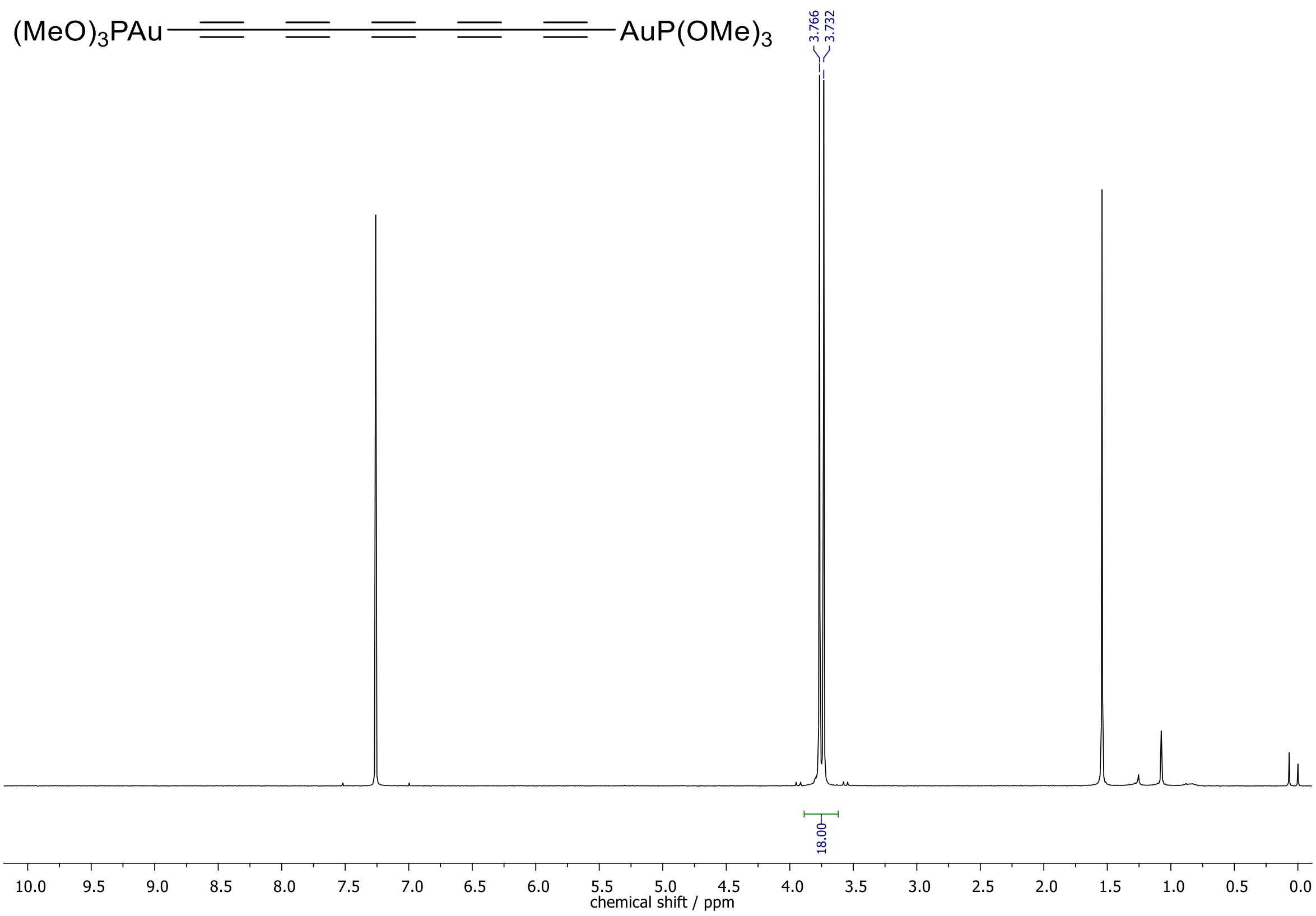

Supplementary Figure 18: ^{1}H NMR spectrum of **1(10)**.

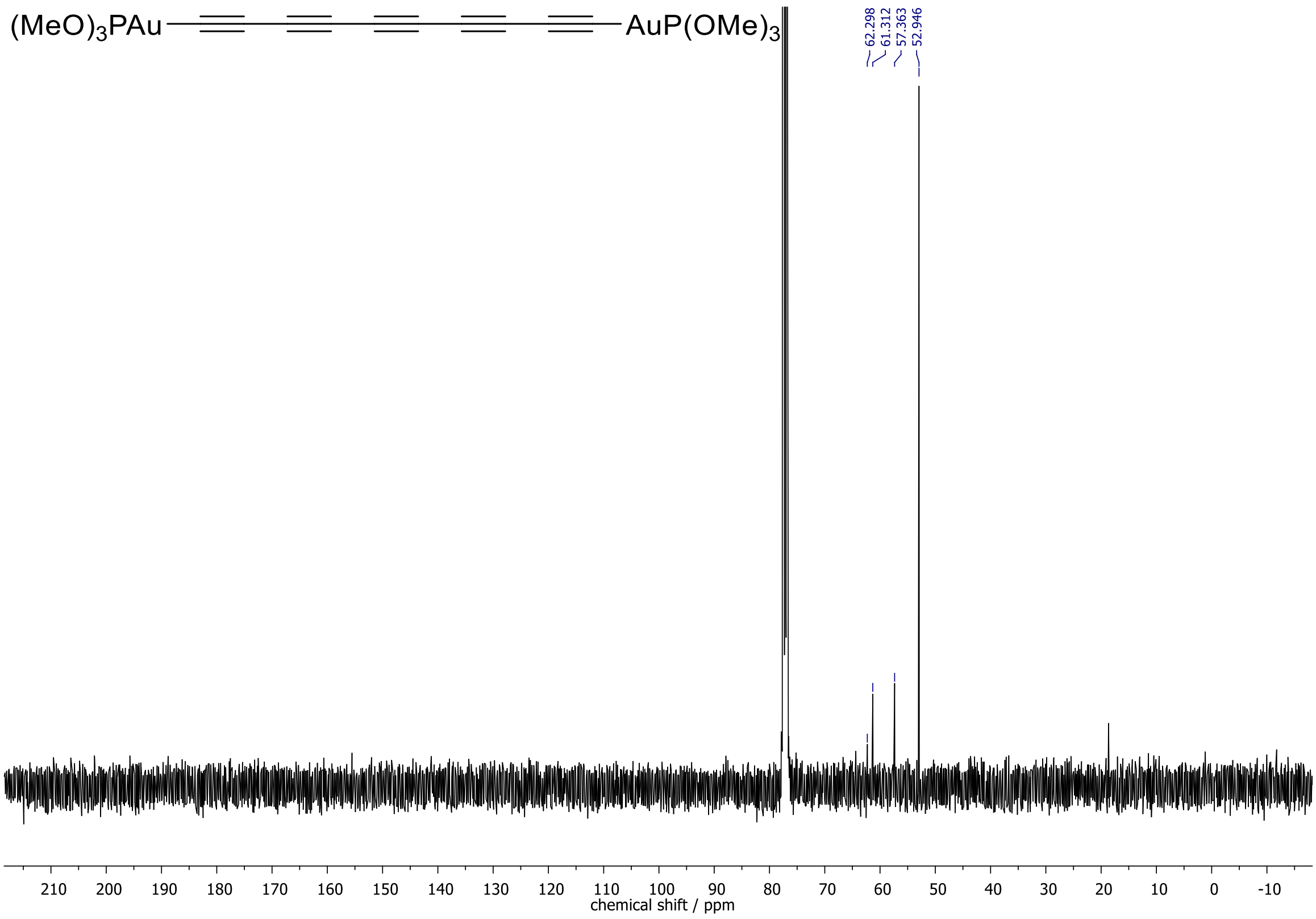

Supplementary Figure 19: $^{13}C\{^1H\}$ NMR spectrum of **1(10)**.

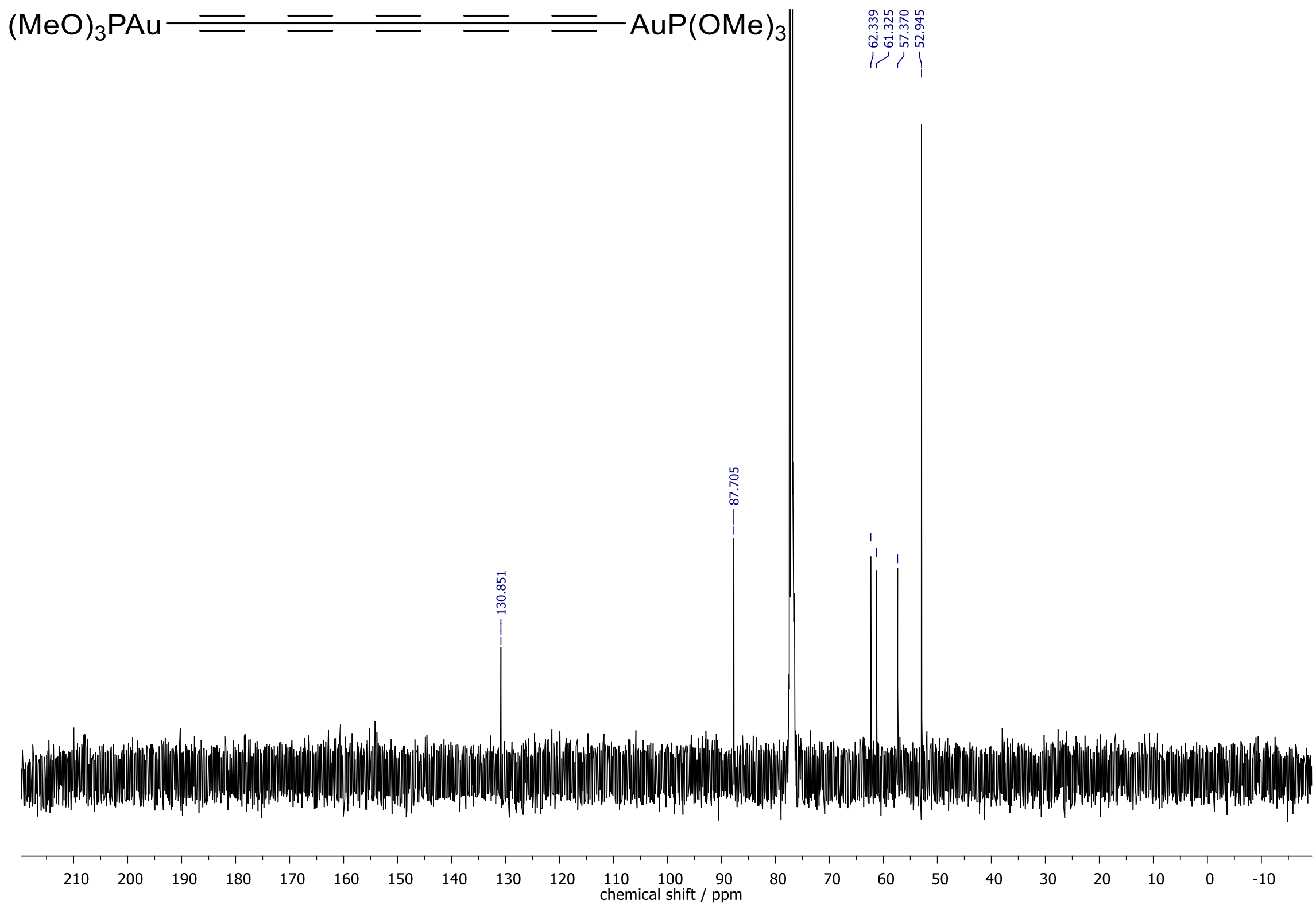

Supplementary Figure 20: $^{13}C\{^1H,^{31}P\}$ NMR spectrum of **1(10).**

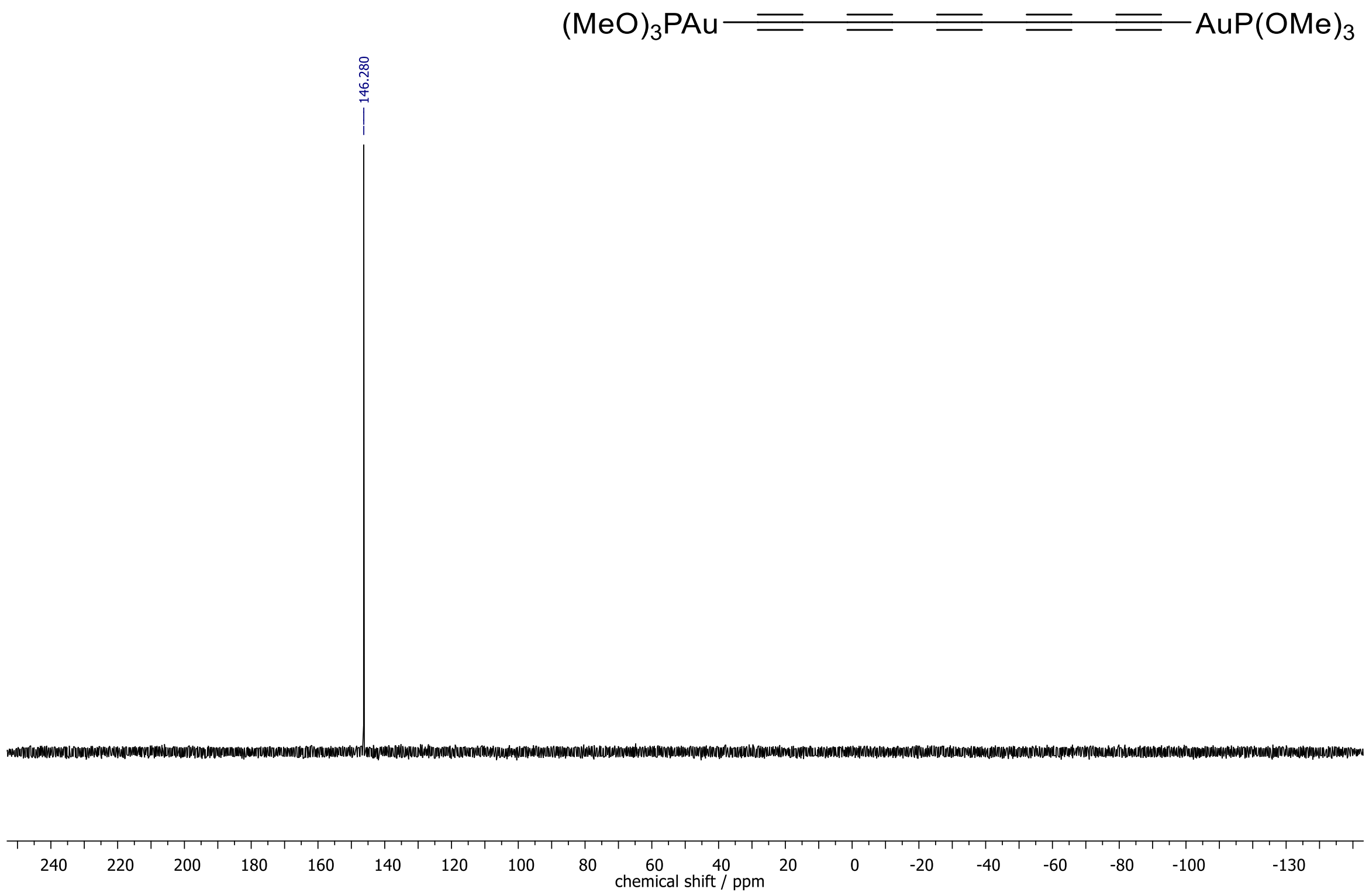

Supplementary Figure 21: $^{31}P\{^{1}H\}$ NMR spectrum of **1(10)**.

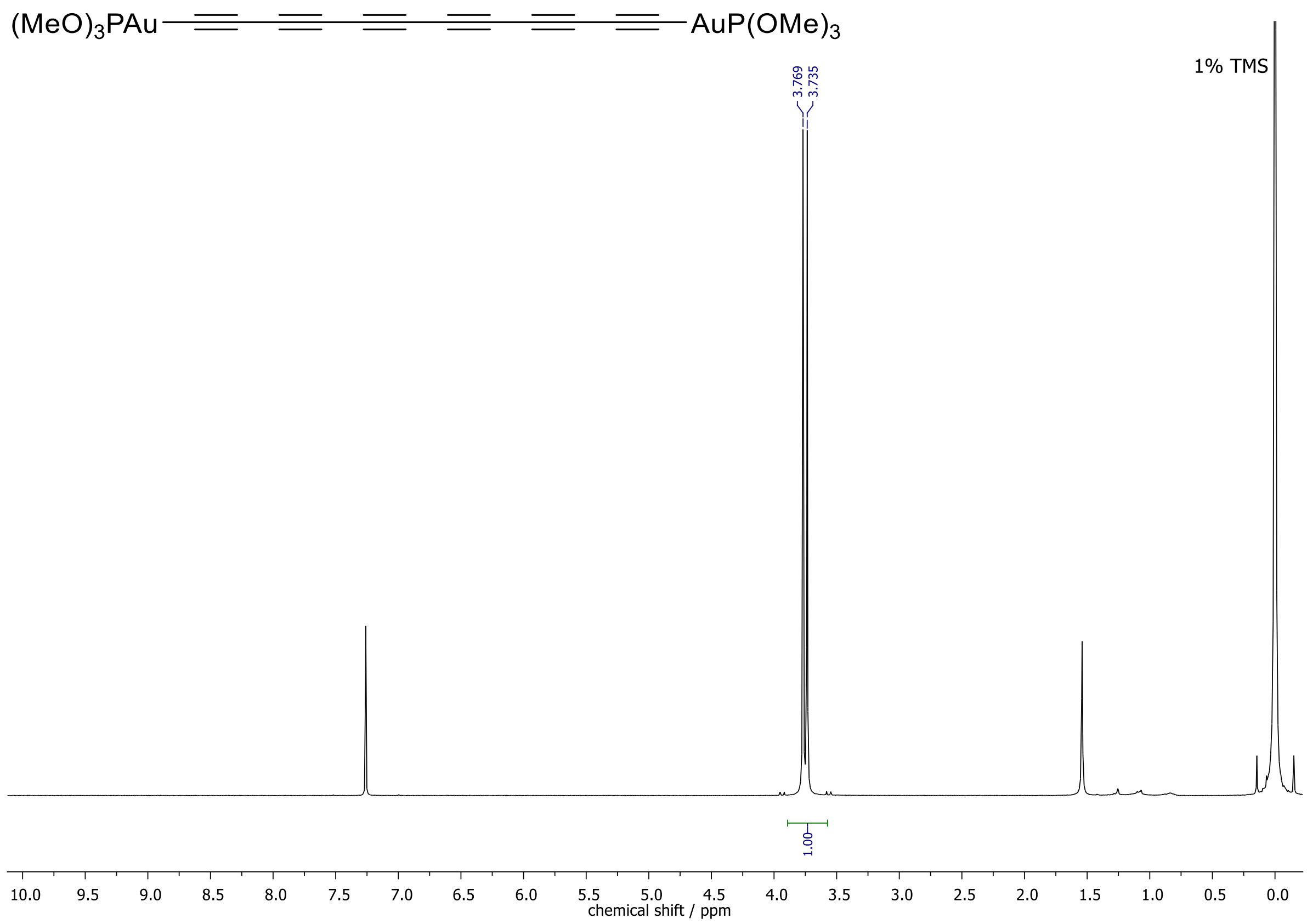

Supplementary Figure 22: ^{1}H NMR spectrum of **1(12)**.

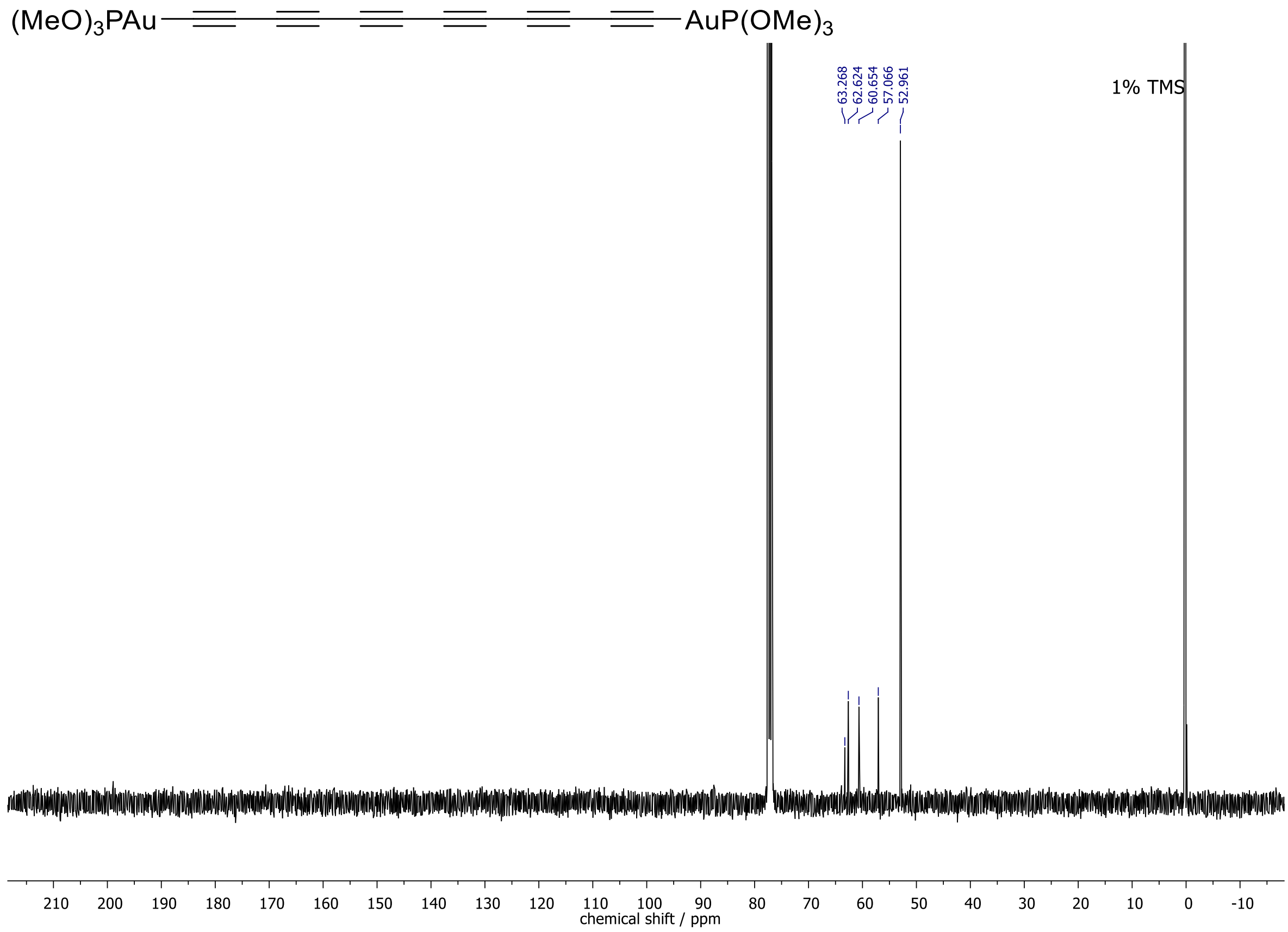

Supplementary Figure 23: $^{13}C\{^{1}H\}$ NMR spectrum of **1(12)**.

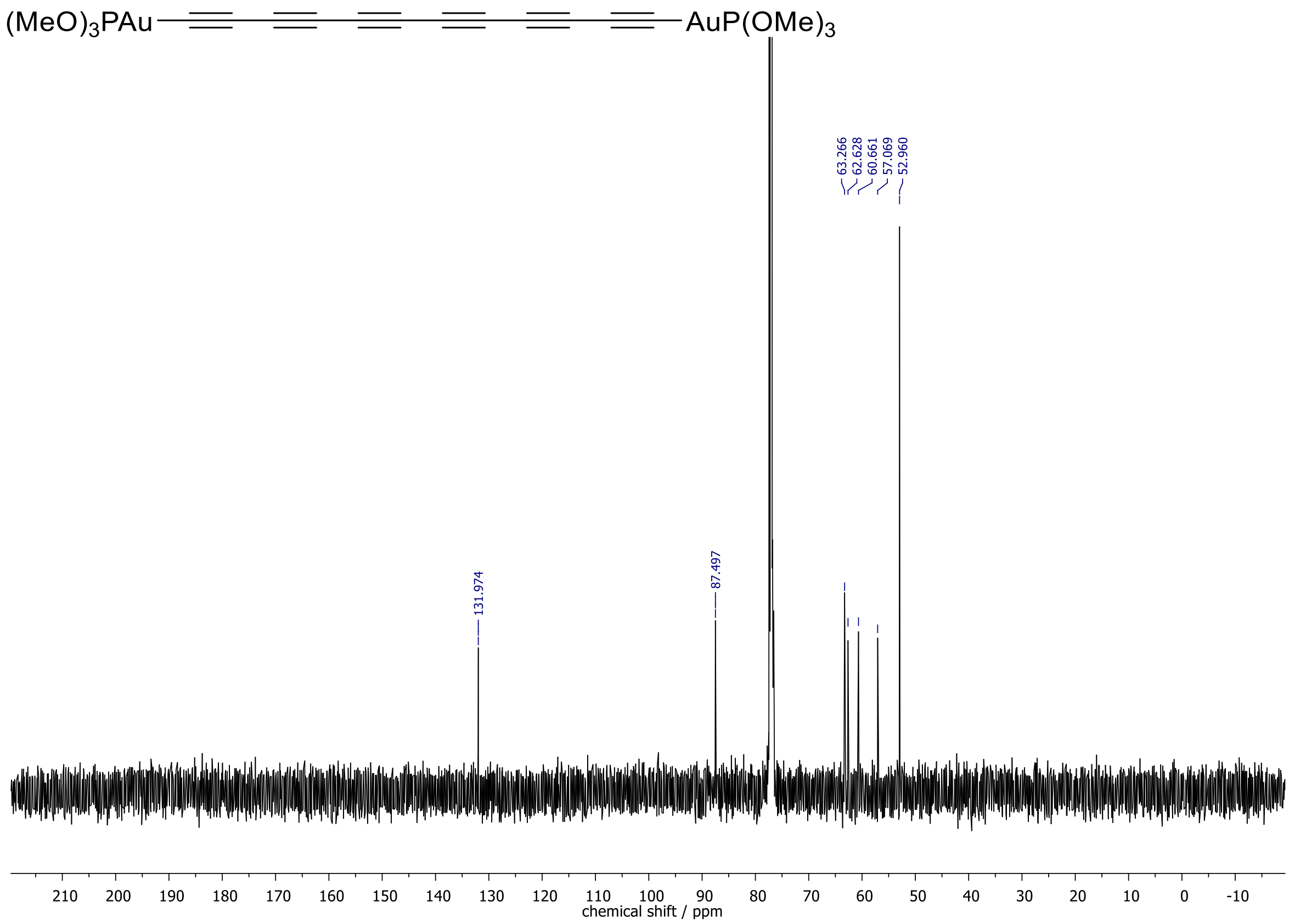

Supplementary Figure 24: $^{13}C\{^{1}H,^{31}P\}$ NMR spectrum of **1(12).**

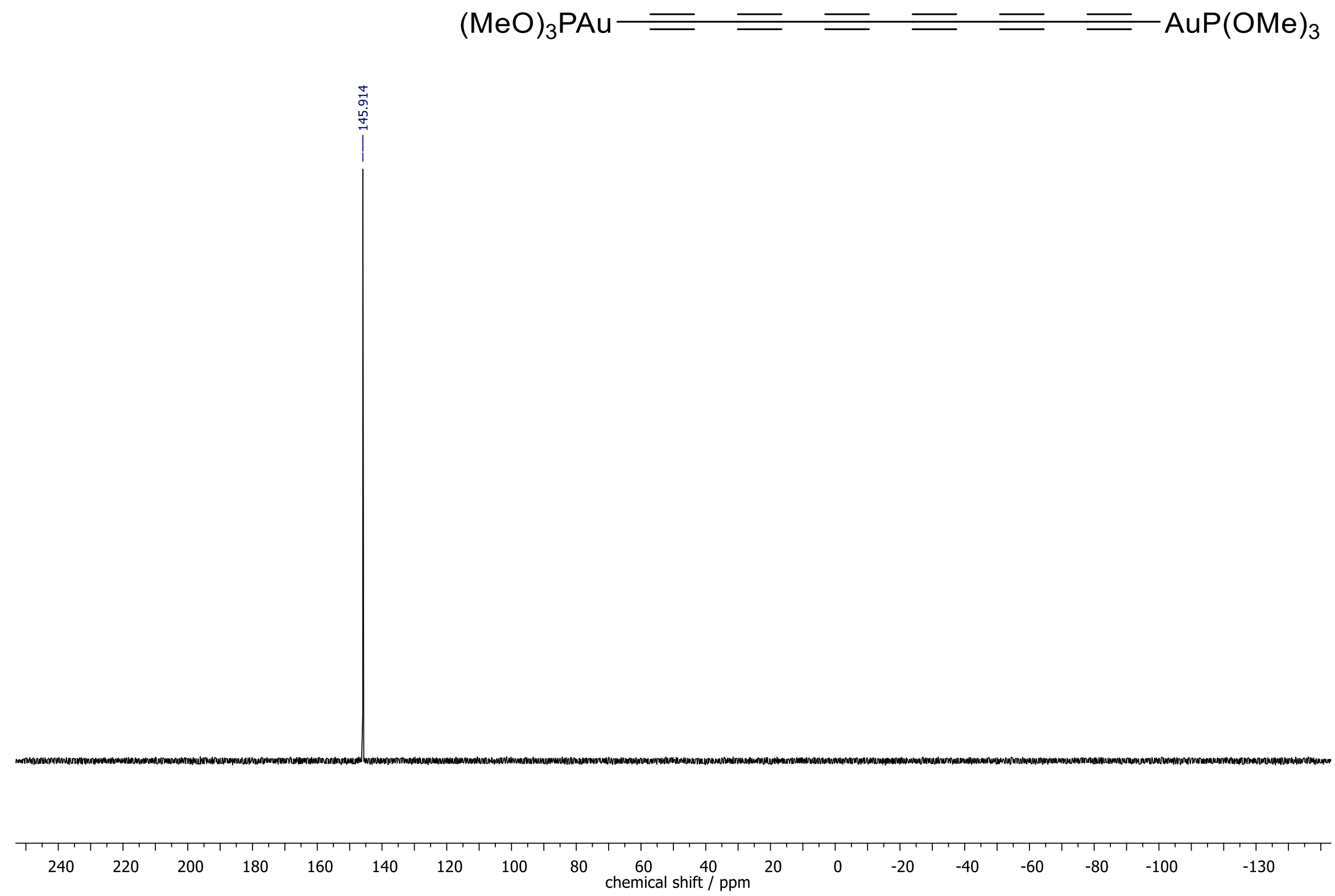

Supplementary Figure 25: $^{31}P\{^{1}H\}$ NMR spectrum of **1(12)**.

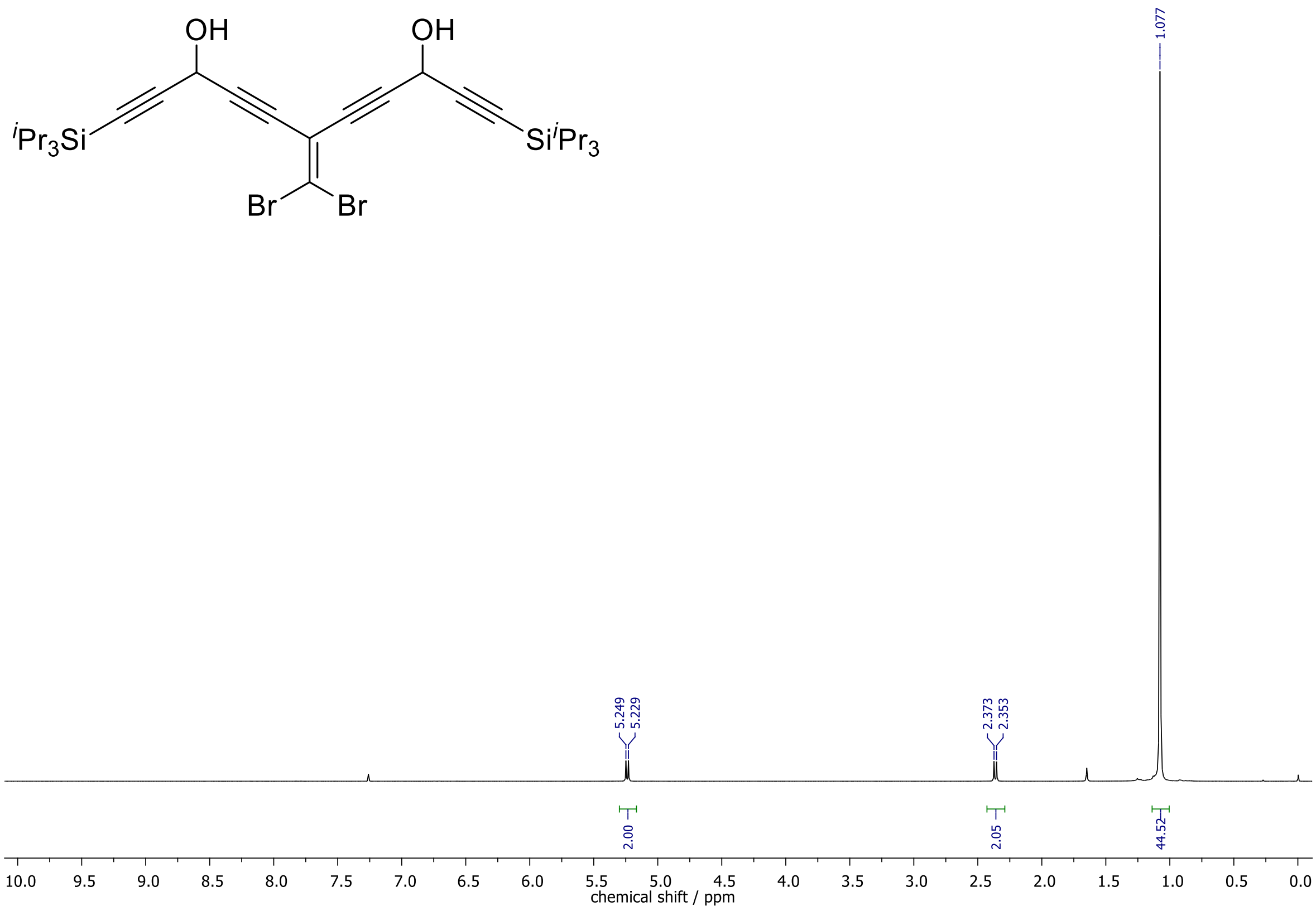

Supplementary Figure 26: ^{1}H NMR spectrum of **S1**.

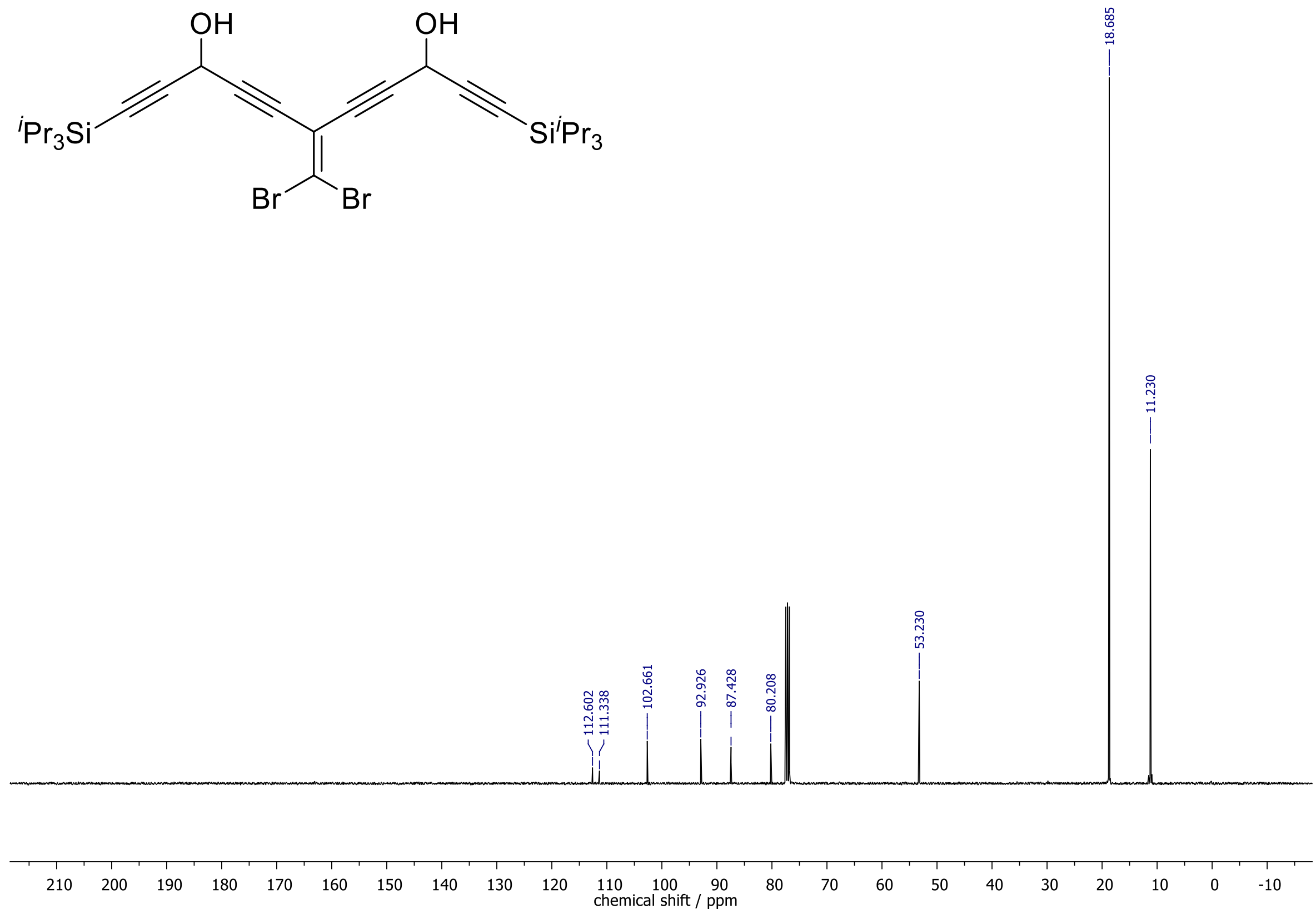

Supplementary Figure 27: $^{13}C\{^1H\}$ NMR spectrum of **S1**.

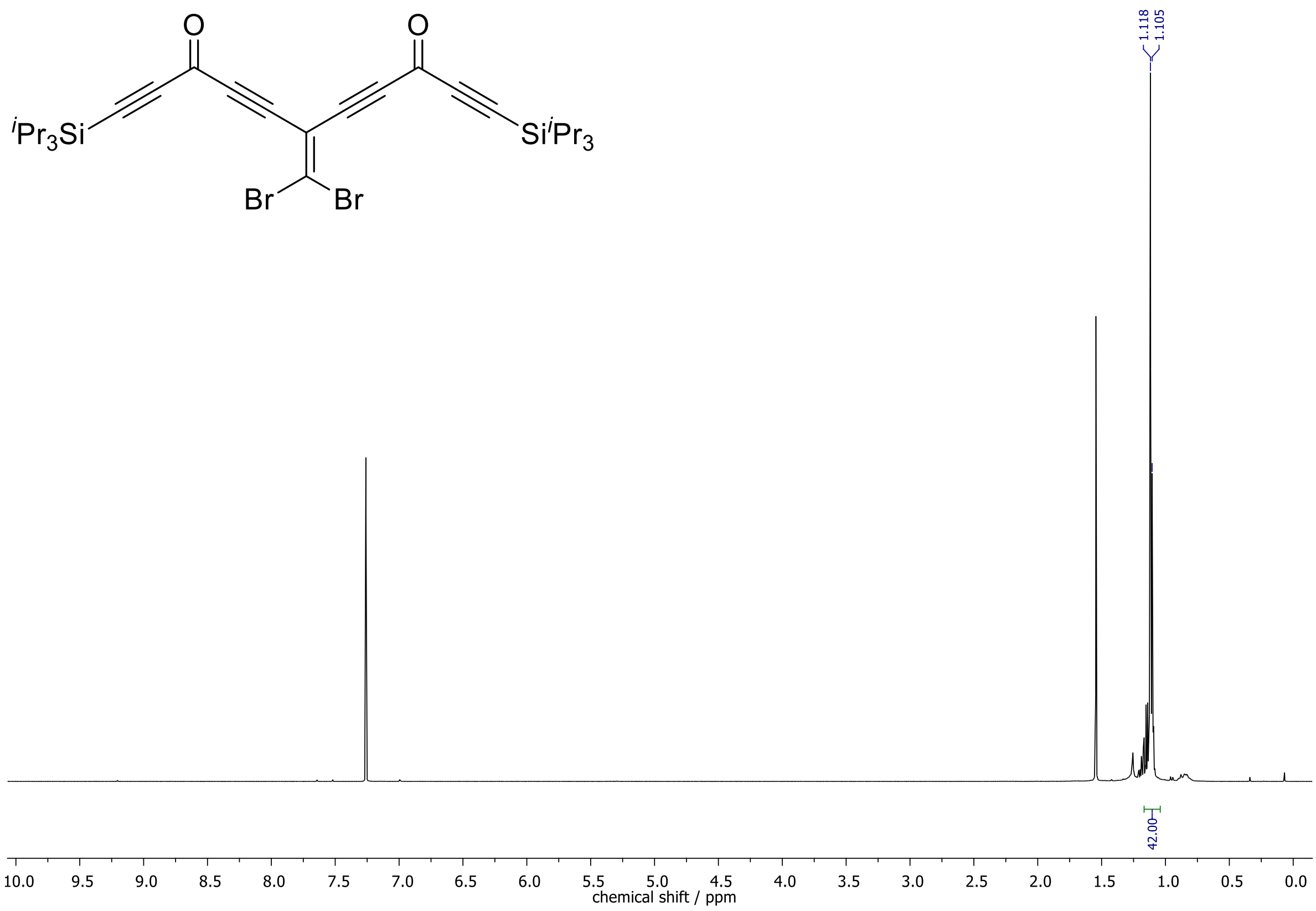

Supplementary Figure 28: ^1H NMR spectrum of **S2**.

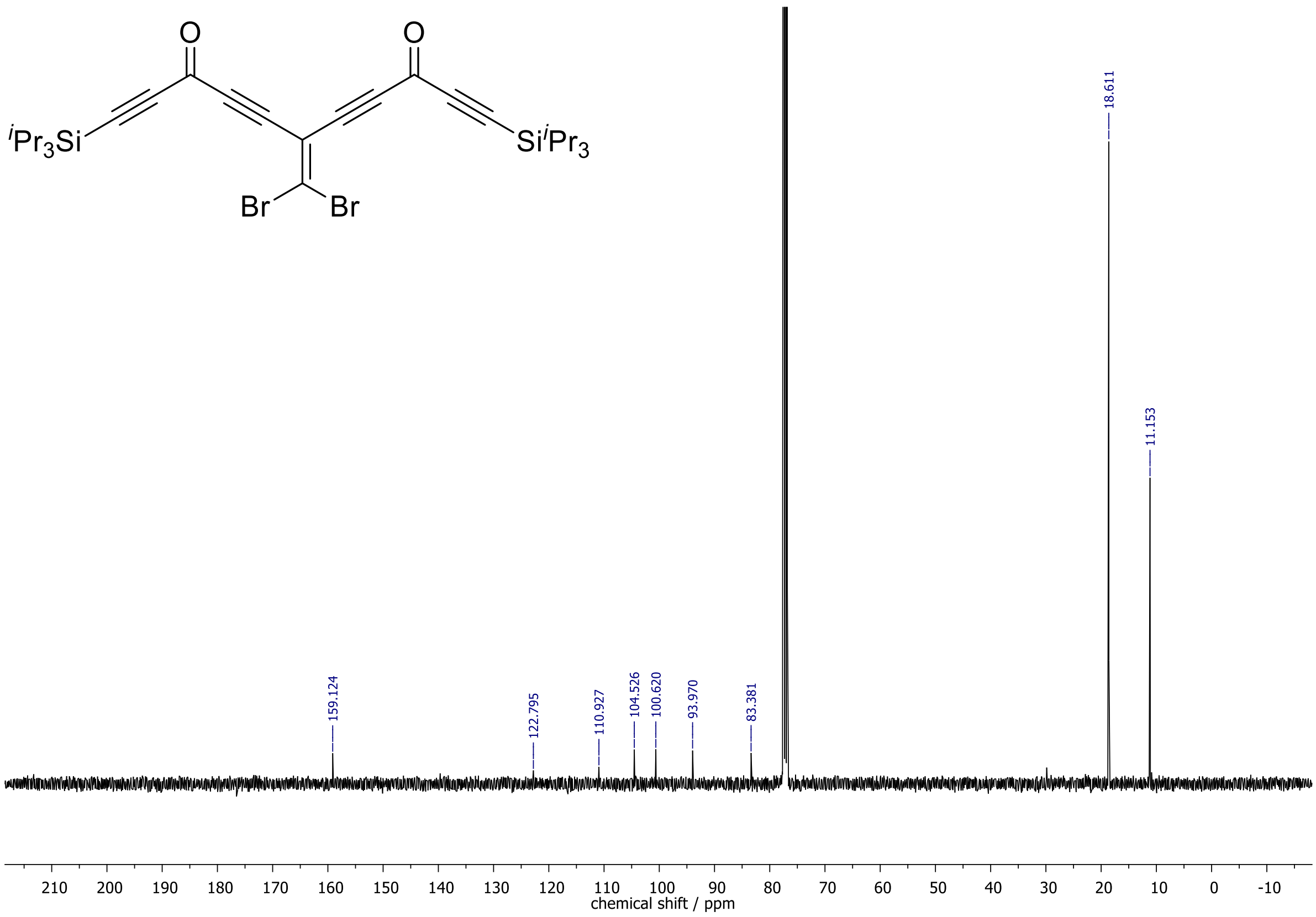

Supplementary Figure 29: ^{13}C{^{1}H} NMR spectrum of **S2**.

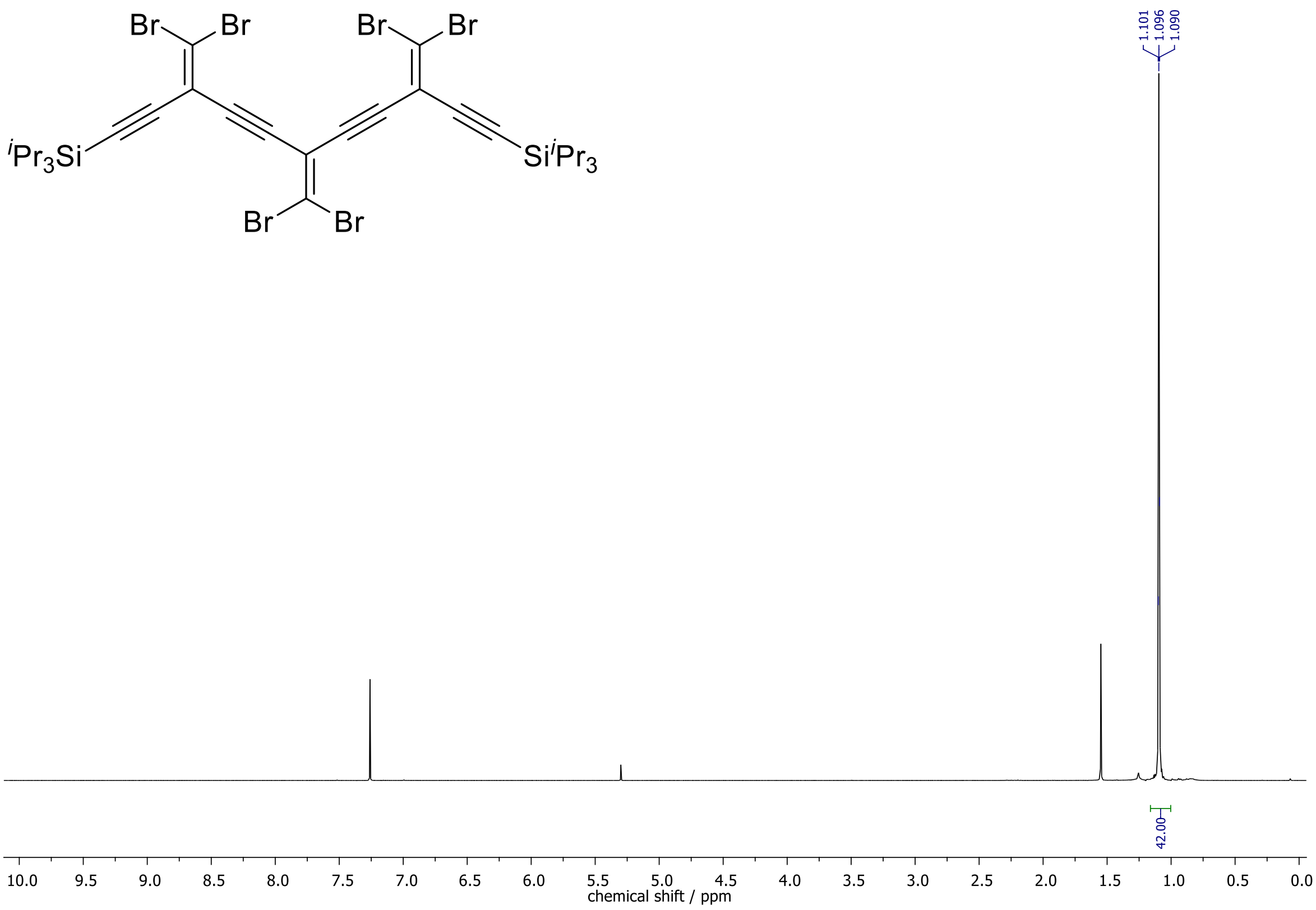

Supplementary Figure 30: ^{1}H NMR spectrum of **S3**.

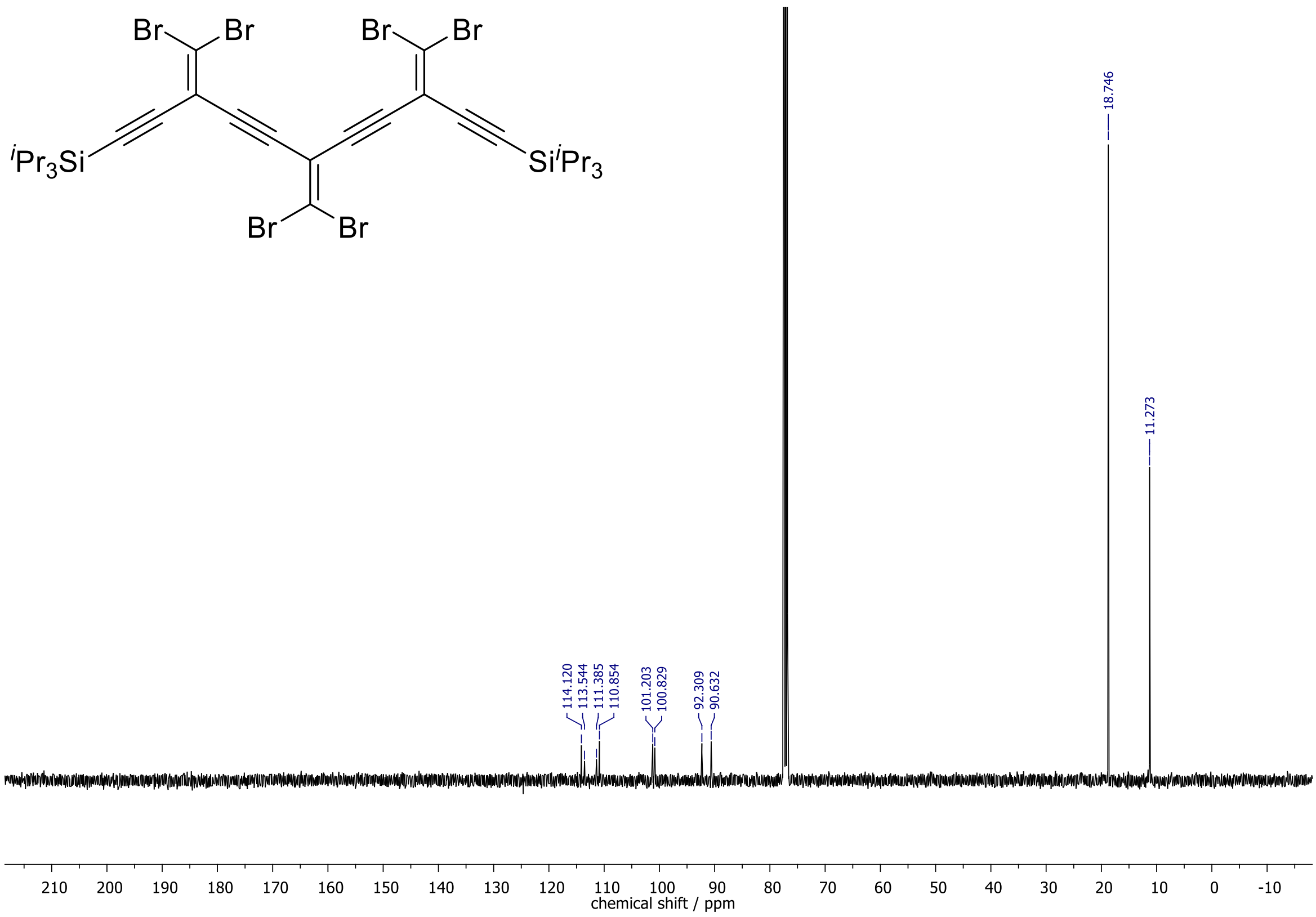

Supplementary Figure 31: $^{13}C\{^{1}H\}$ NMR spectrum of **S3**.

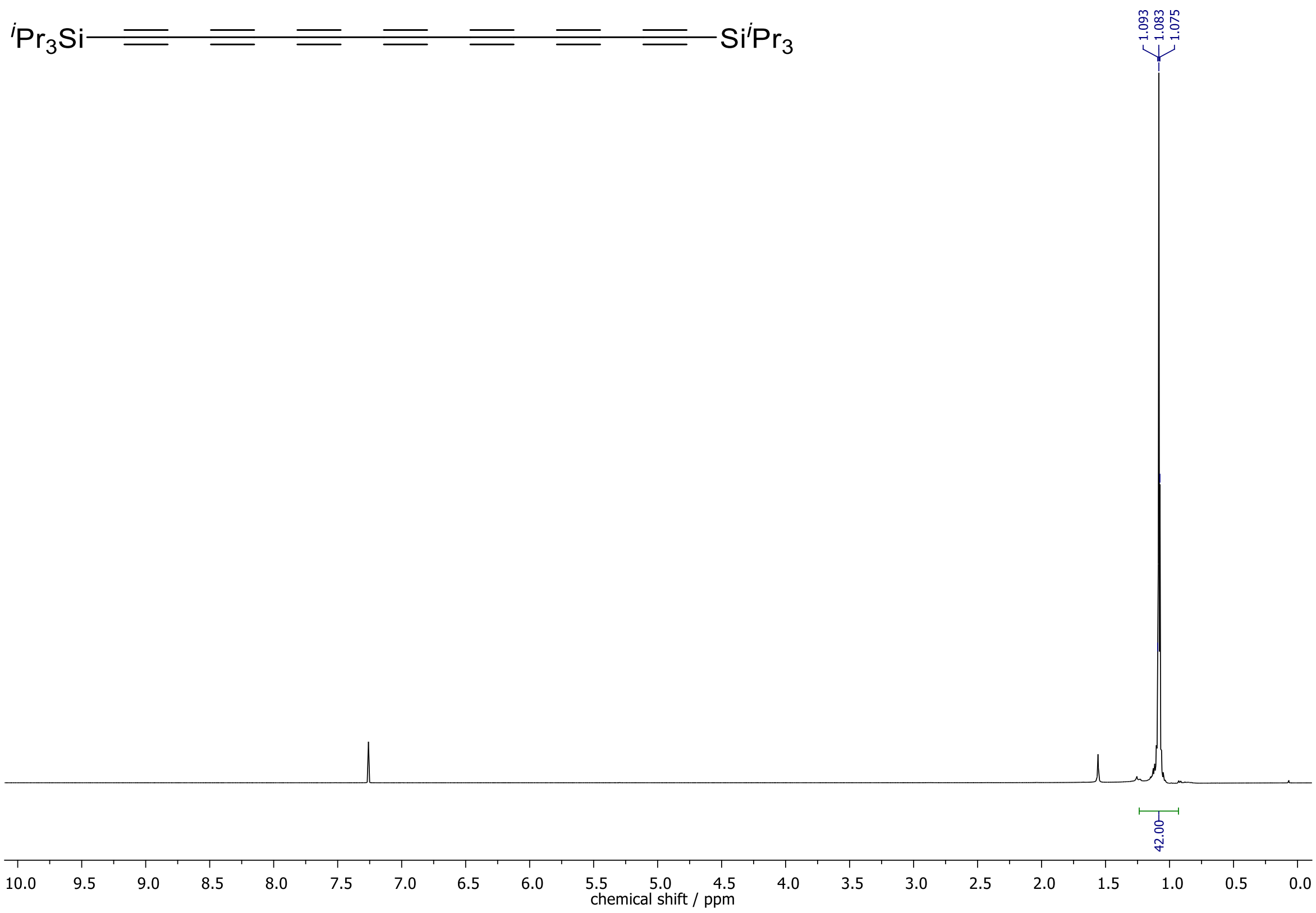

Supplementary Figure 32: The ^{1}H NMR spectrum of **1,14-bis(triisopropylsilyl)-1,3,5,7,9,11,13-tetradecaheptayne**.

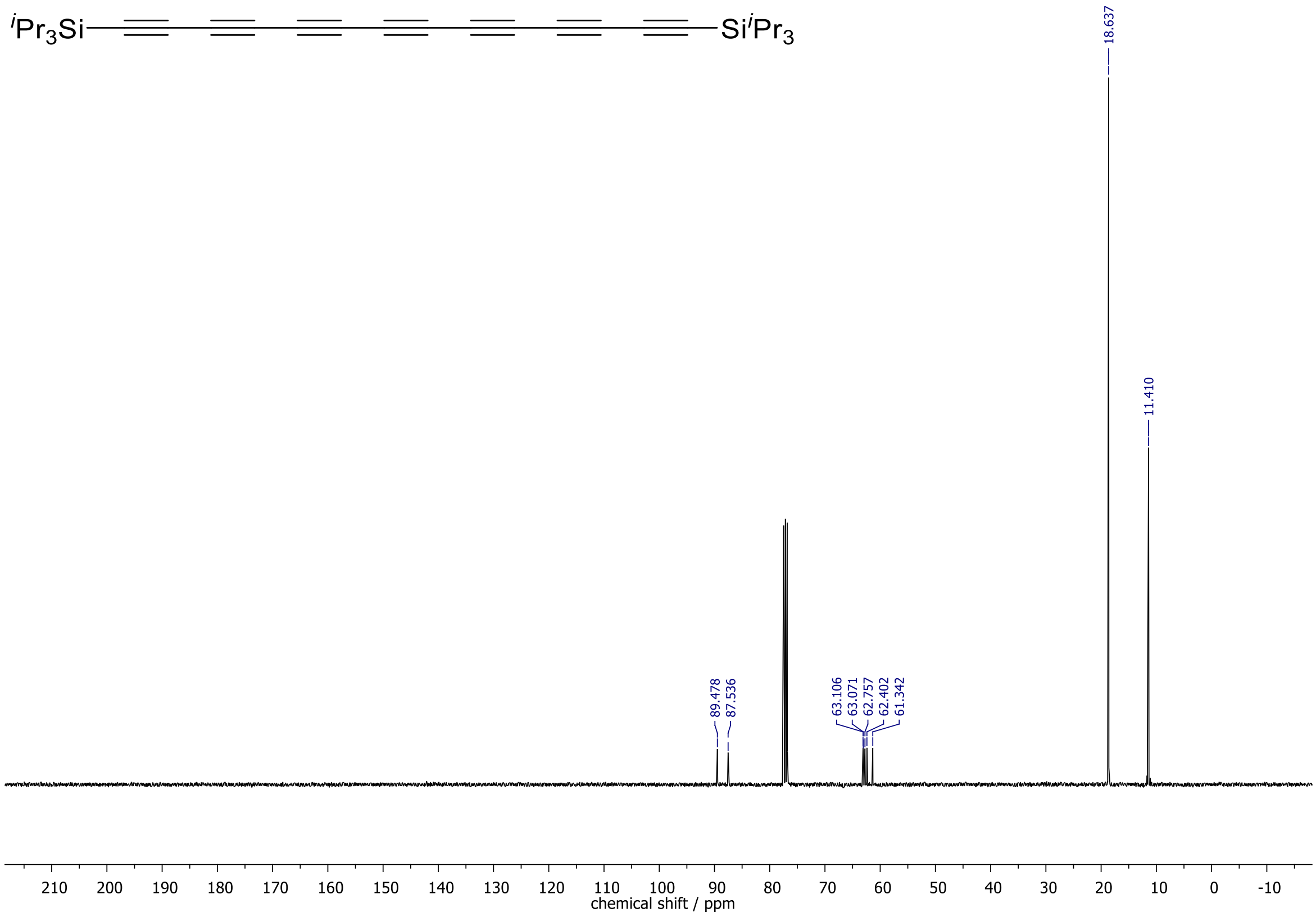

Supplementary Figure 33: $^{13}C\{^1H\}$ NMR spectrum of **1,14-bis(triisopropylsilyl)-1,3,5,7,9,11,13-tetradecaheptayne**.

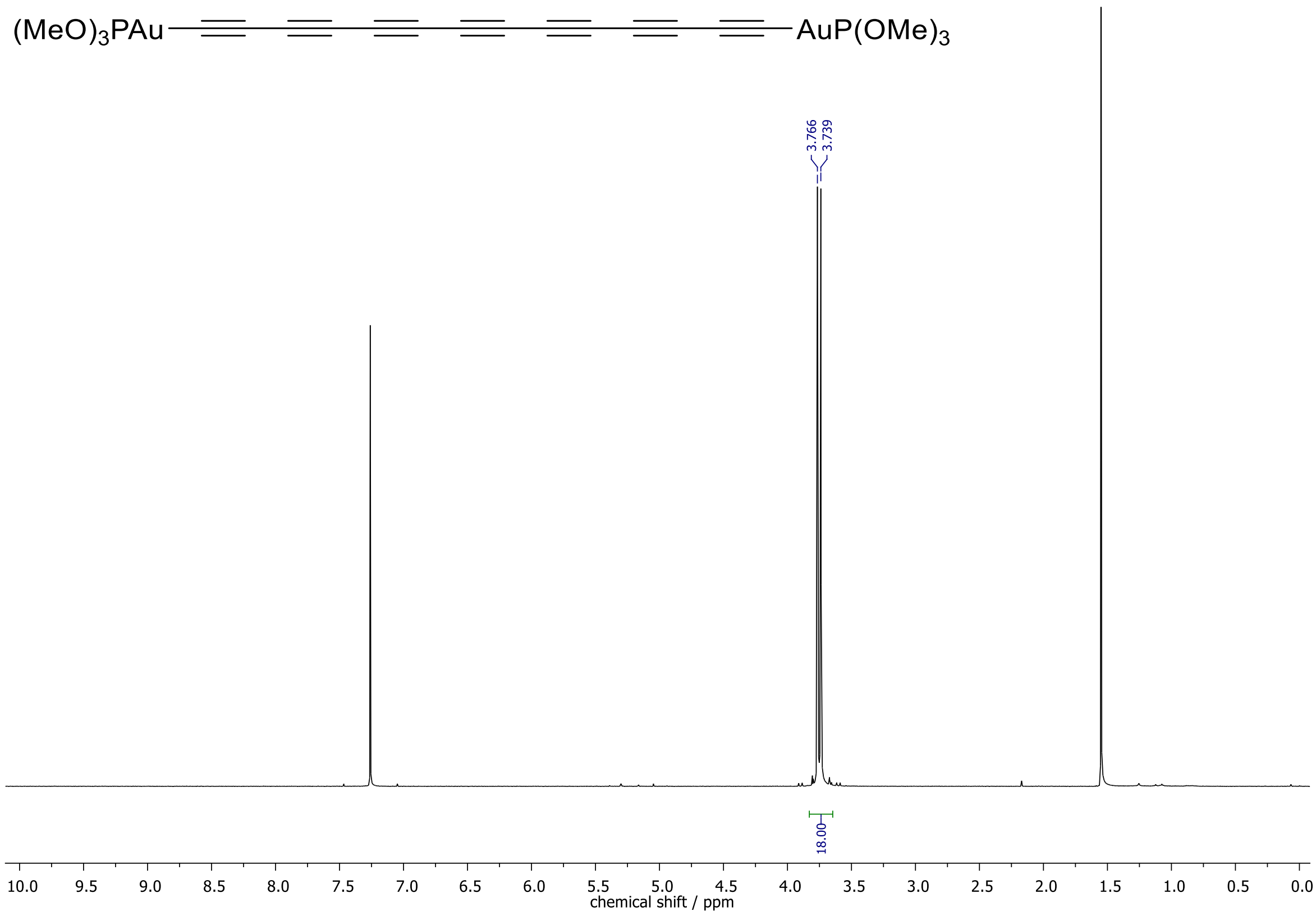

Supplementary Figure 34: ^1H NMR spectrum of **1(14)**.

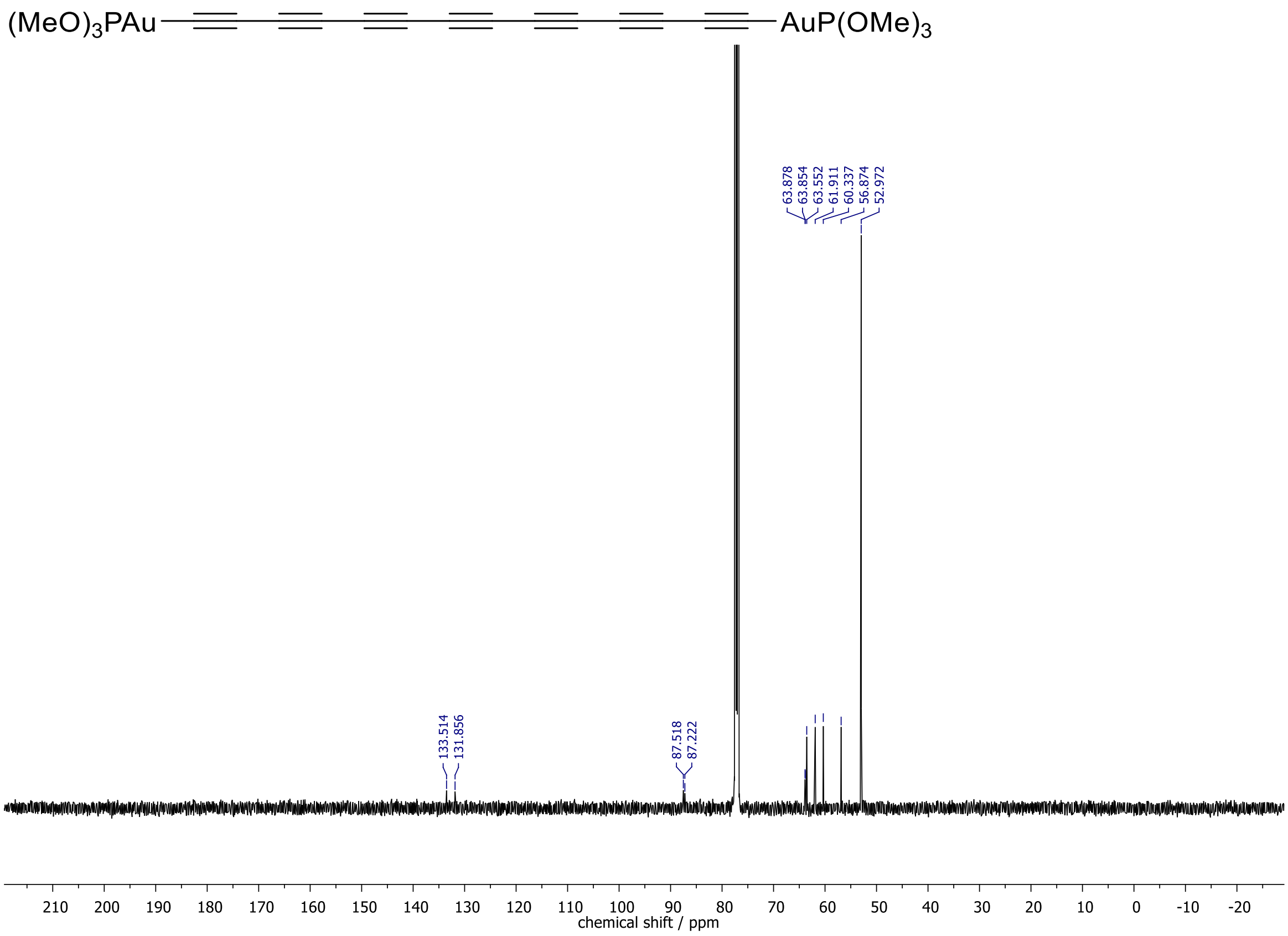

Supplementary Figure 35: $^{13}C\{^{1}H\}$ NMR spectrum of **1(14)**.

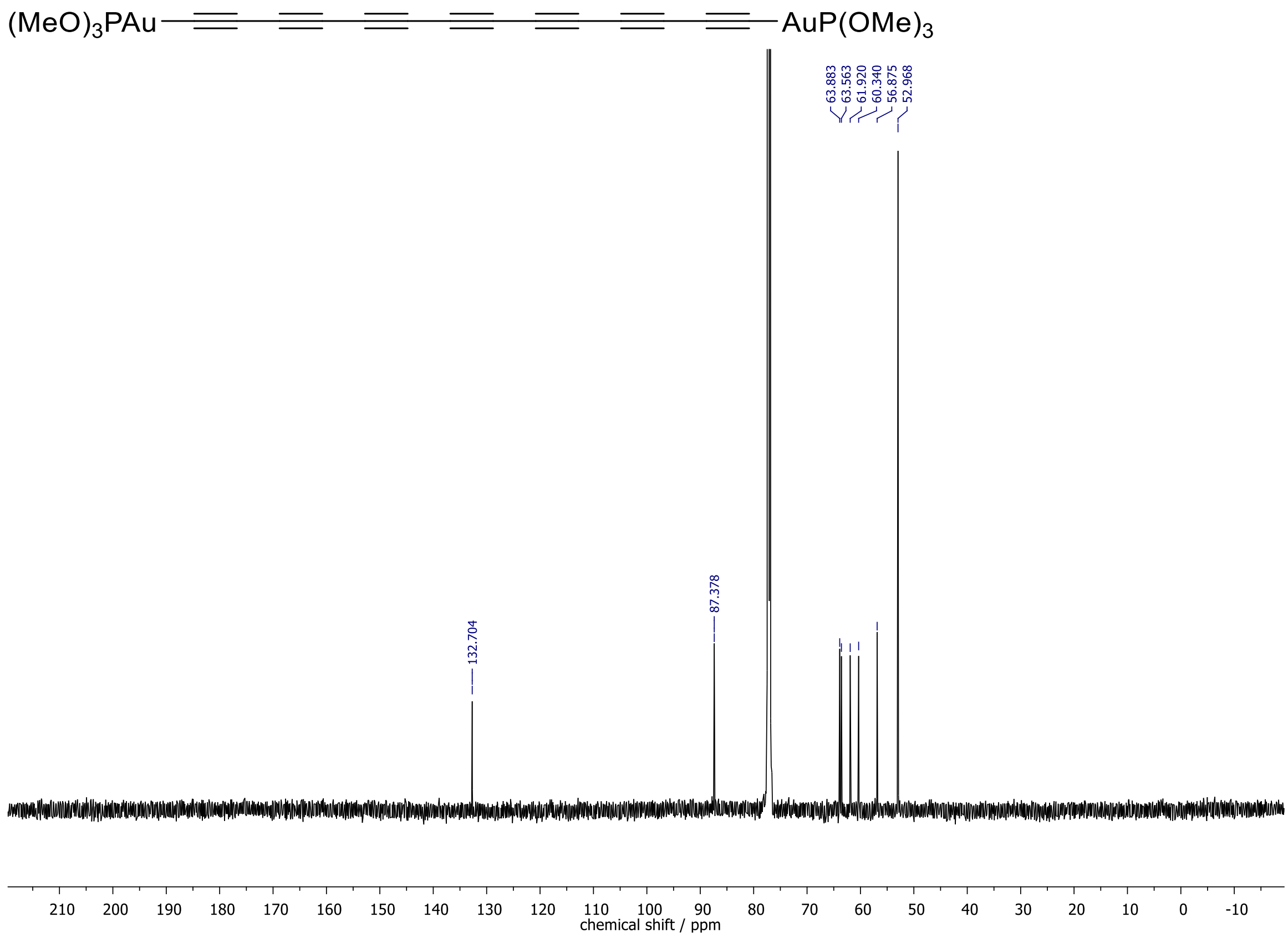

Supplementary Figure 36: $^{13}C\{^{1}H,^{31}P\}$ NMR spectrum of **1(14).**

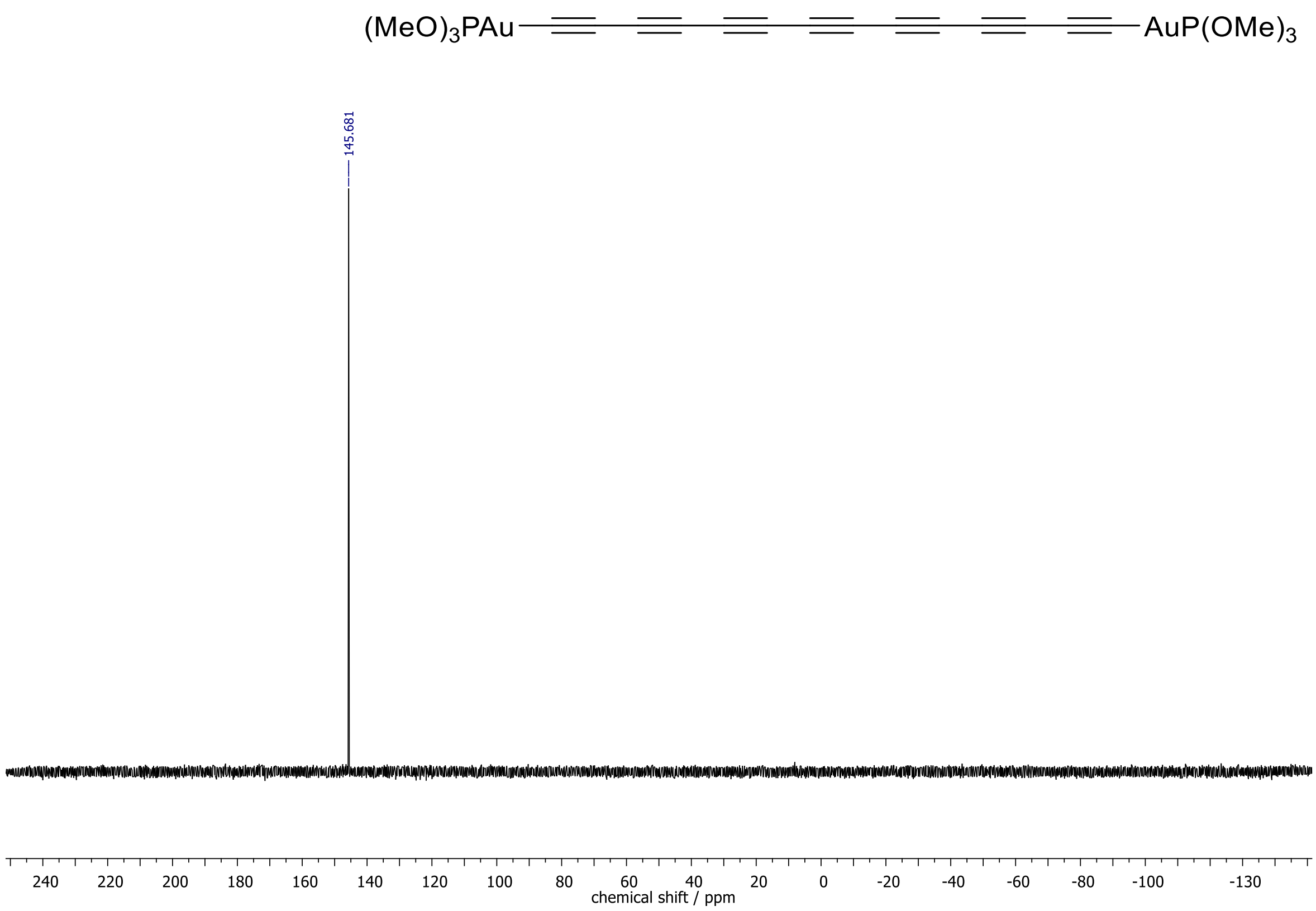

Supplementary Figure 37: $^{31}P\{^{1}H\}$ NMR spectrum of **1(14)**.

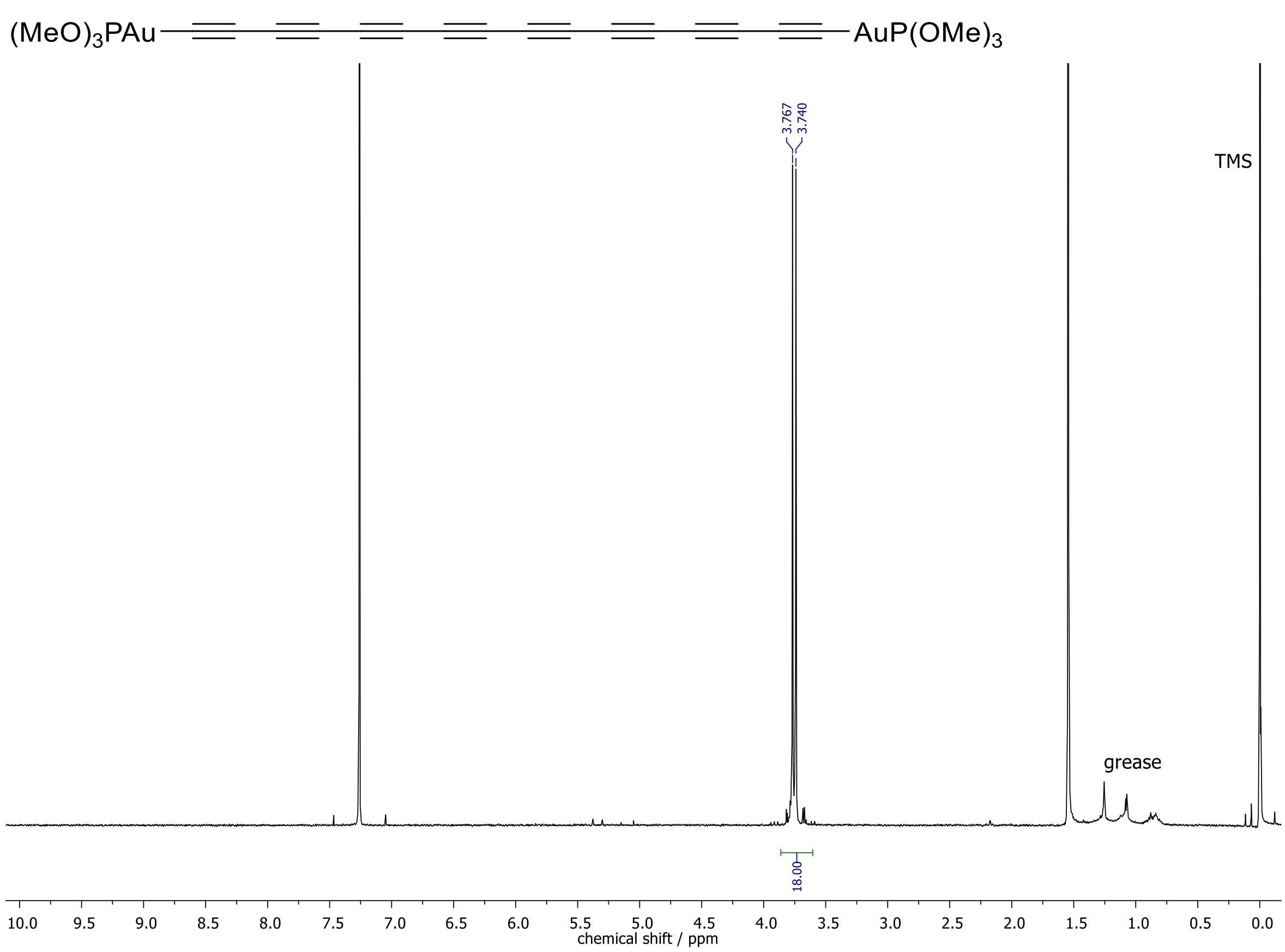

Supplementary Figure 38: ^{1}H NMR spectrum of **1(16)**.

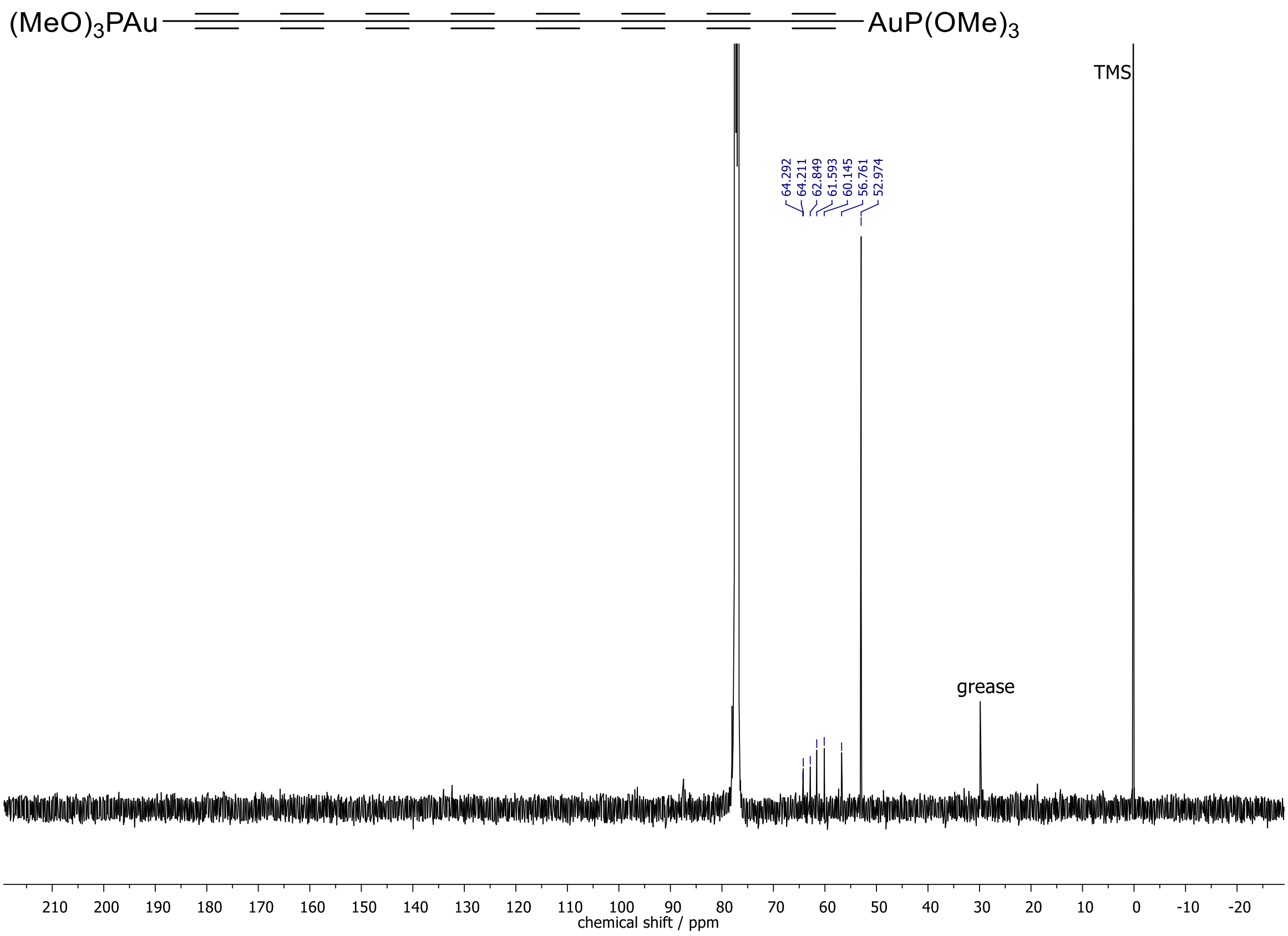

Supplementary Figure 39: $^{13}C\{^{1}H\}$ NMR spectrum of **1(16)**.

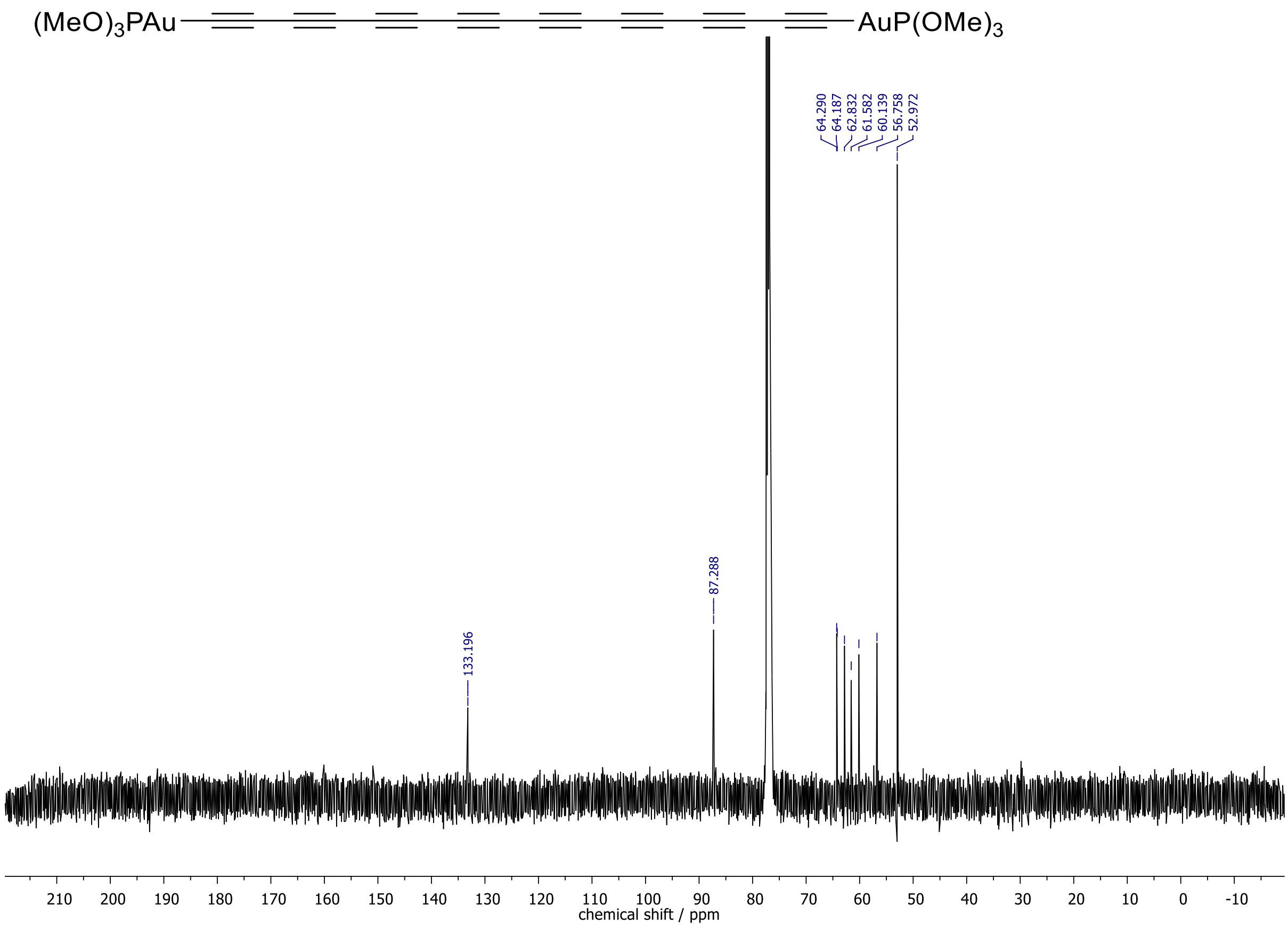

Supplementary Figure 40: $^{13}C\{^{1}H,^{31}P\}$ NMR spectrum of **1(16).**

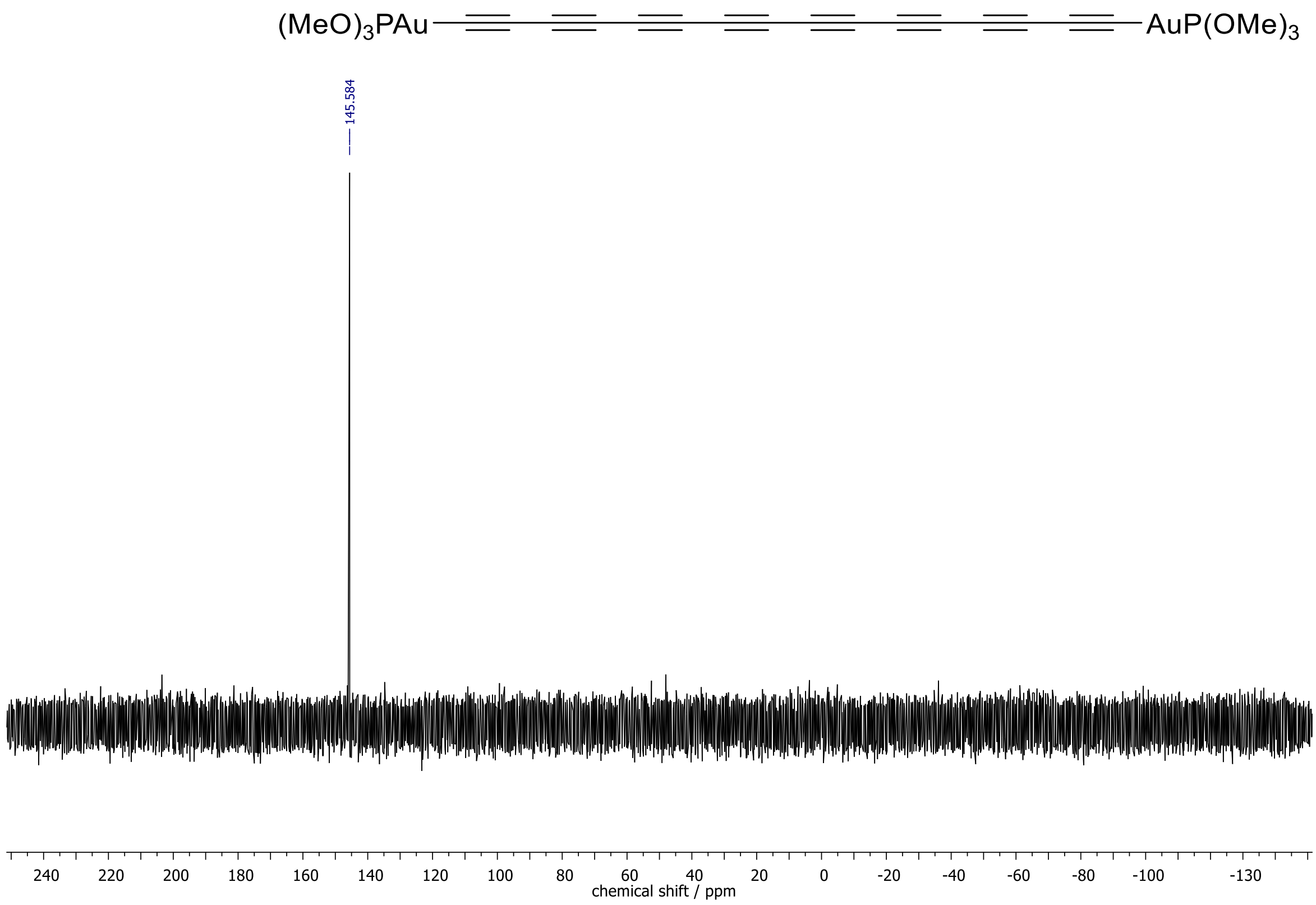

Supplementary Figure 41: $^{31}P\{^{1}H\}$ NMR spectrum of **1(16)**.

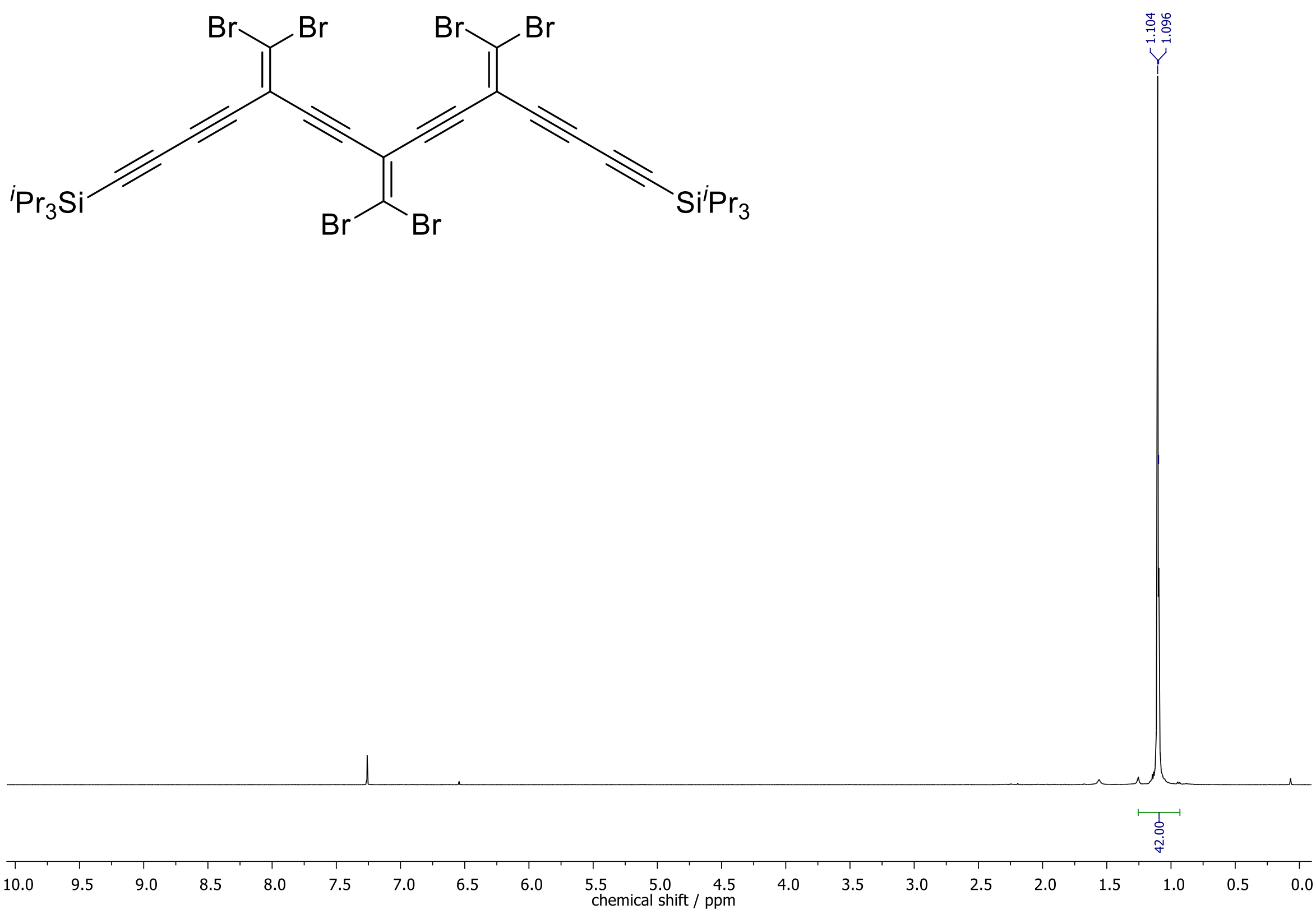

Supplementary Figure 42: ^{1}H NMR spectrum of **S4**.

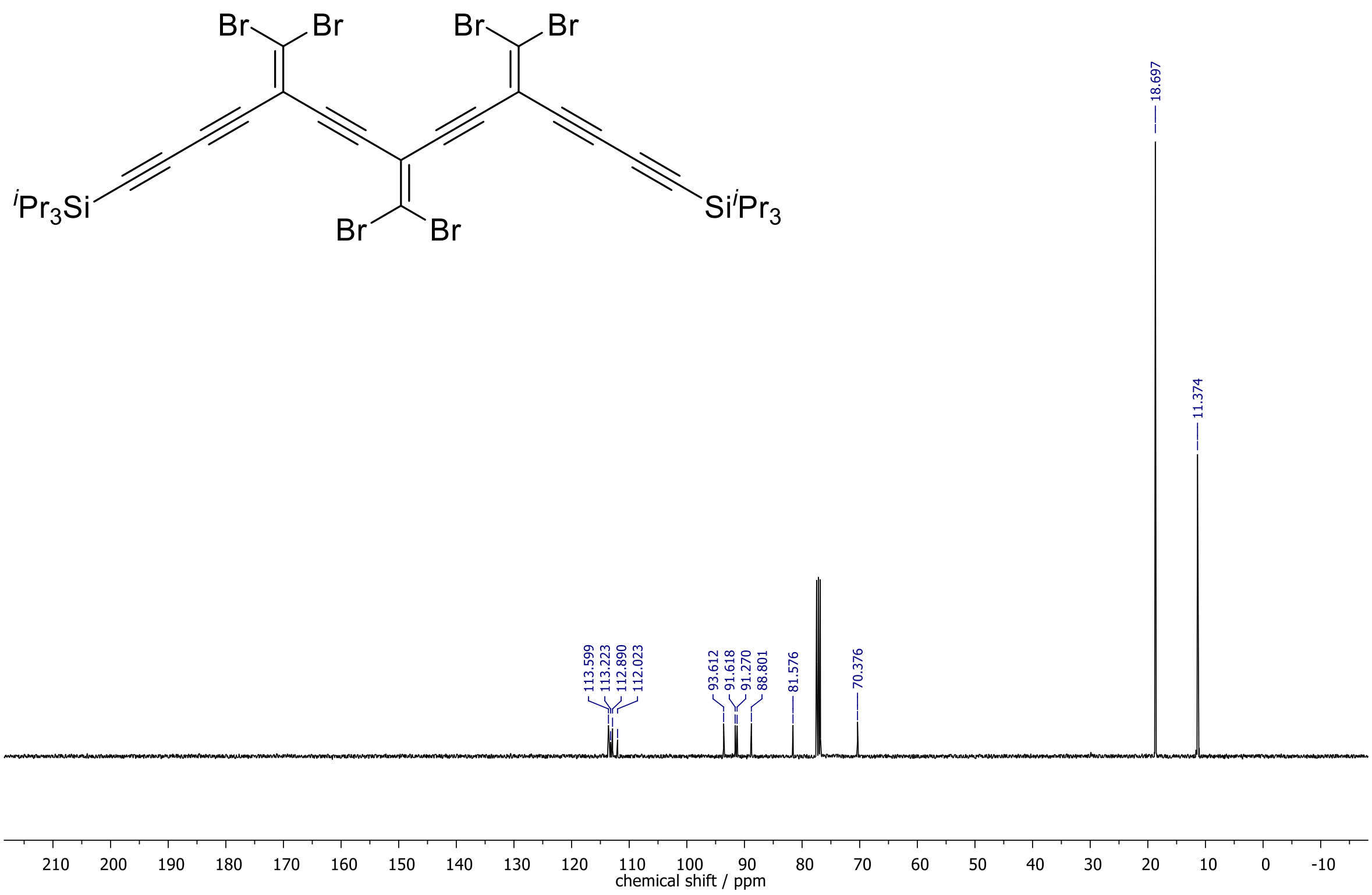

Supplementary Figure 43: $^{13}C\{^{1}H\}$ NMR spectrum of **S4**.

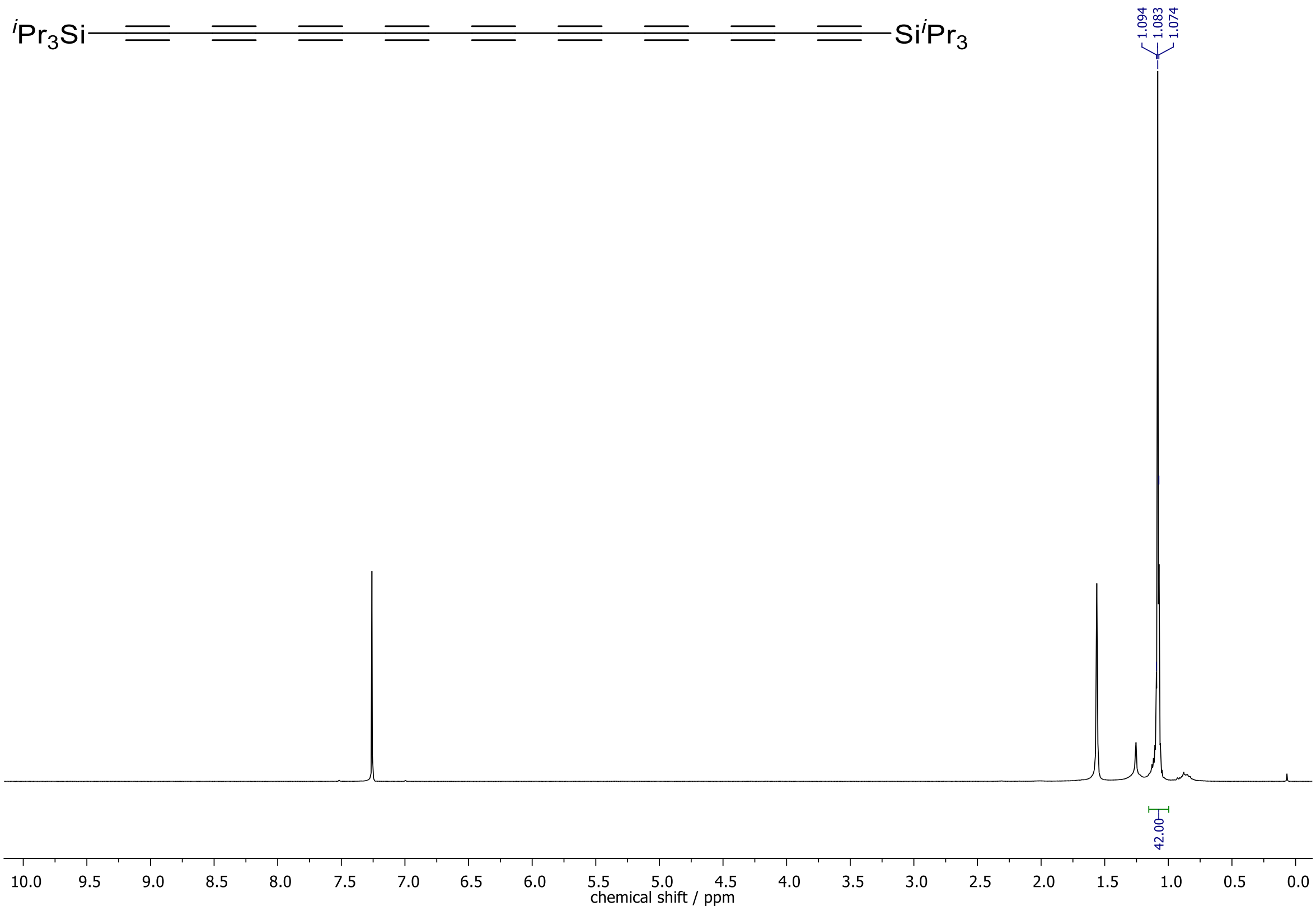

Supplementary Figure 44: ^{1}H NMR spectrum of **1,18-bis(triisopropylsilyl)-1,3,5,7,9,11,13,15,17-octadecanonayne**.

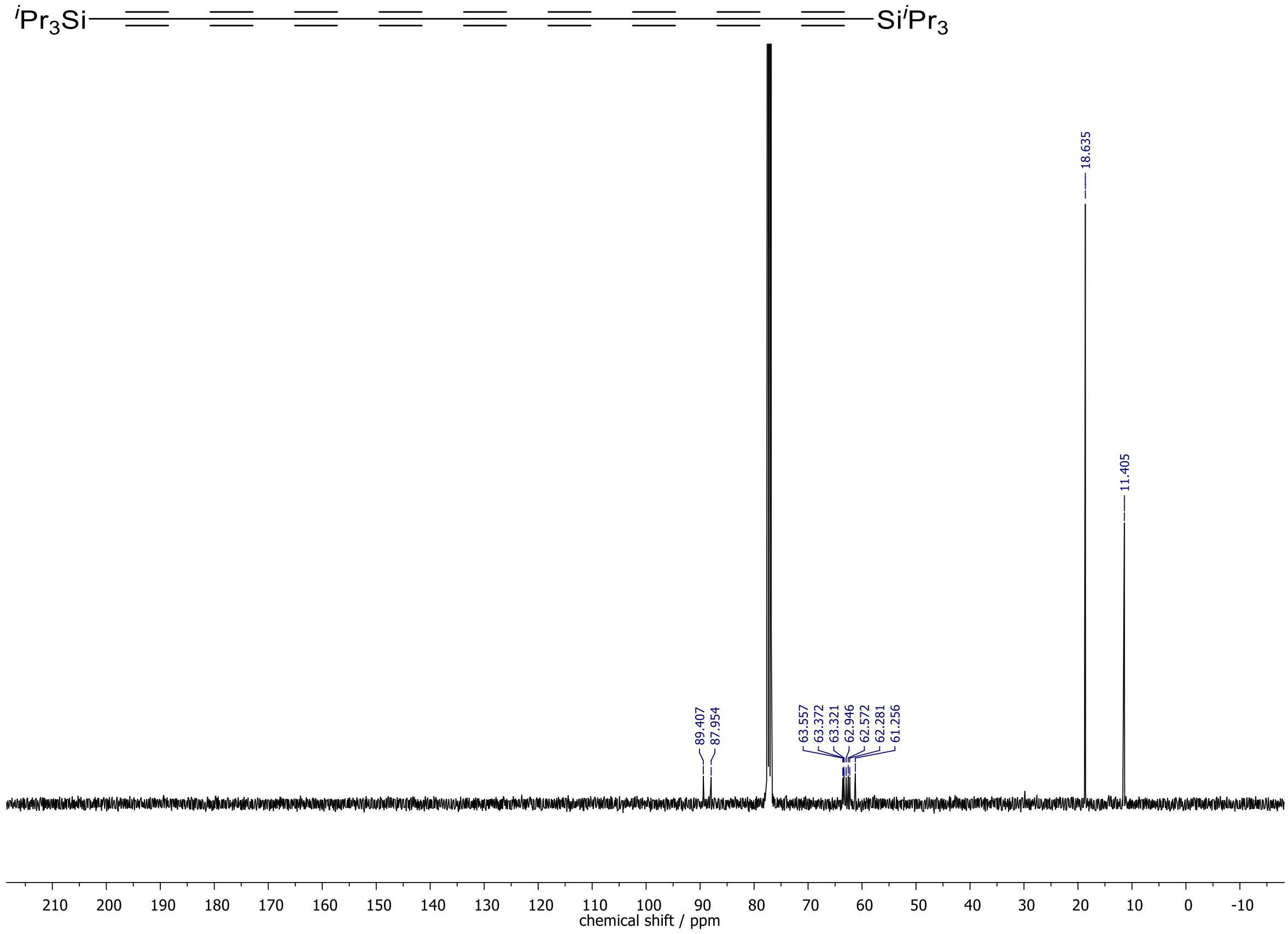

Supplementary Figure 45: $^{13}C\{^{1}H\}$ NMR spectrum of **1,18-bis(triisopropylsilyl)-1,3,5,7,9,11,13,15,17-octadecanonayne**.

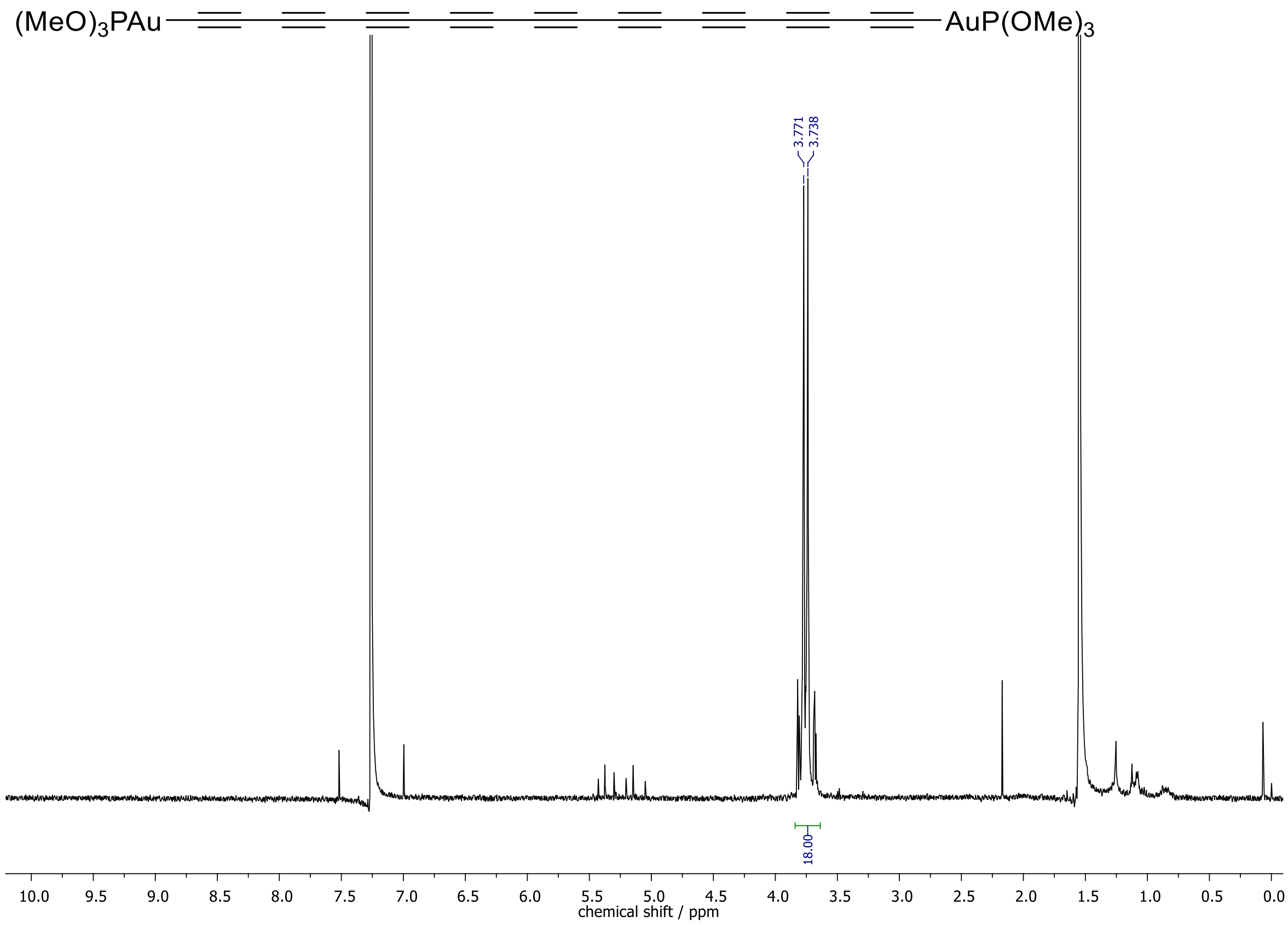

Supplementary Figure 46: ^{1}H NMR spectrum of crude **1(18)**.

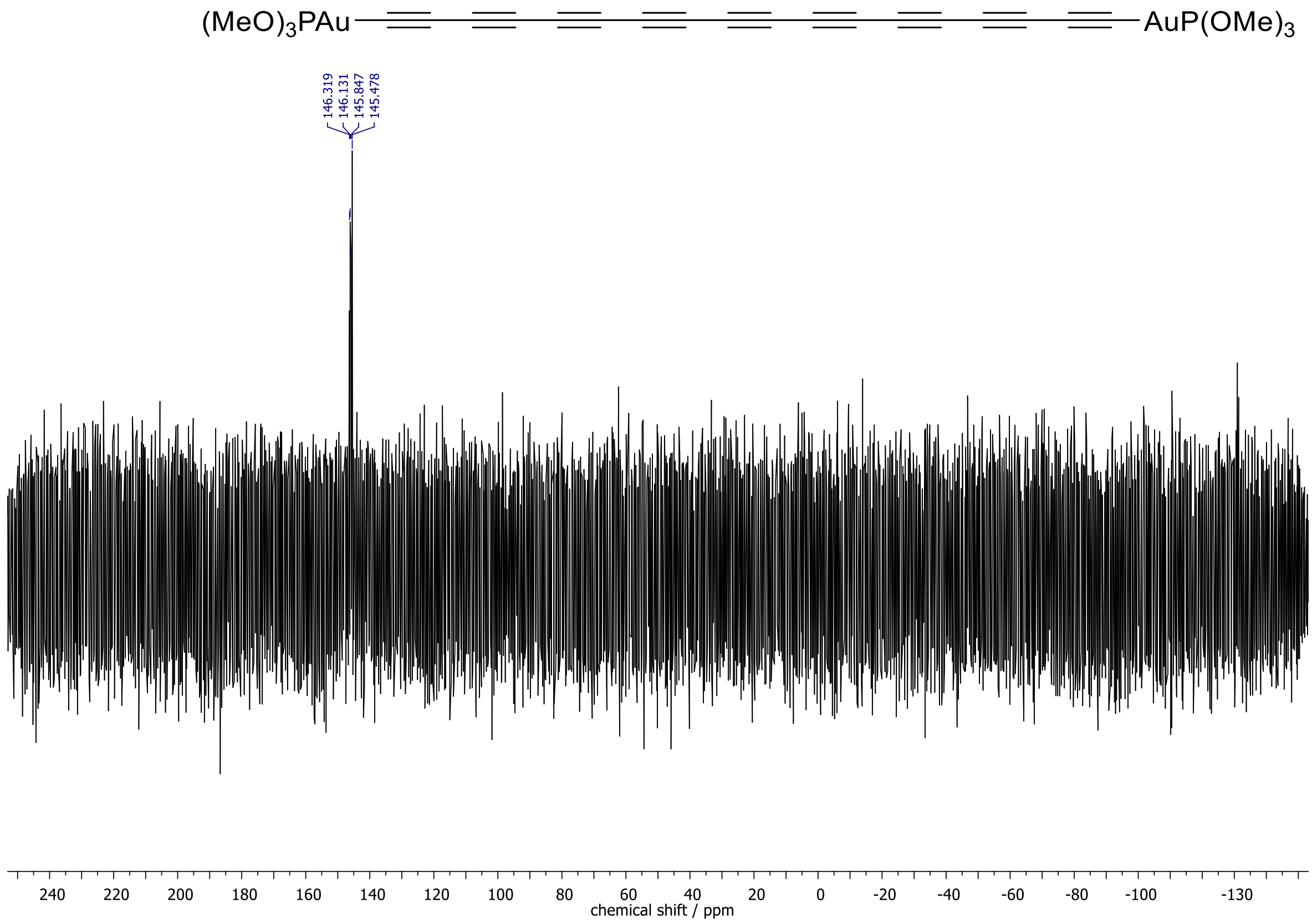

Supplementary Figure 47: $^{31}P\{^{1}H\}$ NMR spectrum of crude **1(18)**.

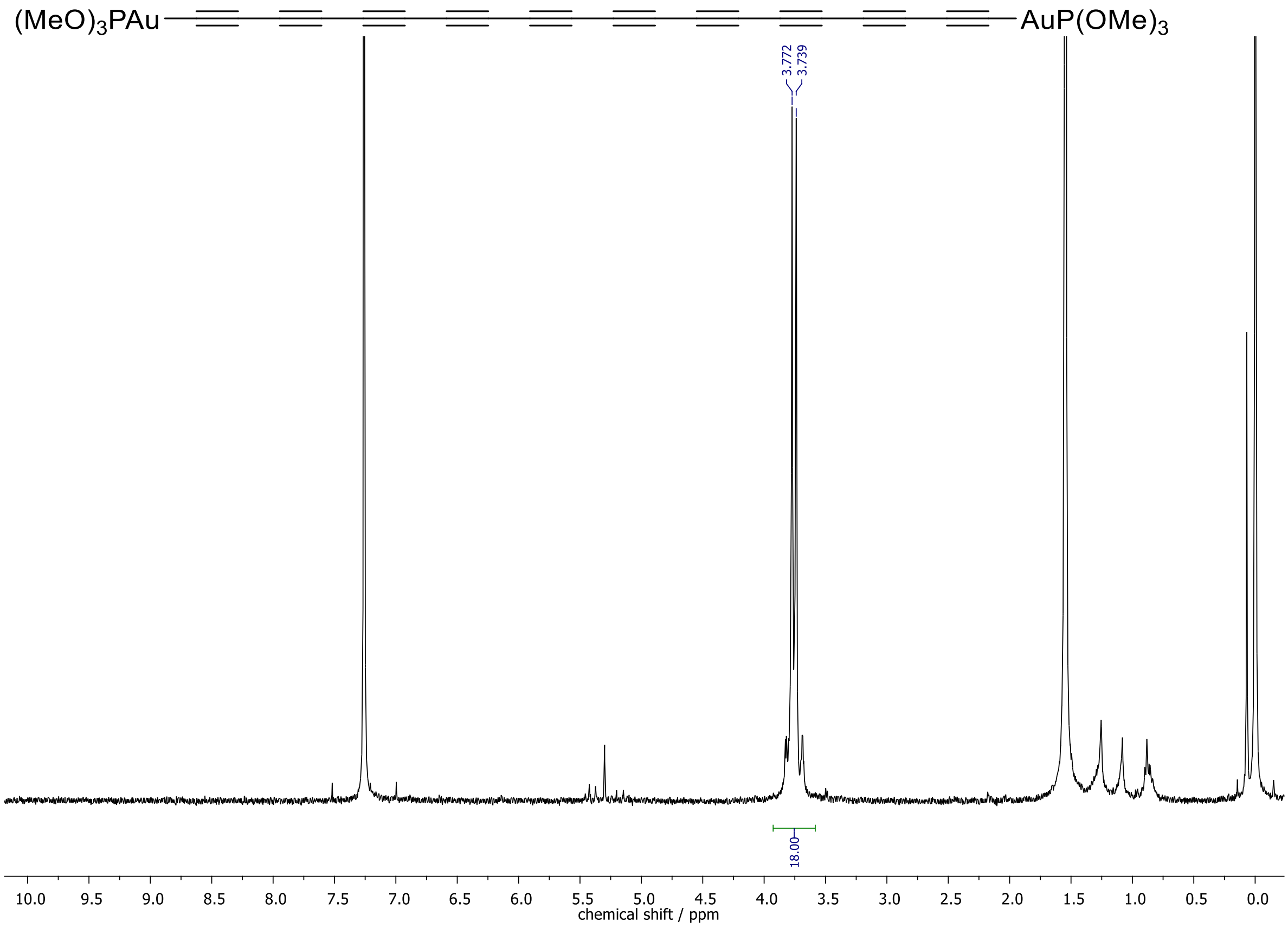

Supplementary Figure 48: ^{1}H NMR spectrum of crude **1(20)**.

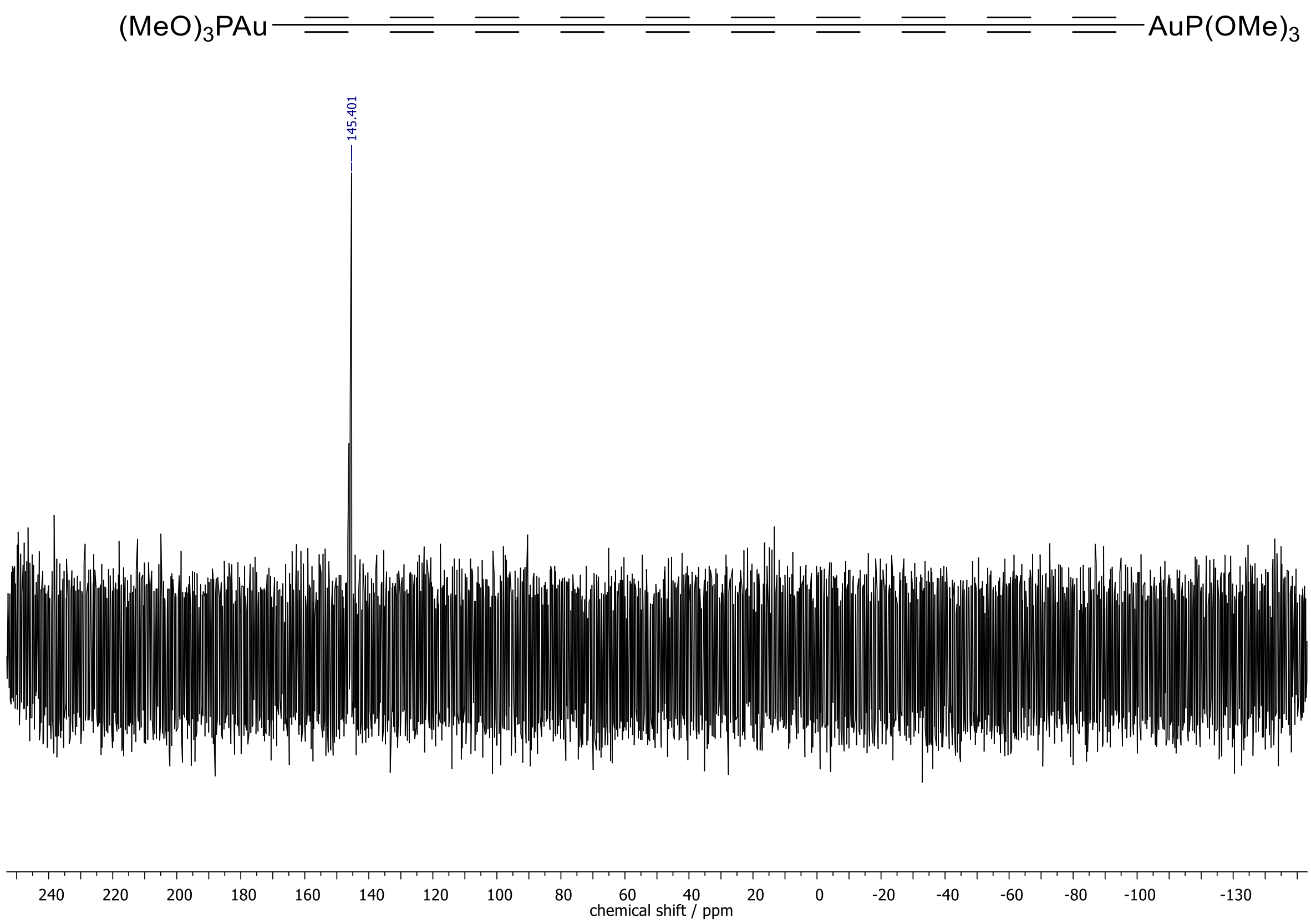

Supplementary Figure 49: $^{13}C\{^1H\}$ NMR spectrum of crude **1(20)**.

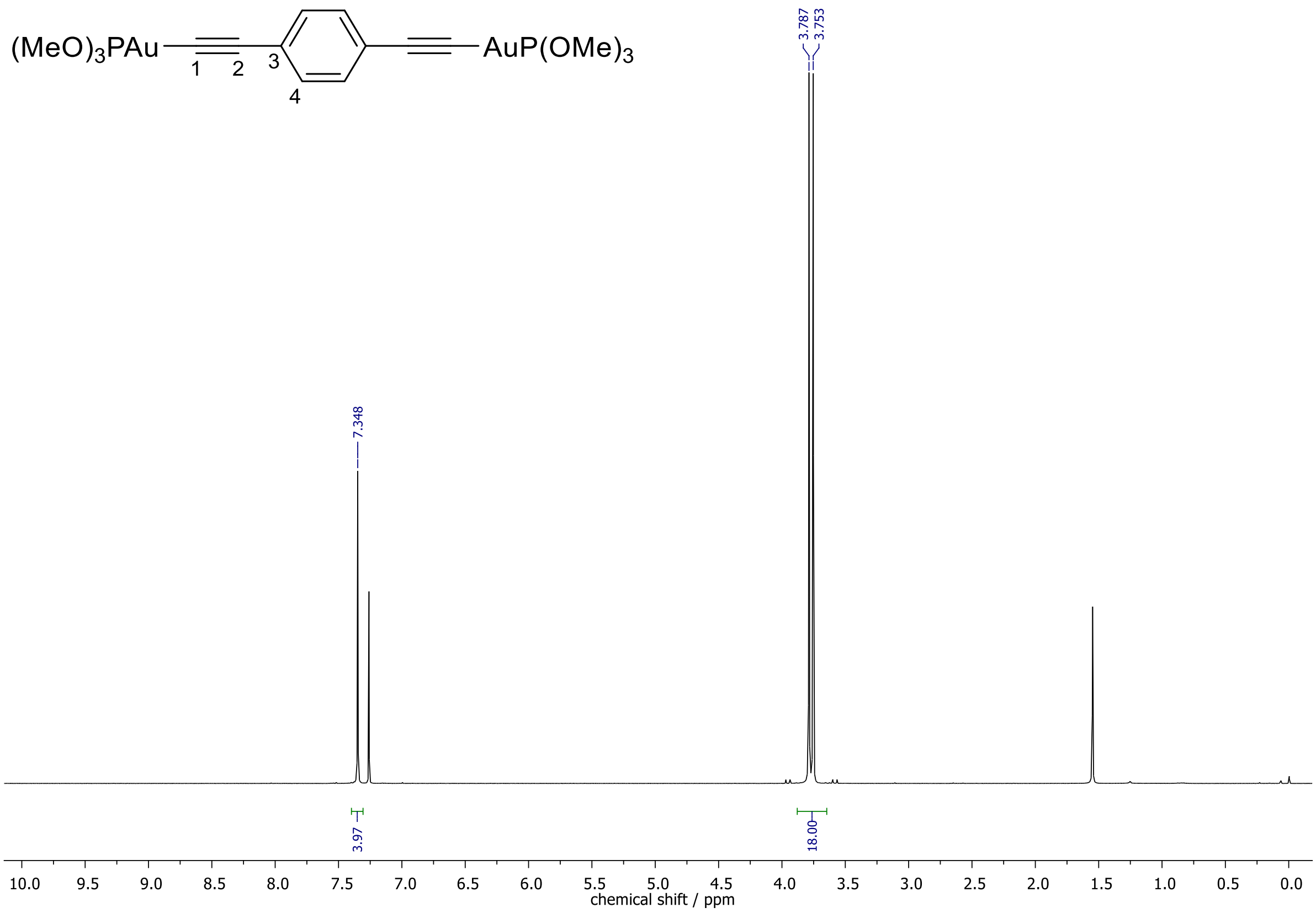

Supplementary Figure 50: ^1H NMR spectrum of **A1**.

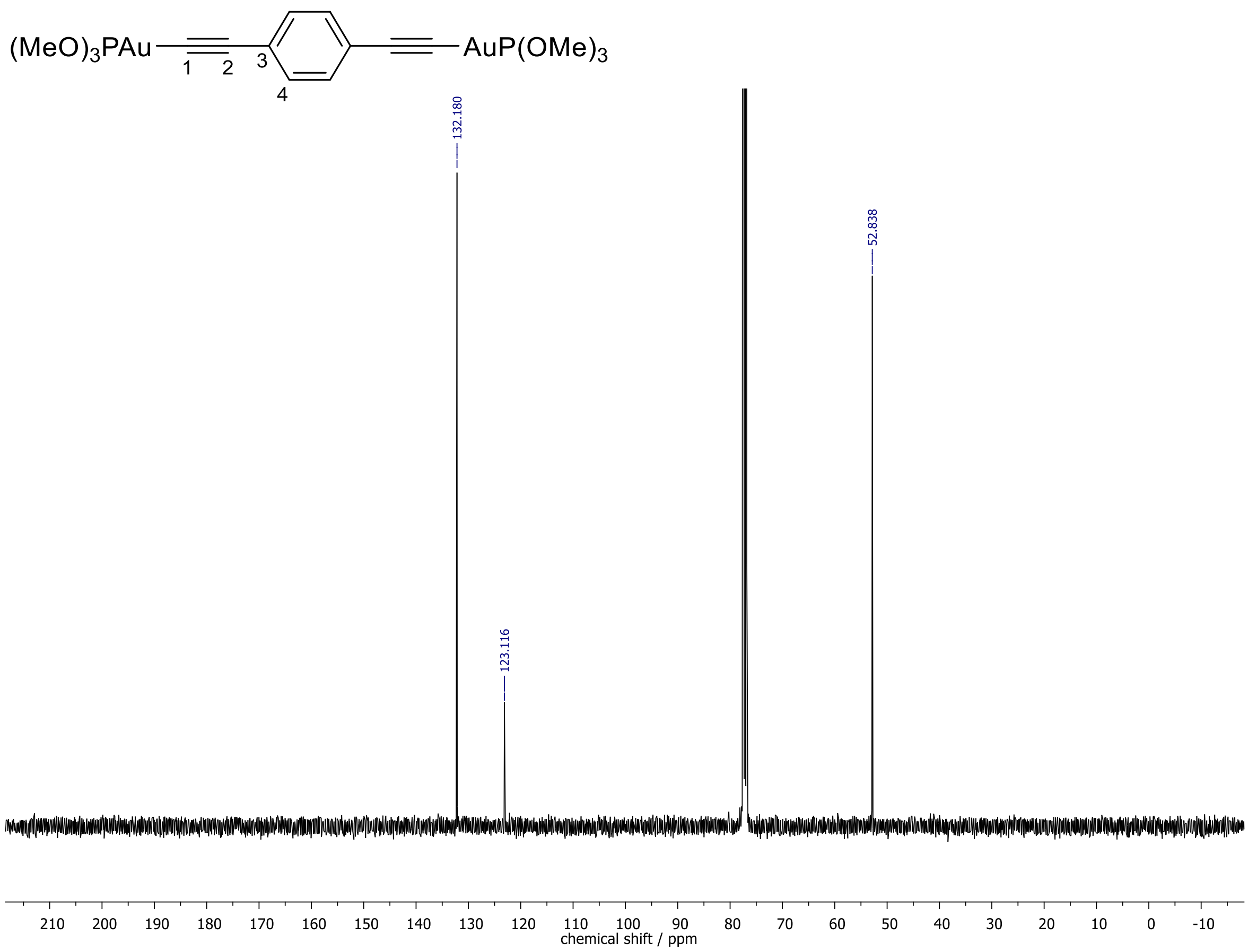

Supplementary Figure 51: $^{13}C\{^{1}H\}$ NMR spectrum of **A1**.

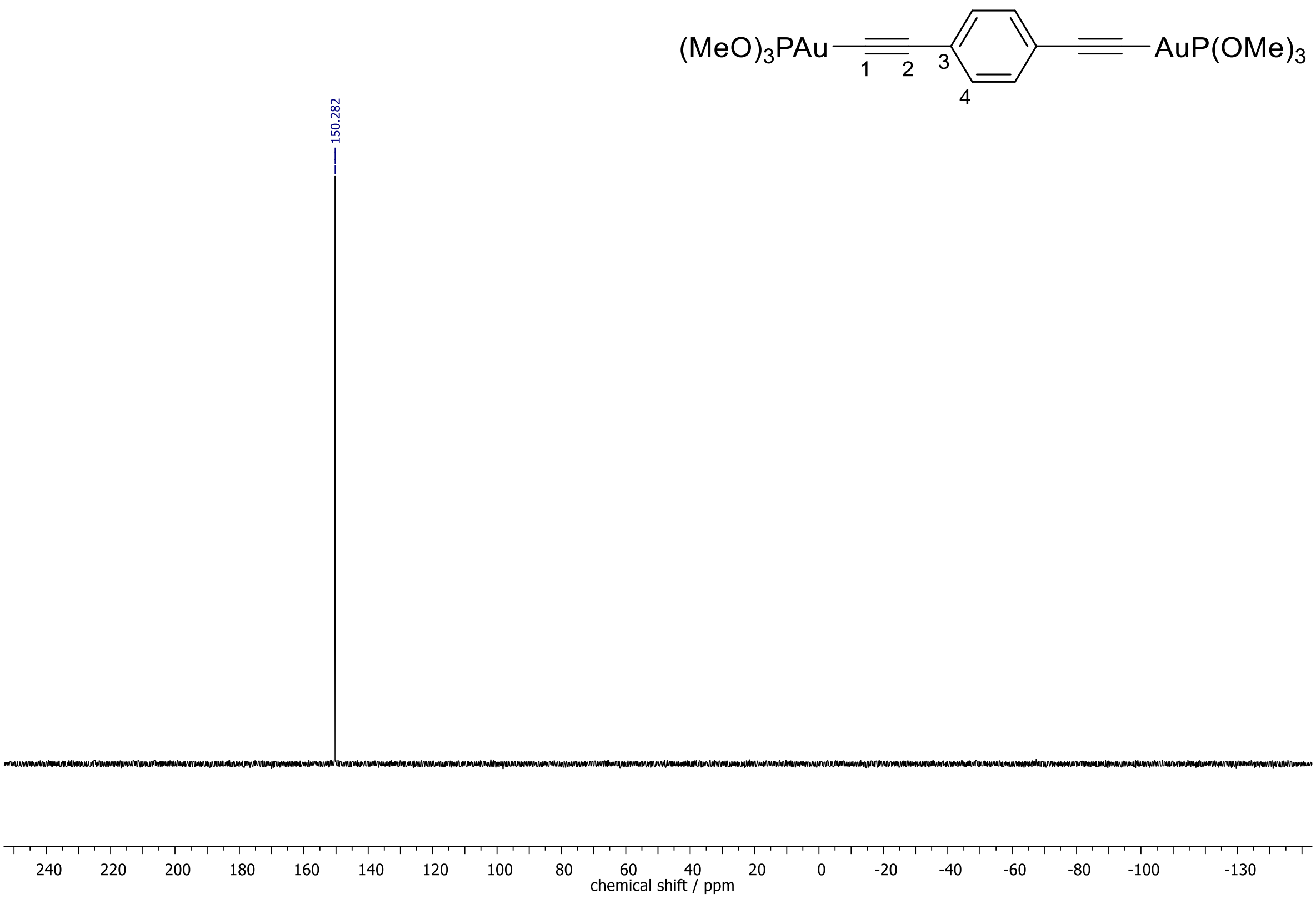

Supplementary Figure 52: $^{31}P\{^{1}H\}$ NMR spectrum of **A1**.

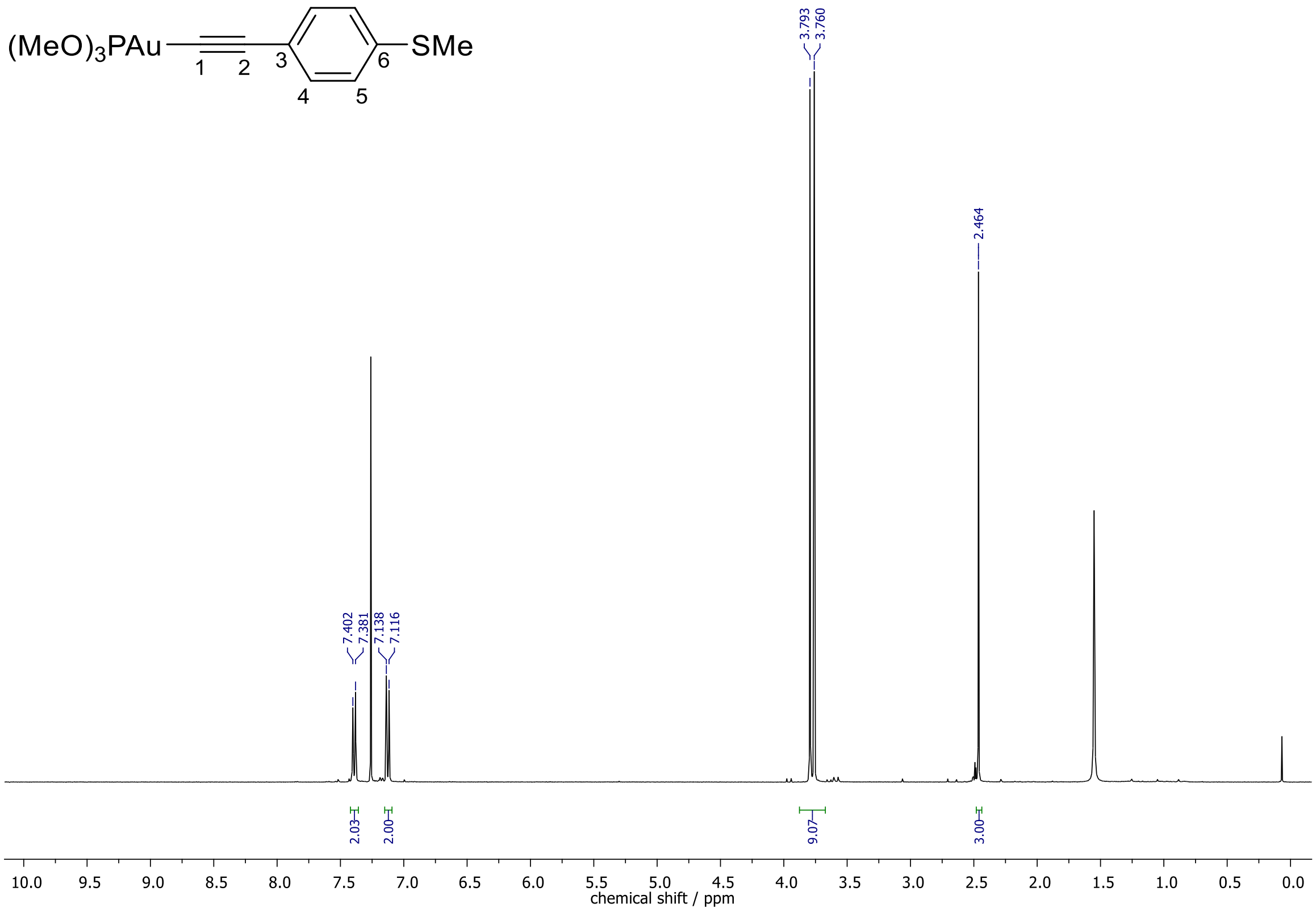

Supplementary Figure 53: ^{1}H NMR spectrum of **A2**.

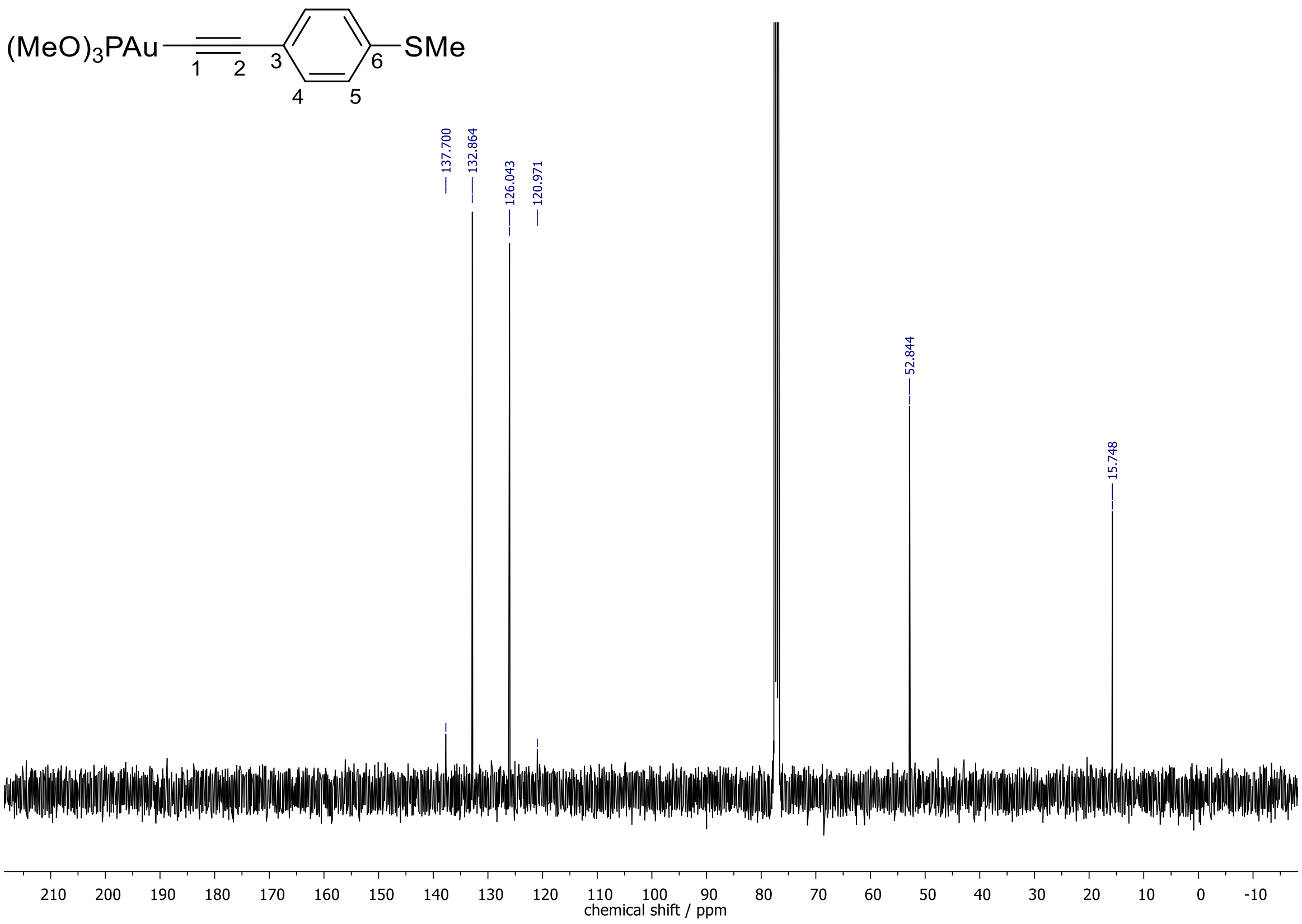

Supplementary Figure 54: $^{13}C\{^{1}H\}$ NMR spectrum of **A2**.

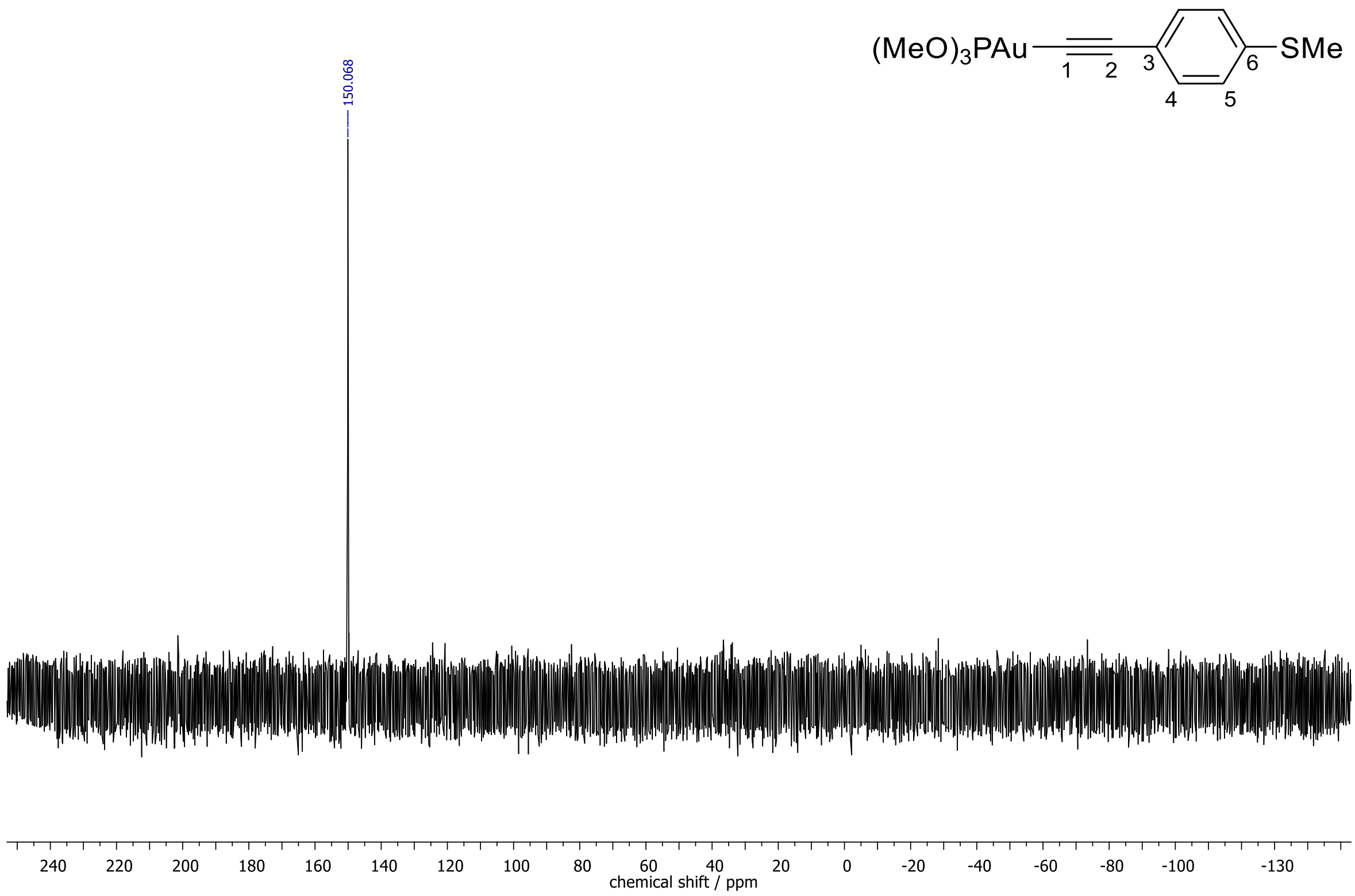

Supplementary Figure 55: $^{31}P\{^{1}H\}$ NMR spectrum of **A2**.

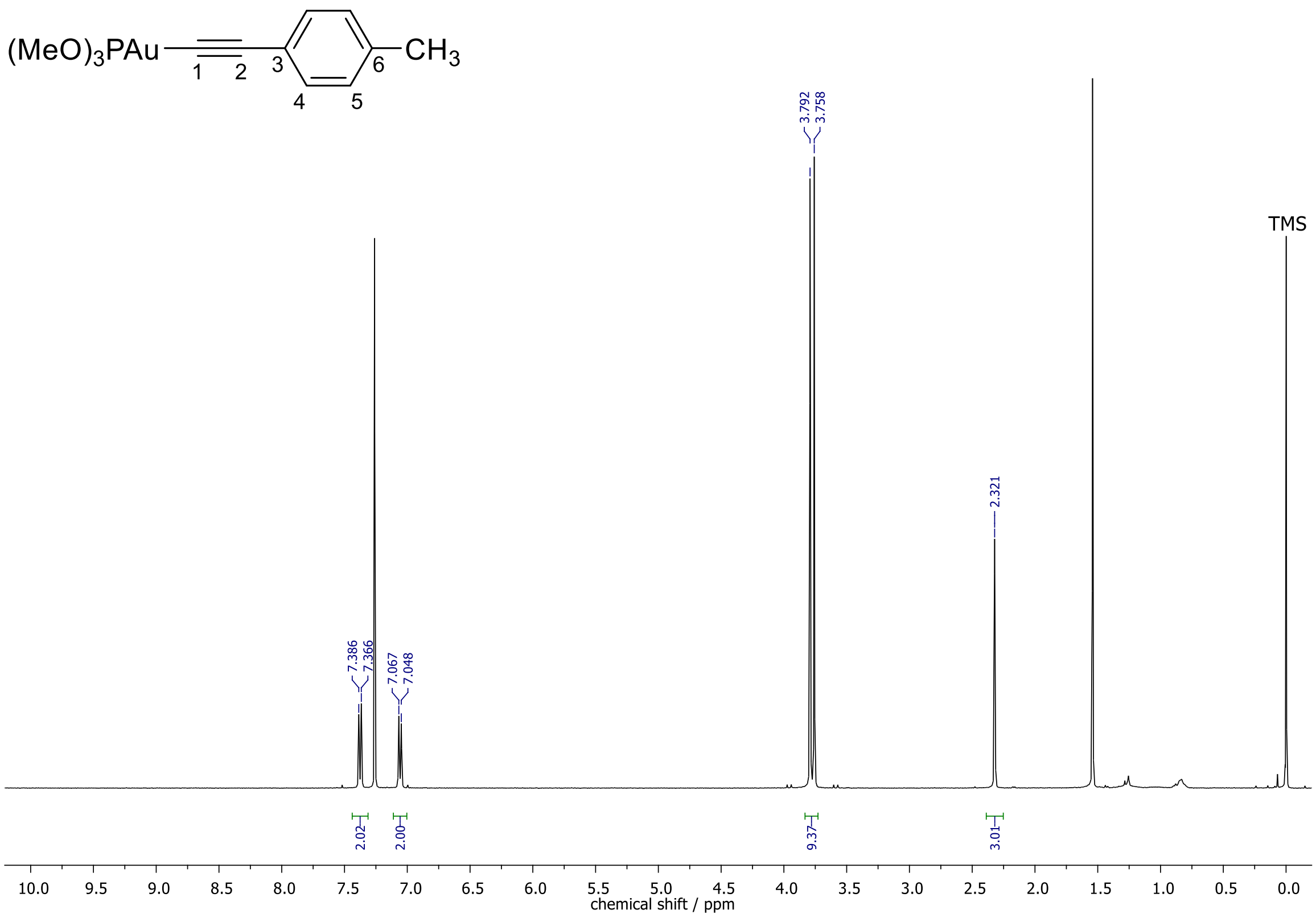

Supplementary Figure 56: ^{1}H NMR spectrum of **A3**.

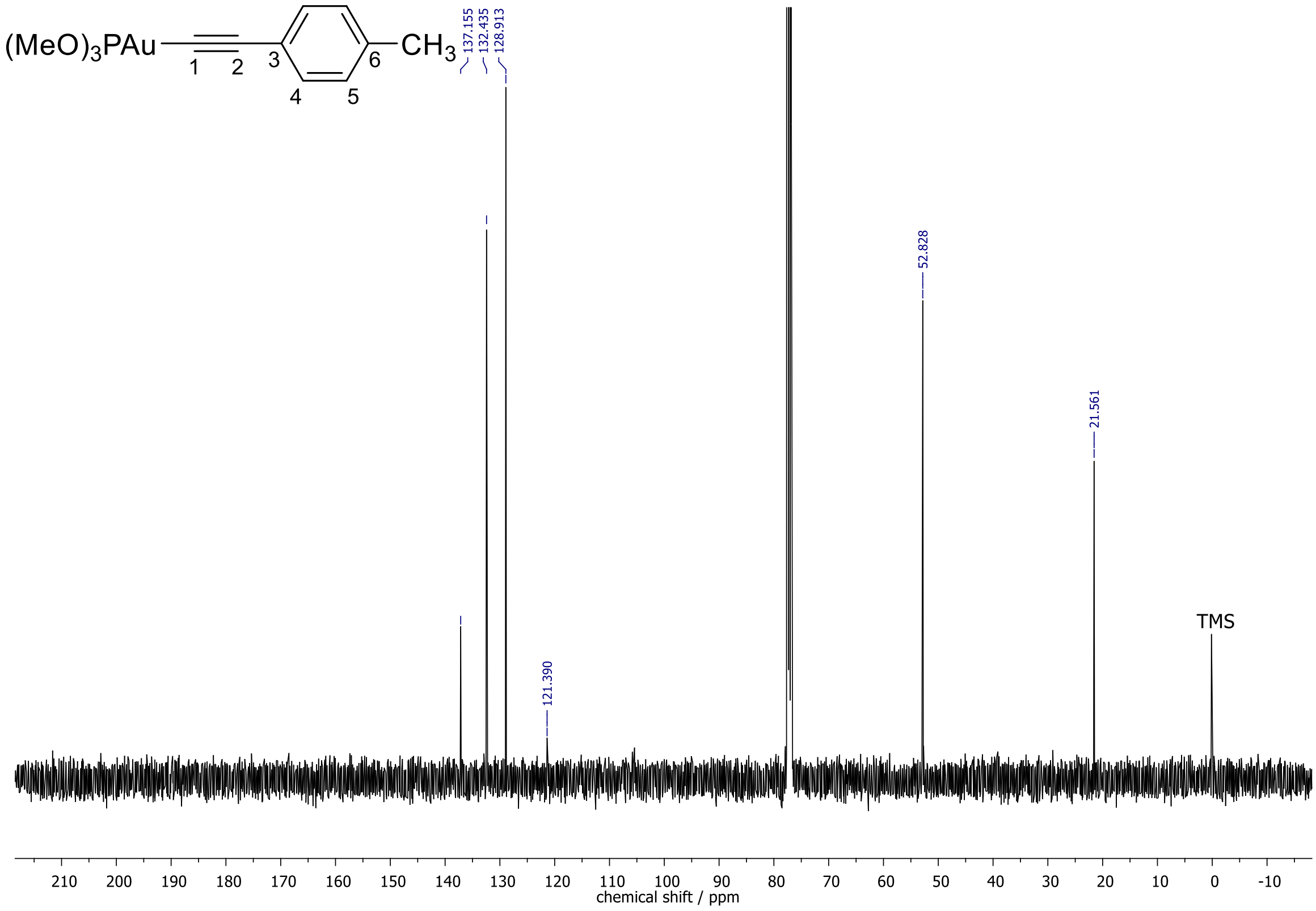

Supplementary Figure 57: $^{13}C\{^{1}H\}$ NMR spectrum of **A3**.

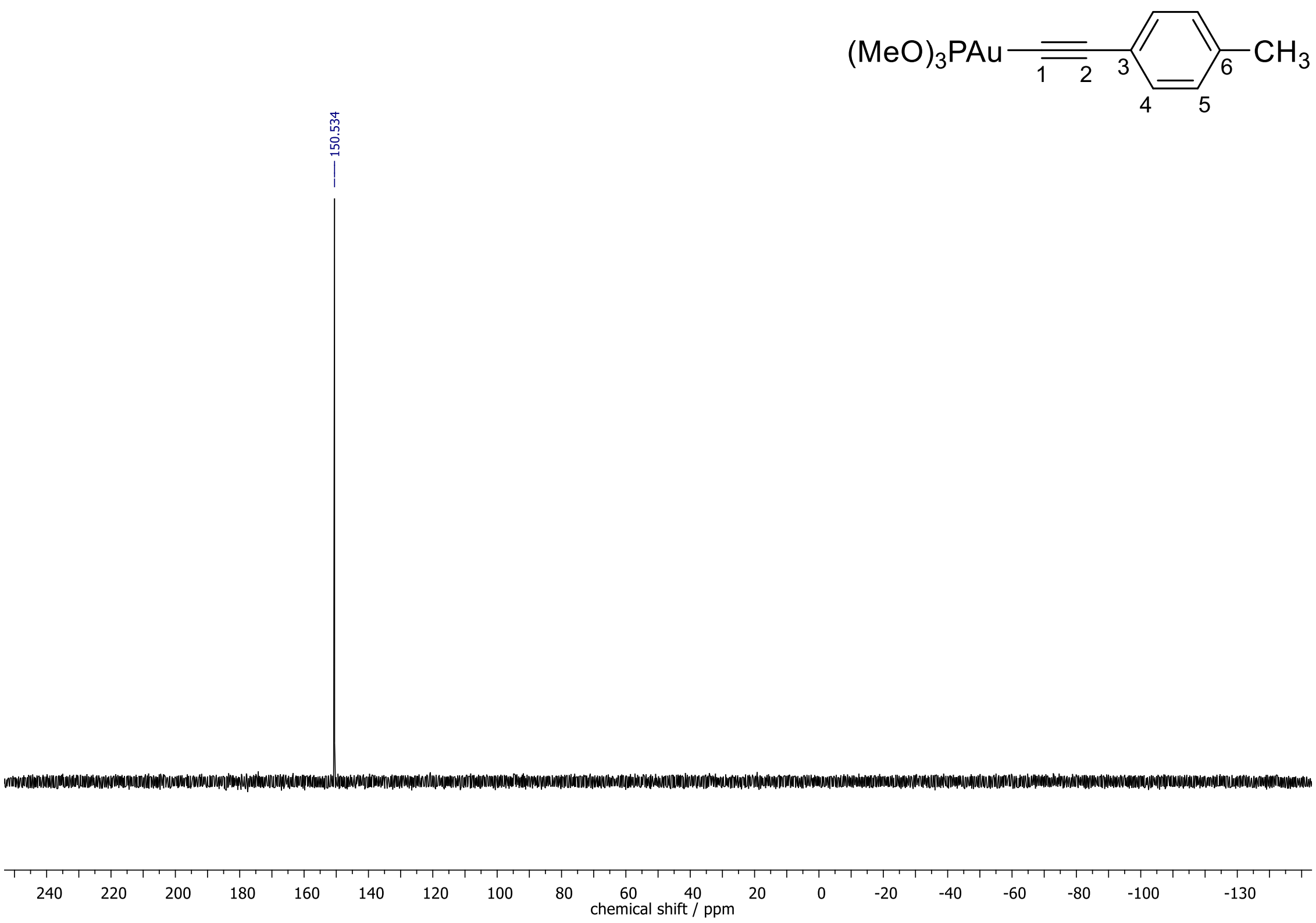

Supplementary Figure 58: $^{31}P\{^{1}H\}$ NMR spectrum of **A3**.

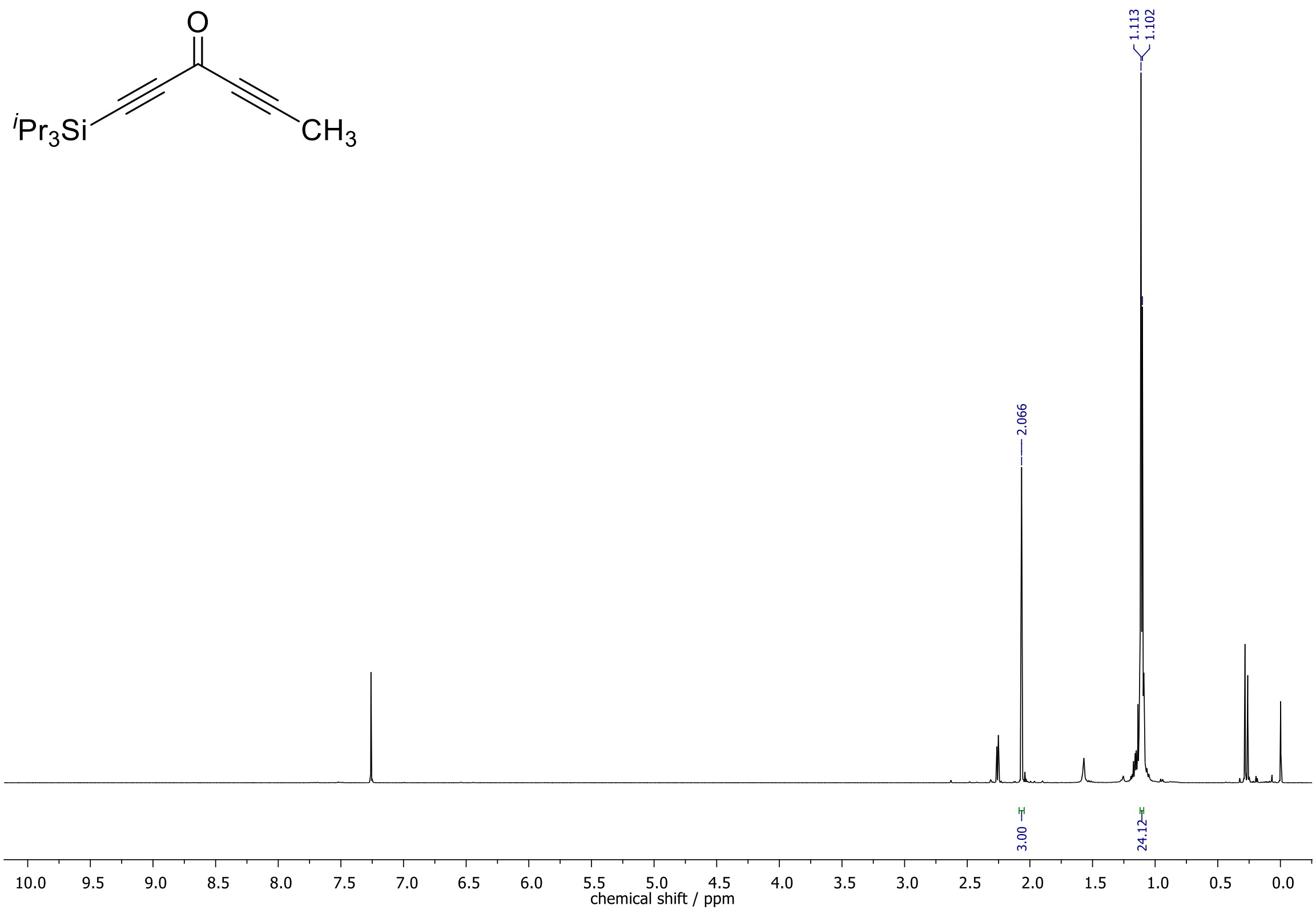

Supplementary Figure 59: ^{1}H NMR spectrum of **S5**.

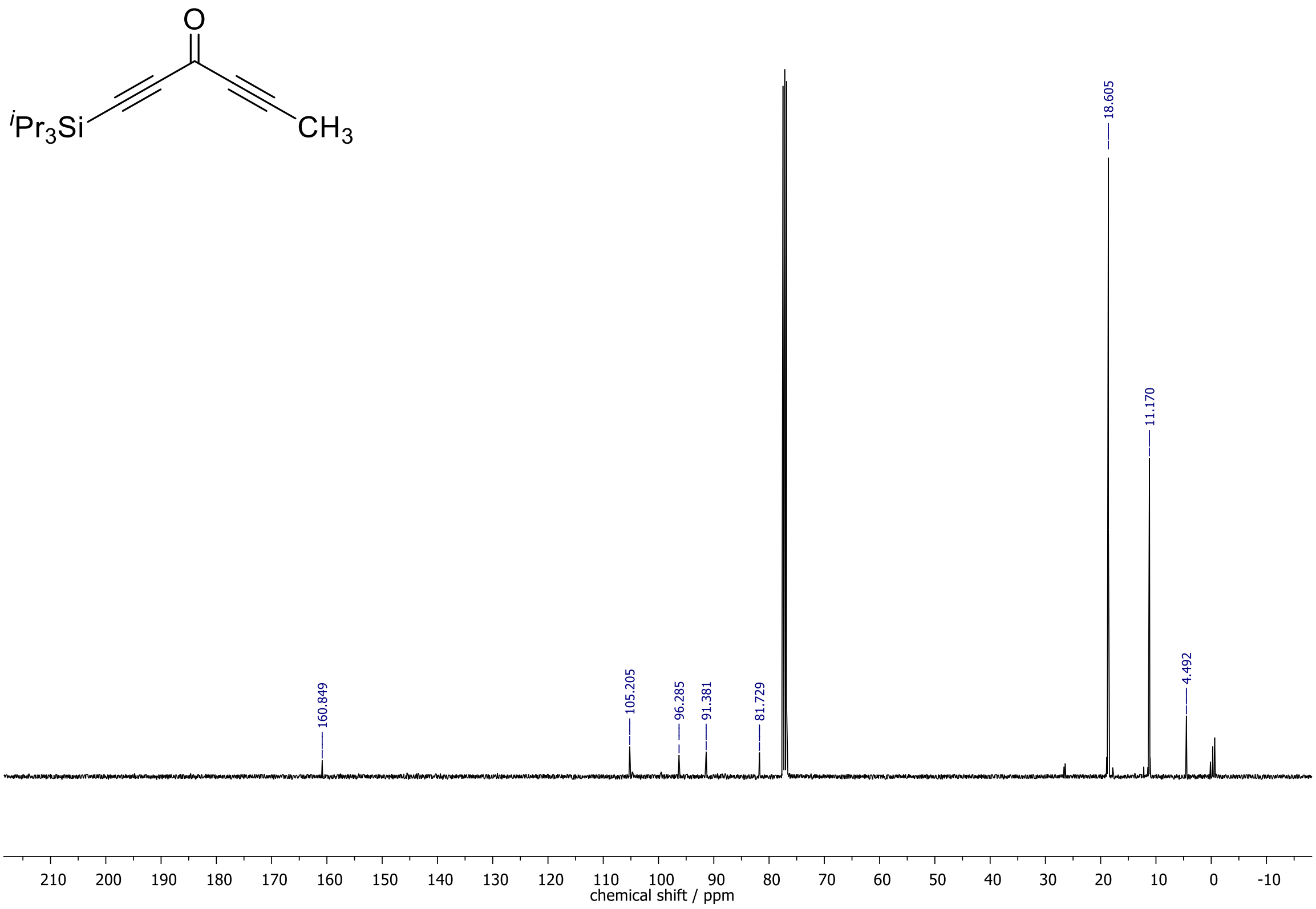

Supplementary Figure 60: $^{13}C\{^{1}H\}$ NMR spectrum of **S5**.

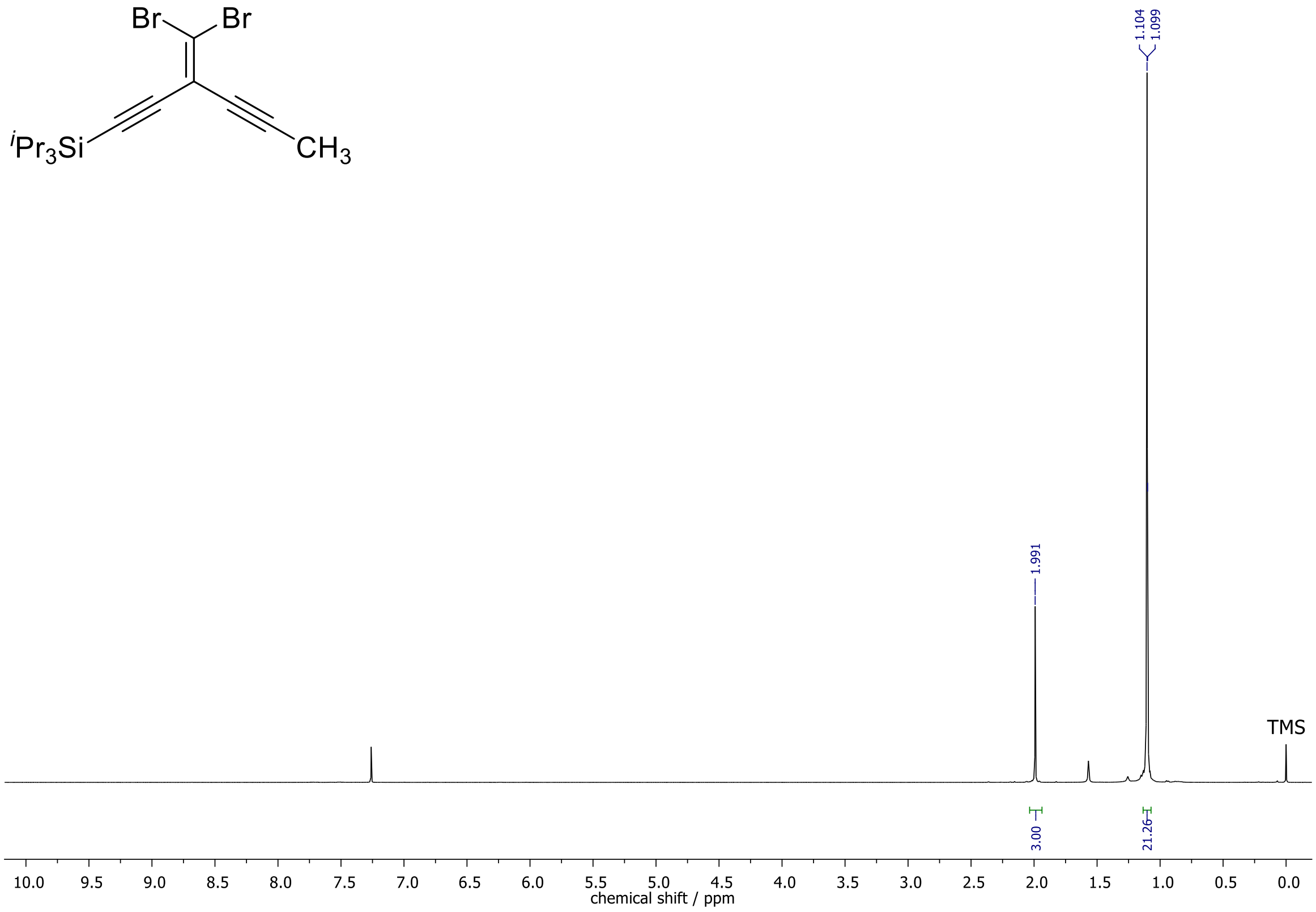

Supplementary Figure 61: ^{1}H NMR spectrum of **S6**.

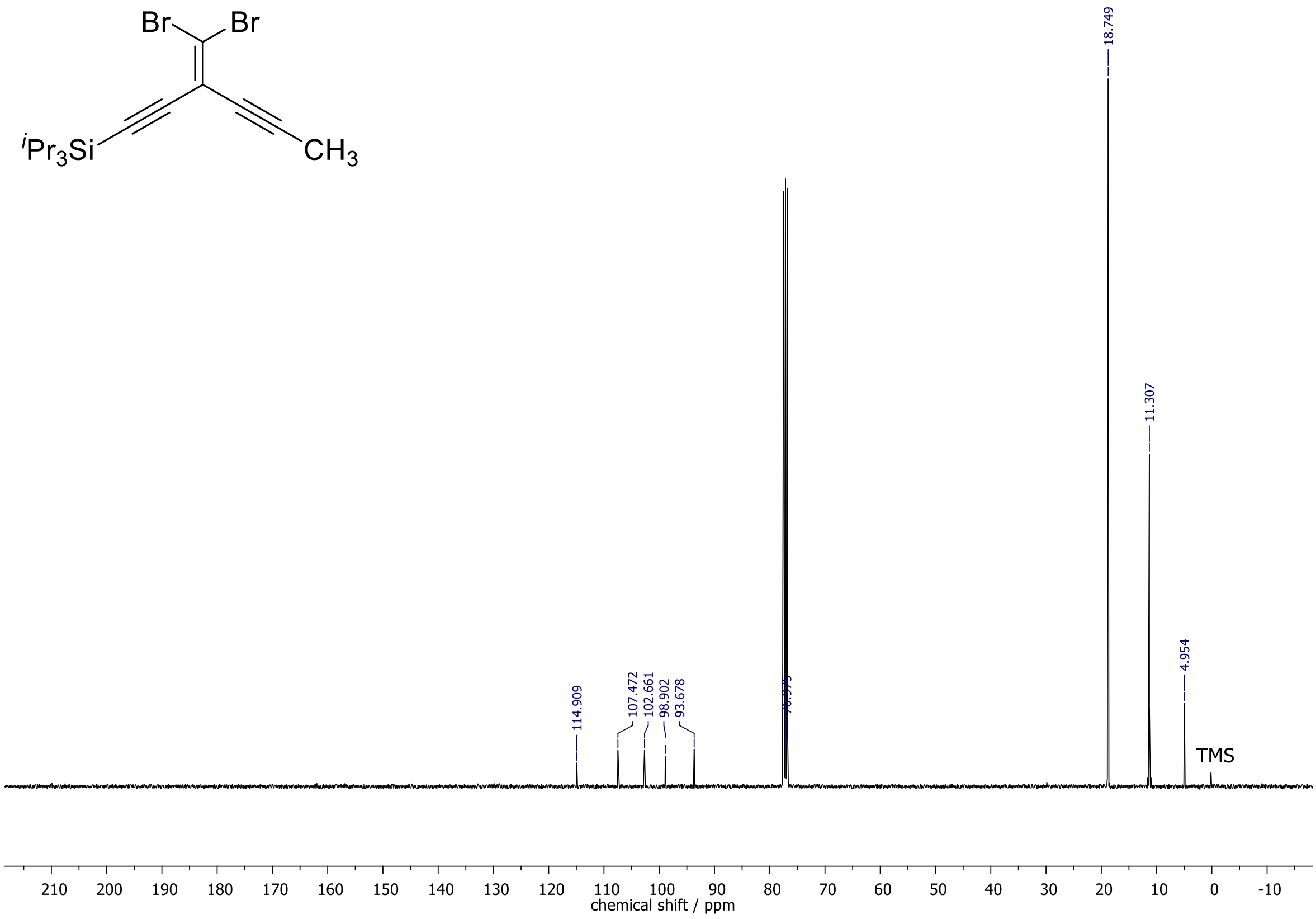

Supplementary Figure 62: ^{13}C{^{1}H} NMR spectrum of **S6**.

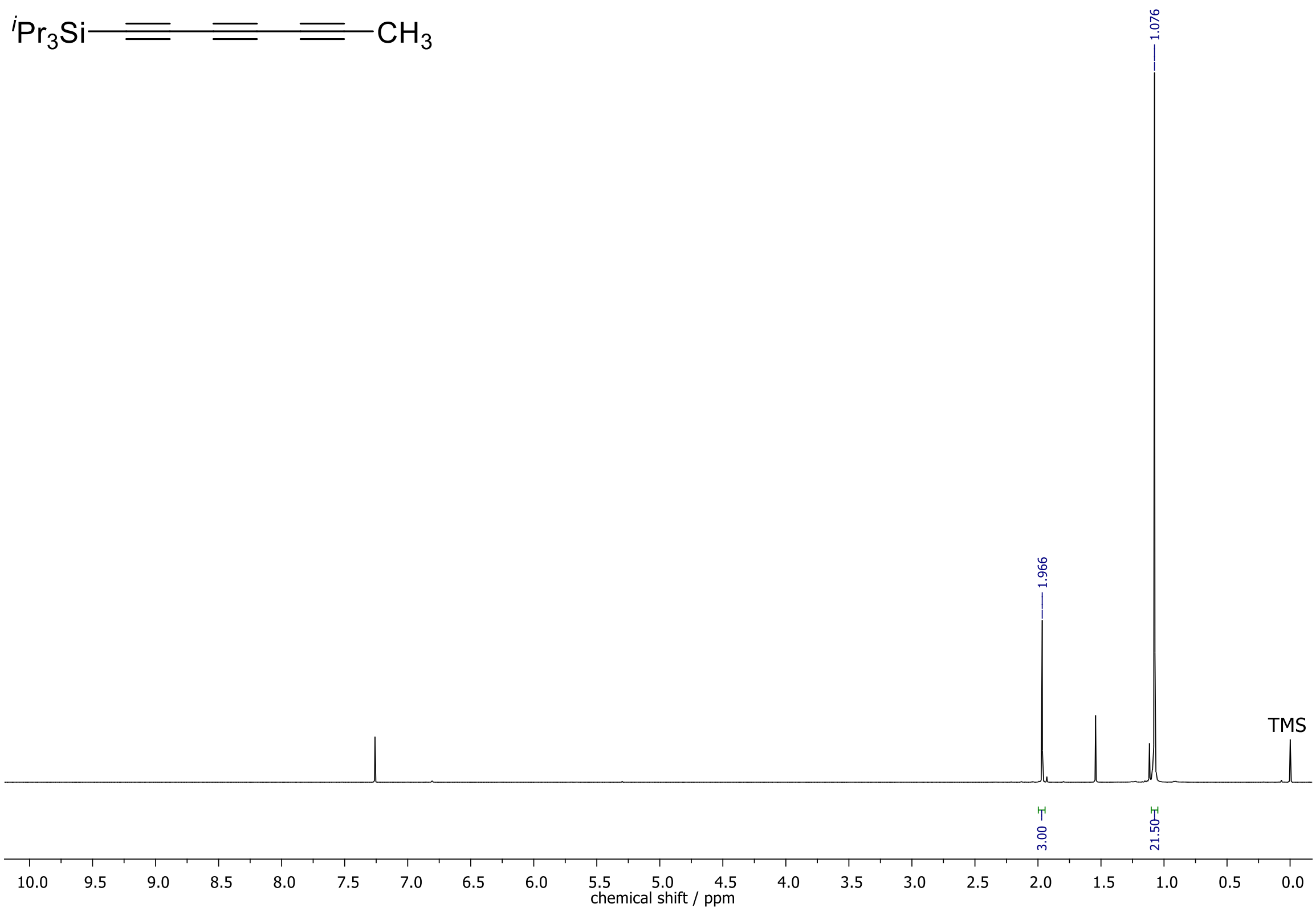

Supplementary Figure 63: ^{1}H NMR spectrum of **S7**.

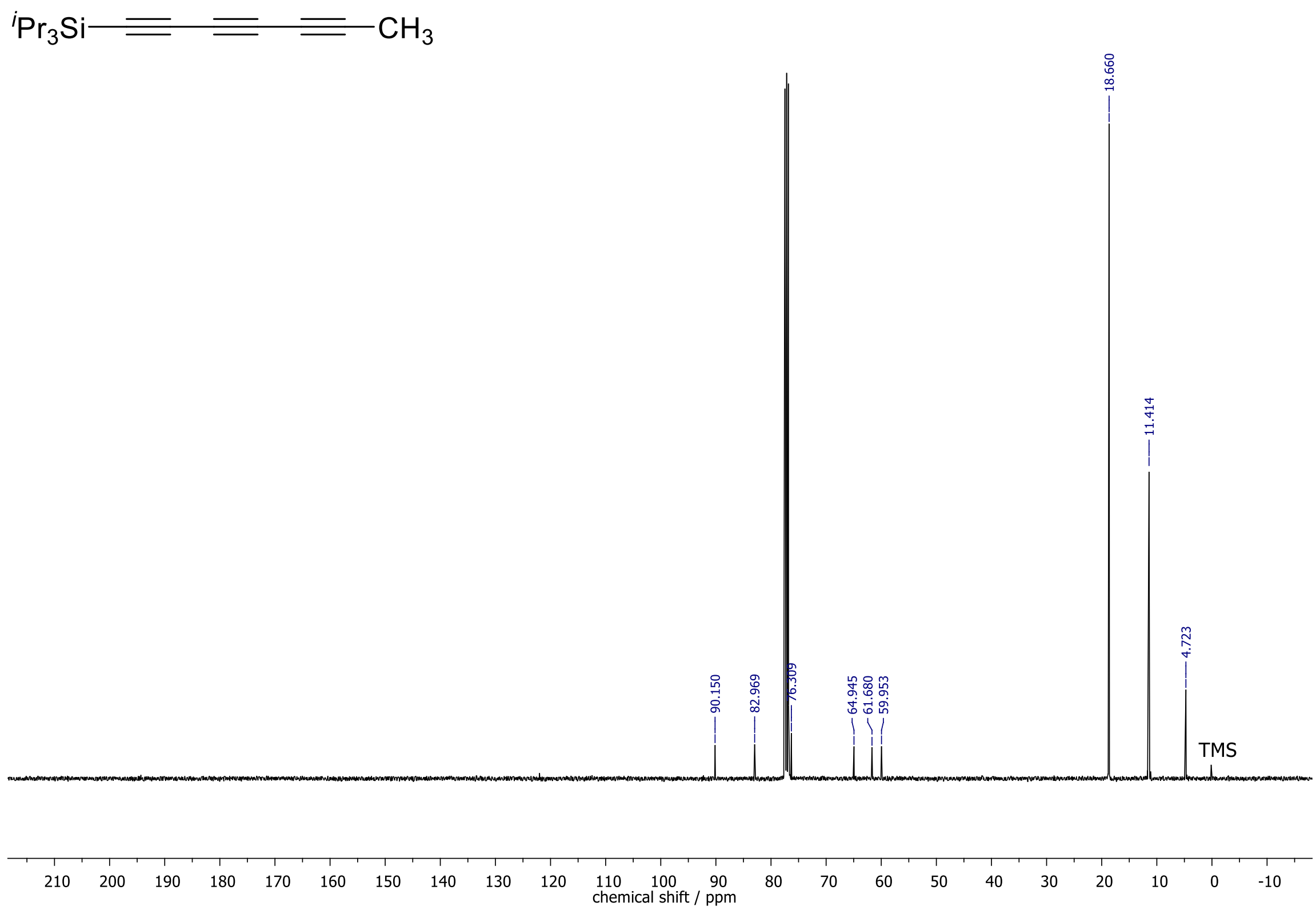

Supplementary Figure 64: $^{13}C\{^1H\}$ NMR spectrum of **S7**.

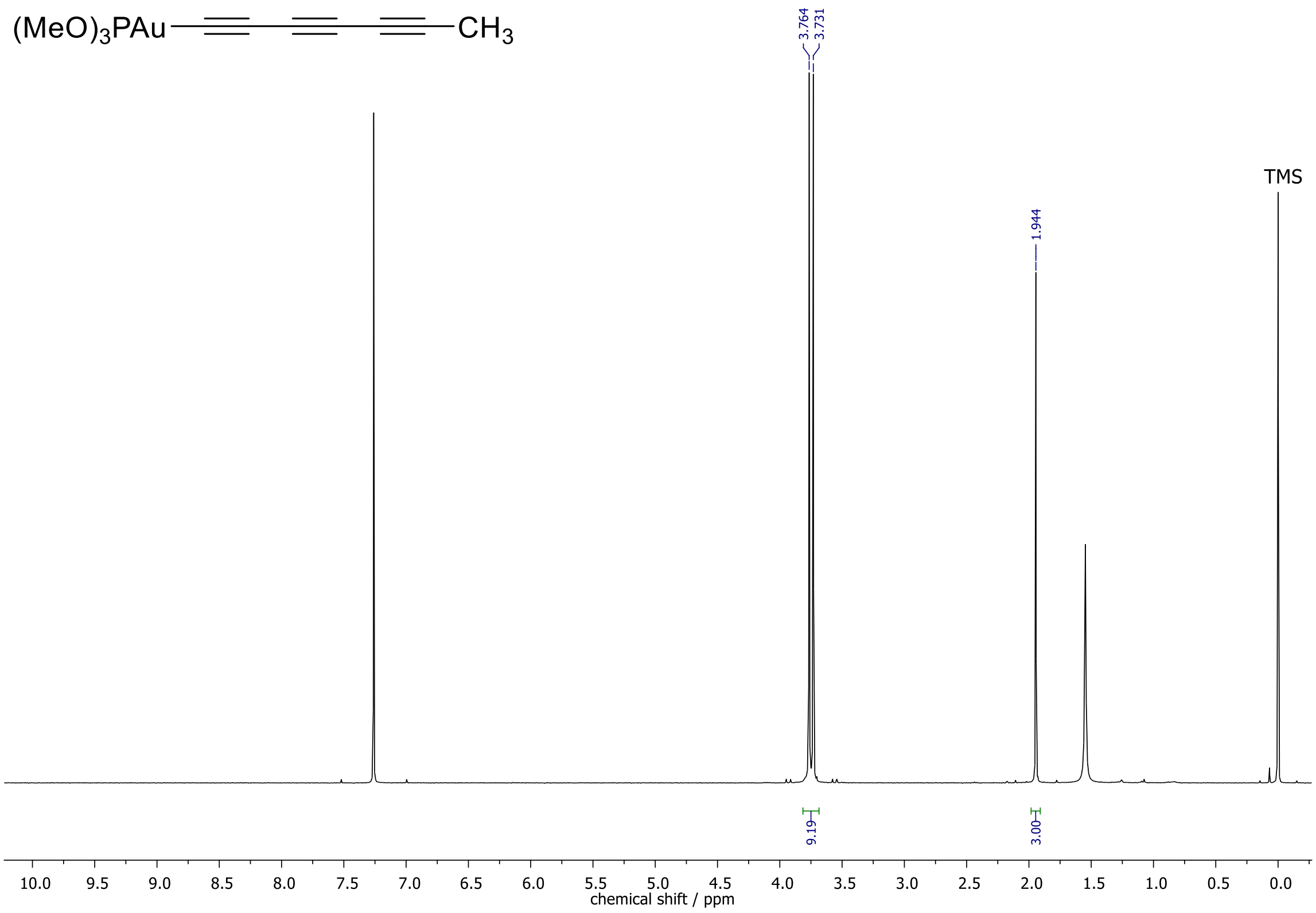

Supplementary Figure 65: ^{1}H NMR spectrum of **A4**.

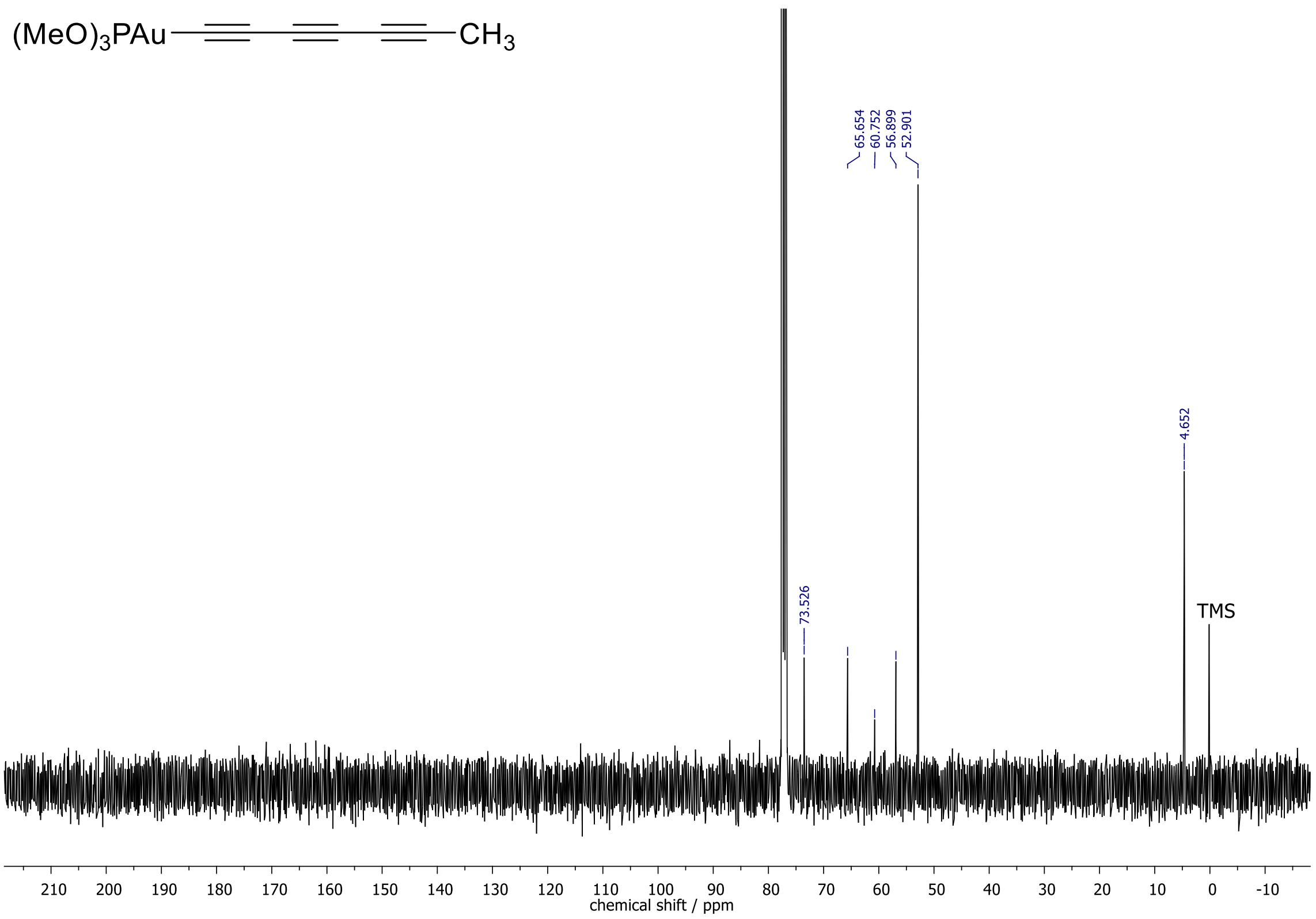

Supplementary Figure 66: $^{13}C\{^{1}H\}$ NMR spectrum of **A4**.

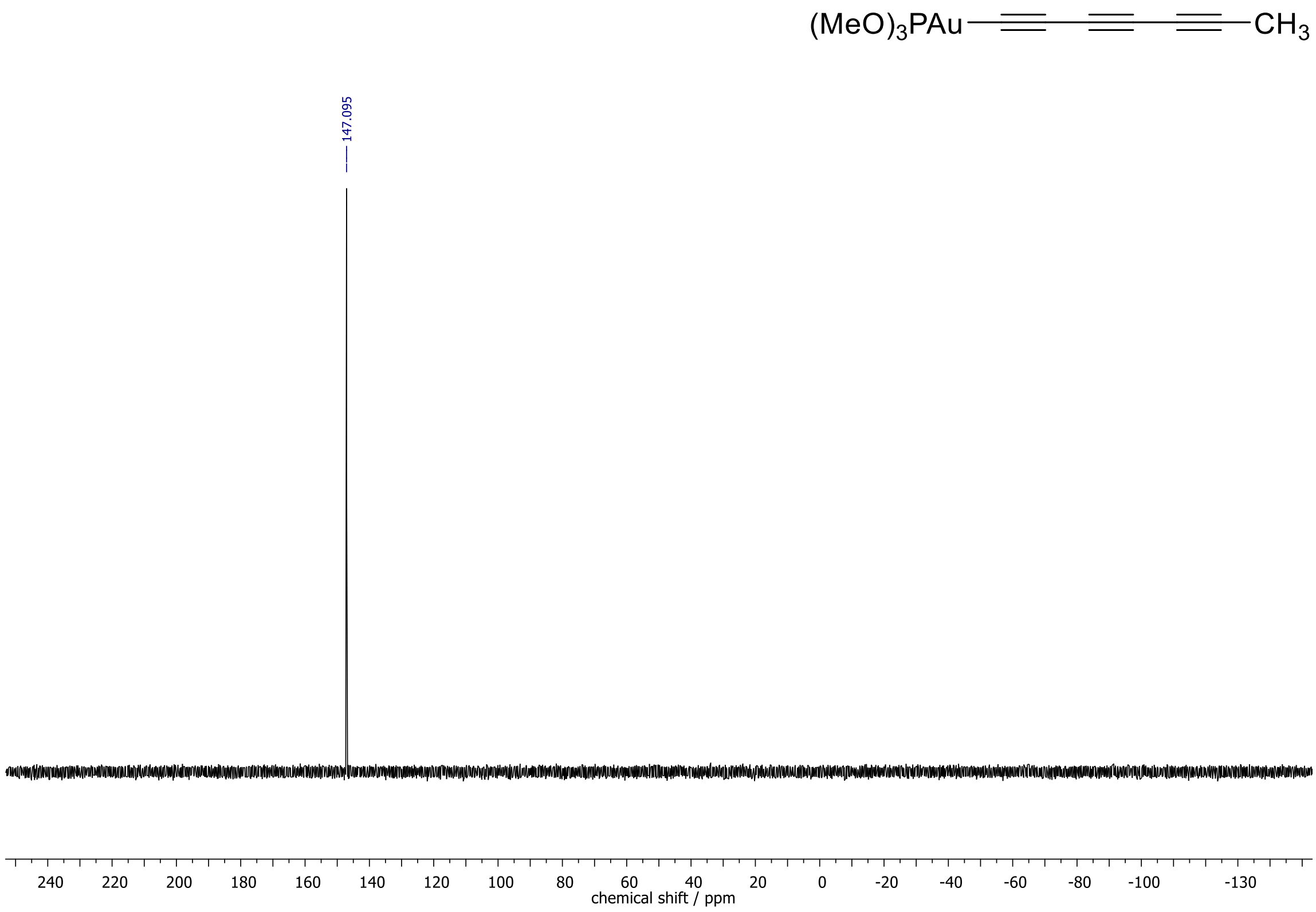

Supplementary Figure 67: $^{31}P\{^1H\}$ NMR spectrum of **A4**.

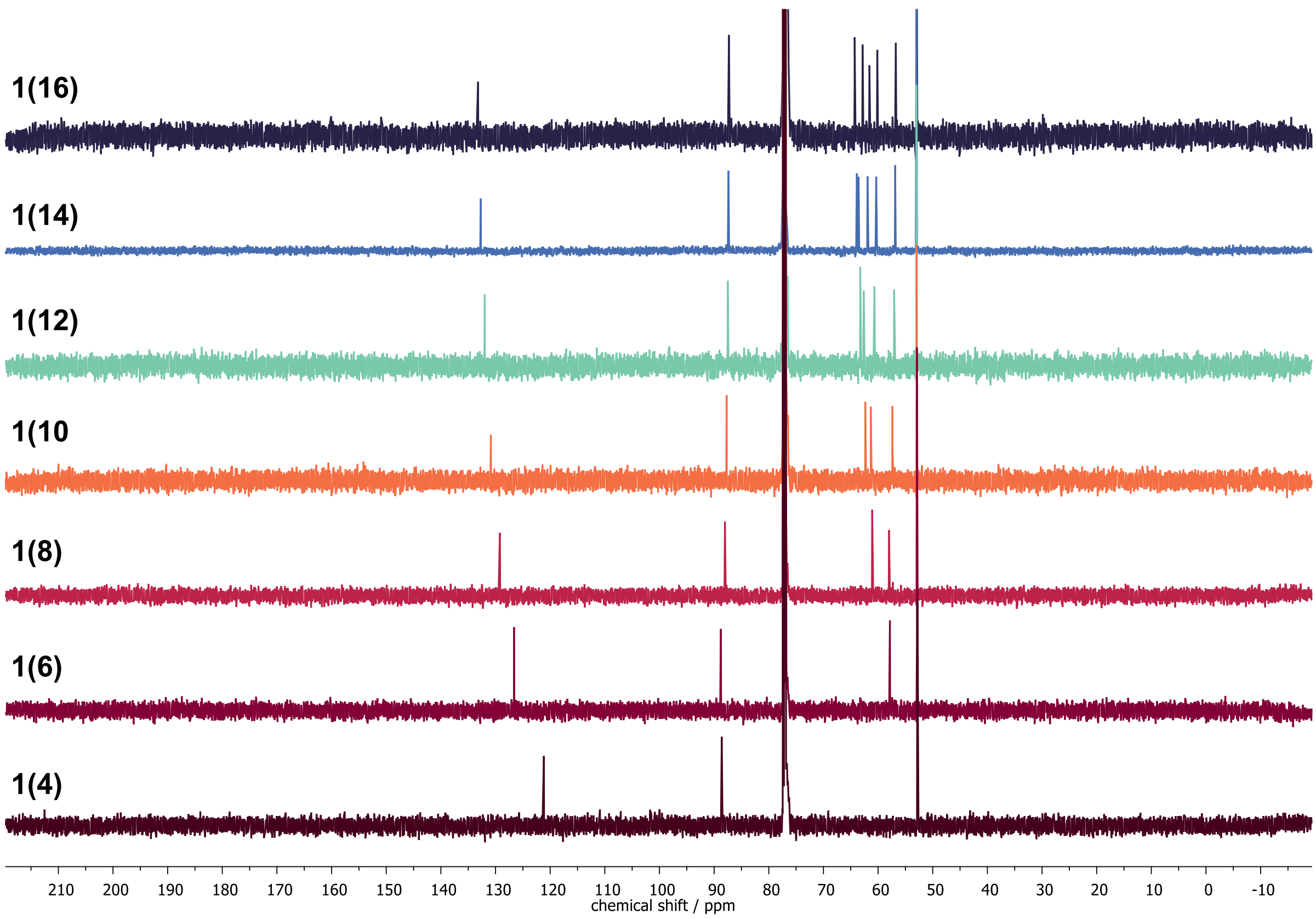

Supplementary Figure 68: Comparison of $^{13}C\{^1H,^{31}P\}$ NMR spectra of series **1(n)**.

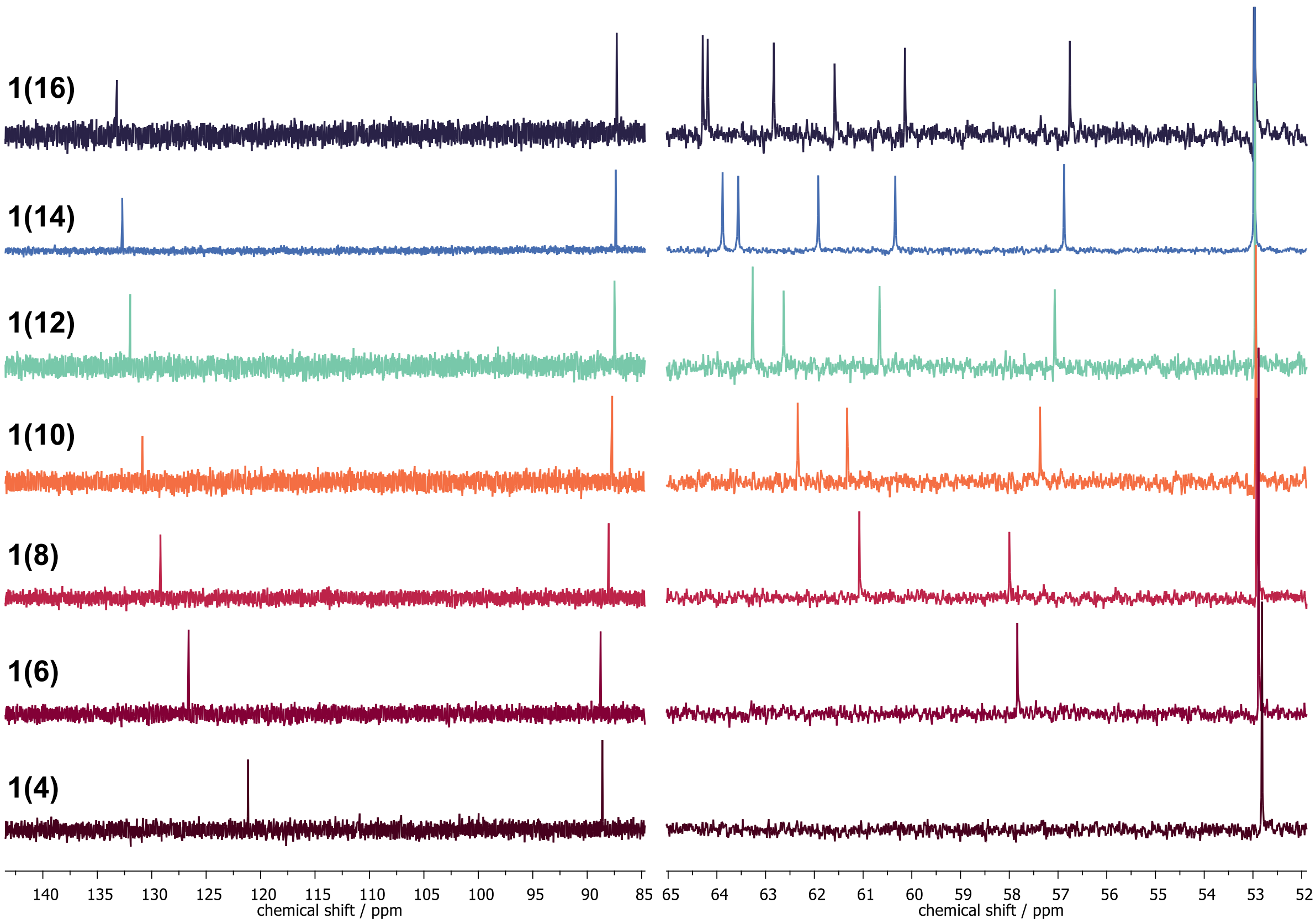

Supplementary Figure 69: Comparison of expanded $^{13}C\{^{1}H,^{31}P\}$ NMR spectra of series **1(n)**.

Supplementary Table 1: Summary of $^{13}C\{^{1}H,^{31}P\}$ NMR data of series **1(n)**.

	P(O**Me**)$_3$	Au–**C**≡C	Au–C≡**C**	Other
**1(4)**	52.8	121.2	88.6	–
**1(6)**	52.9	126.6	88.8	57.8
**1(8)**	52.9	129.2	88.0	58.0, 61.1
**1(10)**	52.9	130.9	87.7	57.4, 61.3, 62.3
**1(12)**	53.0	132.0	87.5	57.1, 60.7, 62.6, 63.3
**1(14)**	53.0	132.7	87.4	56.9, 60.3, 61.9, 63.6, 63.9
**1(16)**	53.0	133.2	87.3	56.8, 60.1, 61.6, 62.8, 64.2, 64.3

1.7 UV-Vis Absorption Spectra

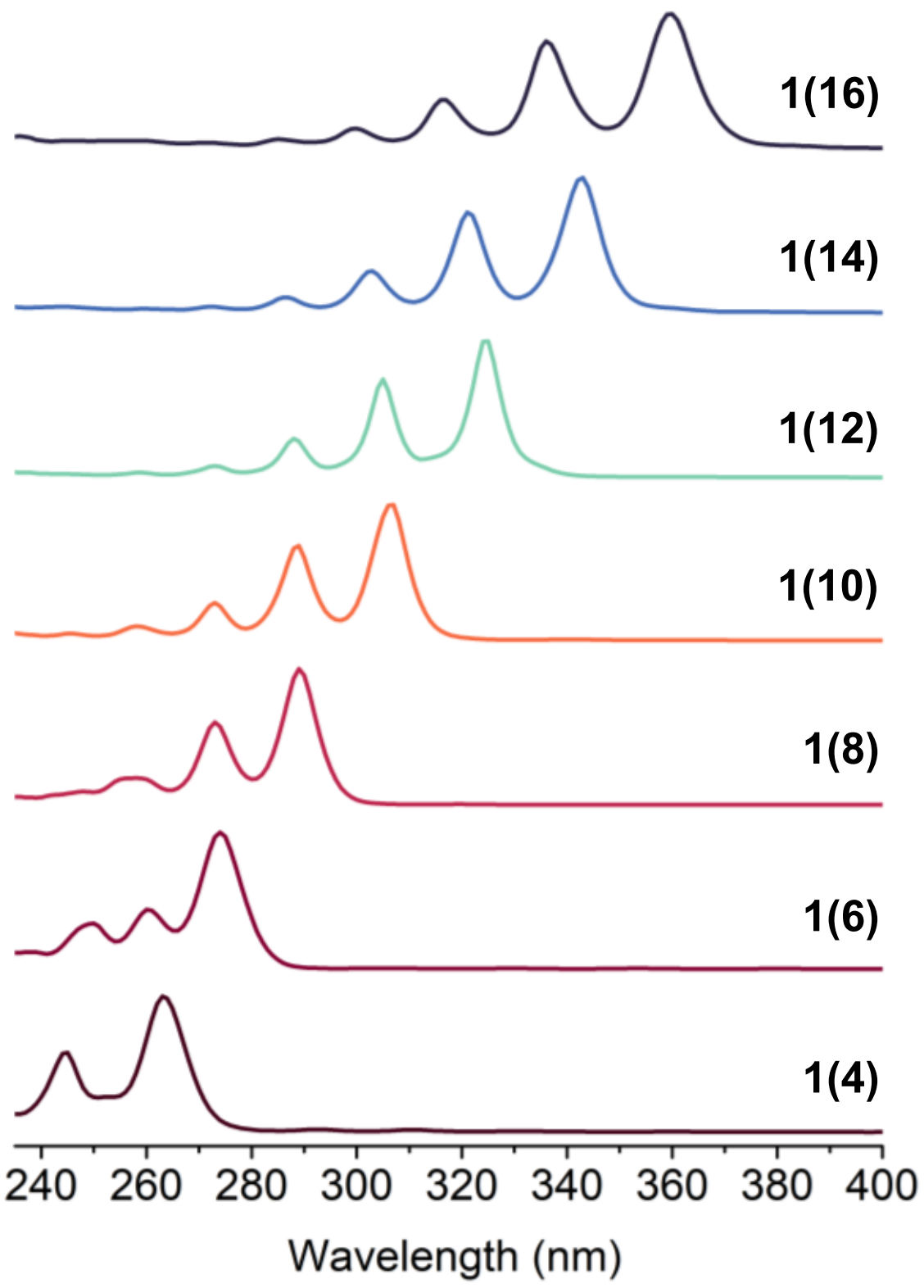

Supplementary Figure 70: Normalised UV-Vis absorption spectra of series **1(n)** illustrating the structured vibronic series λ_{main}.

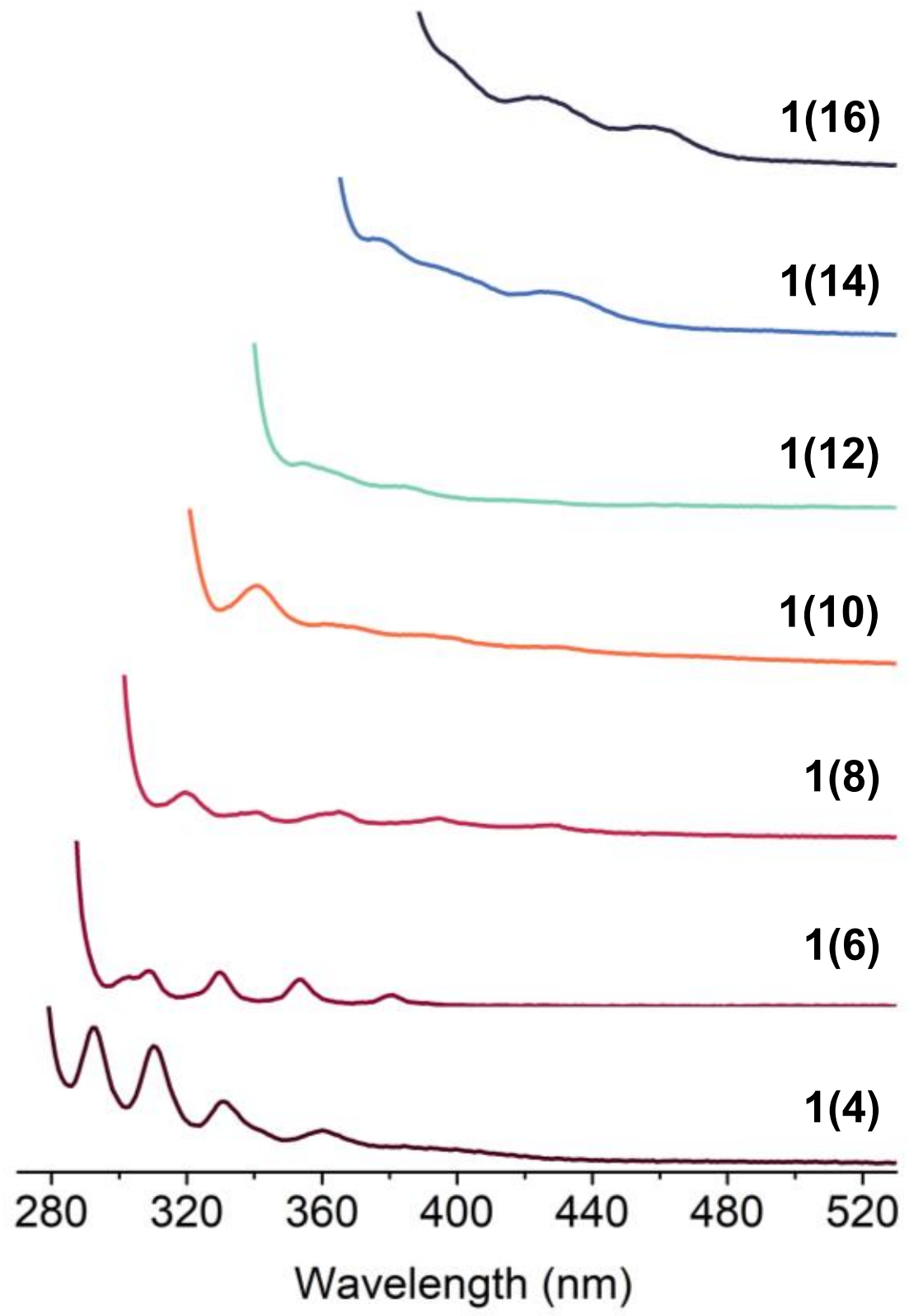

Supplementary Figure 71: Expanded UV-Vis absorption spectra of series **1(n)** illustrating the vibronic series associated with λ_{weak}. The scale of y axes is 0 – 0.025 (arbitrary units).

UV-Vis absorption spectra of series **1(n)** (Supplementary Figure 70) display a consistent pattern of high intensity absorption bands that shift to higher wavelength with increasing length (Supplementary Table 2). Expansion of the UV-Vis absorption spectra reveal weak absorption bands (maximum relative absorbance of 0.022) at longer wavelengths (Supplementary Figure 71) which become less resolved with increasing length. An in-depth analysis of such phenomena observed in the expanded and unexpanded spectra of polyynes has been presented by Guidi, Corminboeuf, Tykwinski, Frauenrath and their colleagues.[24] These authors have noted the $S_0 \rightarrow S_1$ transition is dipole forbidden, and that the weak absorption features (λ_{weak}) likely arise from $S_0 \rightarrow S_{2/3}$ transitions, which gain intensity through local breaking of molecular symmetry. The more prominent vibronic series (λ_{main}) have been attributed to transitions to high excited states $S_0 \rightarrow S_n$. The dark states make the accurate extraction of an optical gap challenging for these pseudo-linear compounds.

Supplementary Table 2: Summary of UV-Vis absorption data for series **1(n)**.

	λ_{main} (nm)	λ_{onset}^{a} (nm)	E_{onset} (eV)
**1(4)**	263, 245	280	4.43
**1(6)**	274, 260, 250	288	4.30
**1(8)**	289, 273, 258	303	4.09
**1(10)**	307, 289, 273, 258, 245	320	3.87
**1(12)**	325, 305, 288, 273, 259	341	3.64
**1(14)**	343, 321, 303, 287, 272, 260	358	3.46
**1(16)**	360, 336, 317, 300, 285, 272	379	3.27

[a]The onset wavelength of the highest intensity absorption band (λ_{main}) determined from the point at which ΔAbs per nanometer exceeds 0.003.

1.8 Single Crystal X-ray Diffraction Data

Crystals suitable for X-ray diffraction of **1(4)** and **1(6)** could be obtained by slow diffusion with CH_2Cl/pentane at 0 ℃ (Supplementary Table 3).

Supplementary Table 3: Crystal structure and refinement details.

	**1(4)**	**1(6)**
CCDC Number	**2440861**	**2440860**
Empirical formula	$C_{10}H_{18}Au_2O_6P_2$	$C_{12}H_{18}Au_2O_6P_2$
Formula weight	690.12	714.14
Temperature/K	100.01(10)	100.01(10)
Crystal system	Monoclinic	Monoclinic
Space group	$P2_1/c$	$P2_1/c$
a/Å	8.0582(2)	7.42340(10)
b/Å	7.78420(10)	18.6565(2)
c/Å	13.3575(2)	6.44870(10)
α/°	90	90
β/°	90.295(2)	91.6110(10)
γ/°	90	90
V/Å^3	837.86(3)	892.76(2)
Z	2	2
ρ_{calc} g/cm^3	2.735	2.657
μ/mm^{-1}	34.444	32.366
F(000)	628	652
Crystal size/mm^3	0.124×0.109×0.053	0.087×0.033×0.02
Radiation	Cu Kα (λ = 1.54184)	Cu Kα (λ = 1.54184)
2Θ range for data collection/°	10.98 to 152.74	9.482 to 150.638
Index ranges	$-9 \le h \le 10$, $-9 \le k \le 9$, $-12 \le l \le 16$	$-9 \le h \le 8$, $-23 \le k \le 23$, $-8 \le l \le 8$
Reflections collected	7703	16651
Independent reflections	1708 [R_{int} = 0.0385, R_{sigma} = 0.0286]	1812 [R_{int} = 0.0462, R_{sigma} = 0.0205]
Data/restraints /parameters	1708/0/94	1812/0/103
Goodness-of-fit on F^2	1.125	1.135
Final *R* indexes [$I \ge 2\sigma(I)$]	R_1 = 0.0242, wR_2 = 0.0584	R_1 = 0.0229, wR_2 = 0.0617
Final *R* indexes [all data]	R_1 = 0.0253, wR_2 = 0.0591	R_1 = 0.0256, wR_2 = 0.0632
Largest diff. peak/hole / e Å^{-3}	1.07/−1.24	0.88/−1.26

Both compounds **1(4)** and **1(6)** crystallise in a monoclinic $P2_1/c$ space group where the asymmetric unit is constituted by half a molecule. The rest of the molecule is generated through an inversion centre situated in the middle of the carbon chain. Both compounds present a slight bend around the C1 atom, yielding a wavy aspect of the polyyne chain (Supplementary Figure 72). The triple bonds present similar lengths of 1.206(7) and 1.204(6)/1.203(8) Å for **1(4)** and **1(6)**, respectively, in the 1.2 Å range usually reported in the literature for C≡C bonds (Supplementary Table 4). The single bonds follow the same pattern, with 1.39(1) and 1.381(6) Å in **1(4)** and **1(6)**, respectively. The environment of the gold atom is comparable in both complexes as well with Au-C1/Au-P bond length of 1.994(5)/ 2.246(1) and 2.010(4)/ 2.255(1) Å in **1(4)** and **1(6)**, respectively. However, the length of the chain seems to affect the packing in the crystal. With four carbon atoms, the distance between

two gold atoms of 7.7783(3) Å seems to not be sufficient to accommodate two bulky P(OMe)$_3$ groups facing each other in neighbouring molecules. Therefore, the packing of **1(4)** presents a herringbone pattern evident following the *a* and *c* crystallographic axes (Supplementary Figure 73) with an intermolecular Au-Au distance of 3.7754(4) Å. The carbon chains are oriented in a parallel fashion following the *b* axis, a feature also observed for **1(6)** (Supplementary Figure 74). In contrast, a chain containing six carbon atoms (a Au-Au intramolecular distance of 10.333(1) Å) appear to be the ideal length to allow face-to-face arrangements of the P(OMe)$_3$ groups, yielding a well packed parallel arrangement of the molecules in the crystal and the formation of aurophilic interactions. Compared to **1(4),** the distance between two neighbouring gold atoms is reduced to 3.2251(1) Å in **1(6)**, evident in the layers organised in a ladder fashion following the *a* and *b* axes. The efficiency of such packing is again demonstrated following the *c* axis with interlocked zig-zag arrangements of neighbouring molecules.

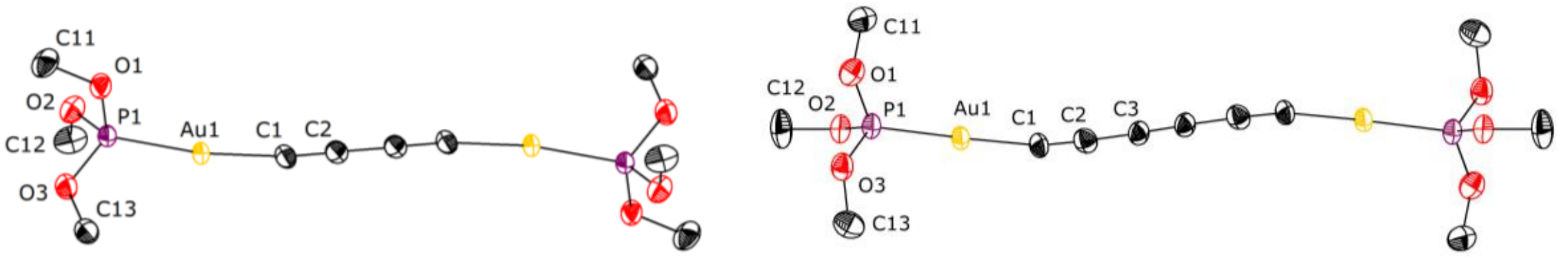

Supplementary Figure 72: Crystal structure obtained for compound **1(4)** (left) and **1(6)** (right). H atoms are omitted for clarity

Supplementary Table 4: Selected bond lengths and angles.

	**1(4)**	**1(6)**
C1-C2	1.206(7)	1.204(6)
C2-C3	n/a	1.381(6)
C2-C2* / C3-C3*	1.39(1)	1.203(8)
Au-C1	1.994(5)	2.010(4)
Au-P1	2.246(1)	2.255(1)
Au-Au (intramolecular)	7.7783(3)	10.333(1)
Au-Au (intermolecular)	3.7754(4)	3.2251(1)

*symmetry related atoms; C2 in **1(4)** and C3 in **1(6)**

Crystallographic Packing

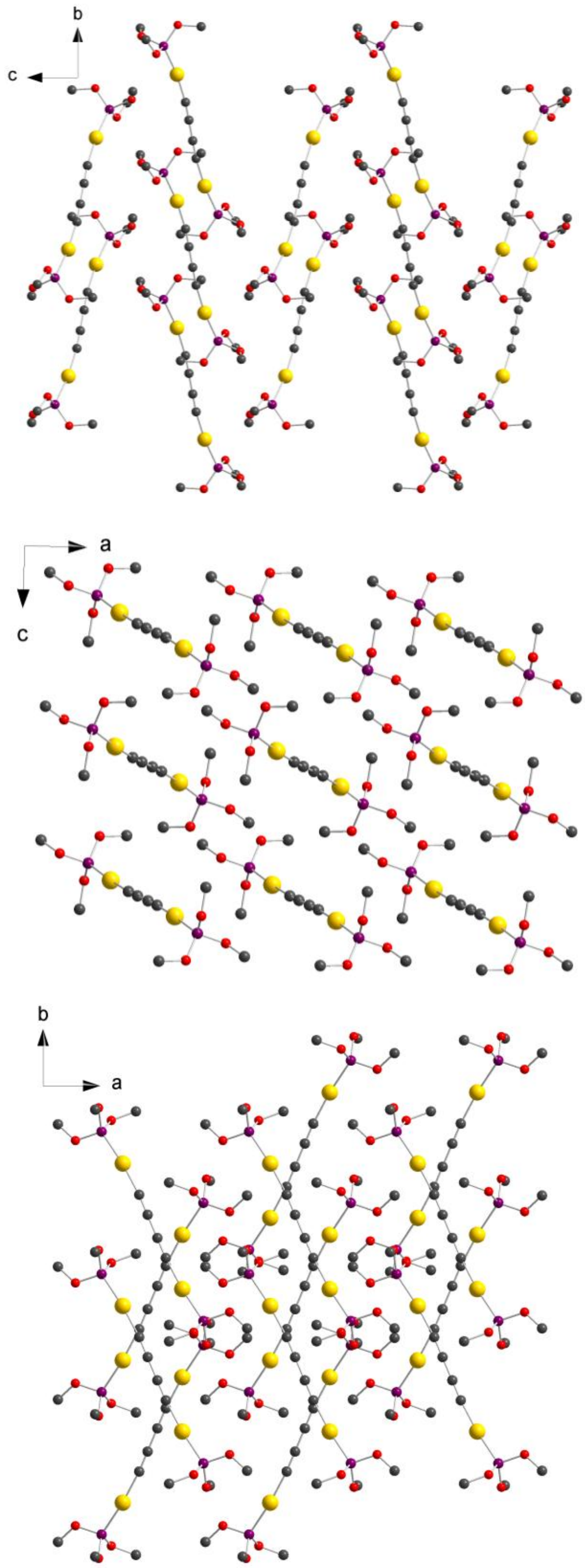

Supplementary Figure 73: Molecular packing of **1(4)** following *a* (top), *b* (middle) and *c* (bottom) crystallographic axes.

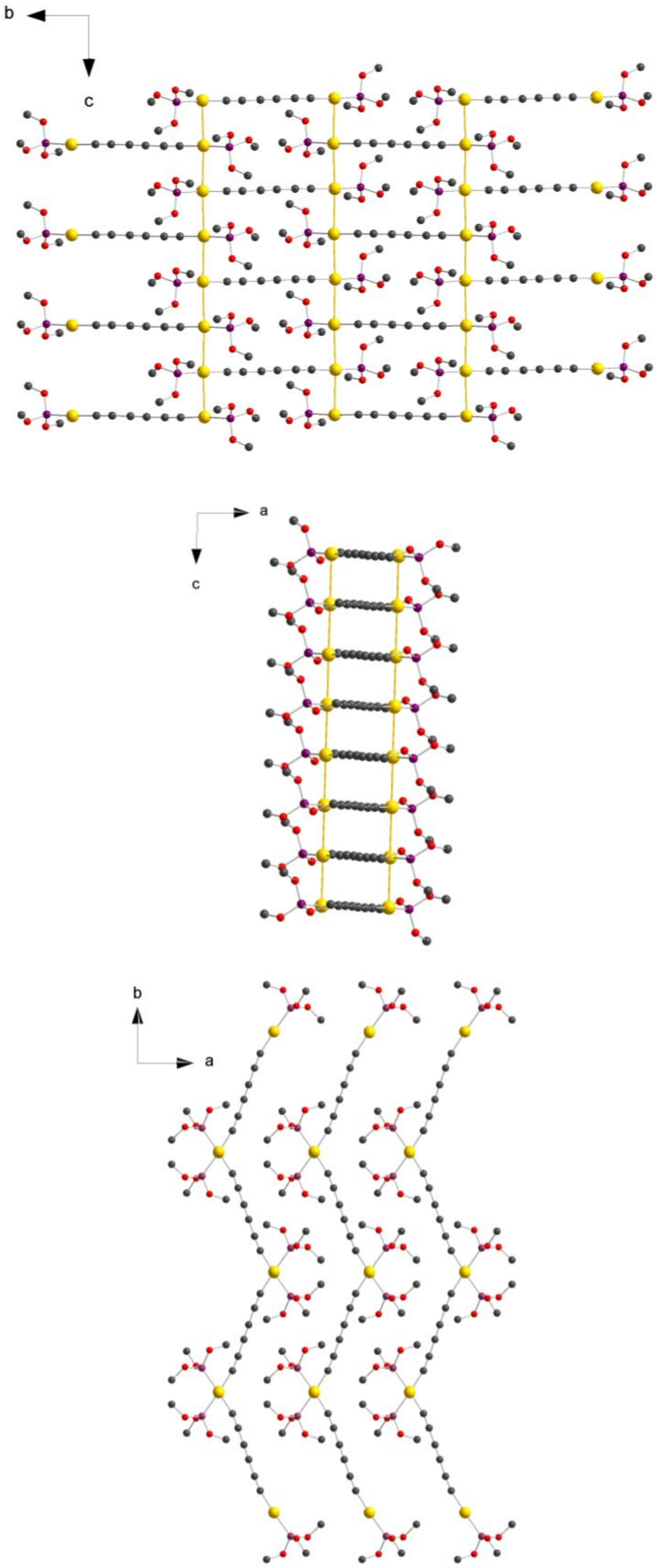

Supplementary Figure 74: Molecular packing of **1(6)** following *a* (top), *b* (middle) and *c* (bottom) crystallographic axes.

2. STM Measurements

2.1 Materials

We used 0.25 mm diameter Au tips mechanically cut from a spool of Au wire (Goodfellows Precious Metals, 99.99+% or ThermoFisher Scientific PREMION 99.998%), and Au-on-mica substrates obtained by e-beam evaporation (Korvustech Tau4) of Au (Advent Research materials, 99.99+%) onto freshly cleaved mica substrates (Agar Scientific Muscovite). Solvents were purchased from TCI UK, with 98%+ purity, and used without further purification.

2.2 Instrumentation

In this study, we employed a modified Keysight 5500 STM to fabricate and characterise single-molecule junctions. While we have described the instrument in detail in our previous publications,[25–27] recent upgrades to both the hardware and the software warrant an updated complete description.

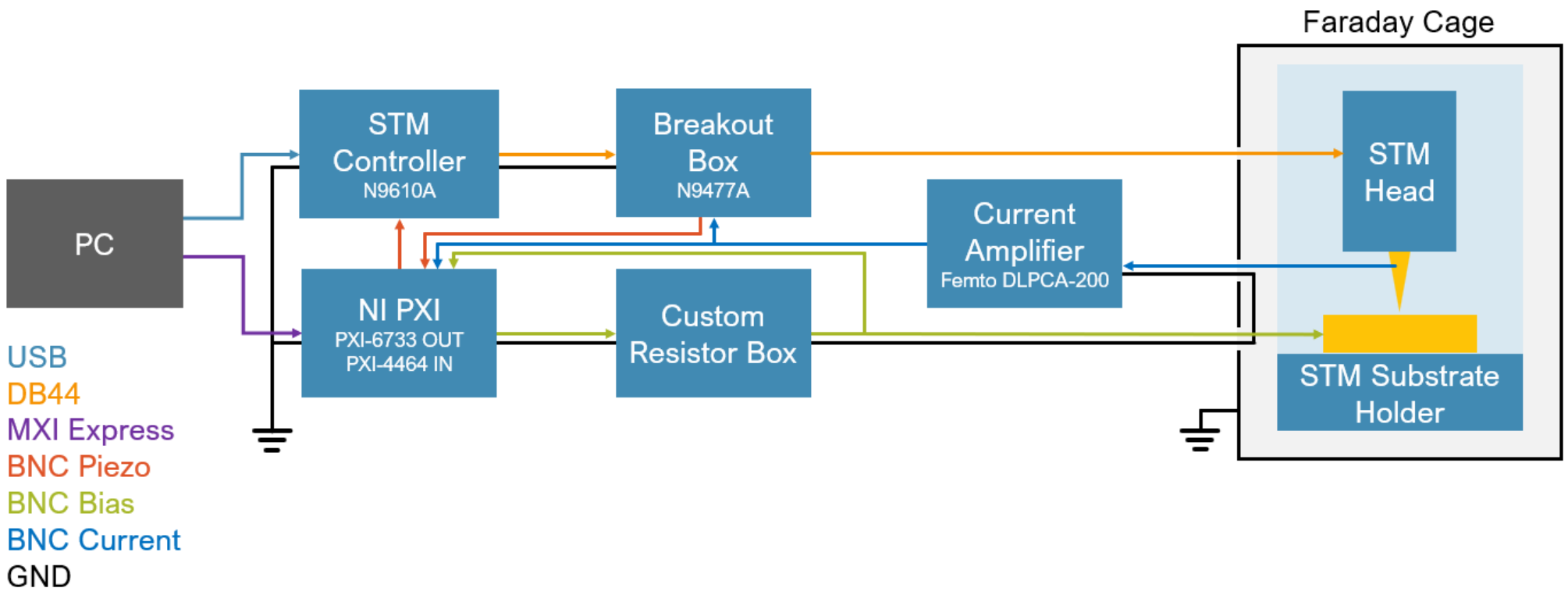

Supplementary Figure 75: Schematics of the modified Keysight 5500 STM "Leviathan3"

The instrument (*Leviathan III*) uses a Keysight N9610A controller only to perform the initial tip-substrate approach. Once the setpoint current is reached, the feedback loop is disabled, and control of the instrument is left to a bespoke LabVIEW VI. The VI uses a National Instruments PXI system (PXIe-1062Q chassis and PXIe-PCIe8381 interface) to interface with the Keysight controller *via* a breakout box (Keysight N9477A). Signals (piezo position, junction bias) are imposed with a PXI-6733 module (through a BNC-2110 terminal) and acquired with a PXI-4464 module. All data is managed point-by-point in real time, in this study at frequencies of $20 - 40\ kHz$ depending on the experiment. The voltage is applied to the junction by the PXI-6733 in series with a resistor ($10\ - 100\ k\Omega$ in this study) to protect the sensitive current amplifier (Femto DLPCA-200) from overload during the formation of the Au microcontact. The presence of the resistor causes an effective bias drop when the conductance of the junction is $> 0.15\ G_0$, allowing us to probe conductance over approximately 7 orders of magnitude with a single-channel current amplifier at a fixed amplification ($10^5 - 10^6\ VA^{-1}$). The bias is recorded after the resistor to directly assess this bias drop and measure the exact bias applied across the junction. Furthermore, the bias drop means that atomic contacts having conductance $1 - 5\ G_0$ are only subjected to a fraction of the full bias applied, protecting them from electromigration phenomena.

At the beginning of each fabrication cycle, a proportional-integral-derivative (PID) controller approaches the tip to the substrate until a current of $0.1\ \mu A$ is detected. After this, the PID is disabled and a single sawtooth ramp is applied by the PXI-6733 module to the piezo transducer, resulting in the tip being driven into the substrate, followed by a retraction by a user-defined distance within a specified time (at rates of $10 - 40\ nm\ s^{-1}$). The

system also includes an offsetting capability to ensure the tip is sufficiently crashed before retraction, resulting in fresh tip reconstruction at each cycle. The junction conductance is continuously monitored by the PXI-4464 module, along with the tip position (reconstructed from the voltage applied to the piezo transducer using its calibration of $19.5\ Å\ V^{-1}$) and the *effective* junction bias, directly probed after the series resistor. At the end of the cycle, the PID re-engages, the tip is reapproached to a current of $0.1\ \mu A$ and another sawtooth ramp is applied. After each sawtooth ramp and before the PID re-engages, all data acquired is released from a memory buffer as an array, processed, and plotted on-the-fly. Each trace is trimmed, and an automated algorithm filters out traces that did not result in the formation of a Au-Au contact having conductance $G > 3\ G_0$ at the beginning of the cycle and that did not decay to the noise level of the instrument when the tip is fully withdrawn. All traces that satisfy these requirements are saved as raw ASCII table containing tip displacement (nm), source-drain bias (V) and junction conductance ($Log(G/G_0$).

Data acquisition is mostly unsupervised and once completed, all raw traces are trimmed and prepared for statistical analysis using another LabVIEW VI. In this VI, an automated algorithm identifies the point of rupture of the Au-Au contact, sets it as zero, and removes all data at displacements below $-1\ nm$ and above $+5nm$, while still ensuring that the conditions for data saving described earlier are met. This procedure greatly reduces the size of each trace (from $\sim 1\ MB$ to $\sim 250\ kB$) ensuring a more computationally efficient data analysis.

It is important to mention here that for the measurement of **C6 – C16** a modified protocol for data acquisition had to be used, because after 10-20 minutes of continuous junction fabrication the conductance traces would consistently fail to reach the noise level at the end of the tip withdrawal process. We[28] and others[29] noticed this phenomenon with other molecular wires bearing metallophilic groups binding strongly to Au substrates, and we attributed it to a rapidly increasing surface concentration of molecular wires (and their coordination oligomers/polymers) preventing a clean rupture of the junction and difficulties in re-engaging the electrodes to $G > G_0$. To overcome this issue, every ~50-100 traces acquired a modified ramp is applied to the piezoelectric transducer that drives the tip 100 nm into the substrate, followed by a fast retraction ($100\ nm\ s^{-1}$) until a clean tunnelling signal is recovered. Data is not recorded during this process. At the end of this extended ramp, the PID is re-engaged and data acquisition is restarted.

2.3 Additional STMBJ Data

In addition to the data shown in the main paper, we report here the conductance histograms for all compounds used in this study (Supplementary Figure 76 − Supplementary Figure 97). Due to overlapping features (*e.g.* peak corresponding to the monomeric junction overlapping with that of the dimeric junction), histograms are less conclusive than 2D maps but are reported here for completeness. The 2D density maps already shown in the manuscript are reproduced here for direct comparison.

All histograms are compiled with 100 bins per conductance decade and the y axis is normalised to the number of traces collected and reported here as $Counts/Trace$. The 2D maps are also compiled with 100 bins per conductance decade and 100 bins per nanometre, and counts are normalised to the number of traces used. All data was acquired at $200\ mV$ source-drain bias, with the target compound at a concentration of $250\ \mu M$ in anisole, except for **C4** that was acquired in anhydrous, neutralised 1,2,4-trichlorobenzene.

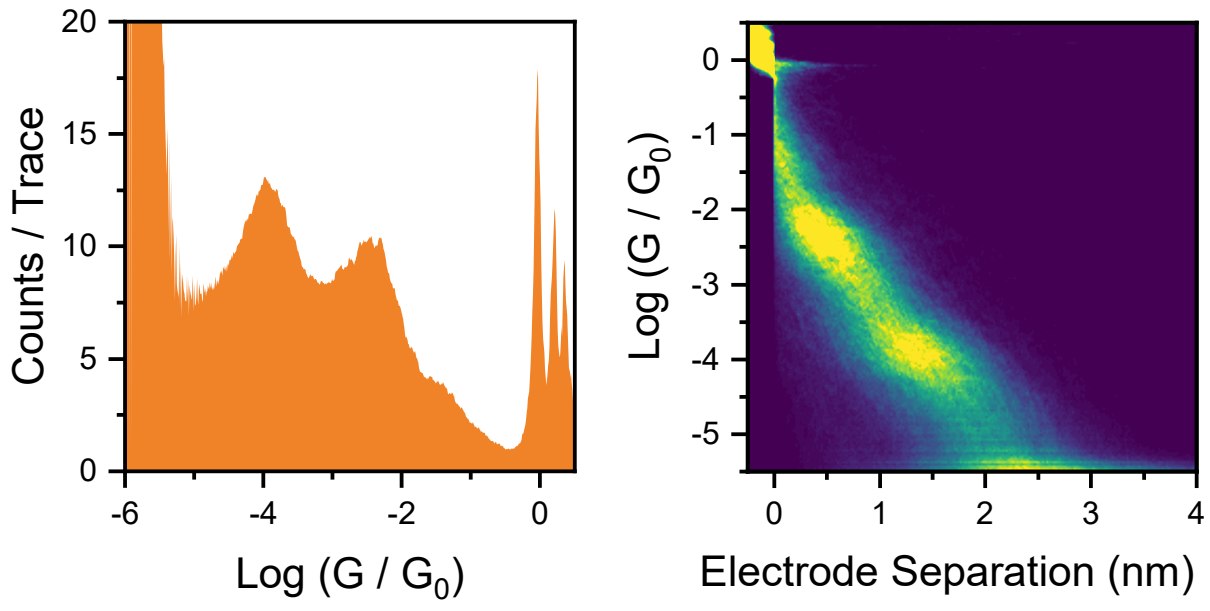

Supplementary Figure 76: Conductance histogram and density map for **C4**. The high-conductance feature does not appear clearly in the histogram, but a shoulder at $\sim 10^{-1.5} G_0$ is clearly visible.

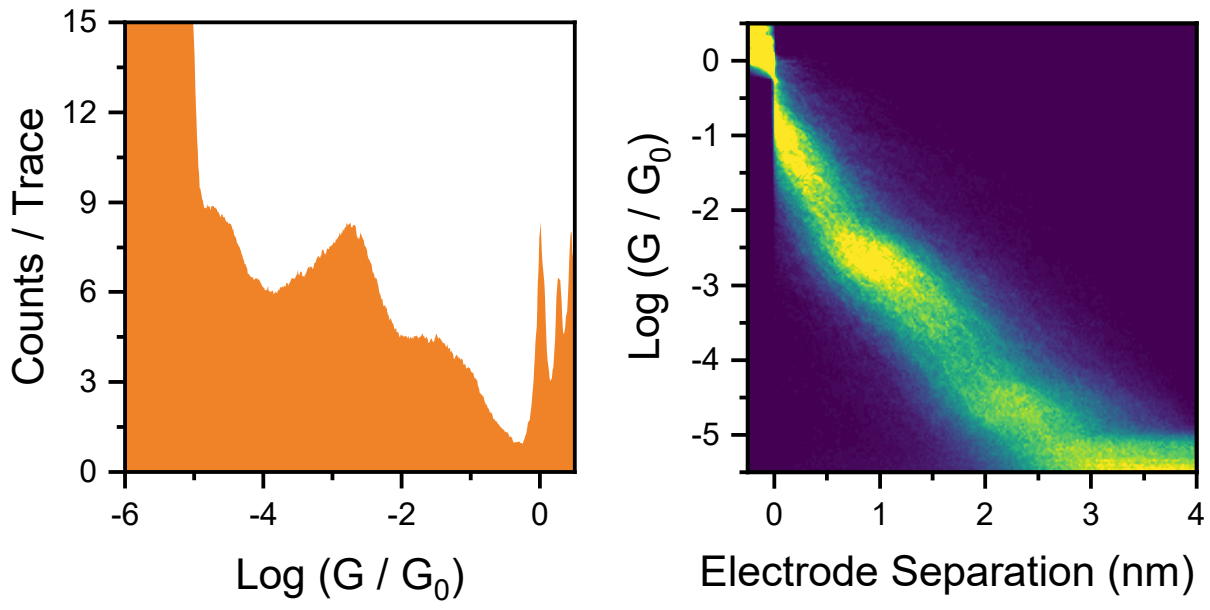

Supplementary Figure 77: Conductance histogram and density map for **C6**. Similar to **1**, the high-conductance feature clearly appears as a shoulder at $\sim 10^{-1.7} G_0$ in the histogram, and contributes to a well-defined density cloud in the 2D plot.

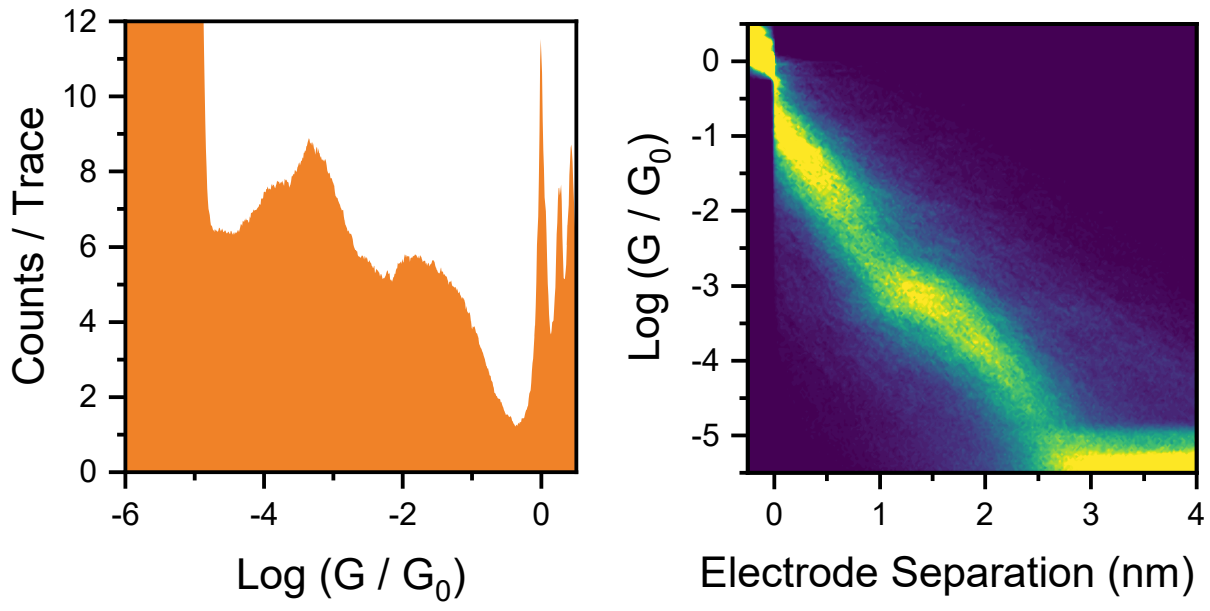

Supplementary Figure 78: Conductance histogram and density map for **C8**.

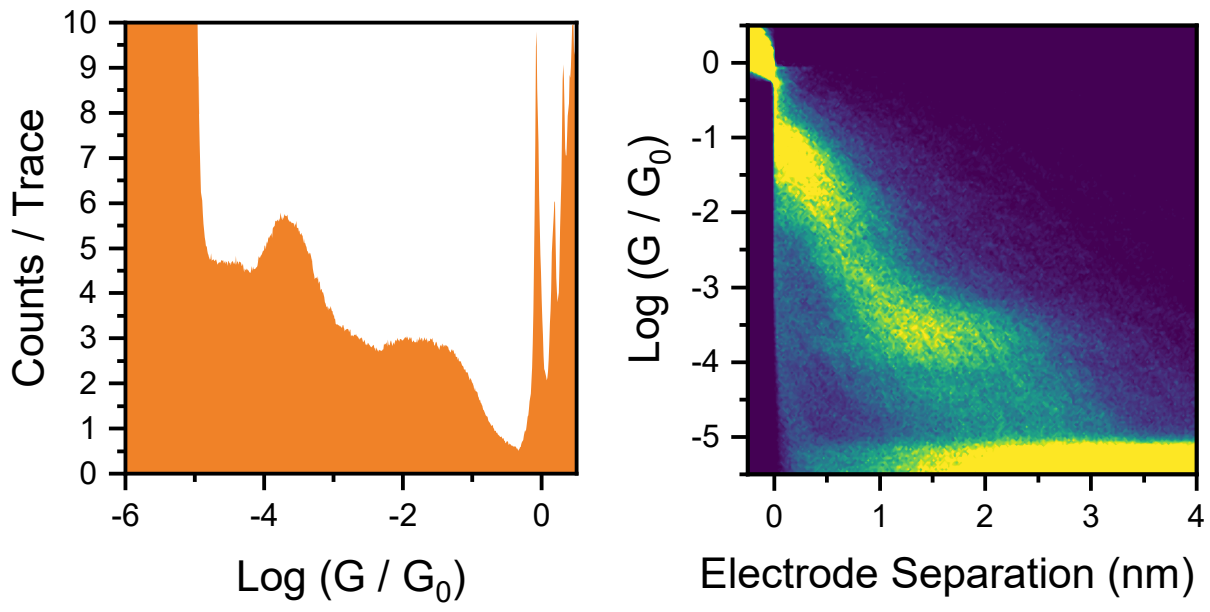

Supplementary Figure 79: Conductance histogram and density map for **C10**.

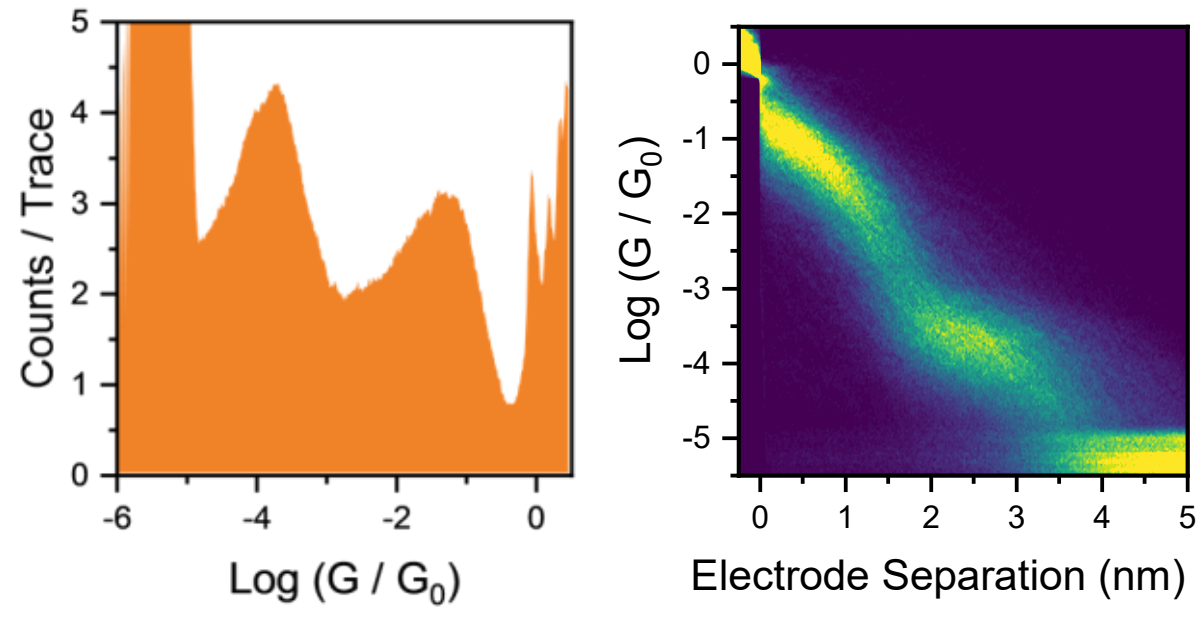

Supplementary Figure 80: Conductance histogram and density map for **C12**.

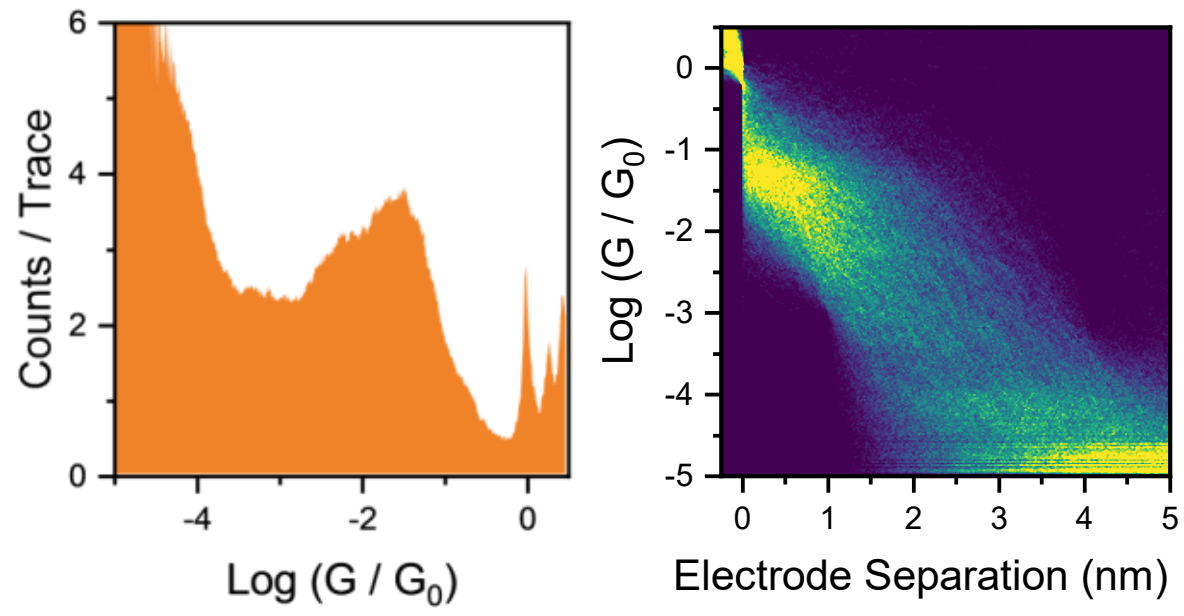

Supplementary Figure 81: Conductance histogram and density map for **C14**. Formation of the dimer can be observed near the noise level of the instrument, but its charge-transport behaviour cannot be resolved conclusively.

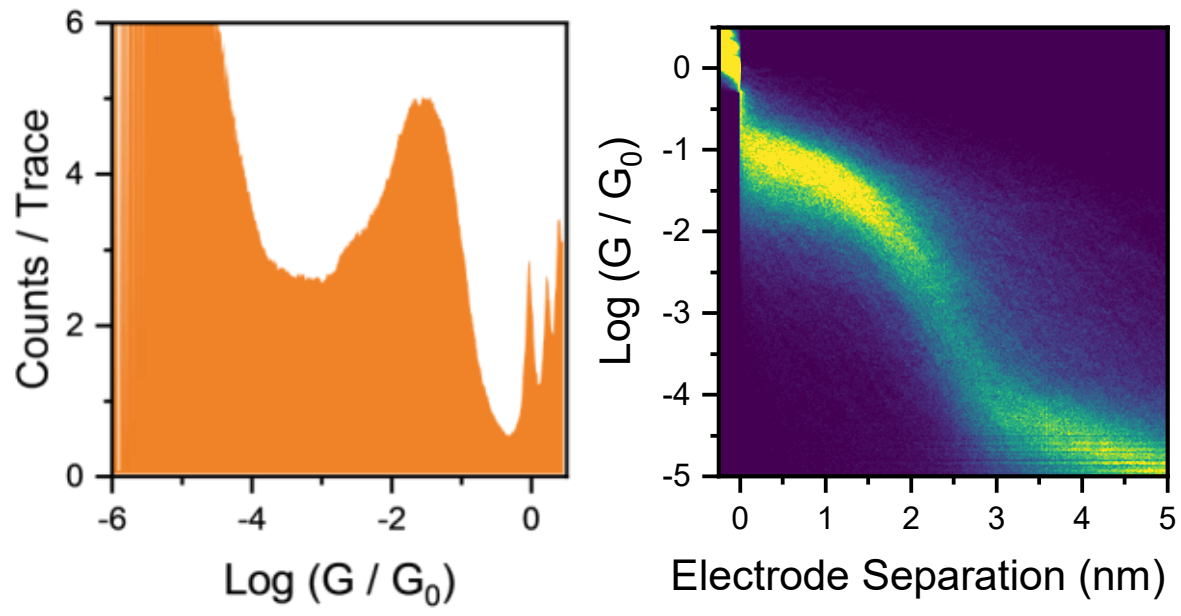

Supplementary Figure 82: Conductance histogram and density map for **C16**.

Examples of STMBJ traces for **C4-C16** can be seen below.

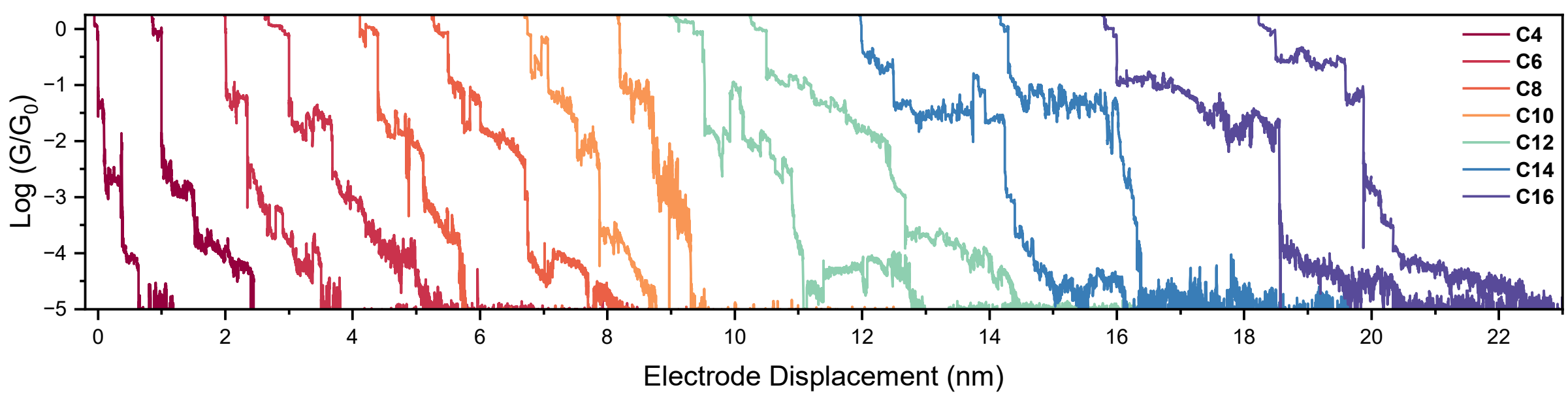

Supplementary Figure 83: Representative *STMBJ* traces for **C4-C16.**

As discussed in the main text, we performed autocorrelation analysis on the STMBJ traces of **C4** – **C16**, using the method developed by Makk *et al.*[30] that we have already implemented and described in some of our past publications.[31,32] In brief, each conductance trace is individually binned (50 bins per conductance decade) and

the correlation coefficient between the counts in each bin is evaluated and accumulated as a 2D array. The array is then plotted as a $Log(G/G_0)$ / $Log(G/G_0)$ heatmap. When two conductance features are correlated, they appear in the heatmap as off-axis features. Results of the autocorrelation analysis are shown in Supplementary Figure 84.

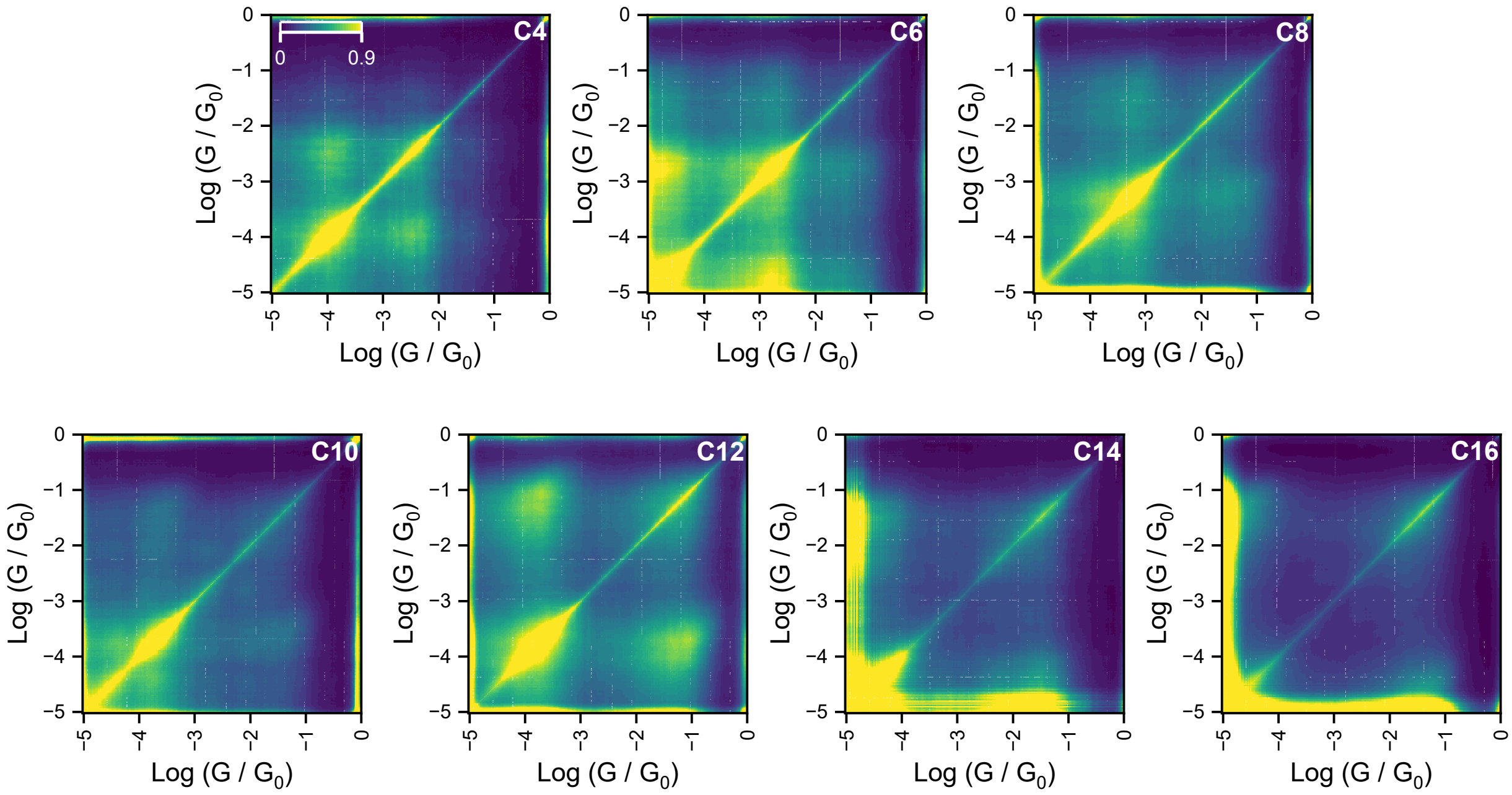

Supplementary Figure 84: Autocorrelation analysis for **C4** – **C16**. A common colour legend is shown in the plot pertaining to **C4**. All plots normalised to the number of traces in the dataset.

In all measurements showing multiple conductance peaks in the 1D histograms (**C4** – **C12**), strong off-axis features (correlation coefficient > 0.5) appear, confirming that multiple conductance plateaux are observed in each single trace. Analysis of **C14** shows some degree of off-axis correlation with a feature at conductance $G \cong 10^{-5} G_0$, evidence that dimer might be formed during withdrawal but they cannot be resolved sufficiently as they fall into the noise level of the instrumentation ($\sim 10^{-5}$ G_0 for these measurements taken with 10^5 V/A amplification). Autocorrelation analysis on **C16** (a species that returned a single conductance peak in the histogram) has no off-diagonal features, and the main conductance peak at $\sim 10^{-1.4} G_0$ is only correlated with the noise floor at 10^{-5} G_0.

Our data therefore suggest a junction evolution process such as that proposed in Supplementary Figure 85.

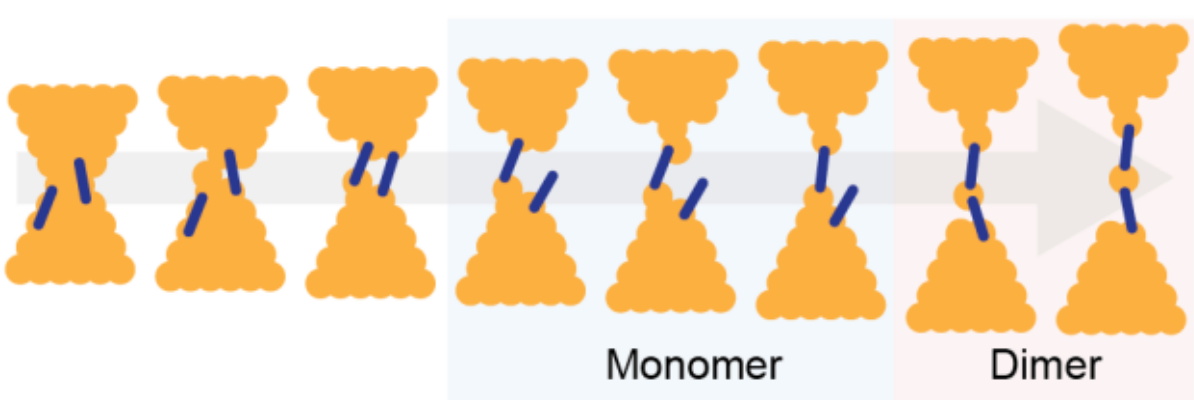

Supplementary Figure 85: Proposed junction evolution during an STMBJ experiments, with formation of monomeric junctions followed by Au extrusion and formation of dimeric Au-**Cn**-Au-**Cn**-Au junctions. Further withdrawal generates trimeric junctions.

To provide further evidence for the proposed formation of dimers and trimers, we performed concentration-dependent measurements on **C4**. Results can be seen in Supplementary Figure 86.

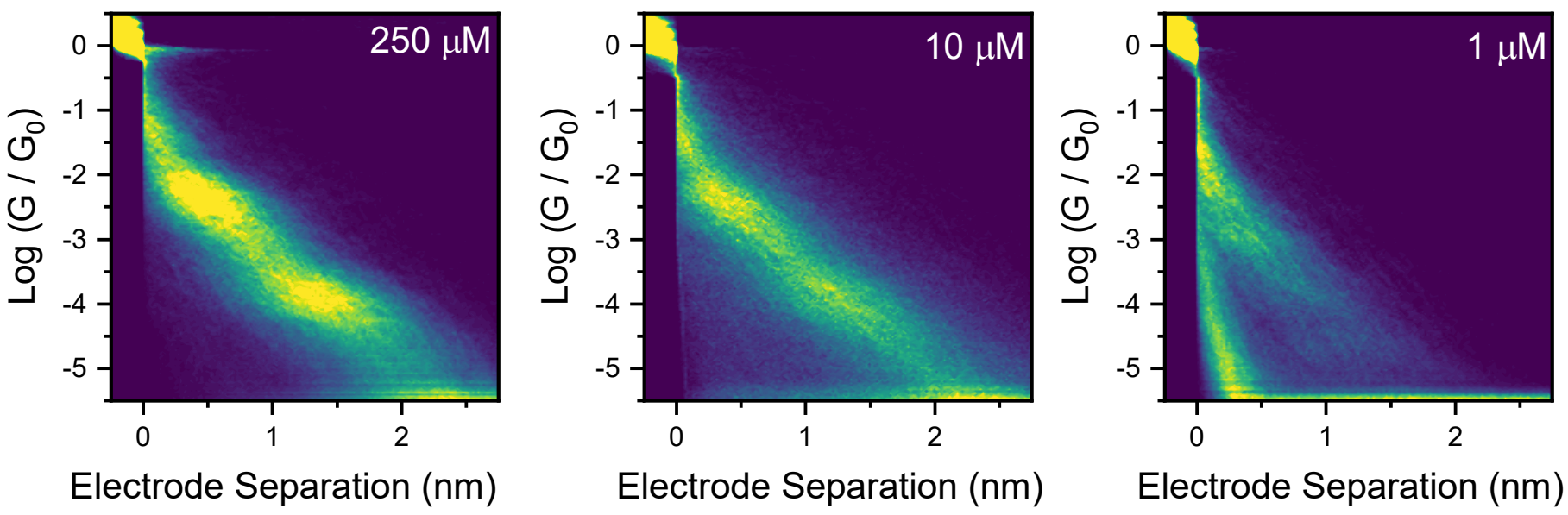

Supplementary Figure 86: 2D density maps for **C4** over a range of concentrations. Maps normalised to the number of traces used ($250\ \mu M$: 5139 traces $10\ \mu M$: 6343 traces; $1\ \mu M$: 4527 traces). All data acquired at $200\ mV$ bias.

"While the results at $10\ \mu M$ are similar to those obtained at higher concentration, at $1\ \mu M$ the only clear feature left is the monomeric junction at $\sim 10^{-1.5}\ G_0$, while the dimeric junction appears severely reduced in intensity, and the trimeric feature is almost completely absent, These concentration-dependent measurements are consistent with the proposed interpretation of multimeric junctions, as the population of multimeric junctions would be expected to increase with concentration"

2.4 Calculation of most probable conductance value

As discussed earlier, features overlap in the 1D histograms customarily used to calculate the most probable conductance value by Gaussian interpolation, resulting in the absence of clear peaks and generally complicating the extraction of numerical values. We used the 2D maps to extract such features, by isolating the various contributions depending on their electrode-electrode separation. A graphical description of the method we employed is shown in Supplementary Figure 87 and the values thus calculated are reported in Supplementary Table 5.

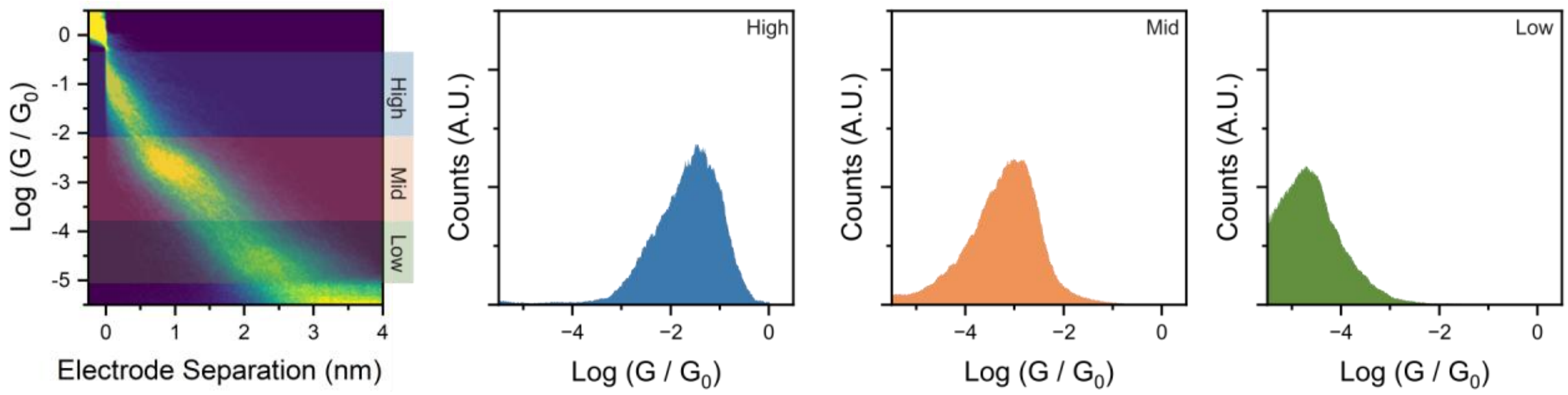

Supplementary Figure 87: Procedure for the extraction of numerical conductance values from the 2D density maps, using data from **C6** as example. The 2D map is sliced into sections depending on the electrode separation and histograms pertaining to each contribution are reconstructed. Gaussian fitting of the resulting peak gives the most probable conductance value and the standard deviation σ.

Supplementary Table 5: Conductance values for **C4 – C16** extracted from the 2D maps and used to generate the plots shown in the manuscript.

**Compound**	**High Conductance Monomer**	**Medium Conductance Dimer**	**Low Conductance Trimer**
**C4**	*not resolved*	$10^{-2.44\pm0.41}\ G_0$	$10^{-3.93\pm0.66}\ G_0$
**C6**	$10^{-1.67\pm0.56}\ G_0$	$10^{-2.77\pm0.62}\ G_0$	$10^{-4.81\pm0.60}\ G_0$
**C8**	$10^{-1.75\pm0.69}\ G_0$	$10^{-3.24\pm0.89}\ G_0$	*not observed*
**C10**	$10^{-1.91\pm0.61}\ G_0$	$10^{-3.57\pm0.72}\ G_0$	*not observed*

**C12**	$10^{-1.37\pm0.67}\ G_0$	$10^{-3.90\pm0.69}\ G_0$	*not observed*
**C14**	$10^{-1.42\pm0.59}\ G_0$	*not resolved*	*not observed*
**C16**	$10^{-1.41\pm0.55}\ G_0$	*not observed*	*not observed*

We used these data to plot our results in the manuscript. We report here the same data in a plot *vs* the end-to-end length of the molecular wire, and a comparison with the same literature results shown in the main manuscript:

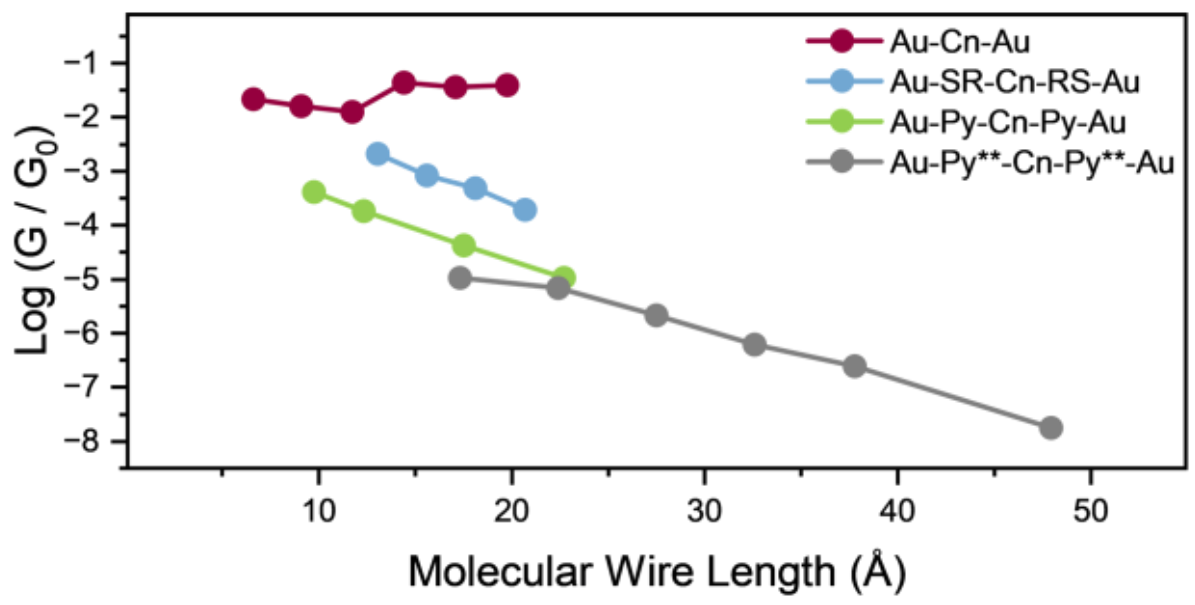

Supplementary Figure 88: Conductance *vs* molecular wire length for the species shown in Fig. 2f of the manuscript.

2.5 Additional Data Analysis

The distributions of break-off distance (maximum junction elongation) was directly evaluated from the raw datasets using an automated algorithm we described elsewhere.[28] Distributions are reported below and numerical values are collated in Supplementary Table 6.

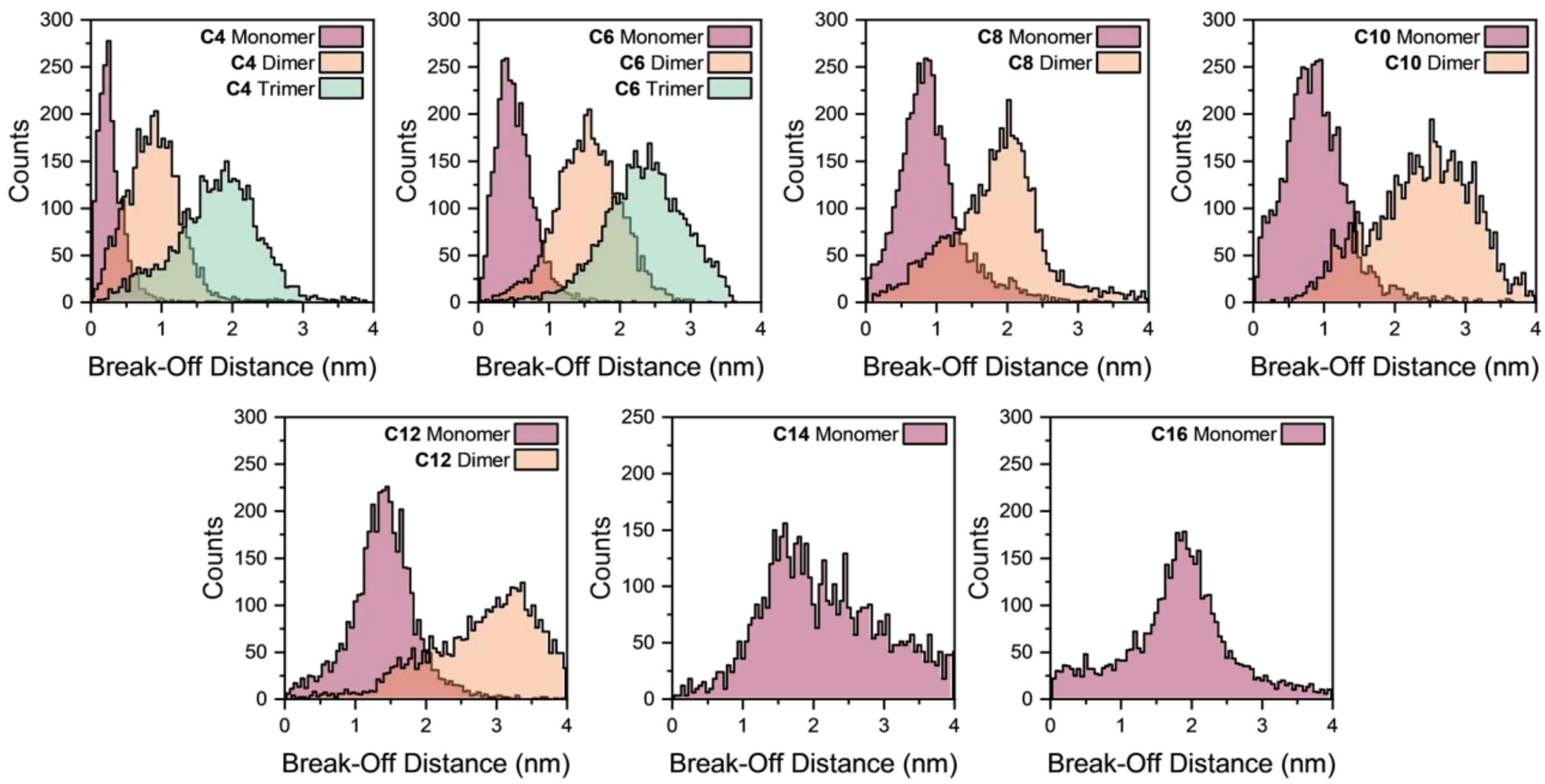

Supplementary Figure 89: Break-off distance distributions for **C4 – C16**. Histograms compiled with 20 bins per nanometre.

Supplementary Table 6: Gaussian fittings of the break-off distance histograms. Data are reported as $x_c \pm \sigma$. DFT distances are calculated from the structures (LDA-def2-SV(P) level of theory). Structures use a Au_2 cluster to connect dimers and trimers.

Compound	Monomer	Dimer	Trimer
**C4 EXP (DFT)**	$not\ resolved\ \ (0.38\ nm)$	$0.87\ \pm 0.37\ nm\ \ (1.16\ nm)$	$1.86\ \pm 0.53\ nm\ \ (1.95\ nm)$
**C6 EXP (DFT)**	$0.42\ \pm 0.27\ nm\ \ (0.66\ nm)$	$1.56\ \pm 0.42\ nm\ \ (1.68\ nm)$	$2.37 \pm 0.47\ nm\ \ (2.72\ nm)$
**C8 EXP (DFT)**	$0.80\ \pm 0.29\ nm\ \ (0.92\ nm)$	$2.01\ \pm 0.31\ nm\ \ (2.19\ nm)$	$not\ observed$
**C10 EXP (DFT)**	$0.91\ \pm 0.38\ nm\ \ (1.18\ nm)$	$2.49\ \pm 0.62\ nm\ \ (2.96\ nm)$	$not\ observed$
**C12 EXP (DFT)**	$1.39\ \pm 0.32\ nm\ \ (1.62\ nm)$	$3.15\ \pm 0.54\ nm\ \ (3.69\ nm)$	$not\ observed$
**C14 EXP (DFT)**	$1.66\ \pm 0.53\ nm\ \ (1.85\ nm)$	$not\ resolved$	$not\ observed$
**C16 EXP (DFT)**	$1.89\ \pm 0.34\ nm\ \ (2.14\ nm)$	$not\ observed$	$not\ observed$

As can be seen in Supplementary Table 6, the trends in break-off distances are consistent and well-commensurate with the theoretical C…C distances calculated by DFT. The difference between experimental and theoretical values (the latter calculated assuming a $2Å$ C-Au bond and using the relaxed distance between terminal C atoms) qualitatively suggests a relatively short "snapback" of the electrodes, with an average Δx of $\sim 0.2 - 0.3\ nm$. This is consistent with phenomena already reported in the literature: strong adsorbates on the Au electrodes reduce the free energy of surface atoms, limiting their mobility and enabling the fabrication of more robust apical structures, with the final result of a shorter "snapback" upon rupture of the Au atomic contact.[28,33–36]

We used these break-off distances to re-calculate the attenuation plot at the most probable junction length, and at 80% extension (to ensure minimal stretching that is predicted to increase the BLA[37]), extracting the relevant value from the 2D density maps. Results can be seen below:

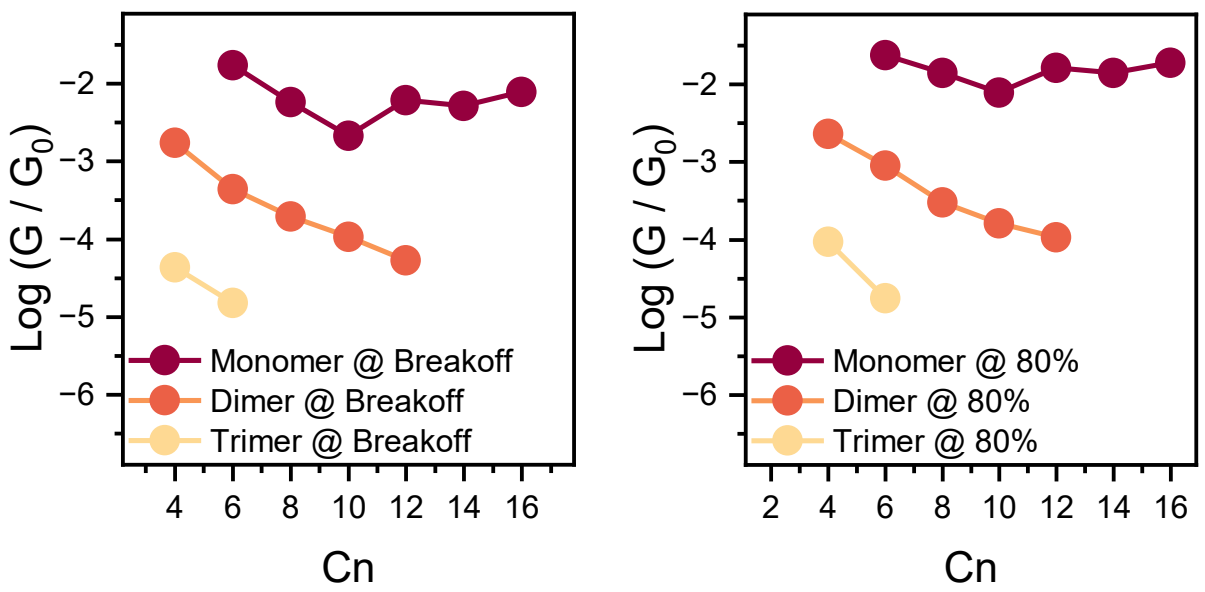

Supplementary Figure 90: Conductance at break-off length (*i.e.* centre of the Gaussian distribution) *vs* number of sp carbons in the **Cn** chain (left), and conductance at 80% junction elongation *vs* number of sp carbons in the **Cn** chain (right).

The results are broadly similar to those shown in the manuscript (where the full junction evolution was considered to calculate the conductance) although the absolute conductance values are – in both cases – slightly lower. Nevertheless, the monomeric junctions show a non-monotonic decay in both cases, again with **C12** showing higher charge-transport efficiency than **C10**, and the values of conductance plateauing off (within the confidence interval) in **C14** and **C16**. The dimeric junctions, on the other hand, show monotonic decay, again with quite similar coefficients ($\cong 0.2$ per carbon).

To further analyse these phenomena we calculated the distribution of the plateau region slopes (identified by the same algorithm[32] used to calculate the break-off distance) with the aim of understanding if different

molecular structure (*e.g.* monomer or dimers) yield different junction evolution processes. We only did this analysis for the monomeric and dimeric junctions, as the trimeric junctions would only yield two datapoints (**C4** and **C6**), and we avoided such analysis in **C4** due to challenges in reliably isolating the monomeric junctions (break-off too short).

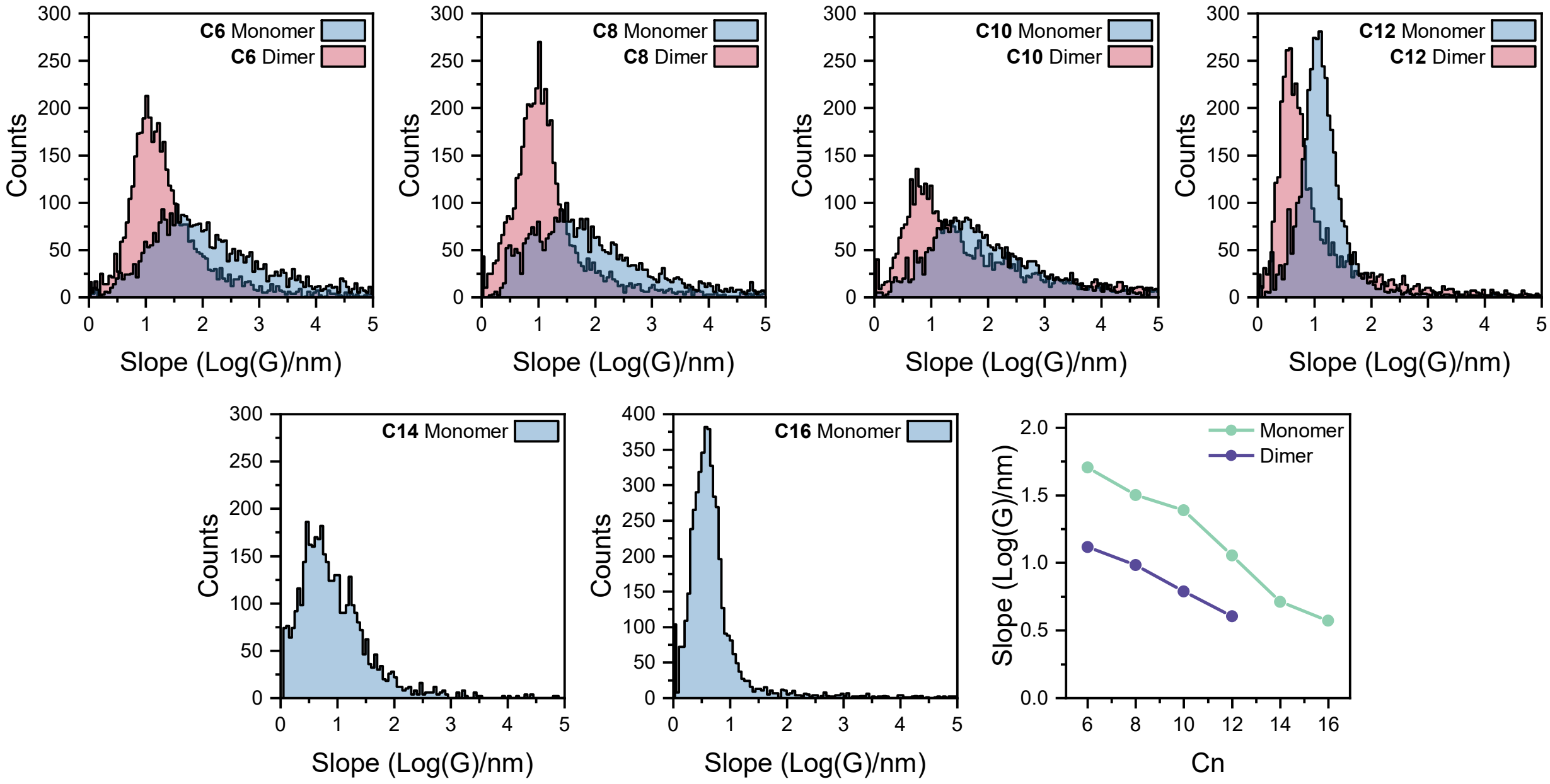

Supplementary Figure 91: Distributions of the slope of the plateau region of the STMBJ traces for compounds **C6-C16.**

In all cases the monomeric junctions deliver more sloped plateaux than the corresponding dimeric ones. We attribute this to the decreased conformational rigidity in the dimers – linked by Au atoms/clusters – that reduces electromechanical coupling effects. The slope of both monomeric and dimeric features decreases across the series as stretching a longer chain by a set amount has a less pronounced effect than stretching a shorter chain by the same amount.

Finally, we used the extracted conductance and break-off distances to re-fit our full histograms and verify the accuracy of the values obtained. The results for **C4-C12** (**C14** and **C16** only show a single peak in the histograms) can be seen below, along with the conductance attenuation plot obtained from the Gaussian fittings. Using this method, we were able to calculate a conductance value for the monomeric **C4**, initialising the Gaussian fit with the values for the dimer and trimer extracted as before, and leaving the algorithm to identify a third, monomeric peak. Given the possibility of over-fitting data, we rely in the manuscript with the data calculated with the simpler method outlined earlier, as the results are essentially the same.

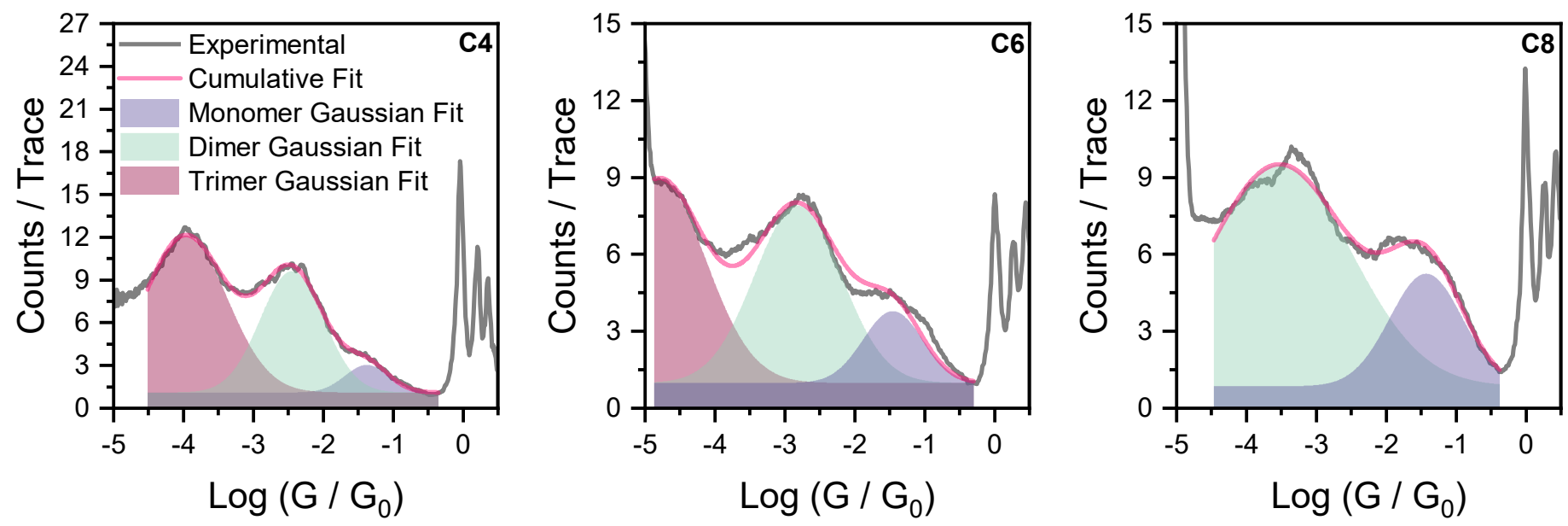

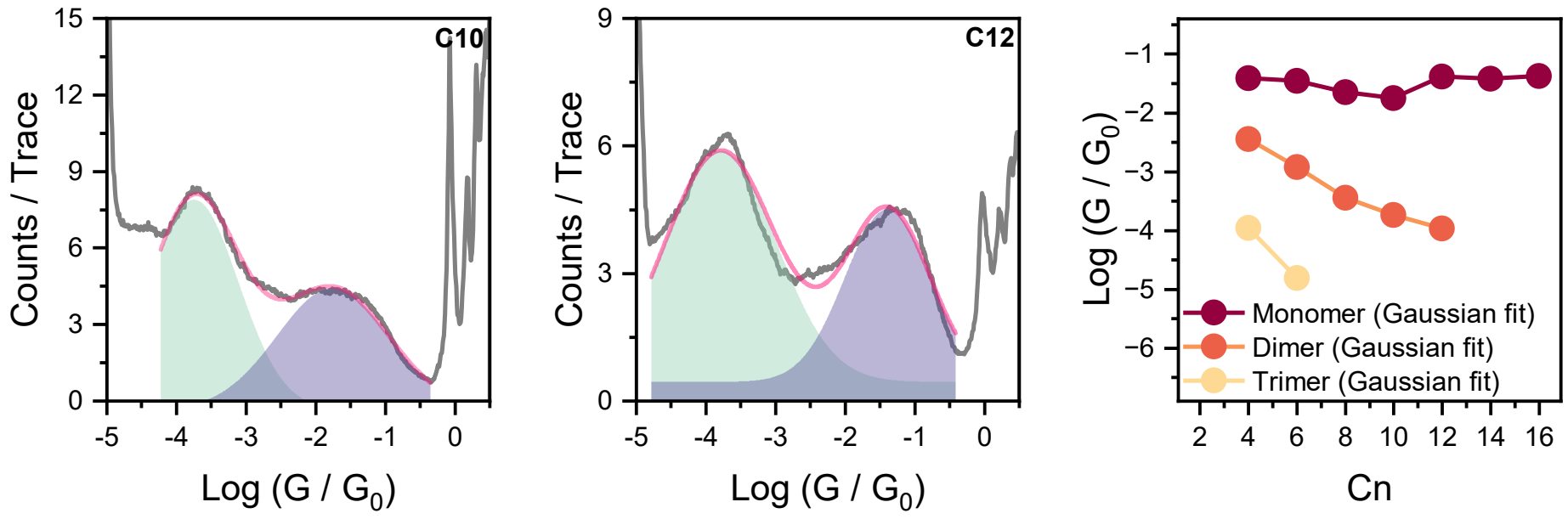

Supplementary Figure 92: Multi-peak Gaussian fitting of the histograms containing multiple conductance signatures. Fittings were initialised with the conductance values calculated with the method explained earlier, and the algorithm was left to converge to a Chi-squared tolerance of 10^{-9}.

2.6 Additional Control Experiments

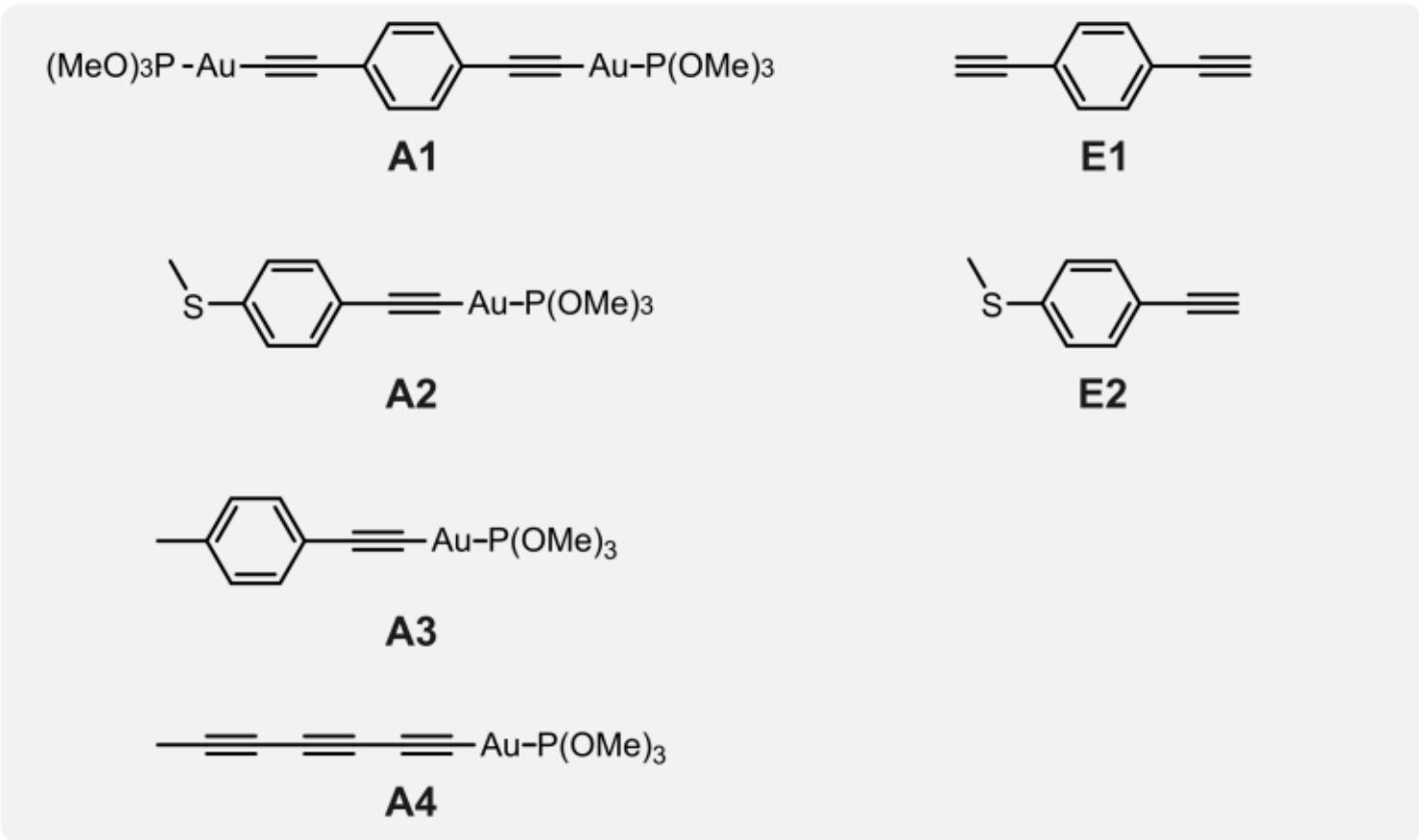

Supplementary Figure 93: Structures of the compounds used as control experiments.

To further test the ability of our AuP(OMe)$_3$-capped alkynes to form stable junctions on Au electrodes, we performed *STMBJ* experiments on compounds **A1** and **A2**, their free ethynyl derivatives **E1** and **E2**, and the compounds with only one viable metallophilic terminus **A3** and **A4.** (structures in Supplementary Figure 93). Details on the preparation of **A1**-**A4** can be found earlier in this document. **E1** was purchased from TCI UK and purified by column chromatography on silica before use. The synthesis of **E2** is described elsewhere.[32] Comparison of the results of measurements on compounds **A1** and **E1** immediately shows the superiority of the transmetallation approach to junction fabrication, as can be observed in Supplementary Figure 94 and Supplementary Figure 95. In this case, the presence of Au(I) trimethylphosphites on the two ethynyl ends of the molecular wire **A1** returns much higher junction formation probability (respectively, ~88% for **A1** and ~47% for **E1**) and more mechanically stable junctions, able to be stretched to the full -C≡C-C_6H_4-C≡C- length and, similar to what has already been observed for **C4-C12**, formation of dimeric junctions at larger electrode separations.

The formation of dimeric junctions, however, appears to be limited to compounds having Au(I) phosphites on both termini of the molecular wire. The 4-ethynylthioanisole derivatives **A2** and **E2** only show a single contribution to the 2D density map (Supplementary Figure 96 and Supplementary Figure 97), with little evidence of stable molecular junctions at electrode separation exceeding the length of the molecule itself. The

model of junction evolution discussed in the manuscript explains this behaviour, as mechanically strong interfaces at both end (e.g. covalent Au-C bonds rather than coordinative Au-S interactions) are required for the extrusion process to happen consistently. Nevertheless, the almost perfect match in the high conductance feature in the comparison of **A2** and **E2** confirms a transmetallation **X-**AuP(OMe)$_3$ → **X**-Au(0)$_{ELECTRODE}$ process underpinning junction assembly.

A3 and **A4**, on the other hand, were used to verify that the Au(**I**)trialkylphosphite does not contribute to any feature in the STMBJ experiments upon transmetallation of the acetylenic carbon to the Au(0) electrodes. in both cases, while the 1D histograms show some features in the range $10^{-4} - 10^{-3} G_0$, the 2D density map shows that these are associated with break-off distances of $0.5 - 0.6\ nm$ (not accounting for electrode snapback), longer than the C-C distance of these molecular wires assuming they assemble into junctions exploiting π-Au interactions. These features are also lower in intensity (compare the >10 counts/trace of **C4-C6** vs the ~ 2 counts/trace for **A3** and **A4**) and therefore most probably due to π-stacked dimers, and their relatively low transport efficiency confirms that the high-conductance features observed in the experiments on **C4-C12** are due to transport through the extended carbon chain."

All experiments below have been performed in anisole, at 250 μM concentration of the target molecular wire, with $200\ mV$ bias. All histograms and 2D maps have been normalised to the number of traces used to compile them.

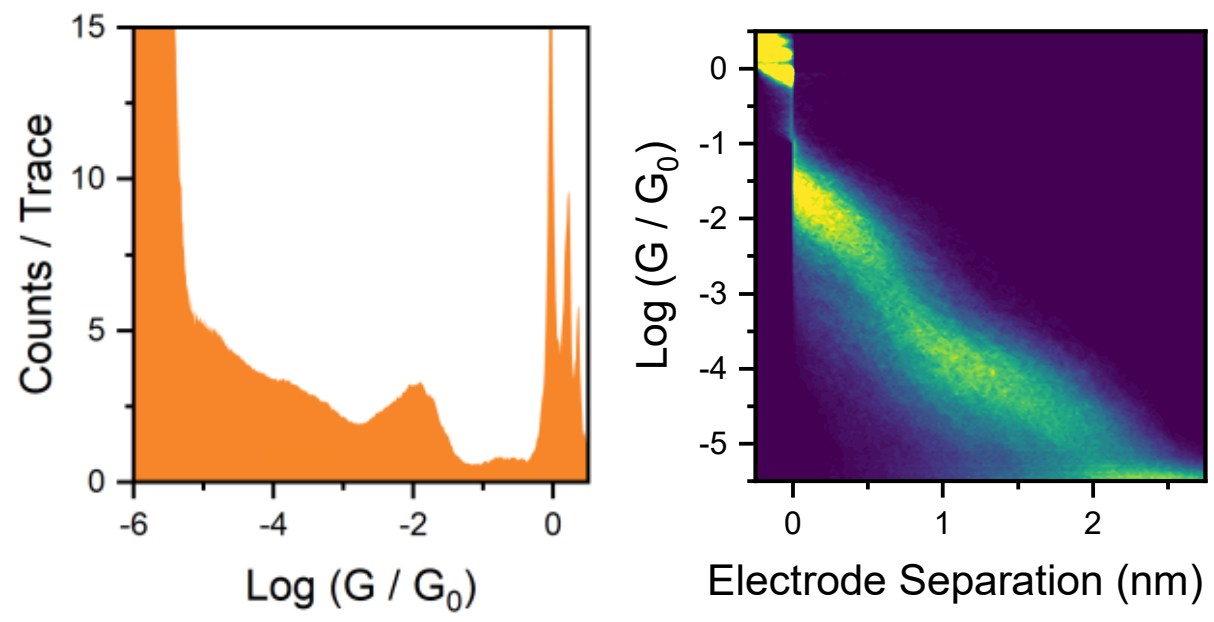

Supplementary Figure 94: Conductance histogram and density map for **A1**. The low-conductance feature (dimeric junction) is not clear in the histogram but appears as a well-defined density "cloud" in the 2D plot.

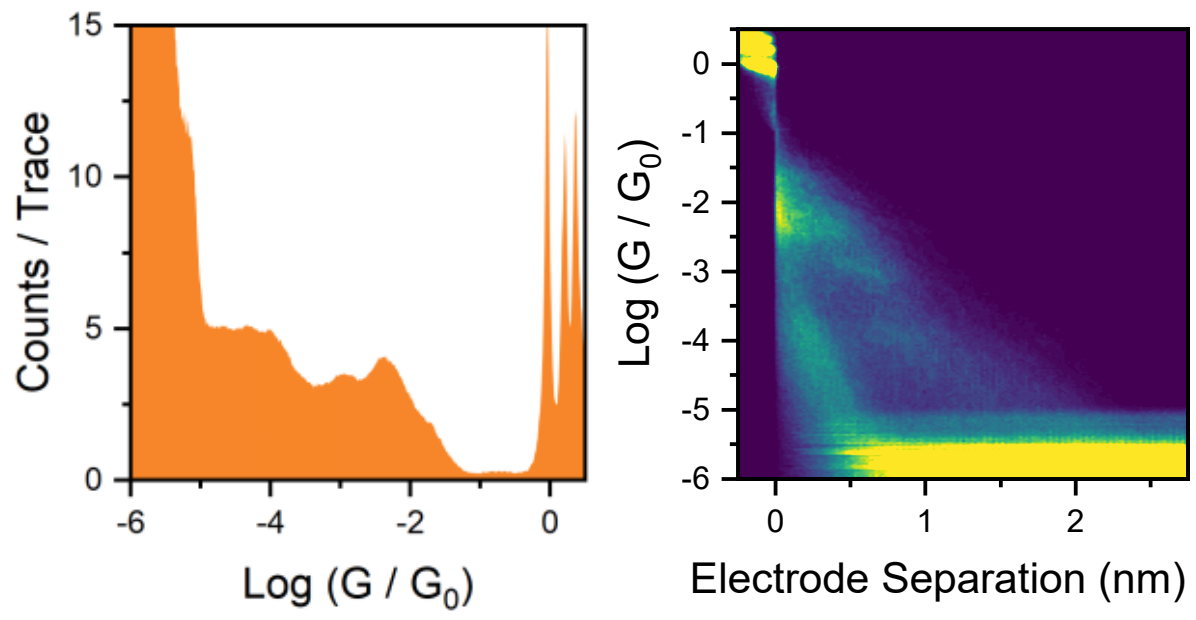

Supplementary Figure 95: Conductance histogram and density map for **E1**.

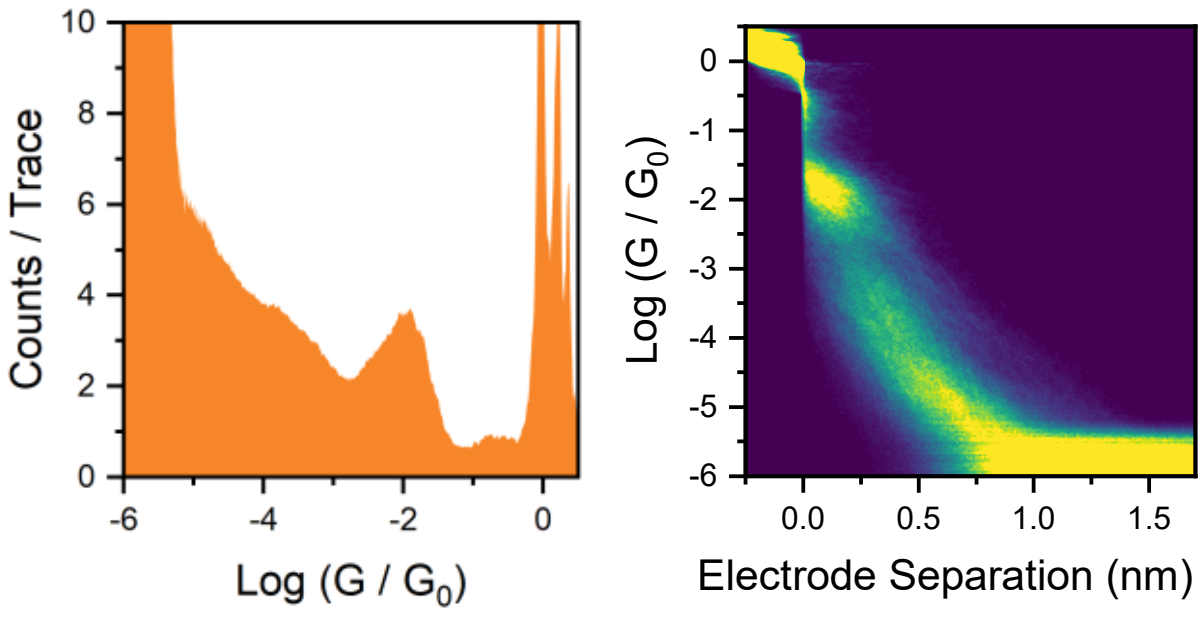

Supplementary Figure 96: Conductance histogram and density map for **A2**.

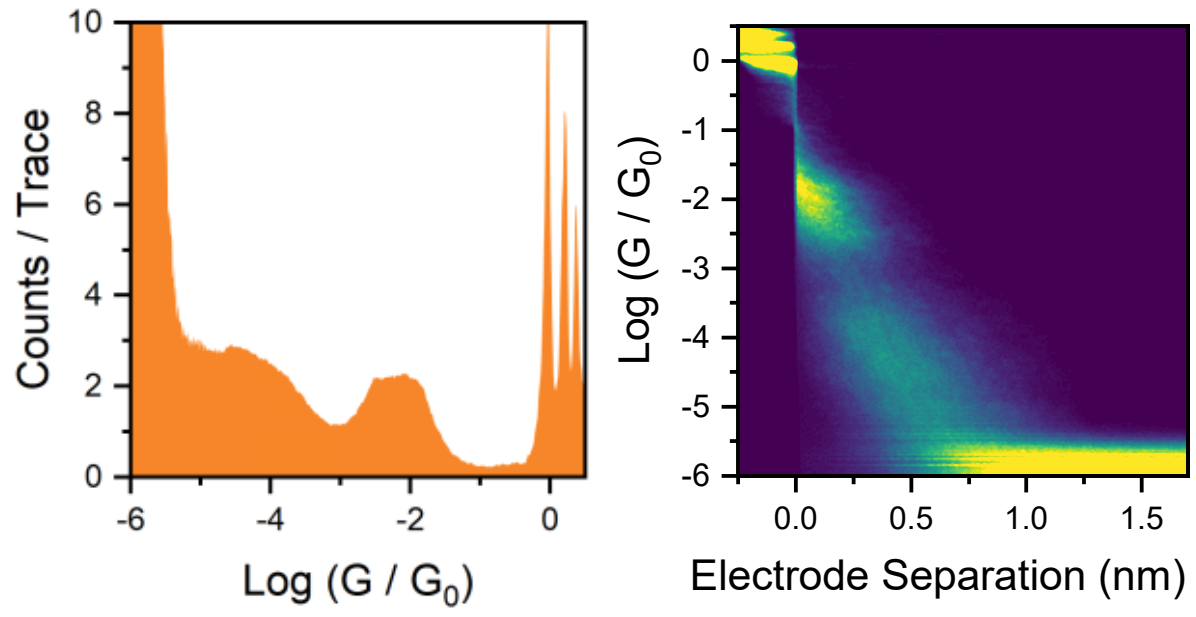

Supplementary Figure 97: Conductance histogram and density map for **E2**.

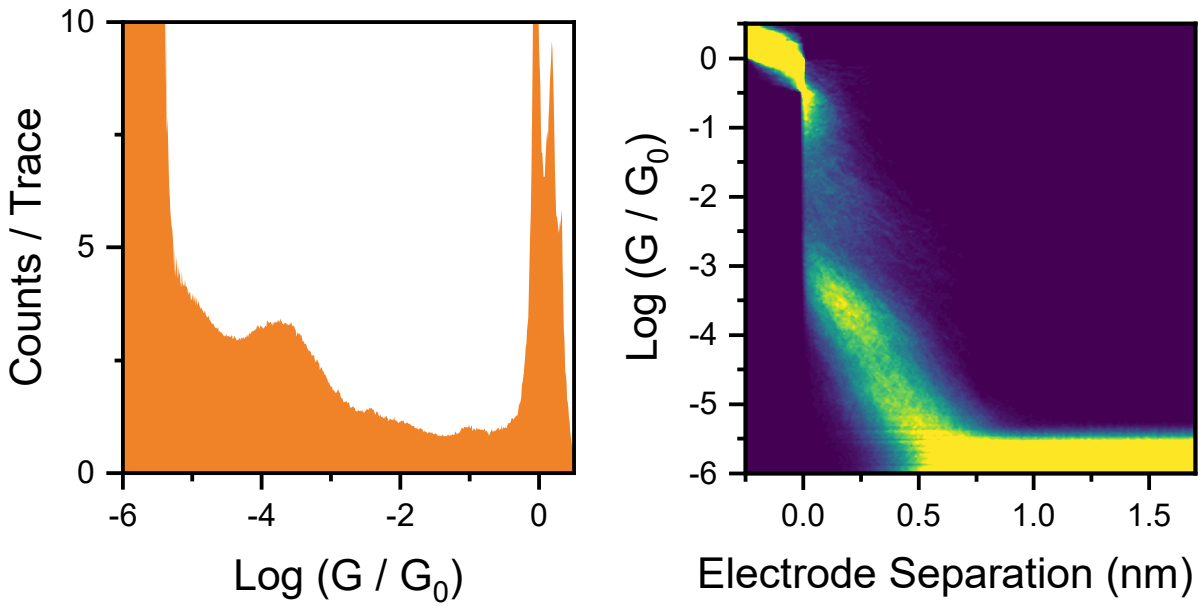

Supplementary Figure 98: Conductance histogram and density map for **A3**.

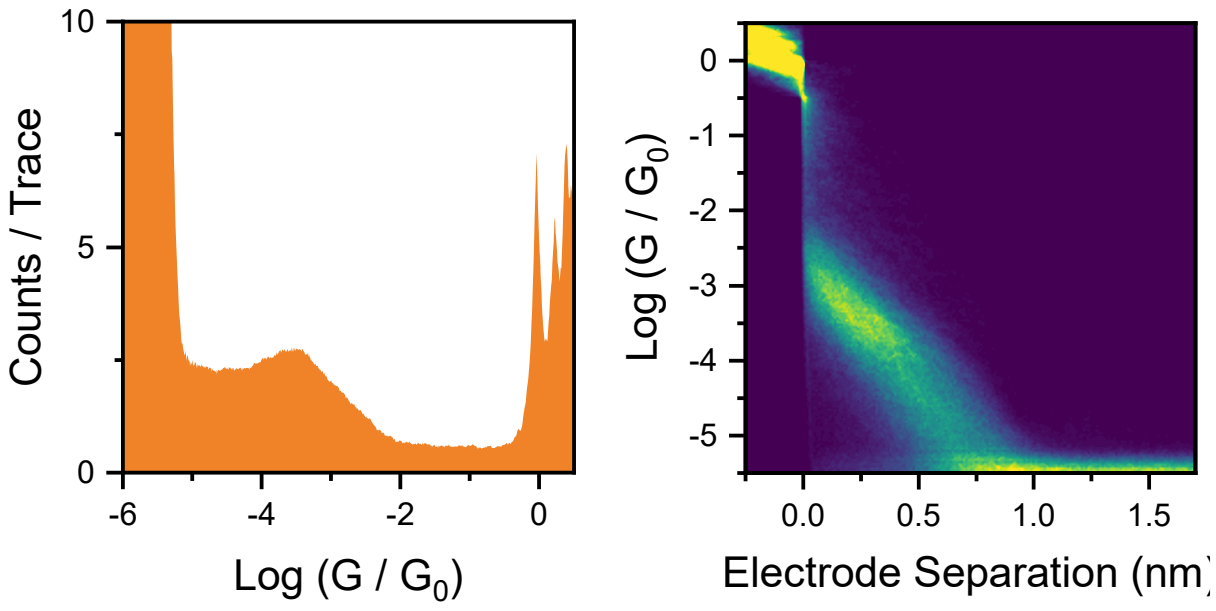

Supplementary Figure 99: Conductance histogram and density map for **A4**.

2.7 $I - V$ Measurements

To measure the $I - V$ characteristics, we used a modified STMBJ technique we have already described in our previous contributions.[25,26,38] In brief, the protocol is the same applied to STMBJ experiments, but the signals applied to the piezoelectric transducer and to the junction bias are different. We applied a staircase ramp to

the piezoelectric actuator, alternating abrupt steps of height commensurate to the junction length measured with regular STMBJ measurements, and "hold" portions of $50 - 100\ ms$. During the "hold", the bias is held at a DC bias of $200\ mV$ for $10\ ms$, linearly ramped between $\pm 1.3\ V$ at a rate of $17.5 - 46.7\ V\ s^{-1}$, and then returned to the DC bias of $200\ mV$ for the remaining $10\ ms$. Data are then processed and analysed using an automated algorithm. The algorithm first slices the traces into individual steps by cutting where the derivative of the piezo signal goes above a threshold to isolate single $I - V$ traces. After that, the algorithm averages the conductance of the DC hold portions of the ramp and only selects traces where its value falls within one standard deviation from the most probable conductance determined by STMBJ measurements. Through this process, the algorithm filters out traces where the junction is metallic (*e.g.* Au-Au), the junction is empty (*e.g.* tunnelling), the junction did not survive the entire bias modulation process, or the junction is not monomeric. This process resulted in a retention rate >35% for all species measured.

We report below all $I - V$ density maps (in the range $\pm 1\ V$ to avoid attenuation of the applied bias by the resistor in series) for the compound measured in this study.

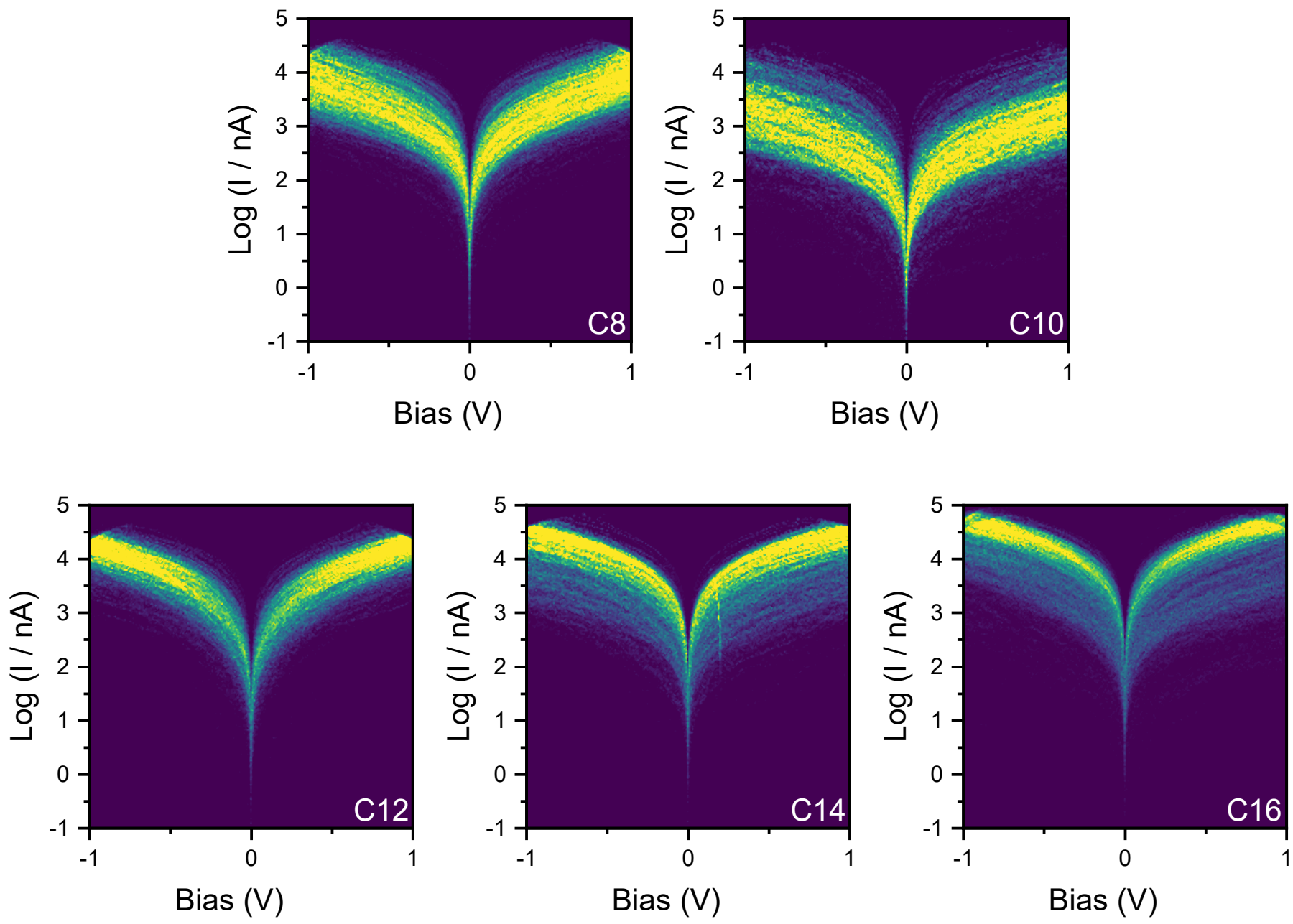

Supplementary Figure 100: $I - V$ density maps for **C8 – C16**. All data compiled with 100 bins per V and 100 bins per current decade. The small feature at 0.2 V in the $I - V$ map for **C14** is an artifact due to a few datapoints pertaining to the "hold" portion of the bias ramp being retained by our data analysis algorithm.

We used the $I - V$ data to calculate the conductance *vs* number of carbon trends for the series across a range of biases, to verify that the behaviour observed in the static *STMBJ* experiments is retained at sweeping bias conditions and, especially, at low bias where possible resonant transport effects[39] should not be dominant.. Results can be found below:

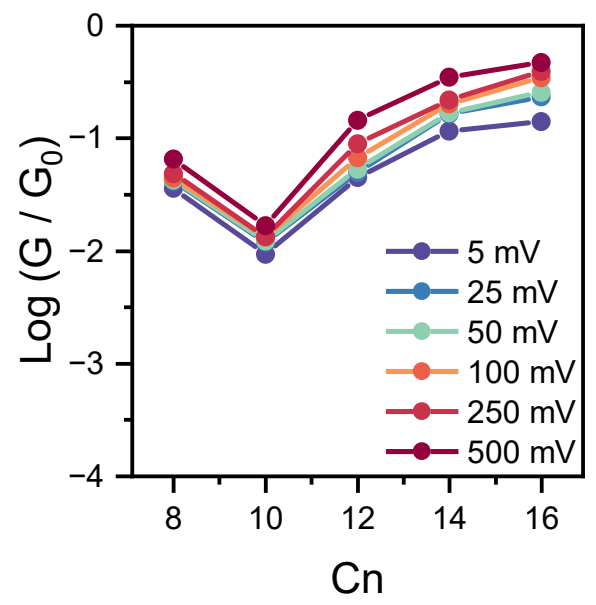

Supplementary Figure 101: Conductance *vs* number of sp carbons in the **C8** – **C16** range extracted from the $I-V$ curves. At all bias voltages probed ($5\ mV - 500\ mV$), conductance follows the same behaviour observed in the static *STMBJ* experiments performed at $200\ mV$.

2.8 STMBJ and $I-V$ Statistics

We report below the size of the datasets (# of traces) used to compile the histograms and 2D plot shown here and in the manuscript.

Supplementary Table 7: Dataset sizes for the STM-based experiments performed. $I-V$ datasets are reported before and after data selection, using the protocols described earlier.

**Compound**	**STMBJ**<br>**# of traces**	**$I-V$ Measurements**<br>**# of traces selected / full dataset**
**C4**	5139	--
**C6**	5576	--
**C8**	5984	2978 / 5408
**C10**	5405	2306 / 5069
**C12**	5223	3666 / 3944
**C14**	5711	1642 / 4557
**C16**	5762	2537 / 7151
**A1**	5092	--
**A2**	5019	--
**A3**	6216	--
**A4**	5559	--
**E1**	4903	--
**E2**	6548	--

3. Raman & SERS Spectroscopy

Powder and SERS measurements were performed using a Renishaw inVia Qontor confocal Raman microscope ($785\ nm$, $\sim 0.15\ mW$). Au nanoparticles (AuNPs) were prepared using a literature procedure based on Turkevich's method by rapid addition of sodium citrate ($34.3\ \mu mol$, $0.7\ mL$) to a solution of refluxed ($30\ min$) $HAuCl_4$ ($29.4\ \mu mol$, $100\ mL$). The resulting colloid was centrifuged (297 minutes, 4000 rpm), the supernatant removed, and the concentrated AuNP suspension deposited onto clean silicon wafers by drop-casting. The substrate was then left to dry under vacuum overnight. SERS samples were produced by drop casting a solution of the target compound ($0.25\ mM$, acetone) onto the silicon/AuNP substrate. The samples were then washed with fresh acetone and left to dry in a desiccator before spectral measurement.

3.1 Additional Raman Spectra

In addition to the narrow range spectra shown in the manuscript, we report below the full spectra acquired on the **1(n)** powder precursors and on the corresponding **Cn** SERS substrates, including also the butadiynyl derivative **1(4)** and **C4** (not discussed in the manuscript).

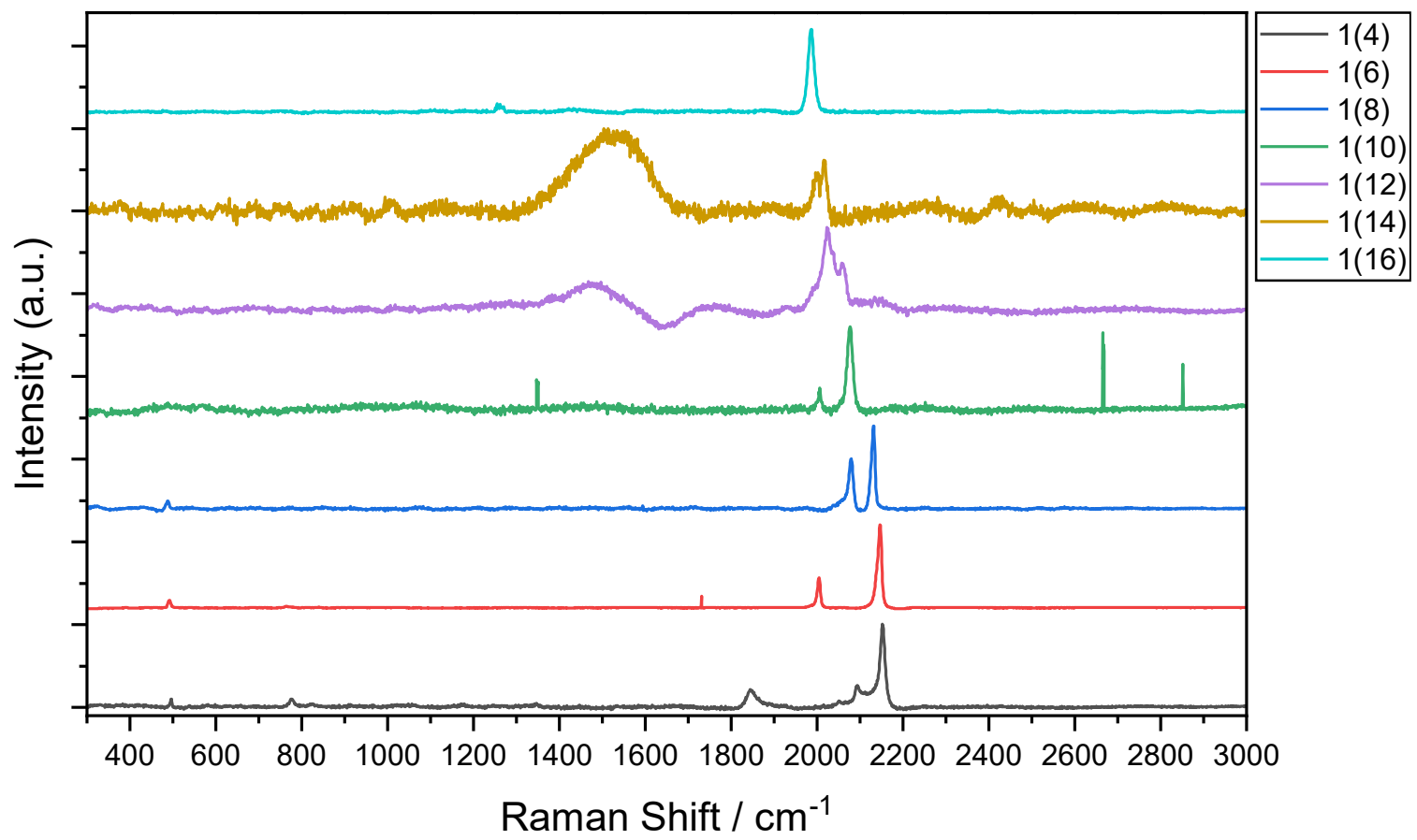

Supplementary Figure 102: Full-range ($300 - 3000\ cm^{-1}$) Raman spectra for powder samples of **1(4) – 1(16)**

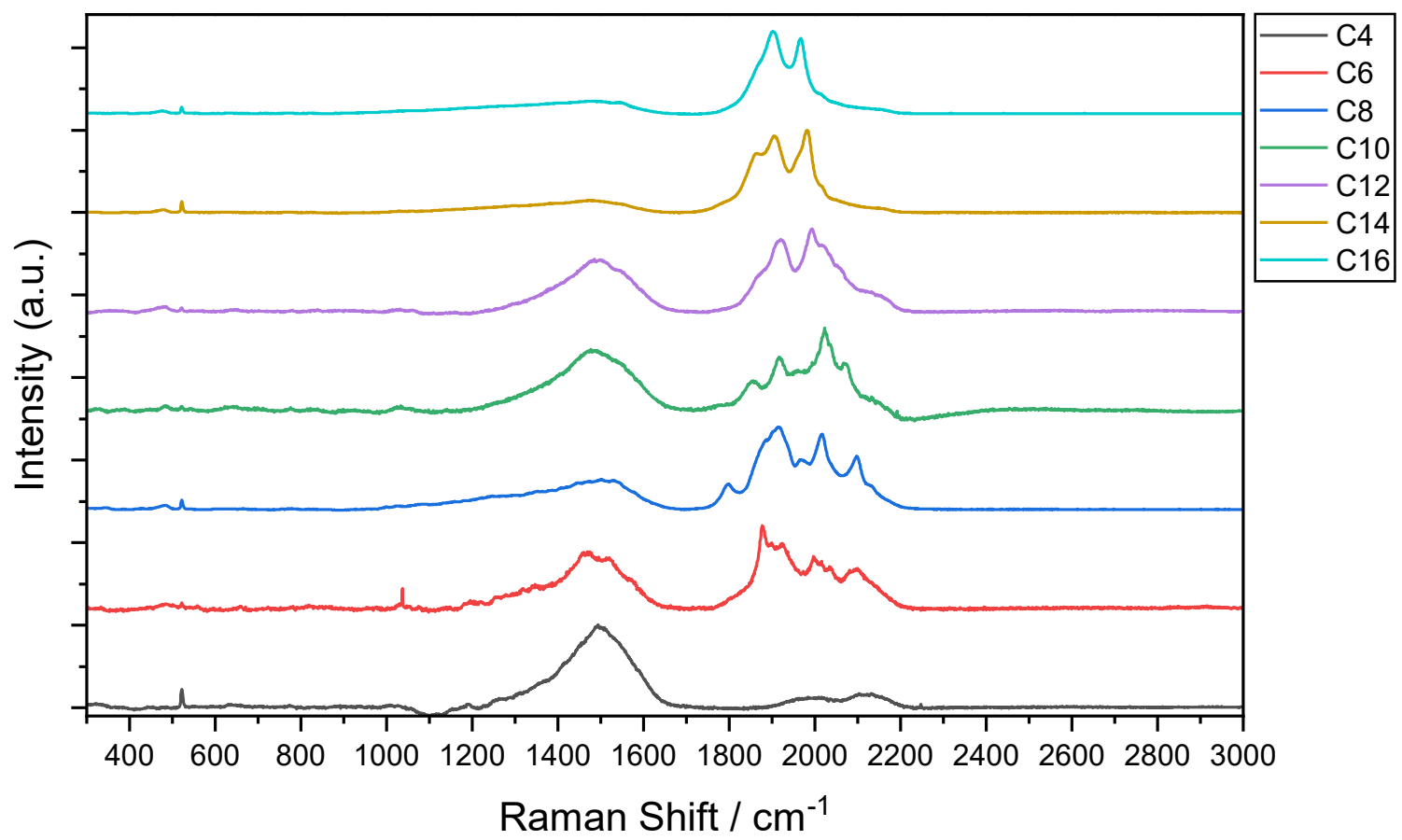

Supplementary Figure 103: Full-range ($300 - 3000\ cm^{-1}$) SERS spectra for **C4 – C16**

As can be observed below, a small signal attributable to the Au-C stretching appears in the SERS spectra at $\sim 480\ cm^{-1}$, a signal not visible in the **1(n)** precursors or in the bare AuNP/Si spectrum. This signal confirms transmetallation of the precursors to the Au nanoparticles.

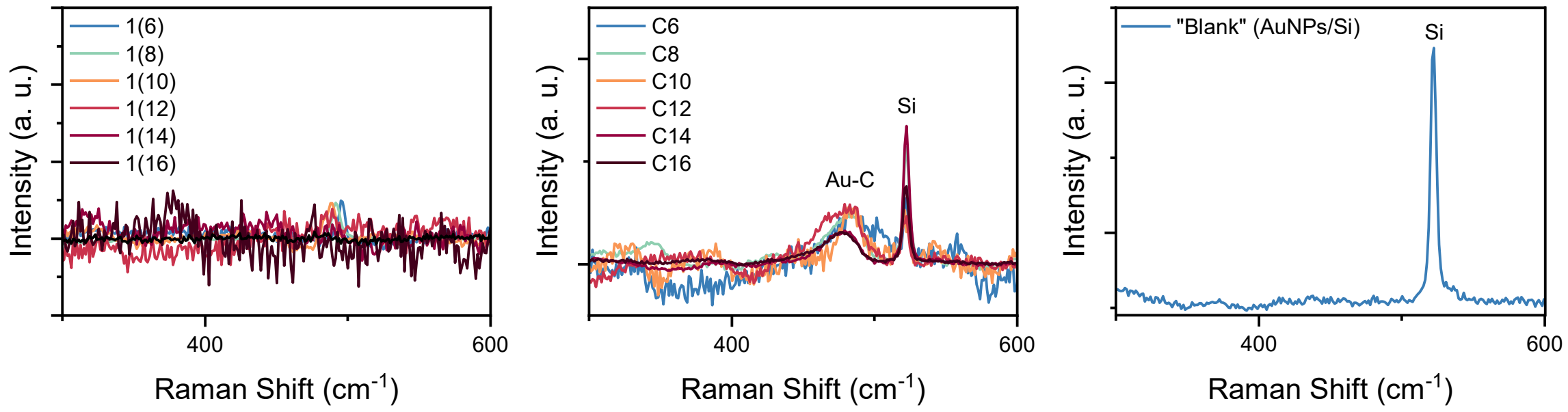

Supplementary Figure 104: Close-up of the $300 - 600\ cm^{-1}$ region for powder Raman (left) and SERS (centre). A blank spectrum of the bare AuNPs on Si is provided as reference (right).

4. Details on Tight-Binding (Hückel) Calculations

A custom tight-binding (Hückel) code (the *Tight-Binding Funtime Engine v1.0a*) was used to determine all zero bias transmission functions used in this paper. To summarise, the model uses the analytical expression for each pair of 1D electrode surface Green's functions $\hat{g}_s(E)$ (internal hopping $t_{Au-Au} = -1.1\ eV$ and on-site energy $\varepsilon_{Au} = 0\ eV$) under the nearest neighbour approximation and overlap integrals $S = \delta_{ij}$. Inside the 1D band $g_s(E) = \frac{E}{2t^2} - i\frac{1}{|t|}\sqrt{1-\left(\frac{E}{2t}\right)^2}$ and $\hat{g}_s(E)$ is a sparse matrix with $g_s(E)$ at the atomic contact site. These electrodes are coupled to the molecule (represented by a Hückel Hamiltonian $\hat{H}_m$, the parameters of which can be found in the manuscript) through self-energy matrices for the left and right electrodes ($\hat{\Sigma}_L = \hat{\Sigma}_R = t^2_{Au-C}\hat{g}_s(E)$) which are determined from $\hat{g}_s(E)$ and the Au-C hopping integral $t_{Au-C} = -0.4\ eV$. Finally the transmission function can be determined analogously to DFT methods using $T(E) = Trace(\hat{\Gamma}_L(E)\hat{G}_m(E)\hat{\Gamma}_R(E)\hat{G}_m^\dagger(E))$ where $\hat{G}_m = \left(E\hat{I} - \hat{H}_m - \hat{\Sigma}_L(E) - \hat{\Sigma}_R(E)\right)^{-1}$ is the retarded Green's function of the full system and $\hat{\Gamma}_{L,R}(E) = -i(\hat{\Sigma}_{L,R}(E) - \hat{\Sigma}^\dagger_{L,R}(E))$ describes the level broadening as a result of the coupling to the electrodes. For computational efficiency, the imaginary component of $g_s(E)$ is obtained prior to construction of $\hat{\Gamma}_{L,R}(E)$ and the trace is multiplied by 4 to obtain $T(E)$.

As discussed in the manuscript, the 'base' term for the carbon to carbon hopping integrals was set to $t = -1.5\ eV$ and modified for the single (α) and triple (β) bonds by the bond order BO of the compounds extended p-orbital system to give $\alpha = t \times (1 - BO)$ and $\beta = t \times BO$ where BO is determined from the infinite polyyne band structure for the case of the oligoynyl wires and then modified between 0.55 and 0.5 to explain the increase in conductance. We note that the exact values of these hoppings are not accurate enough obtain quantitative values. However, as all the base hoppings are kept constant throughout, it is possible to obtain a qualitative description of the series.

We expect that the dimer junctions will have a variable central gold cluster size. However, as the cluster's internal transport is expected to be ballistic, the size of the gold extrusion has little to no effect on the dimer conductance and so we chose to model the nanocluster as an Au dimer (the simplest closed shell system) with the same internal and Au-C hopping terms as described above. Doing so effectively creates a defect representative of the cluster in the experimental junctions without the need for added complexity.

In order to model BLA, we can use a simple model for the local bond order of the triple (β) and single (α) bonds:

$$BO_\beta = \frac{\beta}{\alpha + \beta}$$

$$BO_\alpha = \frac{\alpha}{\alpha + \beta}$$

Introducing Peierls' distortion factors to introduce BLA in the carbon chain as:

$$f(d) = C_i \exp\left(-\eta_i d_i\right)$$

$$\beta(d) = C_\beta \exp\left(-\eta_\beta d_\beta\right)$$

$$\alpha(d) = C_\alpha \exp\left(-\eta_\alpha d_\alpha\right)$$

and the average bond length and BLA values as:

$$d_{avg} = \frac{d_\alpha + d_\beta}{2}$$

$$BLA = d_\alpha - d_\beta$$

Allows the individual bond lengths to be expressed in terms of d_{avg} and BLA:

$$d_\beta = d_{avg} - \frac{BLA}{2}$$

$$d_\alpha = d_{avg} + \frac{BLA}{2}$$

From which, the resonance integrals can be expressed in terms of the BLA to give:

$$\beta = C_\beta \exp\left(-\eta_\beta\left(d_{avg} - \frac{BLA}{2}\right)\right) = C_\beta \exp\left(-\eta_\beta(d_{avg})\right) \exp\left(\eta_\beta \frac{BLA}{2}\right)$$

$$\alpha = C_\alpha \exp\left(-\eta_\alpha\left(d_{avg} + \frac{BLA}{2}\right)\right) = C_\alpha \exp\left(-\eta_\alpha(d_{avg})\right) \exp\left(-\eta_\alpha \frac{BLA}{2}\right)$$

Defining resonance integrals for the average bond length using

$$\beta_{avg} = C_\beta \exp\left(-\eta_\beta(d_{avg})\right)$$

And

$$\alpha_{avg} = C_\alpha \exp\left(-\eta_\alpha(d_{avg})\right)$$

Leads to

$$\beta = \beta_{avg} \exp\left(\eta_\beta \frac{BLA}{2}\right)$$

$$\alpha = \alpha_{avg} \exp\left(-\eta_\alpha \frac{BLA}{2}\right)$$

Which, upon inserting into the bond order relation yields:

$$Bond\ Order = \frac{\beta}{\alpha + \beta} = \frac{\beta_{avg} \exp\left(\eta_\beta \frac{BLA}{2}\right)}{\beta_{avg} \exp\left(\eta_\beta \frac{BLA}{2}\right) + \alpha_{avg} \exp\left(-\eta_\alpha \frac{BLA}{2}\right)}$$

Simplifying the expression leads to

$$Bond\ Order = \frac{1}{1 + \frac{\alpha_{avg}}{\beta_{avg}} \exp\left(-\left(\frac{\eta_\beta + \eta_\alpha}{2}\right) BLA\right)}$$

Which further simplifies to

$$Bond\ Order = \frac{1}{1 + \frac{C_\alpha}{C_\beta} \exp(-\Delta\eta d_{avg}) \exp\left(-\eta_{avg} BLA\right)}$$

Where $\Delta\eta = \eta_\beta - \eta_\alpha$ and $\eta_{avg} = (\eta_\beta + \eta_\alpha)/2$. For bonds with equivalent atoms (such as CC), $\eta_\beta = \eta_\alpha = \eta$ and the expression simplifies even further to:

$$Bond\ Order = \frac{1}{1 + \frac{C_\alpha}{C_\beta}\exp(-\eta BLA)}$$

The coefficients $C_{\alpha/\beta}$ can be obtained from the polynomial prefactor of the overlap integral between the p_z-orbitals $S_{p\pi}$ under Mulliken's approximation which results in:[40]

$$C_i = 1 + \eta_i d_i + \frac{2}{5}(\eta_i d_i)^2 + \frac{1}{15}(\eta_i d_i)^3$$

For $i = \alpha, \beta$, where d_α and d_β are as defined above.

This shows the equivalence of our bond order method and a Peierls distortion factor method, with the former requiring fewer parameterisations and being linear with respect to the resonance integrals for the single (α) and triple (β) bonds. The resulting relationship is demonstrated in Supplementary Figure 105 for $\eta = 29.6\ nm^{-1}$ (estimated from the Clementi-Raimondi orbital exponent[41] for the C 2p orbital), $d_{avg} = 0.137\ nm$,.

Likewise, the tunnelling decay rate with repeating unit number β_N, as derived by Tsuji *et al.* under the Hückel approximation,[42] is given by:

$$\beta_N = -2\ln\left(\frac{\alpha}{\beta}\right) = -2\ln\left(\frac{1}{BO} - 1\right) = 2\eta BLA - 2\ln\left(\frac{C_\alpha}{C_\beta}\right)$$

The above expressions indicate that zero BLA, and therefore $BO = 0.5$, results in zero decay in the semi log conductance plots, as seen experimentally for **C12**-**C16**. These relationships are also shown graphically in Supplementary Figure 105 for clarity.

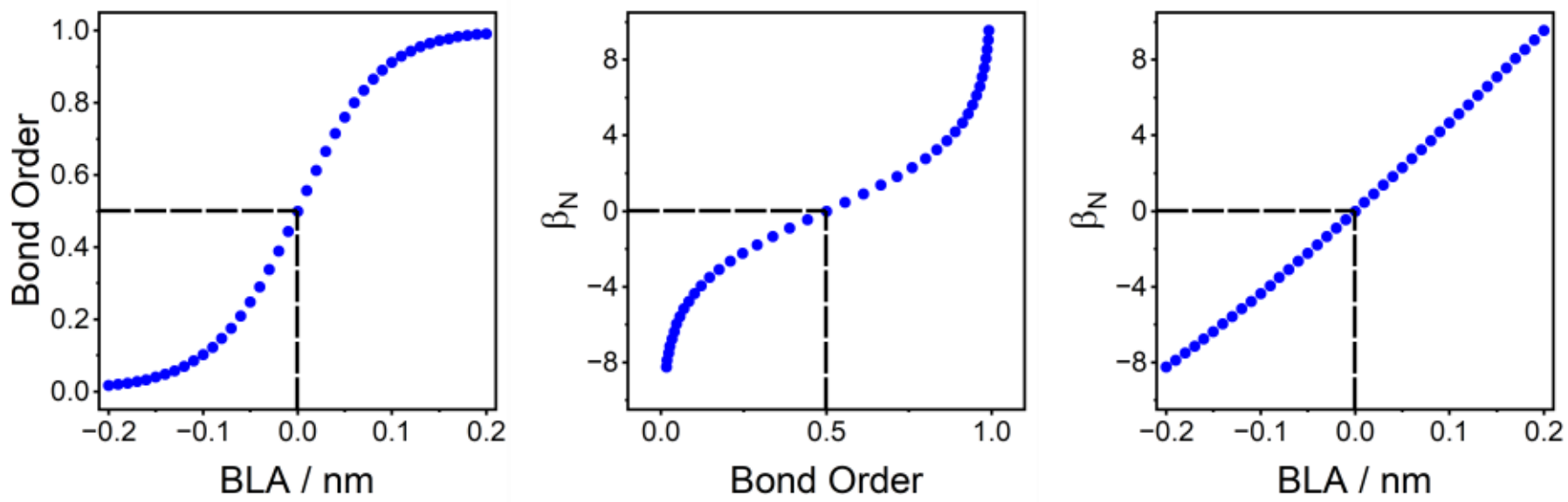

Supplementary Figure 105: Bond order as a function of BLA with a decay coefficient of $\eta = 29.6\ nm^{-1}$ (left). Tunnelling decay coefficient β_N with respect to repeating unit number N as a function of Bond Order (centre). β_N as a function of BLA (right).

4.1 Supporting Tight-Binding (Hückel) calculations

In order to explore other possible causes of the enhanced conductance trend in the main molecular wire series we modified the electrode molecule coupling by increasing the resonance (hopping) integral between the terminal C and the electrode from $t_{Au-C} = -0.4\ eV$ to $t_{Au-C} = -0.8\ eV$. We show the results in Supplementary Figure 106.

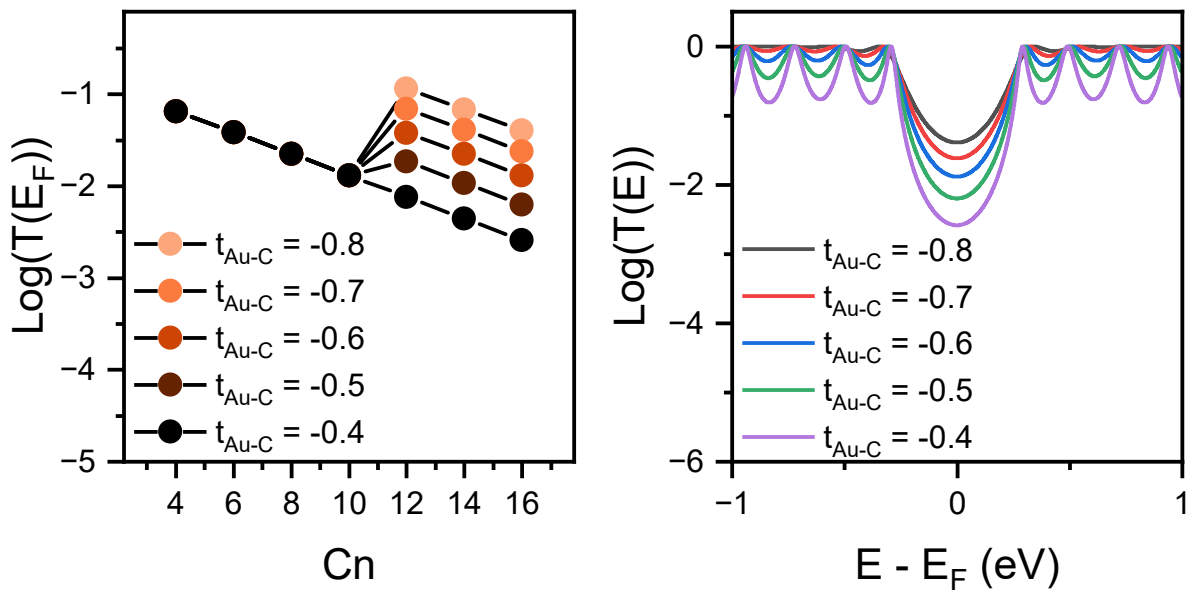

Supplementary Figure 106: The zero bias, zero temperature transmission as a function of carbon length Cn for various molecule to electrode hopping integrals (left). The transmission function of the C16 molecular wire at different molecule to electrode hoppings (right). Values for the dynamic hopping range from -0.4 eV to -0.8 eV.

It is clear that increasing the coupling to the electrode only raises the conductance across the series by a fixed offset but does not reduce the transmission attenuation with length after the initial increase. It is likely, therefore, that any changes in the molecule-electrode coupling across the series has a negligible direct effect on the conductance trend.

5. Density Functional Theory Calculations

Although tight-binding methods were used as the primary explanatory calculations due to their ease of interpretability, we also performed a series of DFT transport calculations. As mentioned in the main manuscript, DFT consistently underestimates changes in bond length alternation (BLA), especially in the presence of bulk electrodes. This is primarily because DFT cannot adequately model charge transfer between the electrode and molecule without sufficiently large basis sets and, often, semi-empirical (*e.g.* hybrid or, preferably split hybrid) functionals. Nevertheless, we attempted to calculate transmission across **C10** (pre-transition) and **C16** (post-transition) between the two Au(111) electrodes (Supplementary Figure 107).

First, we performed DFT-NEGF calculations[43] using the Perdew-Burke-Ernzerhof (PBE) implementation[44] of the Generalised Gradient Approximation (GGA) with a double zeta (DZ) basis set[45] and the Grimme DFT-D3 empirical dispersion correction to account for non-covalent interactions with the QuantumATK (version W-2024.09) software package.[46] The electrostatic potential was determined using a parallel conjugate gradient solver with periodic boundary conditions (PBC) in the transverse directions and Dirichlet boundary conditions (DBC) in the transport direction. The initial atomic configuration was relaxed to its minimum energy state. To simulate bulk stability, atoms in the outermost source and drain regions of the central supercell were fixed. The transmission spectrum was evaluated with an energy step of 0.05 eV.

As expected, Supplementary Figure 107c shows the inverse trend to the experiment, *i.e.* conductance decreasing from **C10** to **C16**, likely due to overestimation of BLA in **C10** ($7.9 \pm 0.3\ pm$) and **C16** ($7.0 \pm 0.3\ pm$).

We then ran a second geometry optimisation of the molecules terminated on both ends with Au_2 (to form a minimum closed shell system) in the ORCA software package[47–49] using the ωB97X functional with the Grimme's D3 dispersion correction[50] and the def2-TZVPPD (C) and def2-TZVPP (Au) basis sets[51–53] (using Stuttgart-Dresden effective core potentials)[54] with an energy change convergence threshold of $1 \times 10^{-9}\ E_h$ and the 'defgrid3' level for the numerical integration grid size. The molecular orbital (MO) diagram (Supplementary Figure 107a) shows only a small decrease in the HOMO-LUMO gap with no significant change in BLA between **C10** ($1.8 \pm 0.3\ pm$) and **C16** ($1.9 \pm 0.3\ pm$), indicating that any change to the BLA is not accurately modelled

without the presence of large Au electrodes. This, combined with the previous PBE calculations imply much higher levels of theory and large Au slabs would be required to accurately capture the experimental behaviour.

Despite this poor performance, we sandwiched the optimised structure between the same Au(111) slabs, and the electrode tips were allowed to relax using the PBE/DZ method described above while the molecular geometry was kept fixed. The transmission function was again calculated in Supplementary Figure 107d at the PBE/DZ level of theory. This led to an opening of the HOMO-LUMO gap relative to the Au_2-**Cn**-Au_2 MO calculations. Since the PBE implementation of DFT generally *underestimates* HOMO-LUMO gaps we attribute this widening to the PBE geometry optimisation of the terminal Au atoms in the junction. Regardless, this method also failed to realise the experimental observations in conductance between **C10** and **C16**, despite the large decrease in BLA compared to the full PBE method.

To confirm that the discrepancy between experiment and theory was because of the geometry optimisation procedures rather than issues within the mean-field Hamiltonian calculation itself, we further calculated the transmission spectra for the previous structure (molecule: ωB97X-D3, electrode: PBE) using the self-consistent-extended Hückel Theory (SC-eHT) method in Supplementary Figure 107e. Again, this method led to the inverse trend being observed compared to experiment. Because of this and considering the computational intractability of running a full geometry optimisation with sufficient basis sets/functionals on the full molecule-electrode system, we instead rely on the tight-binding model above to rationalise the experimental observations.

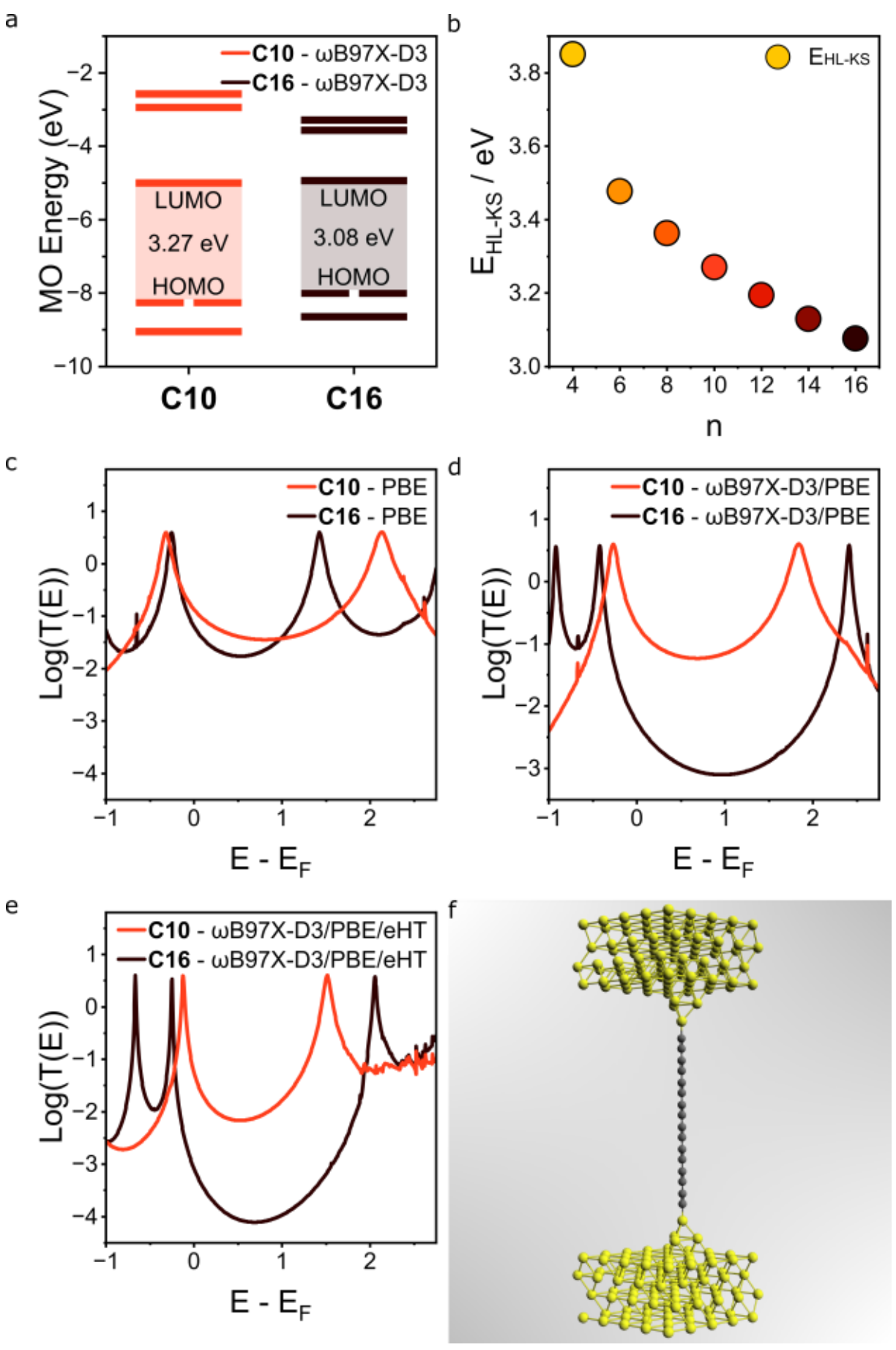

Supplementary Figure 107: Molecular Orbital energies (a) for **C10** and **C16** terminated with Au_2 dimers calculated by DFT (ωB97X-D3) and (b) the HOMO-LUMO gap as determined from the Kohn-Sham orbital levels. DFT-NEGF transport calculation with full PBE (c), mixed PBE/ωB97X-D3 (d), and hybrid

PBE/ωB97X-D3/eHT (e). A depiction of the scattering zone used in our simulations can be seen in panel (f).

Nevertheless, the DFT calculations have proven useful in demonstrating that the HOMO-LUMO gap is relatively featureless, void of signatures of “contact” or “gateway” states observed in other covalent electrode interfaces such as Au-thiolate[55,56] or direct Au-C*sp3*,[57] that could in principle account for the high conductance observed in these compounds.

6. Literature Survey

To further support our claim of these **Cn** devices delivering exceptional charge transport efficiency, we propose here a comparison with the literature of other systems displaying conductance values in excess of $0.1\ G_0$

Supplementary Table 8: Comparison between this work and other structures displaying exceptional charge transport efficiency (*i.e.* $G > 0.1\ G_0$). For each family of molecular wires, only the example displaying the highest conductance is discussed. Experimental junction lengths do not include electrode snapback.

**Molecular Wires**	**Experimental Junction Length**	**Max Conductance (Bias)**<br>**Max Current**	**Reference**	**Notes**
**C16 (this work)**	~ 2 nm	$\sim 0.6\ G_0$<br>(1 V)<br>$\sim 48\ \mu$A	--	--
**1,4-dimethylbenzene**	N/A	$\sim 0.9\ G_0$<br>(25 mV)<br>$\sim 1.7\ \mu A$	Venkataraman, Breslow and Hybertsen[58]	Junctions fabricated by transmetallation of α,ω-bis(trimethyltin) precursors, to deliver Au-C*sp*[3] electrode interfaces. Experimental junction length could not be determined as it proved impossible to isolate the length of the molecular plateau from that of the Au-Au contact.
**Porphyrin Tapes (8-mer)**	~4 nm	$0.3 - 0.8\ G_0$<br>(5 mV)<br>$\sim 0.3\ \mu A$	Leary and Anderson[59]	Molecule must be charged to a transient high-conductance state before high-efficiency transport is attained. Evidence of ballistic transport ($G \approx G_0$) in a small fraction of the dataset.
**Open-Shell Donor-Acceptor Oligomers (~11-mer)**	~10 nm	$\sim 0.4\ G_0$<br>(200 mV)<br>$\sim 6.5\ \mu A$	Wang, Azoulay and Franco[60]	Exact structure unknown. Sample is a mixture of oligomers. Even higher transport efficiency can be attained *via* mechanical modulation.
**Bis(triarylamine) diradical dications (terphenyl derivative)**	~1 nm	$\sim 0.1\ G_0$<br>(750 mV)<br>$\sim 5.8\ \mu A$	Venkataraman, Yin, Campos and Evers[61]	Close-shell material oxidised *in situ* to its high-conductance diradical dicationic state.

7. Supporting Information References

1. Bock, S. & Low, P. J. A Safe and Simple Synthesis of 1,4-Bis(trimethylsilyl)buta-1,3-diyne. *Aust. J. Chem.* **71**, 307–310 (2017).
2. Gückel, S. *et al.* Iron Versus Ruthenium: Evidence for the Distinct Differences in the Electronic Structures of Hexa-1,3,5-triyn-1,6-diyl-bridged Complexes [Cp*(dppe)M]{μ-(C≡C)3}{M(dppe)Cp*}]+ (M = Fe, Ru). *Organometallics* **40**, 346–357 (2021).
3. Bruce, M. I., Low, P. J., Werth, A., Skelton, B. W. & White, A. H. Some transition-metal complexes derived from silylated 1,3-diynes. *J. Chem. Soc. Dalton Trans.* 1551–1566 (1996).
4. Eisler, S. *et al.* Polyynes as a Model for Carbyne: Synthesis, Physical Properties, and Nonlinear Optical Response. *J. Am. Chem. Soc.* **127**, 2666–2676 (2005).
5. Anthony, J. *et al.* Tetraethynylethenes: Fully cross-conjugated π-electron chromophores and molecular scaffolds for all-carbon networks and carbon-rich nanomaterials. *Helv. Chim. Acta* **78**, 13–45 (1995).
6. Kern, N., Hoffmann, M., Blanc, A., Weibel, J.-M. & Pale, P. Gold(I)-Catalyzed Rearrangement of N-Aryl 2-Alkynylazetidines to Pyrrolo[1,2-a]indoles. *Org. Lett.* **15**, 836–839 (2013).
7. Wipf, P. & Graham, T. H. Synthesis and hetero-Michael addition reactions of 2-alkynyl oxazoles and oxazolines. *Org. Biomol. Chem.* **3**, 31–35 (2005).
8. Kendall, J., McDonald, R., Ferguson, M. J. & Tykwinski, R. R. Synthesis and Solid-State Structure of Perfluorophenyl End-Capped Polyynes. *Org. Lett.* **10**, 2163–2166 (2008).
9. Schmidt, H. C. *et al.* Mixed-Valent Molecular Triple Deckers. *Angew. Chem. Int. Ed.* **57**, 11688–11691 (2018).
10. Naher, M. *et al.* Synthesis, Structure and Physical Properties of “Wire-like” Metal Complexes. *Organometallics* **39**, 4667–4687 (2020).
11. Voutyritsa, E. *et al.* Photochemical Functionalization of Heterocycles with EBX Reagents: C−H Alkynylation versus Deconstructive Ring Cleavage. *Chem. – Eur. J.* **26**, 14453–14460 (2020).
12. Dolomanov, O. V., Bourhis, L. J., Gildea, R. J., Howard, J. A. K. & Puschmann, H. OLEX2 : a complete structure solution, refinement and analysis program. *J. Appl. Crystallogr.* **42**, 339–341 (2009).
13. Sheldrick, G. M. SHELXT - Integrated space-group and crystal-structure determination. *Acta Crystallogr. A* **71**, 3–8 (2015).
14. Sheldrick, G. M. Crystal structure refinement with SHELXL. *Acta Crystallogr. Sect. C Struct. Chem.* **71**, 3–8 (2015).
15. Harper, M. J., Emmett, E. J., Bower, J. F. & Russell, C. A. Oxidative 1,2-Difunctionalization of Ethylene via Gold-Catalyzed Oxyarylation. *J. Am. Chem. Soc.* **139**, 12386–12389 (2017).

16. Glemser, O. & Sauer, H. SECTION 19 - Copper, Silver, Gold. in *Handbook of Preparative Inorganic Chemistry (Second Edition)* (ed. Brauer, G.) 1003–1066 (Academic Press, 1965). doi:10.1016/B978-0-12-395591-3.50005-4.
17. Strasser, C. E., Cronje, S., Schmidbaur, H. & Raubenheimer, H. G. The preparation, properties and X-ray structures of gold(I) trithiophosphite complexes. *J. Organomet. Chem.* **691**, 4788–4796 (2006).
18. Walton, D. R. M. & Waugh, F. Friedel-crafts reactions of bis(trimethylsilyl)polyynes with acyl chlorides; a useful route to terminal-alkynyl ketones. *J. Organomet. Chem.* **37**, 45–56 (1972).
19. Milan, D. C. *et al.* Solvent Dependence of the Single Molecule Conductance of Oligoyne-Based Molecular Wires. *J. Phys. Chem. C* **120**, 15666–15674 (2016).
20. Fatiadi, A. J. Active Manganese Dioxide Oxidation in Organic Chemistry - Part I. *Synthesis* **1976**, 65–104 (1976).
21. Cahiez, G. *et al.* Manganese Dioxide. in *Encyclopedia of Reagents for Organic Synthesis* 1–16 (John Wiley & Sons, Ltd, 2017). doi:10.1002/047084289X.rm021.pub4.
22. DeCicco, R. C., Black, A., Li, L. & Goroff, N. S. An Iterative Method for the Synthesis of Symmetric Polyynes. *Eur. J. Org. Chem.* **2012**, 4699–4704 (2012).
23. Millar, D., Zakharov, L. N., Rheingold, A. L. & Doerrer, L. H. Aurophilic interactions in μ-p-phenyl-enediethynyl-bis[(trimethyl phosphite)gold(I)] dichloro-methane hemisolvate. *Acta Crystallogr. C* **61**, m90–m92 (2005).
24. Zirzlmeier, J. *et al.* Optical gap and fundamental gap of oligoynes and carbyne. *Nat. Commun.* **11**, 4797 (2020).
25. Wu, C. *et al.* A Chemically Soldered Polyoxometalate Single-Molecule Transistor. *Angew. Chem. Int. Ed.* **59**, 12029–12034 (2020).
26. Naghibi, S. *et al.* Redox-Addressable Single-Molecule Junctions Incorporating a Persistent Organic Radical. *Angew. Chem. Int. Ed.* **61**, e202116985 (2022).
27. Daaoub, A. *et al.* Not So Innocent After All: Interfacial Chemistry Determines Charge-Transport Efficiency in Single-Molecule Junctions. *Angew. Chem. Int. Ed.* **62**, e202302150 (2023).
28. Ward, J. *et al.* A Systematic Study of Methyl Carbodithioate Esters as Effective Gold Contact Groups for Single-Molecule Electronics. *Angew. Chem. Int. Ed.* **63**, e202403577 (2024).
29. Millar, D., Venkataraman, L. & Doerrer, L. H. Efficacy of Au−Au Contacts for Scanning Tunneling Microscopy Molecular Conductance Measurements. *J. Phys. Chem. C* **111**, 17635–17639 (2007).
30. Makk, P. *et al.* Correlation Analysis of Atomic and Single-Molecule Junction Conductance. *ACS Nano* **6**, 3411–3423 (2012).

31. Sil, A. *et al.* Mechanical Manipulation of Quantum Interference in Single-Molecule Junctions. *Small* **20**, 2308865 (2024).
32. Sil, A. *et al.* Single-Molecule Mechanoresistivity by Intermetallic Bonding. *Angew. Chem. Int. Ed.* **64**, e202418062 (2025).
33. Qiao, X. *et al.* Single-Molecule Junction Formation in Deep Eutectic Solvents with Highly Effective Gate Coupling. *J. Phys. Chem. C* **127**, 12802–12810 (2023).
34. He, H. X., Shu, C., Li, C. Z. & Tao, N. J. Adsorbate effect on the mechanical stability of atomically thin metallic wires. *J. Electroanal. Chem.* **522**, 26–32 (2002).
35. French, W. R., Iacovella, C. R. & Cummings, P. T. The Influence of Molecular Adsorption on Elongating Gold Nanowires. *J. Phys. Chem. C* **115**, 18422–18433 (2011).
36. Murai, D. *et al.* Highly stable Au atomic contacts covered with benzenedithiol under ambient conditions. *Phys. Chem. Chem. Phys.* **16**, 15662–15666 (2014).
37. Liu, M., Artyukhov, V. I., Lee, H., Xu, F. & Yakobson, B. I. Carbyne from First Principles: Chain of C Atoms, a Nanorod or a Nanorope. *ACS Nano* **7**, 10075–10082 (2013).
38. Sil, A. *et al.* Zero-Bias Anti-Ohmic Behaviour in Diradicaloid Molecular Wires. *Angew. Chem. Int. Ed.* **63**, e202410304 (2024).
39. Zang, Y. *et al.* Resonant Transport in Single Diketopyrrolopyrrole Junctions. *J. Am. Chem. Soc.* **140**, 13167–13170 (2018).
40. Gao, Y. *et al.* Length-Dependent Conduction of Polyynes: Searching for the Limit of the Tunneling Regime. *J. Am. Chem. Soc.* **147**, 4052–4059 (2025).
41. Clementi, E. & Raimondi, D. L. Atomic Screening Constants from SCF Functions. *J. Chem. Phys.* **38**, 2686–2689 (1963).
42. Tsuji, Y., Movassagh, R., Datta, S. & Hoffmann, R. Exponential Attenuation of Through-Bond Transmission in a Polyene: Theory and Potential Realizations. *ACS Nano* **9**, 11109–11120 (2015).
43. Brandbyge, M., Mozos, J.-L., Ordejón, P., Taylor, J. & Stokbro, K. Density-functional method for nonequilibrium electron transport. *Phys. Rev. B* **65**, 165401 (2002).
44. Perdew, J. P., Burke, K. & Ernzerhof, M. Generalized Gradient Approximation Made Simple. *Phys. Rev. Lett.* **77**, 3865–3868 (1996).
45. Smidstrup, S. First-principles Green's-function method for surface calculations: A pseudopotential localized basis set approach. *Phys. Rev. B* **96**, (2017).
46. Smidstrup, S. *et al.* QuantumATK: an integrated platform of electronic and atomic-scale modelling tools. *J. Phys. Condens. Matter* **32**, 015901 (2019).

47. Neese, F. The ORCA program system. *WIREs Comput. Mol. Sci.* **2**, 73–78 (2012).

48. Neese, F. Software Update: The ORCA Program System—Version 6.0. *WIREs Comput. Mol. Sci.* **15**, e70019 (2025).

49. Neese, F. Software update: The ORCA program system—Version 5.0. *WIREs Comput. Mol. Sci.* **12**, e1606 (2022).

50. Grimme, S., Antony, J., Ehrlich, S. & Krieg, H. A consistent and accurate ab initio parametrization of density functional dispersion correction (DFT-D) for the 94 elements H-Pu. *J. Chem. Phys.* **132**, 154104 (2010).

51. Rappoport, D. & Furche, F. Property-optimized Gaussian basis sets for molecular response calculations. *J. Chem. Phys.* **133**, 134105 (2010).

52. Weigend, F. Accurate Coulomb-fitting basis sets for H to Rn. *Phys. Chem. Chem. Phys.* **8**, 1057–1065 (2006).

53. Weigend, F. & Ahlrichs, R. Balanced basis sets of split valence, triple zeta valence and quadruple zeta valence quality for H to Rn: Design and assessment of accuracy. *Phys. Chem. Chem. Phys.* **7**, 3297–3305 (2005).

54. Andrae, D., Häußermann, U., Dolg, M., Stoll, H. & Preuß, H. Energy-adjustedab initio pseudopotentials for the second and third row transition elements. *Theor. Chim. Acta* **77**, 123–141 (1990).

55. Sangtarash, S. *et al.* Gateway state-mediated, long-range tunnelling in molecular wires. *Nanoscale* **10**, 3060–3067 (2018).

56. Li, C. *et al.* Charge Transport in Single Au | Alkanedithiol | Au Junctions: Coordination Geometries and Conformational Degrees of Freedom. *J. Am. Chem. Soc.* **130**, 318–326 (2008).

57. Cheng, Z.-L. *et al.* In situ formation of highly conducting covalent Au–C contacts for single-molecule junctions. *Nat. Nanotechnol.* **6**, 353–357 (2011).

58. Chen, W. *et al.* Highly Conducting π-Conjugated Molecular Junctions Covalently Bonded to Gold Electrodes. *J. Am. Chem. Soc.* **133**, 17160–17163 (2011).

59. Deng, J.-R., González, M. T., Zhu, H., Anderson, H. L. & Leary, E. Ballistic Conductance through Porphyrin Nanoribbons. *J. Am. Chem. Soc.* **146**, 3651 (2024).

60. Shen, S. *et al.* Long-Range Resonant Charge Transport through Open-Shell Donor–Acceptor Macromolecules. *J. Am. Chem. Soc.* **147**, 20310–20317 (2025).

61. Li, L. *et al.* Highly conducting single-molecule topological insulators based on mono- and di-radical cations. *Nat. Chem.* **14**, 1061–1067 (2022).
